# Compounded wind gusts and maximum temperature via semiparametric copula in the risk assessments of power blackouts and air conditioning demands for major cities in Canada

**DOI:** 10.1038/s41598-024-65413-6

**Published:** 2024-07-01

**Authors:** Shahid Latif, Taha B. M. J. Ouarda

**Affiliations:** Canada Research Chair in Statistical Hydro-Climatology, Institut National de la Recherche Scientifique, Centre Eau Terre Environnement, INRS-ETE, 490 de la Couronne, Québec City, QC G1K 9A9 Canada

**Keywords:** Wind gust speed, Maximum temperature, Compound events, Semiparametric copula density, Gaussian kernel density estimation, Joint distribution, Conditional distribution, Return period, Climate sciences, Natural hazards, Mathematics and computing

## Abstract

A semiparametric copula joint framework was proposed to model wind gust speed (WGS) and maximum temperature (MT) in Canada, using Gaussian kernel density estimation (GKDE) with parametric copulas. Their joint probability estimates allow for a better understanding of the risk of power blackouts and the demand for air conditioning in the community. The bivariate framework used two extreme sample groups to define extreme pairs at different time lags, i.e., 0 to ± 3 days, annual maximum WGS (AMWGS) and corresponding MT and annual highest MT (AHMT) and corresponding WGS. A thorough model performance comparison indicated that GKDE outperformed the parametric models in defining the marginal distribution of selected univariate series. Significant positive correlations were observed among extreme pairs, except for Calgary and Halifax stations, with inconsistent correlation variations based on selected cities and lag time. Various parametric 2-D copulas were selected to model the dependence structure of bivariate pairs at different time lags for selected stations. AMWGS or AHMT events, when considered independently, would be stressful for all stations due to high estimated quantiles with low univariate RPs. The bivariate events exhibited lower AND-joint RPs with moderate to high design quantiles, indicating a higher risk of power blackouts and heightened air-conditioning demands, which varied inconsistently with time lags across the station. The bivariate AMWGS and corresponding MT events would be stressful in Regina, Quebec City, Ottawa, and Edmonton, while AHMT and corresponding WGS events in Toronto, Regina, and Montreal. Conversely, Vancouver poses a lower risk of joint action of pairs AHMT and corresponding WGS events. These hazard statistics can help in better planning for community well-being during extreme weather.

## Introduction

Climate change (CC) intensifies extreme weather events, such as Heat waves, droughts, floods, etc., causing human health, economic, ecosystem and infrastructural damage globally^1–8^. Hotter-than-average conditions compromise the body’s temperature regulation and can cause heat-related illnesses like cramps, exhaustion, stroke, and hyperthermia^9–11^. It can cause long-term temperature variations, leading to sweltering weather (soaring temperatures and increased occurrence of heatwaves) and drastically increased air conditioning demands in homes and offices as people strive to keep cool^12–16^. Rising electricity demand challenges the energy industry as thermal power plants face limitations from elevated river temperatures and decreased water levels, leading to potential shutdowns^17–19^. Extreme temperatures can also cause disruptions in the distribution and supply of electric power. It can cause transmission lines to expand, sag or fail due to overloads or short circuits from ground contact. During the summer, Canada has experienced a surge in hot weather events, leading to significant loss of life ^20–23^. Rising summer temperatures in mild climate regions like Vancouver, Whitehorse, and Halifax have caused catastrophic events. Lytton, BC, evacuated after recording the highest-ever temperature of 49.6 ℃, causing a deadly wildfire^24,25^. Toronto broke a 78-year-old temperature record on May 31, 2022, with 32.2 ℃, beating 1944’s record of 31.1 ℃^26^. The hottest summer on record in Canada occurred in 2018 in the southern Quebec region, which was devastating^27^. Canada’s land temperatures have risen by 1.7 °C annually since 1948, with some regions (i.e., Northern British Columbia) experiencing even more significant increases ^28^. The Ottawa region in Canada is expected to have more hot days over 30℃ under a high emission scenario (RCP 8.5), as per the National Capital Region report^29^. Because of this, over 60% of Canadians now have air conditioning due to this increased temperature risks^30^, and an increase in cooling demand is predicted for the next 50 years^31^. Similarly, air conditioning demand in Canada’s Quebec province is expected to rise 7.5% by 2100, while winter heating demand may drop 15.3% compared to 2001^32^. Summer heat results in heightened air conditioning demands (HACDs), overloading electrical equipment, and may cause power outages. Communities in various provinces of Canada that depend on air conditioning are at significant risk. Maximum Temperature (MT) is a crucial parameter in assessing HACDs risk, referring to the highest temperature lasting 24 h. However, the combination of high temperatures and humidity can lead to worsened situations due to heat stress. Sweating is the human body’s natural way of cooling, but high temperatures and humidity can interfere with this mechanism. The present study focused on MT events as an indicator of public inconvenience in communities because of high air-conditioning demands. Humidity data were not available in this study area and were not considered. They may be included in a separate study if they become available.

In addition, Canada’s extreme wind gust speed (WGS) frequently causes electric power and telecom failures, making this parameter crucial. A recent example occurred on January 13, 2021, when coastal regions experienced severe winds with gusts of up to 100 km/h in Victoria and the eastern Fraser Valley. Vancouver International Airport also documented gusts of up to 91 km/h, which resulted in power blackouts (PBs) that impacted approximately 212,000 individuals^33^. Similarly, high wind gusts were recorded up to 74 km/h in Montreal, resulting in PBs in the Montreal region and other provinces of Quebec^34^. A power wind gust in Toronto on December 2, 2022, caused PBs across the Greater Toronto region in Canada^35^. Likewise, on January 19, 2021, massive wind gusts hit the Edmonton region in Canada between 87 and 107 km/h^36^. A WGS is a sudden, brief burst of high-speed wind lasting 20 s or less. Extreme WGS increases the risk of PBs, damaging transmission lines, electric poles, or transformers due to falling trees or structures. Also, future projections suggest that WGS events could increase in Canada later this century^37–39^.

About 40% of Canadian households chose to keep the temperature between 20 and 23 $^\circ{\rm C}$, while 9% preferred to keep their dwelling at 19 $^\circ{\rm C}$ or lower when at home and awake^40^. While about 33% of Canadian households preferred to sleep with temperatures between 20 and 23 ℃. Environment and Climate Change Canada has set a WGS threshold of 90–110 km/h, or more can cause structural wind damage. WGS ranging from 74 to 100 km/h have already caused severe impacts in Canada^41^. Temperature and wind speed are physically interrelated^42,43^. Higher temperature differences between two locations can lead to stronger winds as air moves from high-pressure to low-pressure areas, following ideal gas law (i.e., $\text{PV}=\text{nRT})$^44^. The difference between water and land temperature also affects winds. Besides, temperature fluctuations over land are more significant than over water^45^. Convection is another way in which wind facilitates the transfer of heat. Besides, increasing humidity would also exaggerate heat strain; under this climate change scenario, their joint action with temperature (or heat stress) would be much more stressful during summer^46^. Sustained wind speed and WGS differ only in duration. Longer gusts can exert more force and cause more significant damage.

The above review confirms that intense WGS events or elevated summer MT pose a significant threat to households and businesses in various Canadian provinces that rely on uninterrupted power supply. Also confirmed that there have been instances where communities have been affected due to the lack of power supply (due to extreme WGS events) during periods of high demand for air conditioning (such as elevated summer temperatures or heat waves). Developing contingency plans by investigating the probability distribution behaviour or frequency analysis (FA) by examining joint concordance between event magnitude (or design quantiles) and frequency of occurrence (or exceedance probability and returns) is crucial to mitigate potential risks^47^. Previous research pointed out the impact of elevated temperatures on electric power consumption through air cooling demands^48–50^. Nevertheless, few investigated the effect of severe WGS hazards on power disruption ^51,52^. To our knowledge, research efforts have yet to explore the joint probability distribution modelling and bivariate hazard risk evaluation of extreme WGS and MT events compounding to cause power outages when there is already a heightened air-conditioning demand in Canadian communities due to elevated summer temperatures. This study proposes a bivariate probabilistic framework to assess the joint occurrence risk of extreme WGS and MT events in Canadian communities for both primary joint and conditional joint hazard scenarios.

Considering common extremes shared between proxy variables when combining multiple extreme events is essential. Prior literature has explored various probabilistic models in the extremal dependence measures^53–55^. A consistent mathematical description of compounded events is needed to ensure accuracy and reliability in analyzing complex phenomena. The present study captures the complex interplay between summer MT and WGS events, requiring a more adaptable joint density model. Copula functions are more flexible joint distribution tools than traditional multivariate models and provide separate modelling of univariate marginal distributions and their dependence structures^56–58^. Most existing studies incorporated copula in the parametric setting, having prior statistical assumptions of their univariate marginal density and parametric-class copula joint density (Archimedean or Elliptical or Extreme value etc.) ^59–61^. Previous literature did not suggest using a fixed probability density function (PDF) to model the marginal structure. Samples from WGS or MT distribution would follow different distributions and must be modelled separately without imposing any restriction in any pre-defined or fixed distribution type. Nonparametric kernel density estimation (KDE) is a reliable data-driven approach with no prior distributional assumption and is especially suited for skewed or multimodal distributions^62–66^. KDE can approximate density more robustly than the parametric model by deriving numerical PDF directly from random samples (and have no explicit expression). Combining the parametric copula class with nonparametric KDE margins creates a semiparametric framework that can outperform the traditional parametric copula framework, which has already been proven in previous work^62,66^. The semiparametric copula joint framework can reduce the risk of misspecification when the statistical assumption underlying the univariate marginal distribution is violated.

The arguments presented above confirm that Canada’s provinces are experiencing a growing issue of power blackouts due to extreme wind gusts. This problem could further exacerbate the level of inconsistencies within communities when there are high cooling demands due to elevated summer temperatures or heat waves. The study was motivated by the joint risk of elevated temperatures and wind gust events during the summer period that increase the risk of power outages during high cooling demands. Moreover, this study introduced a methodological advancement in using highly flexible semiparametric copula joint dependency simulation for bivariate hazard modelling of WGS and MT events. This study examines the joint probability action by gathering extreme value sample groups (EVSGs), consisting of the extreme WGS and MT sample groups (see Method “Study area and data delineation”). Each group was defined based on specific bivariate events, including pairs of WGS and MT events. If MT (or WGS) event reaches their extreme value on a specific date, D, of any calendar year, and corresponding WGS (or MT) occurs on the same date or in close temporal proximity (or within specific time lags) from the occurrence of first events, it may result in PBs and HACDs simultaneously, leading to a stressful situation within the community. The extreme WGS sample groups were defined by considering the maximum WGS observed during the summer period for each calendar year; the corresponding MT events were defined simultaneously or in close succession, resulting in compound events. The extreme MT group comprised the highest annual MT and corresponding WGS events. The proposed semiparametric copula joint framework utilized different bivariate EVSG datasets collected for Canada’s major cities. In the research paper, the “Methods” section is dedicated to the methodology and divided into sub-sections. These sub-sections include the data selection and analysis approach to define extreme sample groups (EVSGs) and developing a semiparametric copula density with a nonparametric GKDE model. Section “Results and discussions” applies the developed models to analyze the joint (for both AND and OR hazard scenarios) and conditional joint risk for compounded WGS and MT events in selected cities. Section “Conclusion and future recommendations” concludes the study and includes recommendations for future research.

## Methods

### Study area and data delineation

The study focused on nine major Canadian cities across four geographical regions (see Supplementary Table ST1). Canada’s climate is diverse, ranging from arctic conditions in the north to continental in the south and sub-arctic in the central areas. The southwest coast of British Columbia (or B.C) experiences a cool and rainy oceanic climate, while regions along the east coast (or Atlantic Canada) have reduced sea influence due to cold currents. For instance, Vancouver City in B.C has dry and warm summers, but the rest of the year is rainy, particularly from October to March. Calgary, located in the Canadian Prairies, has a prairie-steppe-type climate and is quite windy due to its location. Edmonton has a humid continental climate (or dry) with frequent summer thunderstorms that can produce large hail, damaging winds, funnel clouds, and occasionally tornadoes. In Regina, summers are typically long, comfortable, and partly cloudy, while winters are characteristically frigid, snowy, windy, and mostly cloudy. Similarly, in the Central Canada region, Toronto’s summer humidity is high due to its proximity to Lake Ontario, other lakes, and faraway sources like the Gulf of Mexico. Quebec City has a continental climate with no dry season and a warm summer. Ottawa has a semi-continental climate with warm and humid summers and very cold winters. It has higher summer temperatures compared to other Ontario cities because it is not situated on the shore of Lake Ontario. Montreal also has a semi-continental climate, with a warm, humid summer and a very cold winter, where summers usually have a generous number of warm or hot sunny days. Finally, Halifax, located in the Atlantic Canada region, has a short, warm summer and a cold winter, with common disturbed and changeable weather throughout the year due to its eastern maritime climate.

The study analyzed daily-based long-term datasets of WGS and MT for Canada’s summer months (June to September) obtained from Environment and Climate Change Canada (https://climate.weather.gc.ca/). Both data series were selected for the same calendar date and for the same years. Summer heat increases air-cooling demands, and this could stress on power distribution or supply, while WGS raises power blackout risk within community. It may be more significant to observe both events (WGS and MT) at specific time lags or in close succession, such as ± 1, ± 2, or ± 3 days, rather than on the same date or concurrently (time lag of 0 day). We considered two different bivariate extreme value sample groups (EVSG) at each selected station (Supplementary Fig. SF1).

- The EVSG-1 consists of the highest WGS events observed, also called the annual maximum WGS (AMWGS) for each calendar year and the corresponding highest maximum temperature (MT) recorded within a time lag of 0, ± 1, ± 2, or ± 3 days from the date of AMWGS events.
- While the EVSG-2 datasets group consists of the annual highest MT (AHMT) events for each calendar year and the corresponding maximum WGS events observed on the same calendar date (Time lag of 0 day) and time lags of (± 1, ± 2, ± 3 days) from the date AHMT events.

The rationale behind the selection of different time lags (e.g., 0 to ± 3 days) used in this study in defining bivariate extreme random pairs is based on the fact that there were instances (refer to the literature review in the “Introduction”) where it was documented that severe wind gusting affected communities through power blackouts occurring 0, 1, or 2 days before or after extreme temperature events during the summer period. In other words, when there is a high demand for cooling due to extreme heat, a lack of power supply increase stress level. Therefore, the joint probability action of extreme WGS and corresponding MT events, or vice versa, occurring on the same calendar date or in close temporal proximity can be stressful within the community in the form of HACDs and PBs. The dependency strength was analyzed for bivariate dataset pairs obtained from EVSG-1/2 groups (as detailed in Sect. 3.1). Only those pairs of bivariate extreme (AMWGS-MT or AHMT-WGS) that demonstrated significant positive correlation were chosen from each selected station. These selected pairs were then utilized to develop bivariate copula density models to outline the joint action of given bivariate pairs, followed by performing bivariate joint risk assessments.

### Semiparametric copula joint density

**Figure 1:**
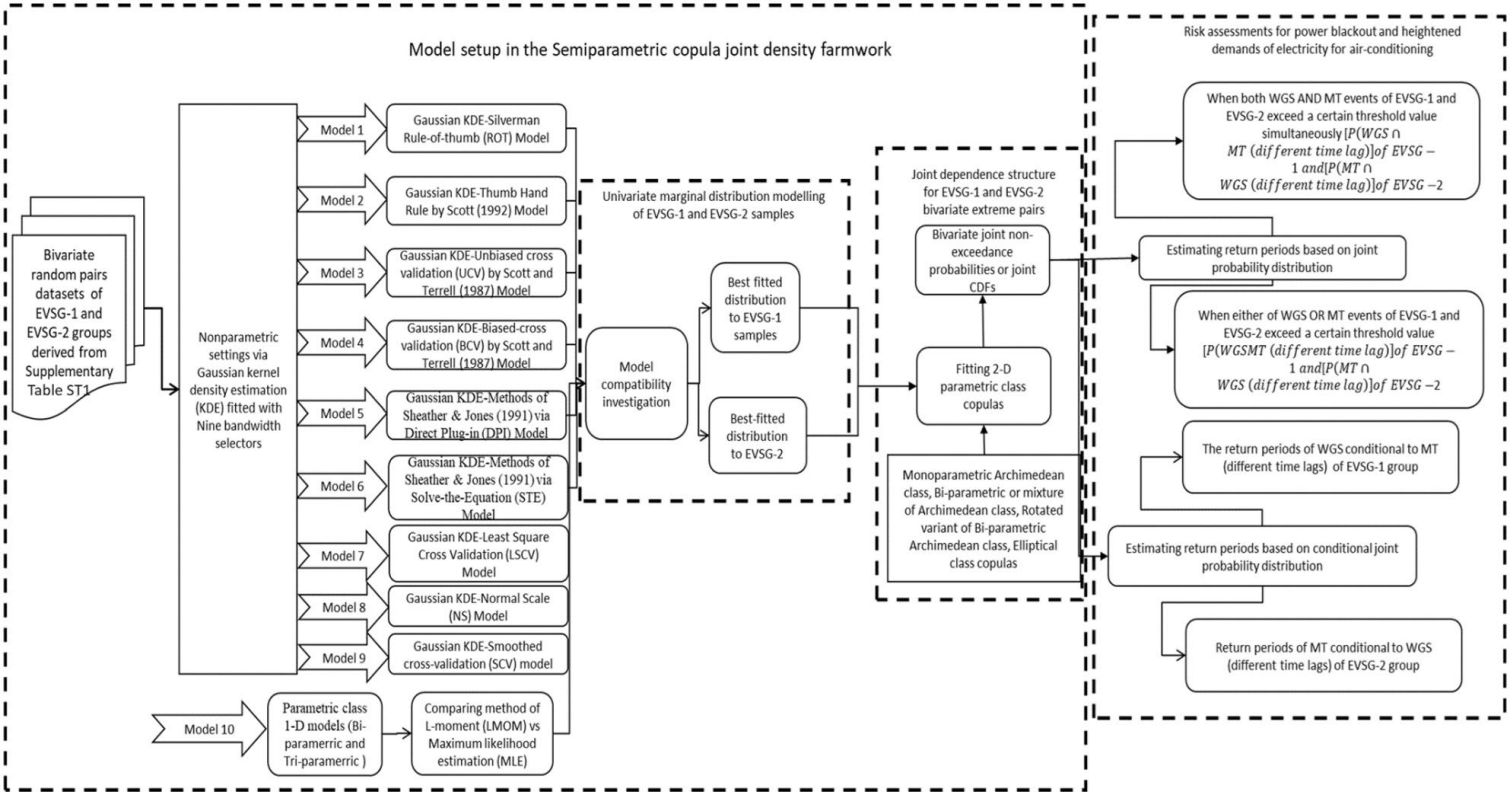
Methodological workflow in the bivariate risk modelling of compounded AMWGS-MT (and AHMT-WGS) events via semiparametric copula distribution framework.

The methodological approach of this study was divided into two phases as shown in Fig. 1. In the first phase, a bivariate extreme dependence model was developed by simulating a highly flexible copula joint density in the semiparametric setting. For this study, a distinct varieties of positively dependent 2-D parametric copula class functions were selected as a candidate model in describing most justifiable joint dependence of bivariate extreme pairs for each selected city (see Supplementary SA1). While the nonparametric distribution-free based marginal density via GKDE model was fitted with nine different bandwidth selector approach, absence of any prior assumption about the marginal pdf type and that allows for greater flexibility the developed model can reduce risk of misspecification that may occur when there is a violation in the selection of marginal density. In phase two, bivariate risk assessments were conducted for various hazard scenarios using semiparametric joint models. These models estimated exceedance probabilities (EPs) for both joint and conditional distribution cases, as well as primary joint (for both OR and AND hazard scenario) and conditional RPs. The joint probability action of the selected bivariate extreme pairs, AMWGS would be stressful within the community if exhibited lower AND-joint RPs at high design quantile values (or when it reaches high magnitude). The study highlighted the variation in estimated RPs between stations and even within the same station, to show how bivariate extreme pairs would contribute simultaneous PBs and HACDs. This helps us understand how the risk varies across different times and locations.

#### Bivariate copula joint dependence

Copula modelled bivariate joint cumulative distribution functions (CDFs) of two correlated observations says $({Z}_{1}, {Z}_{2})$, with continuous univariate marginal functions, say $F\left({z}_{1}\right), F\left({z}_{2}\right)$ and their unknown dependence parameter $\uptheta$, it can be expressed as^56,58,59^

1 $$\text{F}\left({\text{z}}_{1}, {\text{z}}_{2}\right)={\text{C}}_{\uptheta }(\text{F}\left({\text{z}}_{1}\right),\text{ F}\left({\text{z}}_{2}\right);\uptheta )$$

The 2-D Archimedean class copula is mathematically estimated by.

2 $$C\left({\text{z}}_{1}, {\text{z}}_{2}\right)={\Phi }^{-1}(\Phi {(\text{z}}_{1}), ({\text{z}}_{2})), \text{for} {\text{z}}_{1}, {\text{z}}_{2}\in [\text{0,1}]$$

where, $\Phi (\cdot )$ and ${\Phi }^{-1}(\cdot )$ are the generator function of selected Archimedean copulas and its inverse. For more statistical details and information on the selected copulas, including mono-parametric and mixed Archimedean copulas (and their rotated variant by 180 degrees, known as the survival copula), extreme value copulas, and elliptical copulas, please refer to Supplementary SA1. The unknown dependence parameters of candidate copulas for each station were estimated via the rank-based distribution, called the maximum pseudo-likelihood (MPL) estimation, which works by maximizing the pseudo-log-likelihood functions (see Supplementary SA2 (Eqs. 1, 2)). This approach estimated copula parameters without depending upon marginal distributions. The performance evaluation of fitted copulas was evaluated using the Cramer von Mises (CvM) functional statistics, ${S}_{n}$, with a parametric bootstrap procedure, taking N = 1000 bootstrapping samples (Supplementary SA3 (Eqs. 1–3)). Minimum value of CvM-based ${S}_{n}$ (with estimated p-value must be greater than 0.05 (at 5% significance level), must indicate optimal performance. A graphical-based visual inspection was conducted to examine the consistency of selected copulas. This involved overlapped 2-D scatterplots between historical bivariate random series and synthetically generated bivariate samples. The purpose was to demonstrate how effectively the selected bivariate copula density regenerates the natural dependency between historical bivariate datasets.

#### Marginal modelling of univariate series

The marginal distribution of AMWGS and corresponding MT of EVSG-1 (or AHMT and corresponding MT of EVSG-2) series would follow the different distributions and need to be modelled separately without imposing any fixed or predefined probability distribution family. The nonparametric KDE did not posses any prior assumptions about marginal probability density function (PDF) that exhibits higher flexibility compared to parametric models and can effectively model skewed distributions. In other words, parametric distributions assume that random samples come from the known population whose pdf is predefined. The combination of parametric copulas with KDE margins can approximate joint density much better than parametric margins. In this study, KDE was employed in approximating margins of AMWGS (or AHMT) and corresponding MT (WGS) events defined by EVSG-1/2 that can have the following statistical properties estimated by^63,64,67–71^

3 $${\int }_{-\infty }^{+\infty }\text{K}\left(\text{z}\right)\text{dz}=1$$

and,

4 $${\text{K}}_{\text{h}}\left(\text{z}\right)=\frac{1}{\text{h}}\text{K}(\text{z}/\text{h})$$

An appropriate kernel bandwidth ‘h’ must be selected for accurate KDE estimation that controls smoothness and roughness levels in the fitted Kernel density^67^. A very low h value can result in under-smoothing, while setting it too high leads to over-smoothing. The univariate KDE was obtained by taking the average of Eq. (4) and estimated by

5 $$\widehat{{{\text{f}}_{{\text{h}}} }}\left( {\text{z}} \right) = \frac{1}{{{\text{nh}}}}\sum\limits_{{{\text{i}} = 1}}^{{\text{n}}} {{\text{K}}_{{\text{h}}} \left( {\frac{{{\text{z}} - {\text{z}}_{{\text{i}}} )}}{{\text{h}}}} \right)}$$

This study compared the adequacy of nonparametric KDE models, including Gaussian KDE (GKDE), with nine different bandwidth selectors in describing WGS and MT margins. GKDE is often a popular choice and frequently accepted in nonparametric density modelling and can be mathematically estimated by^72,73^

6 $${\text{K}}\left( {\frac{{{\text{z}} - {\text{z}}_{{\text{i}}} )}}{{\text{h}}}} \right) = \frac{1}{{\sqrt {2{{\uppi}}} }}{\text{e}}^{{\left( { - \frac{{\left( {{\text{z}} - {\text{z}}_{{\text{i}}} } \right)^{2} }}{{2{\text{h}}^{2} }}} \right)}}$$

Nine different bandwidth selectors for GKDE were estimated; this includes Silverman’s Rule-of-Thumb (SiROT)^69^, Scott’s Rule-of-Thumb (ScROT)^74^, Solve-the-Equation (STE) and Direct-plug-in (DPI)^75^, Biased cross-validation (BCV) and Unbiased cross-validation (UCV)^76^, Normal scale (NS)^77^, Smooth cross-validation (SCV)^78,79^, and Least square cross-validation (LSCV)^80^ (Supplementary SA4 (Eqs. 1–6)). The performance of some frequently used parametric models, such as Gamma-2P, GEV-3P, Normal-2P and Logistic-2P, was also tested and fitted via the maximum likelihood estimation (MLE)^59,61^ and method-of-L-moments (LMOM)^81^ estimator. MLE can possess minimum sampling variance when estimating parameters and is also more effective in case of large sample size just because of its asymptotic optimal properties ^82^. In contrast, L-moments are the measures of the location, scale and shape of data samples and are based on the linear combination of order statistics.

The fitness consistencies of fitted 1-D models were investigated by comparing empirical probabilities^83^ with theoretical probabilities. Different goodness-of-fit (GOF) statistics were calculated for each fitted model: Mean square error (MSE)^84^, Akaike Information Criterion (AIC)^85^, Bayesian Information Criterion (BIC)^86^ and Hannan-Quinn Information Criterion (HQC)^87,88^ (Supplementary SA5 (Eqs. 1–3)). The minimum AIC, BIC, HQC, and MSE test values must indicate better performance.

### Bivariate risk assessments via joint and conditional probability estimates

The joint probability action of selected extreme pairs AMWGS-MT (or AHMT-WGS) would be stressful only if their quantile strength or magnitude surpasses a threat (specified threshold) level and possesses the lowest AND-joint RPs (or higher co-occurrence probabilities, also called AND-joint probabilities which indicated a higher exceedance in the simultaneous occurrence or more risky events). The selected bivariate densities (for each selected station and for each time lag) were employed in estimating joint probability density function (PDF) and joint cumulative distribution function (CDF) and was used further in evaluating their associated RP for joint (bivariate OR and AND hazard scenario) and conditional distribution ^2,59,61,82,83^. The RP represents the average inter-arrival between two consecutive occurrences of extreme events. Different notations of probability distribution scenarios were considered to analyze the joint risk (Supplementary SA6, Eqs. 1–6). This study followed and utilized the full bivariate copula joint in estimating bivariate joint RPs, which is an intuitive extension of the univariate RP approach.

The AND-joint distribution highlights the bivariate risk when both events simultaneously exceed a specific threshold value (i.e., AMWGS AND MT, in case of EVSG-1 datasets) (or AHMT AND WGS, in case of EVSG-2 group). While OR-joint distribution and their associated RPs outlined the joint risk when either of the events exceeded the specified threshold (i.e., AMWGS OR MT) (or AHMT OR WGS). Besides, this study also established a conditional joint framework for investigating the bivariate joint risk of AMWGS events given various percentile values of corresponding MT events observed at different time lags. Additionally, also examined the joint RP of AHMT event conditional on WGS events. The conditional joint RP were estimated for two different joint situations, (1) defined bivariate joint RP of AMWGS given various percentile value of MT events for different time lag (say 0 to ± 3 days), (2) defined bivariate joint RP of AHMT given various percentile value of WGS events for time lag, say 0 to ± 3 days (see Supplementary SA6).

## Results and discussions

### Dependency measures for bivariate extreme pairs

The correlation strength of extreme pairs was analyzed using analytical (i.e., Pearson correlation (r), Kendall’s tau ($\tau )$, and Spearman rho ($\rho )$ coefficients) and graphical inspection (i.e., Chi-plot^89^, Kendall’s (or K) plot^90^, and 2-D scatterplots^90^). Kendall’s and Spearman’s coefficients are better with outliers and invariant under monotonic nonlinear transformations with no distributional assumption^82^. Here, following results were discussed obtained for each station (refer to Supplementary ST2a–i & Supplementary SF2a-1, a-2 to i-1, i-2).

- In Montreal, EVSG-1-based AMWGS-MT pair exhibited positive correlations that vary inconsistently across time lags, with the highest correlation was at 0 day. The Chi, Scatter, and Kendall plots confirm correlation strength, as some pairs are outside the control limit region (for Chi-plot) and datasets deviate from the main diagonal (for K-plot). At the same station, only the pair AHMT-WGS of EVSG-2 showed a significant positive correlation with a lag of 0 days.
- In both Quebec City and Ottawa, significant positive correlations were found for pair AMWGS-MT at all selected time lag and was inconsistently varying. The strongest correlation for Ottawa was observed at lag ± 1 days, while, at lag of 0 day for Quebec City.
- Positive correlation was observed only for EVSG-2 between AHMT-WGS pair at Toronto with a time lag of 0 to ± 3 days, stronger dependency at ± 2 days.
- There was a consistent decrease in positive dependency observed for EVGS-1/2 at Regina as the time lag increased. The Edmonton exhibited positive correlations only for EVSG-1 pair AMWGS-MT at 0 and ± 1 days, while no significant correlations were found in the Calgary for both EVSG-1/2 pairs.
- Vancouver City had a positive correlation only for EVSG-2 AHMT-WGS pair at all selected time lags, with stronger concurrency at ± 1 days. Conversely, no significant correlations were identified for Halifax.

The degree of mutual concurrency varied inconsistently across different lags for most selected station. Some station showed maximum concurrency when events occurred concurrently (i.e., Montreal or Quebec or Regina), while for other, it was with a time lag of ± 1 days (i.e., Ottawa), or ± 2 days (i.e., Toronto) etc. Only the bivariate pairs that showed significant correlation were selected in the joint extreme modelling, excluding Calgary and Halifax. Box-whisker and Histogram distribution plots were used to illustrate the statistical behavior of the selected extreme pair datasets of EVSG-1/2, which can be found in Supplementary SF3a–g and SF4.1a–g. Besides, Supplementary SF4.2a–g, time series plots of selected univariate series for EVSG-1 and EVSG-2 datasets are illustrated.

### Marginal distribution modelling of EVSG-1/2 datasets

Supplementary ST3a–g summarizes each nonparametric GKDE model’s estimated bandwidth and parametric model distribution parameters. Supplementary ST4a–g summarises the most justifiable marginal distribution models for each station. Supplementary ST5a–g compared the fitness consistency of GOF performed for selected nonparametric versus parametric models. The study found that the nonparametric GKDE model was more effective than the parametric class distribution in describing the marginal behaviour of the selected EVSG-1/2 datasets at all selected stations. All selected models exhibited minimum values of AIC, BIC, HQC and MSE GOF statistics. Additionally, there was variability in the selected marginal model between different stations or either, in between time lags for the same stations. For example, the GKDE-SiROT and GKDE-LSCV models were the best fit for selected EVSG-1datasets (i.e., AMWGS and corresponding MT at different time) in Montreal and Quebec City. Meanwhile, at Regina, the GKDE-SiROT model was the best fit for the marginal of AMWGS and corresponding MT with lag ± 1 days, the GKDE-UCV model with 0 & ± 3 days, and the GKDE-STE with ± 2 days. GKDE-SiROT models were most justifiable in describing marginal distribution at Ottawa and Edmonton. Selected EVSG-2 datasets at Toronto were used GKDE-SiROT and GKDE-UCV models, while GKDE-LSCV, GKDE-STE, and GKDE-UCV models were used at Vancouver City. This distribution-free margins can reduce the risk of misspecification if an inappropriate parametric class density is selected with the prior assumption in their marginal pdf. Supplementary SF5a–g showed probability density plots that confirmed the effectiveness of selected marginal models. Recognized margins were integrated with parametric copula to semiparametric joint density.

### Copula joint density for extreme pairs

This study thoroughly compared the adequacy of different positively dependent parametric class 2-D copulas fitted via MPL estimator (refer to Method Sects. 2.2.1, Supplementary SA1, SA2 & SA3, Supplementary ST6a–g) to established bivariate dependence for AMWGS-MT and AHMT-WGS. Besides Supplementary SF6a–h illustrated the overlapped 2-D scatterplots between bivariate observed extreme pairs (red) and a set of 1000 random samples simulated from the best-fitted 2-D copulas (light blue) for each bivariate extreme pair. There was an interstation variability in the copulas type selected and/or in between different time lags for same station.

It was discovered that the Tawn type-1 copula was the most appropriate density for bivariate pairs AMWGS-MT (Time lag = 0, ± 1 & ± 3 days) in Montreal. Conversely, the BB7 copula for AMWGS-MT (Time lag =  ± 2 days) and AHMT-WGS (Time lag = 0 day). The selected copulas were exhibited lowest S_n_ and its estimated p-value was above 0.05 (measured at a 5% significance level). In addition, the 2-D scatterplot confirmed they accurately replicated the historical observations’ natural concurrency for bivariate extreme pairs. In addition, the chosen copulas (Tawn type-1 and BB7) effectively captured upper tail dependence structures, highlighting mutual concurrency for extreme pairs. Likewise, at the Quebec City, different copulas were work best in joint density of AMWGS-MT pairs at varying time lags. For instance, BB8 copulas (at 0 day), Survival BB8 (at ± 1 days), Survival Clayton copula (at ± 2 days) and Survival BB7 (at ± 3 days). In Ottawa, Normal copula was best for pair AMWGS-MT at 0 day, while Survival BB1 and Survival BB7 were best at ± 1, ± 2 and ± 3 days, indicating inter-lag variability in the copula describing joint correlation behaviour at the same stations. However, both Survival BB7 and Survival BB1 copulas effectively captured both upper and lower tail dependence. While at Toronto, the Tawn type-1 and BB7 copulas performed optimum and best described the joint dependence for pair AHMT-WGS at 0 days and ± 1 to ± 3 days.

Meanwhile, different copula classes were identified for selected bivariate extreme pairs of EVSG-1 and EVSG-2 datasets in Regina. For AMWGS-MT pair of EVSG-1, BB8 was the most optimum copula at lag 0 & ± 1 days, Plackett copula at ± 2 days, and Survival Tawn type-1 copula at ± 3 days. Similarly, for AHMT-WGS pair of EVSG-2, Normal, AMH, and Survival Tawn type-1 copulas were justifiable at lag 0 & ± 2 days, ± 1 days, and ± 3 days. Optimum performance for AMWGS-MT pairs was observed at a lag of 0 and ± 1 days using the Plackett and Survival BB1 copula at the Edmonton. Conversely, at Vancouver City, Normal and Clayton copula described joint dependence of bivariate events AHMT-WGS at lag 0 and ± 3 days, while Survival Tawn type-1 copula was at ± 1 & ± 2 days. The 2-D copula density was integrated with the most efficient marginal densities to estimate bivariate joint CDFs and PDFs. The analysis further examined bivariate hazard modelling for joint and conditional risk scenarios in the following Sects. 3.4 and 3.5. Supplementary SF7a–g were depicted station-wise surface density plots, while Supplementary SF8a–g illustrated 3-D scatterplots of the joint PDF created using 1000 random samples from the best-fitted copula density.

### Bivariate risk evaluations via joint probability distribution

According to the threshold mentioned in NOAA^91^, a WGS above 58 mph (93.34 km/h) can be extremely dangerous for human life and property, while, between 40 and 57 mph (64.37–91.73 km/h) are considered high-risk, between 35 and 57 mph (56.32–91.73 km/h) are moderately threatening, and between 30 and 35 mph (48.28–56.32 km/h) fall under the low-threat level category. The study examined the joint impact of extreme pairs AMWGS-MT (or AHMT-WGS) by analyzing primary joint RPs for OR and AND hazard scenarios (see Supplementary SA6, Table 1a–g and Method Sect. 2.3). In the AND hazard scenario, the lower AND-joint RPs must be indicated for higher concurrence probability or joint exceedance probability in the simultaneous occurrence of compounded events. At the same time, when these bivariate events are defined with higher design quantile values at lower AND-joint RP (highly probable events), it could be stressful. It may severely impact the community through higher power demands for air conditioning with the risk of power failure. Furthermore, Supplementary SF9a–g were depicted the bivariate RPs for both OR and AND hazard scenarios for the historical extreme pair observed at selected stations. Similarly, Supplementary Fig. SF10a–g illustrated the bivariate joint CDFs (or joint NEP) of the same datasets. Finally, Supplementary Fig. SF11a–g depicted synthetically generated bivariate events using the generated semiparametric copula framework. All discussion related to this illustration were in the below paragraph.

**Table 1:** Comparing bivariate versus univariate joint RPs (for different bivariate hazard scenario, i.e., AND and OR scenario) for station (a) Montreal (b) Quebec City (c) Ottawa (d) Toronto (e) Vancouver (f) Regina (g) Edmonton.

(a) MONTREAL													
Univariate RPs (years)	Design quantiles (AMWGS (km/hr))	Design quantiles (Corresponding highest MT (Time lag = 0 day) (°C))	OR-JRP (AMWGS-MT (Time lag = 0 day) (Years)	AND-JRP (AMWGS-MT (Time lag = 0 day) (Years)	Design quantiles (Corresponding highest MT (Time lag = ± 1 days) (°C))	OR-JRP (AMWGS-MT (Time lag = ± 1 days) (Years)	AND-JRP (MT-WGS (Time lag = ± 1 days) (Years)	Design quantiles (Corresponding highest MT (Time lag = ± 2 days) (°C))	OR-JRP (AMWGS-MT (Time lag = ± 2 days) (Years)	AND-JRP (AMWGS-MT (Time lag = ± 2 days) (Years)	Design quantiles (Corresponding highest MT (Time lag = ± 3 days) (°C))	OR-JRP (AMWGS-MT (Time lag = ± 3 days) (Years)	AND-JRP (AMWGS-MT (Time lag = ± 3 days) (Years)
2	80.16	27.24	1.44	3.30	28.79	3.57	3.57	29.04	1.43	3.30	29.07	1.37	3.73
5	94.24	30.25	3.14	12.31	31.20	1.51	15.64	31.28	2.91	17.91	31.30	2.89	18.37
10	102.00	31.41	6.03	29.22	32.19	1.21	41.36	32.26	5.40	68.08	32.28	5.51	53.82
20	108.01	32.31	11.84	64.36	32.79	1.10	98.47	32.84	10.39	265.17	32.85	10.77	140.03
30	112.42	32.72	17.65	99.89	33.05	1.06	157.67	33.09	15.39	591.30	33.08	16.03	233.40
50	122.16	33.14	29.27	171.22	33.30	1.04	277.74	33.33	25.39	1630.52	33.31	26.56	426.95
70	124.66	33.37	40.90	242.65	33.44	1.03	398.55	33.46	35.39	3185.73	33.43	37.08	623.79
80	125.17	33.45	46.71	278.39	33.49	1.02	459.07	33.51	40.39	4158.00	33.48	42.34	722.75
90	125.52	33.52	52.52	314.13	33.54	1.02	519.64	33.55	45.39	5257.62	33.52	47.61	821.90
100	125.78	33.58	58.34	349.87	33.57	1.02	580.21	33.59	50.39	6485.08	33.55	52.87	921.23

Univariate RPs (years)	Design quantiles (Annual highest MT (°C))	Design quantiles (Corresponding WGS (Time lag = 0 day) (km/h))	OR-JRP (AHMT-WGS (Time lag = 0 day) (Years)	AND-JRP (AHMT-WGS (Time lag = 0 day) (Years)									
2	32.53	40.34	1.44	3.30									
5	33.78	57.64	2.91	17.89									
10	34.40	68.75	5.40	67.97									
20	35.33	79.75	10.39	264.73									
30	36.06	82.34	15.39	590.28									
50	37.41	85.59	25.39	1627.87									
70	37.54	86.51	35.39	3180.66									
80	37.56	86.69	40.39	4149.38									
90	37.58	86.81	45.39	5249.34									
100	37.60	86.90	50.39	6476.68									

(b) QUEBEC CITY													
Univariate RPs (years)	Design quantiles (AMWGS (km/hr))	Design quantiles (Corresponding highest MT (Time lag = 0day) (°C))	OR-JRP (AMWGS-MT (Time lag = 0 day) (Years)	AND-JRP (AMWGS-MT (Time lag = 0 day) (Years)	Design quantiles (Corresponding highest MT (Time lag = ± 1 days) (°C))	OR-JRP (AMWGS-MT (Time lag = ± 1 days) (Years)	AND-JRP (AMWGS-MT (Time lag = ± 1 days) (Years)	Design quantiles (Corresponding highest MT (Time lag = ± 2 days) (°C))	OR-JRP (AMWGS-MT (Time lag = ± 2 days) (Years)	AND-JRP (AMWGS-MT (Time lag = ± 2 days) (Years)	Design quantiles (Corresponding highest MT (Time lag = ± 3 days) (°C))	OR-JRP (AMWGS-MT (Time lag = ± 3 days) (Years)	AND-JRP (AMWGS-MT (Time lag = ± 3 days) (Years)
2	80.85	26.79	1.46	3.16	27.41	1.42	3.36	27.68	1.41	3.41	28.45	1.42	3.38
5	103.92	30.12	3.10	12.87	30.21	3.14	12.21	30.67	3.10	12.87	30.41	3.13	12.46
10	123.97	31.59	5.71	40.38	31.41	6.05	28.87	32.21	5.95	31.24	32.23	6.01	29.78
20	139.80	32.66	10.78	137.73	32.27	11.83	64.59	33.26	11.63	71.47	32.99	11.75	67.19
30	147.12	33.18	15.81	291.43	32.68	17.60	101.65	33.73	17.28	113.78	33.22	17.47	106.21
50	174.46	33.75	25.84	767.75	33.12	29.09	177.66	34.22	28.54	201.40	33.45	28.87	186.51
70	177.44	34.09	35.85	1469.29	33.39	40.57	255.02	34.50	39.78	291.26	33.57	40.25	268.46
80	178.36	34.22	40.86	1904.40	33.49	46.30	294.02	34.61	45.39	336.71	33.61	45.93	309.85
90	179.10	34.34	45.86	2395.78	33.57	52.03	333.18	34.70	51.00	382.45	33.65	51.61	351.43
100	179.71	34.43	50.86	2943.77	33.65	57.75	372.45	34.77	56.60	428.41	33.69	57.28	393.14

(c) OTTAWA													
Univariate RPs (years)	Design quantiles (AMWGS (km/hr))	Design quantiles (Corresponding highest MT (Time lag = 0day) (°C))	OR-JRP (AMWGS-MT (Time lag = 0 day) (Years)	AND-JRP (AMWGS-MT (Time lag = 0 day) (Years)	Design quantiles (Corresponding highest MT (Time lag = ± 1 days) (°C))	OR-JRP (AMWGS-MT (Time lag = ± 1 days) (Years)	AND-JRP (AMWGS-MT (Time lag = ± 1 days) (Years)	Design quantiles (Corresponding highest MT (Time lag = ± 2 days) (°C))	OR-JRP (AMWGS-MT (Time lag = ± 2 days) (Years)	AND-JRP (AMWGS-MT (Time lag = ± 2 days) (Years)	Design quantiles (Corresponding highest MT (Time lag = ± 3 days) (°C))	OR-JRP (AMWGS-MT (Time lag = ± 3 days) (Years)	AND-JRP (AMWGS-MT (Time lag = ± 3 days) (Years)
2	77.05	27.38	1.37	3.70	28.92	1.41	3.44	29.27	1.40	3.51	29.61	1.38	3.66
5	86.32	30.44	2.86	19.80	31.36	3.09	13.18	31.88	3.03	14.23	32.27	2.95	16.53
10	93.85	31.83	5.39	69.63	32.80	5.91	32.38	33.12	5.79	36.46	33.59	5.60	46.51
20	102.53	33.17	10.43	243.42	34.14	11.54	74.89	34.17	11.29	87.63	34.58	10.89	122.55
30	109.51	33.86	15.46	505.28	34.67	17.14	119.90	34.70	16.76	143.19	35.04	16.15	210.85
50	131.00	34.56	25.50	1266.30	35.28	28.31	213.59	35.32	27.65	261.16	35.57	26.63	409.10
70	132.54	34.93	35.54	2317.50	35.68	39.45	310.04	35.71	38.50	384.54	35.89	37.07	626.13
80	133.02	35.05	40.55	2945.51	35.83	45.02	358.94	35.85	43.93	447.59	36.01	42.29	739.75
90	133.40	35.16	45.56	3639.01	35.97	50.58	408.20	35.98	49.34	511.35	36.11	47.50	856.09
100	133.71	35.24	50.58	4395.60	36.08	56.13	457.73	36.09	54.76	575.74	36.20	52.70	974.94

(d) TORONTO													
Univariate RPs (years)	Design quantiles (AHMT (°C))	Design quantiles (Corresponding maximum WGS (Time lag = 0day) (km/h))	OR-JRP (AHMT-WGS (Time lag = 0 day) (Years)	AND-JRP (AHMT-WGS (Time lag = 0 day) (Years)	Design quantiles (Corresponding maximum WGS (Time lag = ± 1 days) (km/h))	OR-JRP (AHMT-WGS (Time lag = ± 1 days) (Years)	AND-JRP (AHMT-WGS (Time lag = ± 1 days) (Years)	Design quantiles (Corresponding maximum WGS (Time lag = ± 2 days) (km/h))	OR-JRP (AHMT-WGS (Time lag = ± 2 days) (Years)	AND-JRP (AHMT-WGS (Time lag = ± 2 days) (Years)	Design quantiles (Corresponding maximum WGS (Time lag = ± 3 days) (km/h))	OR-JRP (AHMT-WGS (Time lag = ± 3 days) (Years)	AND-JRP (AHMT-WGS (Time lag = ± 3 days) (Years)
2	32.80	45.87	1.36	3.78	53.79	1.39	3.54	52.43	1.43	3.31	54.54	1.44	3.28
5	34.64	58.33	2.87	19.27	59.63	3.07	13.55	60.61	3.24	10.95	61.09	3.18	11.65
10	35.70	60.59	5.47	58.55	63.48	5.95	31.40	64.79	6.35	23.55	64.87	6.19	26.02
20	36.38	66.58	10.68	157.93	73.89	11.75	67.14	70.68	12.61	48.37	71.29	12.25	54.45
30	36.64	67.20	15.89	267.99	74.15	17.57	102.71	74.64	18.88	73.06	75.33	18.32	82.70
50	36.90	79.92	26.32	499.00	79.92	29.21	173.63	77.66	31.42	122.30	77.98	30.48	139.01
70	37.03	80.00	36.75	735.51	80.01	40.85	244.37	78.87	43.98	171.48	79.06	42.65	195.20
80	37.07	80.03	41.96	854.70	80.03	46.67	279.75	79.26	50.25	196.06	79.40	48.73	223.28
90	37.11	80.05	47.18	974.28	80.05	52.50	315.09	79.57	56.53	220.63	79.67	54.81	251.36
100	37.14	80.07	52.39	1094.09	80.07	58.32	350.41	79.82	62.81	245.21	79.90	60.90	279.42

(e) VANCOUVER													
Univariate RPs (years)	Design quantiles (AHMT (°C))	Design quantiles (Corresponding maximum WGS (Time lag = 0 day) (km/h))	OR-JRP (AHMT-WGS (Time lag = 0 day) (Years)	AND-JRP (AHMT-WGS (Time lag = 0 day) (Years)	Design quantiles (Corresponding maximum WGS (Time lag = ± 1 days) (km/h))	OR-JRP (AHMT-WGS (Time lag = ± 1 days) (Years)	AND-JRP (AHMT-WGS (Time lag = ± 1 days) (Years)	Design quantiles (Corresponding maximum WGS (Time lag = ± 2 days) (km/h))	OR-JRP (AHMT-WGS (Time lag = ± 2 days) (Years)	AND-JRP (AHMT-WGS (Time lag = ± 2 days) (Years)	Design quantiles (Corresponding maximum WGS (Time lag = ± 3 days) (km/h))	OR-JRP (AHMT-WGS (Time lag = ± 3 days) (Years)	AND-JRP (AHMT-WGS (Time lag = ± 3 days) (Years)
2	28.81	0.02	1.41	3.44	32.21	1.04	2.01	0.12	1.44	3.29	39.27	1.39	3.59
5	29.99	0.14	2.96	16.20	40.78	1.16	5.09	40.79	2.97	15.94	46.49	2.84	20.79
10	31.43	34.40	5.54	51.31	47.62	1.35	10.49	46.40	5.53	52.51	49.78	5.33	81.02
20	31.90	36.01	10.66	161.05	51.17	1.59	21.73	51.64	10.61	173.02	52.29	10.32	319.83
30	33.30	36.90	15.75	313.52	52.72	1.86	34.12	54.94	15.68	347.54	53.61	15.32	716.44
50	34.38	37.82	25.89	724.01	54.56	2.32	61.21	67.97	25.77	836.89	55.44	25.32	1982.95
70	34.39	38.80	36.01	1255.18	67.87	3.06	96.62	68.02	35.84	1493.21	67.88	35.32	3881.99
80	34.39	39.20	41.05	1560.55	67.92	4.11	129.60	68.03	40.87	1878.29	67.93	40.31	5065.86
90	34.40	39.44	46.10	1891.79	67.95	5.91	183.95	68.04	45.90	2300.44	67.96	45.32	6410.26
100	34.40	39.59	51.13	2246.18	67.98	8.73	271.06	68.05	50.92	2757.10	67.99	50.31	7911.39

(f) REGINA													
Univariate RPs (years)	Design quantiles (AMWGS (km/hr)	Design quantiles (Corresponding highest MT (Time lag = 0day) (°C))	OR-JRP (AMWGS-MT (Time lag = 0 day) (Years)	AND-JRP (AMWGS-MT (Time lag = 0 day) (Years)	Design quantiles (Corresponding highest MT (Time lag = ± 1 days) (°C))	OR-JRP (AMWGS-MT (Time lag = ± 1 days) (Years)	AND-JRP (AMWGS-MT (Time lag = ± 1 days) (Years)	Design quantiles (Corresponding highest MT (Time lag = ± 2 days) (°C))	OR-JRP (AMWGS-MT (Time lag = ± 2 days) (Years)	AND-JRP (AMWGS-MT (Time lag = ± 2 days) (Years)	Design quantiles (Corresponding highest MT (Time lag = ± 3 days) (°C))	OR-JRP (AMWGS-MT (Time lag = ± 3 days) (Years)	AND-JRP (AMWGS-MT (Time lag = ± 3 days) (Years)
2	102.87	26.58	1.46	3.16	28.27	1.42	3.38	29.10	1.41	3.45	29.25	1.39	3.53
5	120.25	29.64	3.23	11.09	31.57	3.05	13.77	32.53	2.93	17.15	32.90	2.88	18.76
10	133.11	31.19	6.04	29.14	32.92	5.70	40.77	33.70	5.45	61.04	34.12	5.41	66.30
20	140.10	32.28	11.36	83.29	33.84	10.83	130.08	34.57	10.46	227.35	35.16	10.45	234.36
30	143.24	32.77	16.54	161.20	34.29	15.90	266.09	34.96	15.47	498.23	35.60	15.47	490.51
50	147.05	33.27	26.72	387.82	34.78	25.96	677.78	35.34	25.47	1353.73	36.01	25.51	1243.78
70	149.35	33.55	36.82	708.57	35.08	35.99	1275.51	35.54	35.47	2627.43	36.22	35.54	2295.68
80	150.18	33.65	41.85	904.16	35.20	41.00	1644.20	35.61	40.47	3421.14	36.29	40.55	2928.26
90	150.86	33.74	46.88	1123.34	35.29	46.01	2059.31	35.67	45.47	4319.65	36.36	45.57	3628.45
100	151.44	33.82	51.90	1365.93	35.38	51.01	2520.80	35.72	50.47	5321.98	36.41	50.58	4395.60

Univariate RPs (years)	Design quantiles (AHMT (°C))	Design quantiles (Corresponding maximum WGS (Time lag = 0day) (km/h))	OR-JRP (AHMT-WGS (Time lag = 0 day) (Years)	AND-JRP (AHMT-WGS (Time lag = 0 day) (Years)	Design quantiles (Corresponding maximum WGS (Time lag = ± 1 days) (km/h))	OR-JRP (AHMT-WGS (Time lag = ± 1 days) (Years)	AND-JRP (AHMT-WGS (Time lag = ± 1 days) (Years)	Design quantiles (Corresponding maximum WGS (Time lag = ± 2 days) (km/h))	OR-JRP (AHMT-WGS (Time lag = ± 2 days) (Years)	AND-JRP (AHMT-WGS (Time lag = ± 2 days) (Years)	Design quantiles (Corresponding maximum WGS (Time lag = ± 3 days) (km/h))	OR-JRP (AHMT-WGS (Time lag = ± 3 days) (Years)	AND-JRP (AHMT-WGS (Time lag = ± 3 days) (Years)
2	36.29	50.46	1.43	3.33	60.54	1.42	3.36	65.51	1.41	3.47	70.07	1.37	3.68
5	37.28	60.13	3.00	14.89	77.06	2.91	17.60	78.81	2.94	16.56	82.43	2.85	20.61
10	37.68	75.02	5.62	45.23	87.05	5.41	65.70	87.99	5.52	53.05	89.36	5.35	75.86
20	38.13	82.21	10.79	135.99	93.87	10.41	253.33	93.40	10.63	168.42	93.78	10.37	279.21
30	38.44	90.37	15.93	258.09	96.81	15.41	562.87	95.79	15.71	330.11	97.31	15.39	598.41
50	40.59	92.25	26.13	577.07	100.32	25.41	1547.75	99.90	25.84	768.99	110.84	25.41	1563.48
70	40.60	93.05	36.30	978.86	102.47	35.41	3020.24	102.94	35.94	1340.48	113.65	35.42	2942.91
80	40.60	93.31	41.37	1207.15	103.25	40.41	3938.56	103.87	40.98	1671.12	114.18	40.43	3782.15
90	40.61	93.51	46.44	1452.22	103.91	45.41	4980.08	104.53	46.02	2029.63	114.54	45.43	4721.44
100	40.61	93.67	51.50	1712.62	104.46	50.41	6142.51	105.02	51.05	2414.29	114.80	50.43	5753.74

(g) EDMONTON													
Univariate RPs (years)	Design quantiles (AMWGS (km/h))	Design quantiles (Corresponding highest MT (Time lag = 0 day) ((°C)))	OR-JRP (AMWGS-MT (Time lag = 0 day) (Years)	OR-JRP (AMWGS-MT (Time lag = 0 day) (Years)	Design quantiles (Corresponding MT (Time lag = ± 1 days))	OR-JRP (AMWGS-MT (Time lag = ± 1 days) (Years)	AND-JRP (AMWGS-MT (Time lag = ± 1 days) (Years)						
2	88.90	25.17	1.39	3.54	26.84	1.40	3.49						
5	104.22	28.46	2.89	18.40	29.60	2.90	18.21						
10	109.54	29.80	5.40	67.13	30.71	5.43	63.52						
20	113.32	30.88	10.41	254.16	31.45	10.47	221.65						
30	115.04	31.43	15.41	560.54	31.79	15.51	460.41						
50	116.78	32.03	25.41	1531.39	32.17	25.55	1156.47						
70	117.72	32.38	35.42	2979.74	32.41	35.59	2121.34						
80	118.05	32.51	40.42	3881.99	32.50	40.60	2698.33						
90	118.33	32.63	45.42	4904.36	32.57	45.62	3336.67						
100	118.56	32.72	50.42	6045.95	32.64	50.63	4035.51						

#### Selected cities within the Central Canada region

Firstly, the quantiles of a design variable were estimated at various annual non-exceedance probabilities (NEPs) (or univariate RPs T) at a probability level of (1 − μ/T, where μ is the number of extreme events happening per years) using the best-fitted marginal CDF inverse (or quantile function) (refer to Supplementary SA6 (Eqs. 5–7)). NEPs considered were 2–100 years. It has been discovered that AMWGS and corresponding MT quantiles were more intense at lower univariate RPs (e.g., 2, 5, or 10 years) or high occurrence probabilities at Ottawa, Quebec City, and Montreal. For instance, at Quebec City, AMWGS reached an extremely dangerous level of 103.92 km/h (as mentioned in NOAA^91^) at a lower RP of 5 years as compared to Ottawa (i.e., 102.53 km/h at 20 years) and Montreal (102 km/h at 10 years). This further inferred that Quebec City have higher risk of PB than Montreal and Ottawa. On the other hand, the corresponding MT events were stronger and more impactful at Ottawa than at other stations at the same univariate RPs. For example, at 5 years of univariate RP, the MT quantiles for Ottawa at different time lags ranged from 30.44 (0 day) to 32.27 (± 3 days), while for Montreal, it ranged from 30.25 (0 day) to 31.30 (± 3 days), and for Quebec City, it ranged from 30.12 (0 day) to 30.41 (± 3 days). Furthermore, the estimated MT quantiles for all these stations increased consistently with time lags. MT events of lag ± 3 days may pose a greater risk to communities through HACD for all three station.

This investigation studied the joint risk occurrence of bivariate events for selected EVSG-1 datasets, AMWGS-MT, with a lag of 0 to ± 3 days. The joint EPs were used to calculate RP for both AND and OR hazard scenarios. It was found that the occurrence of bivariate events simultaneously was less frequent in the AND-joint case than in the OR-joint case. This means that T^OR^ (OR-joint RP) is less than T^Univariate^ (Univariate RP) is less than T^AND^ (Bivariate AND-joint RP) for stations in Montreal, Quebec City, and Ottawa. At lower univariate RPs, such as 2, 5, or 10 years, the selected stations had considerably lower AND-joint RPs defined by combining design quantiles of AMWGS and MT events, which exhibited higher magnitude at all specified time lags. For instance, at Quebec City, with univariate RP of 2, 5 & 10 years, the estimated AND-joint RPs were 3.16, 12.87 & 40.28 years (at lag 0 day), 3.36, 12.21 & 28.87 (at ± 1 days), 3.41, 12.87 & 31.24 (at ± 2 days), and 3.38, 12.46, 29.78 (at ± 3 days) respectively. The estimated quantile strength associated with these AND-joint RPs was sufficiently higher. AMWGS was at a moderate risk level to may cause PBs, and MT events were also significant for HACDs. Lower AND-joint RPs were found for bivariate events at a time lag of ± 1 day (when univariate RP was 5 years). At Quebec City, bivariate events AMWGS-MT (Time lag =  ± 1 days) followed by AMWGS-MT (Time lag =  ± 3 days) posed a greater risk than those observed at other time lags (for example, 0 & ± 2 days).

In Montreal, it was observed that the lower AND-joint RP (or with higher concurrence probability) occurred at higher quantiles of AMWGS and MT events that were defined at lower univariate RP (i.e., 2, 5, or 10 years). The lowest AND-joint RPs were observed at a time lag of 0 day. The bivariate events consisted of high AMWGS with 94.24 or 102 km/h quantiles and MT with 30.25 ℃ or 31.41 ℃. This indicates that their joint action or exceedance of AMWGS and MT events would be stressful within the community. This could increase the risk of PB and HACDs simultaneously because of higher design quantiles with higher AND probabilities. Additionally, bivariate events AMWGS-MT (Time lag = 0 day) would be riskier for the community than AMWGS-MT (Time lag =  ± 1 or ± 2, or ± 3 day). However, the risk is comparatively lower than that of Quebec City.

While, for Ottawa, it was observed that the moderate risk threat level of AMWGS and higher MT events quantiles occurred at lower univariate RPs. These events would be stressful if considered separately for PB or HACD. However, when these events were combined, it resulted high joint concurrence probabilities (or lower AND-joint RP) at different time lags. For example, the AND-joint RP was 13.18 years (at ± 1 days), 16.53 years (at ± 3 days), 19.8 years (at 0 day), and 14.23 years (at ± 2 years). The estimated joint RPs varied inconsistently between time lags. Based on these statistics, it can be inferred that the joint action of bivariate events AMWGS-MT (Time lag =  ± 1 days) would pose a higher risk and stress and may result in the simultaneous PBs and HACDs events in Ottawa. Also, the stress level is comparably lower than Quebec City. In conclusion, it was found that the compounded AMWGS-MT events would be more impactful or severe at ± 1 days for both Quebec City and Ottawa, while for the Montreal, it occurs at 0 days.

The bivariate events in Toronto and Montreal were identified based on the EVSG-2 datasets, compounded AHMT and its corresponding WGS events. At Toronto, AHMT would have high levels of stressful situations (i.e., 32.80 ℃ or 34.64 ℃, etc.) at lower univariate RP levels, while the WGS events posed a moderate risk, with speeds of 60.61 km/h (at 5-year RP) and 64.79 km/h (at 10-year RP). When was investigated the joint action of AHMT and WGS, the bivariate event at lag of ± 2 days posed more stress (with a moderate-risk level) than other bivariate events defined at different time lags. For example, when the AHMT of 34.64 ℃ or 35.70 ℃ was combined with WGS (Time lag =  ± 2 days) of 60.61 or 64.69 km/h, the estimated RP for the AND-hazard scenario was 10.95 or 23.55 years. Therefore, events AHMT-WGS (Time lag =  ± 2 days), followed by AHMT-WGS (Time lag =  ± 3 days) events, would be risky within the community in Toronto. Conversely, for Montreal, AHMT had higher estimated quantiles for lower univariate RPs, while corresponding WGS quantiles had a moderate level of risk at a time lag of 0 days. The joint risk of AHMT-WGS resulted in a lower AND-joint RP with moderate risk levels. Comparing the risk level between Toronto and Montreal at a time lag of 0 days, Toronto had higher quantiles than Montreal, resulting in higher joint probability risk for events AHMT-WGS at 0-day time lag.

#### Selected cities within Pacific region

It is unlikely that the occurrence of AHMT and WGS events together in Vancouver City will have a significant impact on the community. At lower univariate RPs, the quantiles of AMHT were high, while those of WGS were low. However, when moderate-level WGS occurred at a time lag of ± 2 or ± 3 days and combined with AHMT quantiles, the joint RPs were low. The associated WGS quantiles at this joint RPs did not show significant threat levels compared to other cities like Toronto. This confirms that Vancouver City’s combined hazard risk of AHMT and WGS is very low. This could further confirm that there is a lower risk of simultaneous PB and HACDs within community.

#### Selected cities within Canadian Prairies region

At Regina, the AMWGS and MT quantiles were higher than all other stations, even at lower univariate RPs, and the associated MT quantiles consistently increased with a time lag. The joint action for AMWGS and MT hazard resulted in significantly higher concurrence or AND-joint probabilities (i.e., 3.53 years (AMWGS of 102.87 km/h & MT (Time lag =  ± 3 days) of 29.25 $^\circ{\rm C}$). The estimated AND-joint RPs were consistently increased with a time lag for any possible combination of AMWGS and MT quantiles, with the MT quantiles being more significant at higher time lags. The bivariate pair observed at lag of ± 1 day & 0 day may pose a significant threat.

Conversely, for same station, it was observed that the AHMT quantiles were very high when was considering EVSG-2 datasets (AHMT and WGS), with lower univariate RPs of 5 or 10 years. While the corresponding WGS quantiles posed a moderate threat at the same RPs. However, their magnitude was higher when observed at a higher time lag. This confirmed that when both events happened independently, it may result for PB or HACDs putting impactful stress within the community. On the other side, the AND-joint RP were ranged between 14.89 (at 0 days) to 20.61 years (at ± 3 days) when modelled the joint action of AHMT and WGS hazard quantiles estimated at univariate RP of 5-years. When exceeded the univariate RP of 10 years or above, it resulted in higher AND-joint RPs ranging between 45.23 years (at 0 day) to 75.86 years (at ± 3 days). Therefore, at a time lag of ± 3 day, the joint probability stress of AHMT and WGS is more significant, and both AHMT and WGS events attained significant value. This investigation revealed that mutual concurrency of AHMT and WGS at lag ± 2 and ± 3 days would be riskier and may significantly impact Regina community.

Bivariate events based on EVSG-1 were selected for joint impact analysis at Edmonton with a time lag of 0 and ± 1 days. AMWGS and MT quantiles were higher at lower univariate RPs. Lower joint RPs were observed for AMWGS-MT, but their associated quantiles were not much more impactful than Regina. Both AMWGS and MT quantiles were high at lower RP must, indicating a higher risk impact of occurring independently. Bivariate events AMWGS-MT would be more stressful when observed at a lag of ± 1 days than 0 days.

#### Bivariate hazard risk estimates via conditional distribution

**Figure 2:**
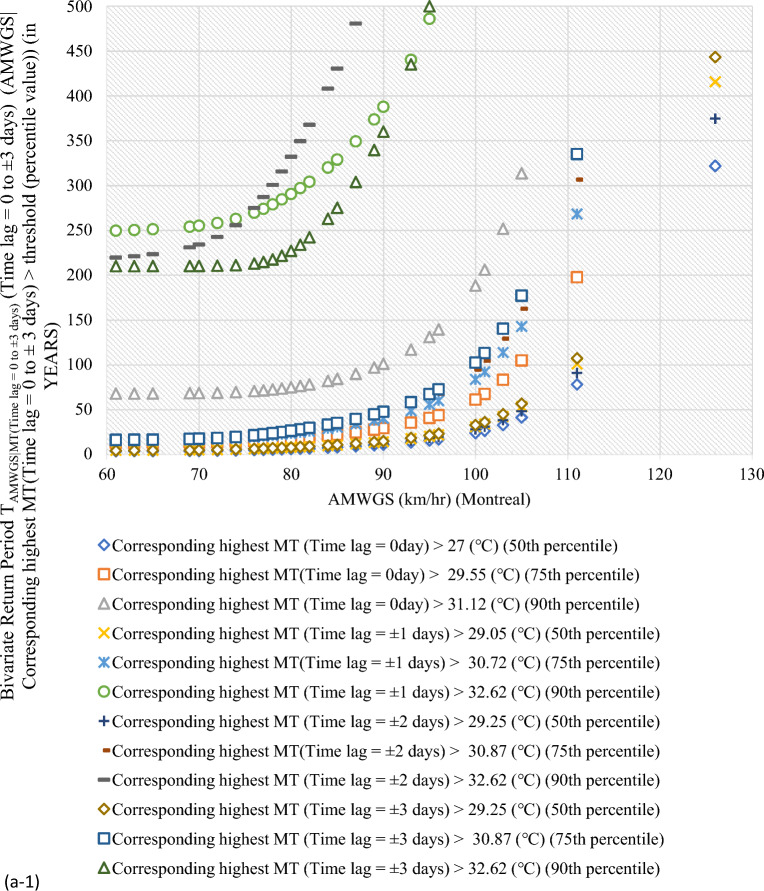

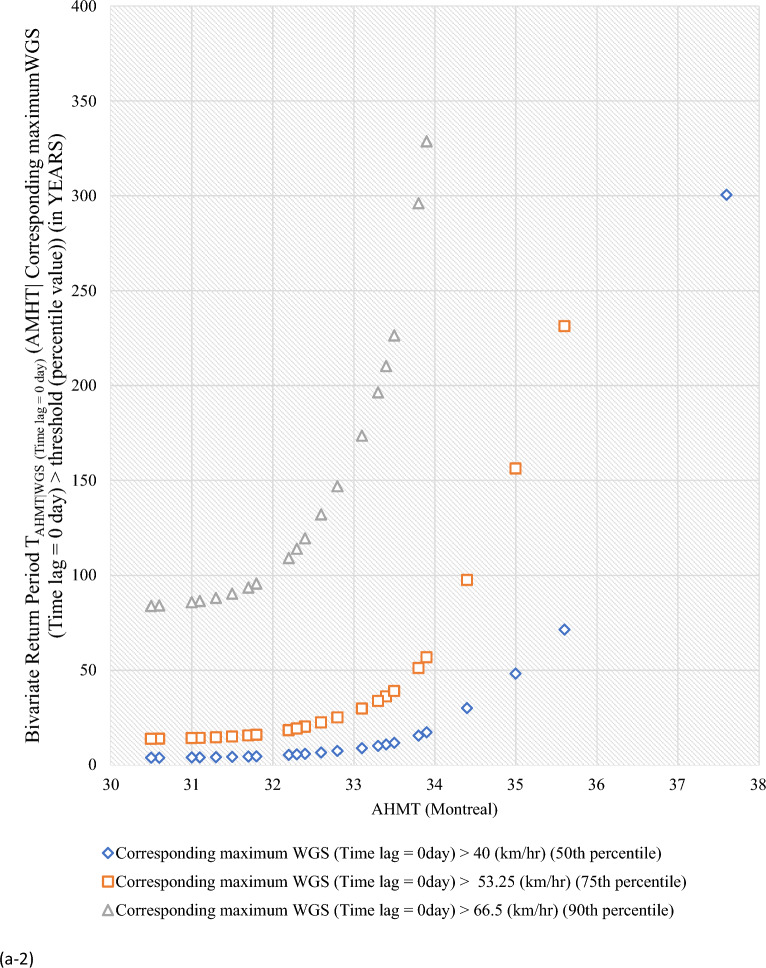

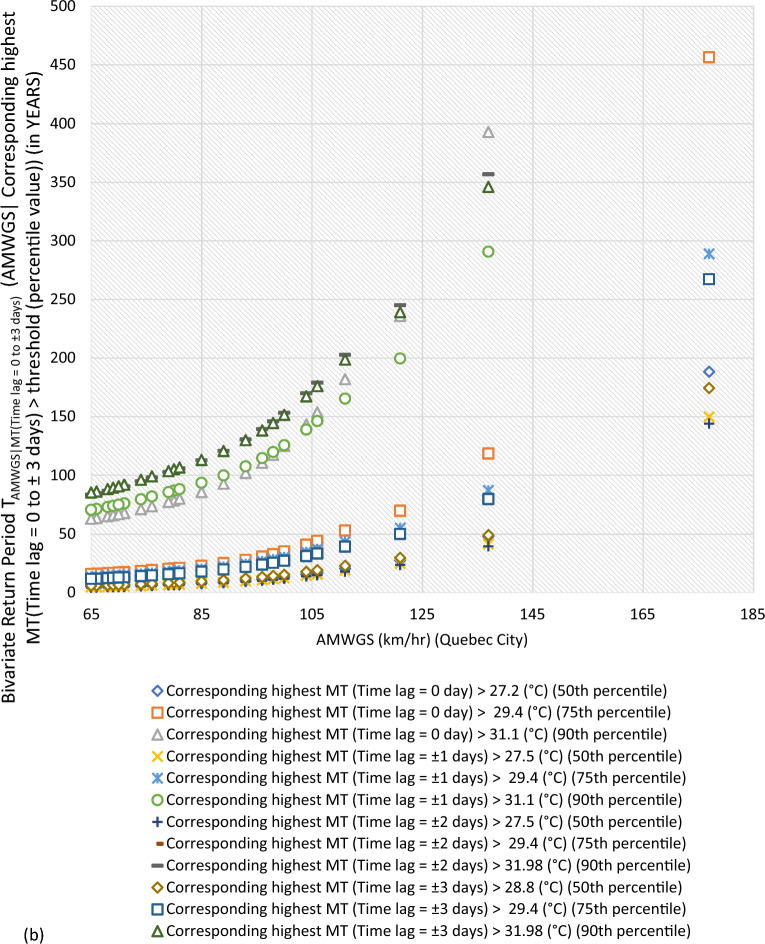

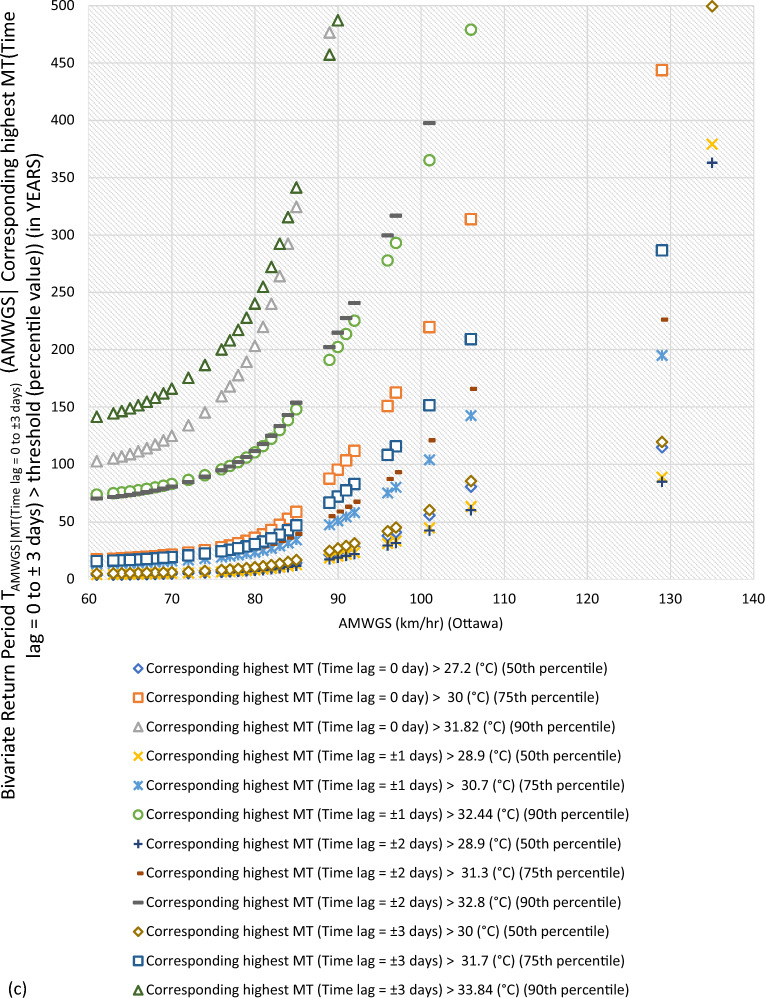

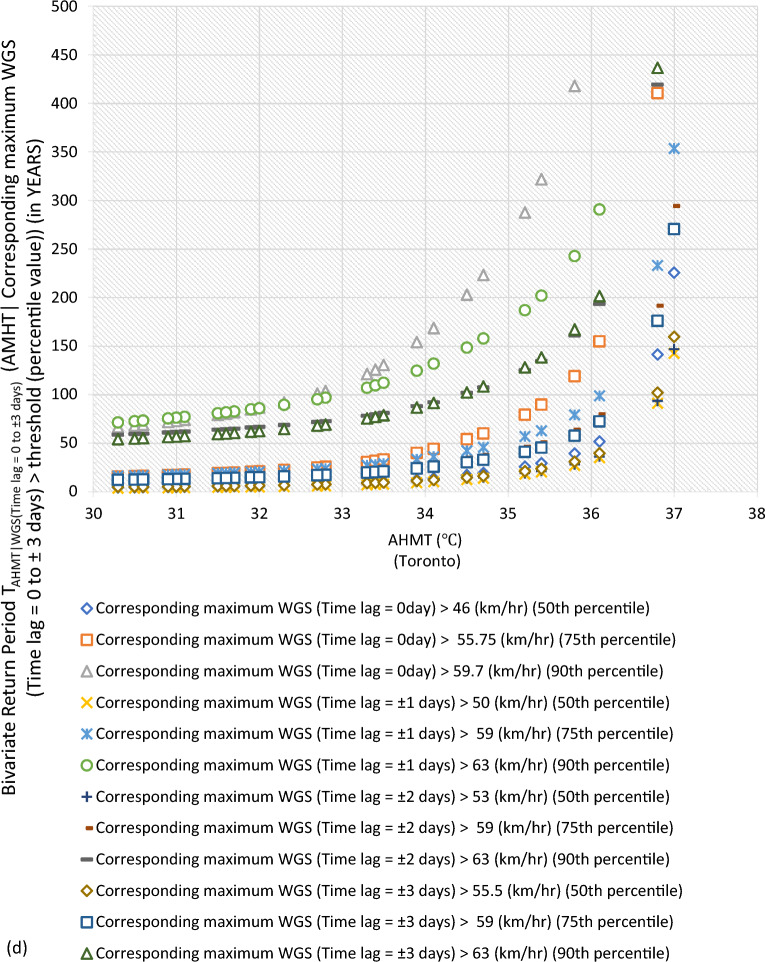

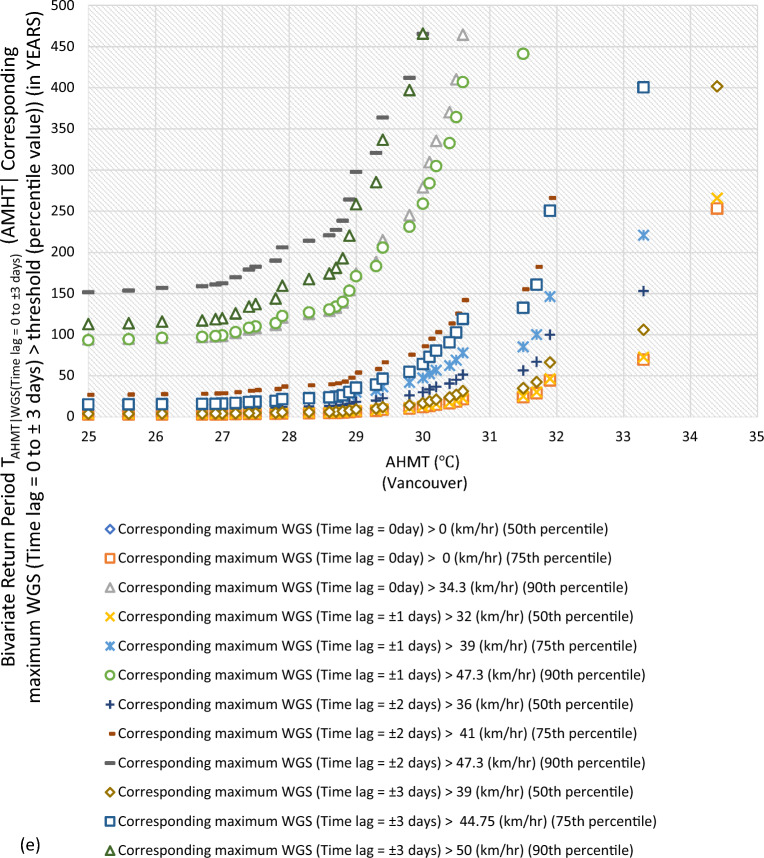

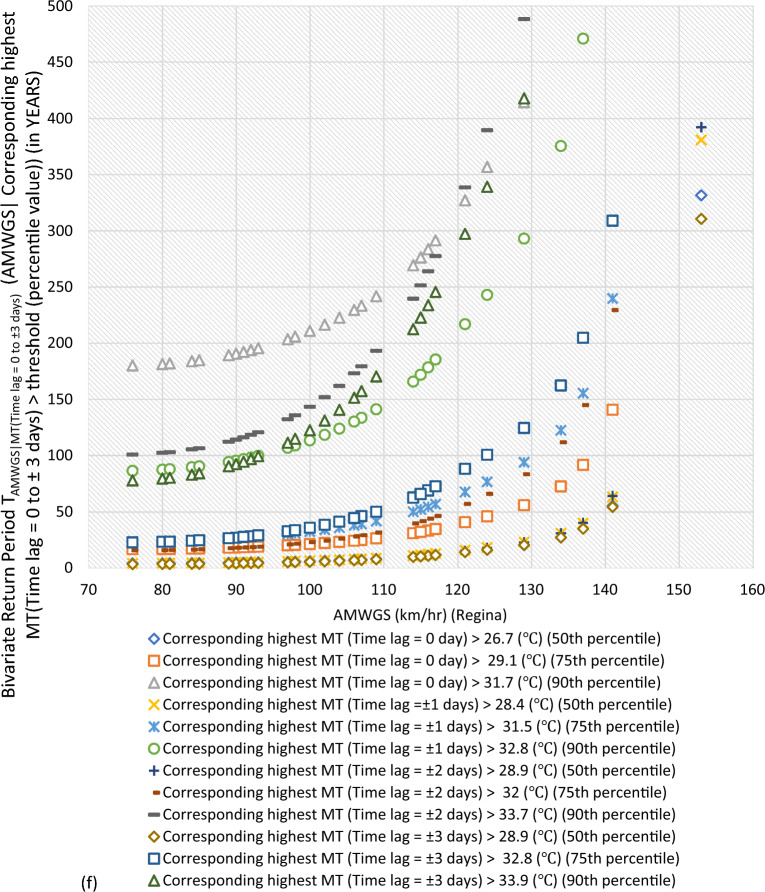

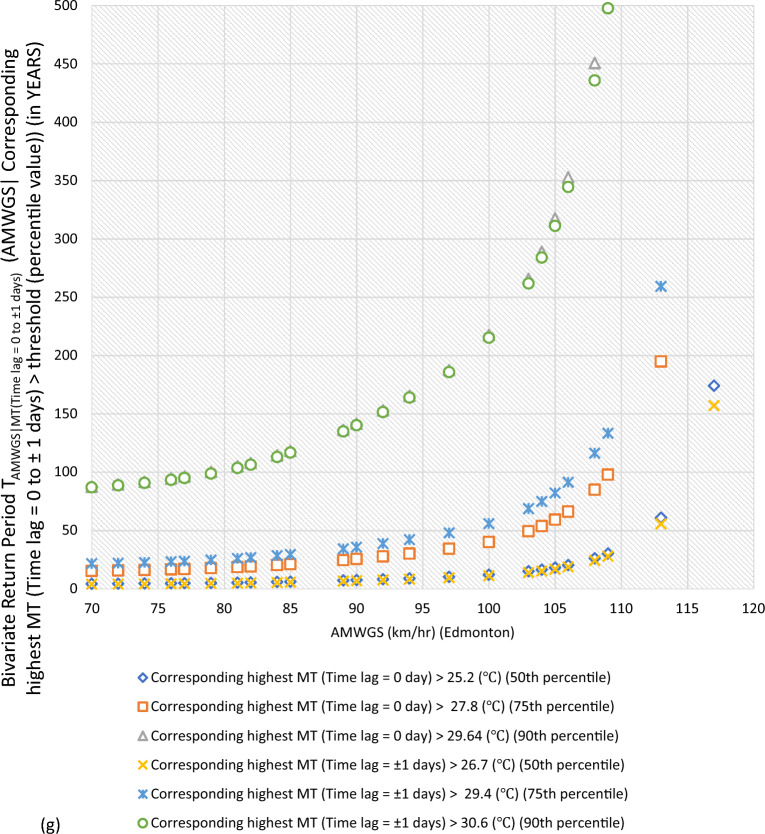
Bivariate hazard risk modelling based on conditional joint return period of events AMWGS (or AHMT) given various percentiles (50th, 75th & 90th) values of corresponding MT (or WGS) observed at different time lag for station (**a-1** & **a-2**) Montreal (**b**) Quebec City (**c**) Ottawa (**d**) Toronto (**e**) Vancouver (**f**) Regina (**g**) Edmonton.

In Supplementary SA6, Eqs. (3) and (4) estimated joint occurrence risk of AMWGS (or AHMT) with percentile values of MT (or WGS) events at different time lags (see Fig. 2a–g). This study focused on two percentile levels: 50th & 75th percentiles when conditional on MT (EVSG-1 dataset), and 75th & 90th percentiles when conditional on WGS (EVSG-2 dataset).

#### Joint risk of AMWGS conditional on MT events for EVSG-1 datasets

For Montreal station, when the 50th and 75th percentile of MT (Time lag = 0 day) events combined with a higher threatening level of AMWGS, such as those that occurred on July 4, 1955, and June 22, 1972 (at speeds of 95 km/h and 100 km/h), the associated joint RP was 15.62 and 40.606 years (or 23.81 and 61.09 years). When compounded MT (Time lag =  ± 1 days) with same AMWGS level, the corresponding RP was 20.28 and 55.72 years (or 30.87 and 83.64 years). The lower conditional joint RPs indicated that these bivariate events would be stressful and may pose a severe threat when their joint actions are compounded, as both percentiles of MT have already exhibited a higher value. It is important to note that the selected AMWGS events already pose a high level of risk (at speeds of 95 km/h or 100 km/h). The 50th percentile of MT (Time lag =  ± 2 days) reached a higher temperature level (≥ 29.25 ℃) than any other time lag, which would increase the demand for air conditioning. Even when combined MT (Time lag =  ± 2 days) with the aforementioned AMWGS events, it still resulted a lower joint RP (i.e., 18.35 years or 27.87 years). In conclusion, at Montreal station, the joint effect of AMWGS-MT at lag 0 day would pose a high risk compared to other time lags.

For Quebec City, at the same percentile level of MT events, the joint RP was 16.98 years with 106 km/h AMWGS and 44.06 years with 111 km/h AMWGS. Alternatively, it was 20.63 years with 106 km/h AMWGS and 52.84 years with 111 km/h AMWGS. The MT events’ intensity was already moderate at any given time lag, whether at the 50th percentile, ranging from 27.2 ℃ (time lag = 0 day) to 28.8 ℃ (time lag =  ± 3 days), or at the 75th percentile, which is 29.4 ℃ (time lag = 0 to ± 3 days). This could lead to moderate air conditioning needs. Meanwhile, AMWGS events were already posed a higher risk threat level. Combining them with MT (time lag of ± 3 days) resulted in lower joint RPs of 19.13 years (with AMWGS of 106 km/h) and 22.84 years (with AMWGS of 111 km/h). Even when combined with MT (time lag of 0 day), joint RPs were lower. Joint RPs were lowest when AMWGS was compounded with MT (time lag of ± 2 days). This further infers that Quebec City resulted in higher conditional joint exceedance compared to Montreal, which means the joint stress of higher AMWGS and MT events may lead to simultaneous PB and HACD.

At Ottawa, when higher AMWGS levels and MT levels were combined, the joint RPs were relatively higher compared to Montreal or Quebec City. For example, joint RPs were 80.59 and 313.79 years for AMWGS levels of 106 km/h and 90 km/h with MT levels of 50th percentile (≥ 28.9 ℃, at lag ± 2 days) and 75th percentile (≥ 30.7 ℃, at lag ± 1 day). However, moderate AMWGS levels (≤ 90 km/h) with 50th percentile of MT events (≥ 30 ℃, at a time lag of ± 3 days) resulted in lower joint RPs of 26.99 years, which is still potentially severe but lower than at Quebec City or Montreal.

Similarly, for Edmonton, it has been discovered that when moderate levels of AMWGS (i.e., 89 km/h or 77 km/h) combine with MT events at the 50th percentile (≥ 25.2 ℃ or ≥ 26.7 ℃), they do not cause significant stress. However, higher levels of AMWGS events such as ≥ 100 km/h combined with MT events at the 75th percentile (≥ 27.8 ℃ or ≥ 29.4 ℃) have a lower probability of occurring or higher return (i.e., RP > 40.08 years). When the 75th percentile of MT events combined with moderate AMWGS events like 90 or 97 km/h, the bivariate joint RPs were quite less, 25.55 and 35.70 years, respectively. In summary, it can be said that bivariate events of AMWGS-MT at Edmonton are riskier and may have a potential to exert stress within community when it observed at a time lag of 0 days than at ± 1 days. However, compared to other stations like Ottawa, Quebec City, or Montreal, Edmonton has a lower risk from the joint action of AMWGS and MT events.

At Regina, the 50th percentile of MT events at lag 0 day was not high enough in magnitude compared to other time lags (i.e., ≥ 28.4 (℃) at ± 1 days, ≥ 28.9 (℃) at lag ± 2 & ± 3 days). However, at the 75th percentile, it consistently increases with a time lag, which was high enough. The conditional RPs between high magnitude AMWGS levels (i.e., > 116 km/h) and the 50th or 75th percentile of MT events are very low, indicating higher conditional probability (or more risky events). For example, the conditional joint RPs at the 75th percentile of MT events and very high threatening levels of AMWGS events (say, 100 km/h or 117 km/h) were 21.37 or 34.78 years (at lag 0 day), 32.49 or 56.86 years (at lag ± 1 days), 22.95 or 46.34 years (at lag ± 2 days), and 35.95 or 72.52 years (at lag ± 3 days). Due to the lower MT events observed at the 50th percentile, the joint stress with AMWGS would be insignificant compared to when compounded with the 75th percentile of MT events. Also, when combined with the same AMWGS level with the 75th percentile of MT events, the lowest joint RPs were obtained at a time lag of 0 days compared to any other time lags.

#### Joint risk of AHMT conditional on WGS events for EVSG-2 dataset

This study analyzed EVSG-2 datasets for several Canadian cities, such as Toronto, Vancouver City, Regina, and Montreal. Toronto has high AHMT events that can cause HACDs. WGS events show moderate threat levels at the 75th percentile, ranging from 55.75 to 59 km/h. At the 90th percentile, moderate threat levels are significantly higher, ranging from 59.7 to 63 km/h. Joint occurrence with higher AHMT events resulted in lower joint RPs of 14.37–19.59 years (at a time lag of 0 to ± 3 days). At the 90th percentile of WGS events, the estimated conditional RPs range from 60.43 to 68.99 years (at ± 3 days) to 81.77 to 103.66 years (at 0 days).

In Vancouver City, both the 75th and 90th percentiles of WGS events showed very low threat levels, ranging from 44.75 km/h (at ± 3 days) to 0 km/h (at 0 day) than Toronto City. Although individually, they may pose a low risk for PB events, when higher AHMT events (such as 27.8 ℃, 29.4 ℃, or 31.7 ℃) were combined with the 75th percentile of WGS (time lag =  ± 3 days), the conditional RPs increase significantly, say, 19.55 years, 46.22 years, or 160.57 years, respectively. Similarly, when considering the 90th percentile of WGS (Time lag =  ± 3 days) with the same AMHT magnitude levels, the joint RP was higher than the former indicated for low stress. In conclusion, Vancouver City’s estimated conditional joint RPs were still much higher than Toronto City’s compared to the same magnitude of AHMT and WGS events. Therefore, the joint probability risk of these bivariate events (AHMT-WGS) would be much less impactful within the Vancouver City community than in Toronto.

Regina had moderate WGS risk levels, occurring at the 75th percentile (80 km/h, lag ± 3 days to 74 km/h, lag ± 1 day) and 50th percentile (70 km/h, lag ± 3 days to 59 km/h, lag ± 1 day). Still, the risk level was slightly lower than the 75th percentile. Combined with higher AHMT events, it resulted in low joint RPs (4.70 and 19.36 years). Regina would be more stressed than Toronto and Vancouver City due to the combined impact of these events.

In Montreal, the 75th and 90th percentiles of WGS events were moderately threatening at speeds of at least 53.25 km/h and 66.5 km/h, respectively. When AHMT levels were increased to 30.6 or 32.2 °C, the estimated conditional joint RPs were 13.96 and 84.07 years or 18.44 and 109.139 years. The joint stress of EVSG-2 (AHMT-WGS) is less severe in Montreal than EVSG-1 (AMWGS-MT) events. AHMT events, when combined with the 90th percentile of WGS, resulted in high conditional joint RPs (at lag 0 day) (less probable).

## Conclusion and future recommendations

This study incorporated a flexible semiparametric copula-based bivariate risk hazard framework to model the joint probability action of compounded WGS and MT events in Canadian cities, given the increasing prevalence of elevated summer temperatures and intense wind gust events. Air conditioning demand is on the rise in several Canadian provinces due to the hot summer weather. However, frequent extreme wind gusts have been causing power outages, which is having a negative impact on the communities that rely on electrical power-based equipment, such as air conditioning. If these events occur simultaneously or successively, their combined effect can be more severe, especially if they exhibit higher magnitudes (higher WGS and MT quantiles) with high concurrence probabilities or joint RP for AND hazard scenarios. A joint density model was created to investigate the joint action by using parametric copulas and nonparametric GKDE margins with nine bandwidth selectors. This semiparametric model can help minimize the risk of misspecification in case the underlying distributional assumption of the marginal pdf is violated when compared to traditional parametric copula settings. The bivariate distribution models were used to perform risk analysis. This involved estimating exceedance probabilities of joint and conditional distributions, return for primary OR and AND-joint hazard scenarios, and conditional joint RP. Bivariate density frameworks were created for bivariate extreme pairs AMSGW-MT (time lag = 0 to ± 3 days) in the case of EVSG-1, and AHMT-WGS (time lag = 0 to ± 3 days) in the case of EVSG-2 at each selected station. Based on significant findings, this study has drawn the following conclusions:

- Positive correlations were observed between AMWGS and MT pair events of EVSG-1 datasets at Montreal, Quebec City, Ottawa, and Regina. However, at Edmonton, positive correlation was noted for the same pairs but only at a time lag of 0 and ± 1 days. Similarly, a positive correlation was observed between bivariate events pair AHMT-WGS of EVSG-2 datasets at Regina, Toronto, and Vancouver City. The study found no significant correlation at Calgary and Halifax stations. The estimated dependency measures revealed that the correlation strength varied inconsistently within selected lags at the above-mentioned stations. The varying correlation measures between cities may be influenced by temperature, pressure, and local geography. For example, the strong positive correlation between WGS and higher summer temperatures in Quebec City may result from atmospheric processes and local features. Toronto usually experiences lower gusts than Quebec City due to its location on the northern shore of Lake Ontario, which generates less strong gusts than the river valley in Quebec City. Also, warmer summer temperatures in Quebec City usually create favorable conditions for convection and localized wind patterns. Similarly, Vancouver’s coastal climate, ocean currents, and local geography usually play a significant role influencing WGS and so on.
- The nonparametric GKDE outperformed parametric models in capturing the marginal distribution behavior. Silverman’s ROT, LSCV, and UCV were recognized as the most suitable bandwidth selectors in estimating GKDE margins at most of the selected stations. Besides, the chosen marginal pdf type varies between different time lags, even at the same station. Some stations have the same model type (i.e., GKDE with same bandwidth selector) for all selected time lags.
- Different 2-D parametric class copulas were chosen based on the CvM-based GOF test for extreme pairs AMWGS-MT and AHMT-WGS at different lags. The selected copulas effectively captured the natural dependence. At the same station, different time lags resulted in variation in the chosen copula family or class (also their estimated dependence parameters) for each bivariate extreme pair.
- In most of the selected cities, the AMWGS or AHMT quantiles were higher or of sufficient magnitude at lower univariate RPs with higher annual exceedance probabilities. For instance, Regina exhibited higher AMWGS or elevated AHMT at lower univariate RPs, suggesting that this area would pose a higher risk of PBs and HACDs during summer periods and where the occurrence of both events is considered independently based on the univariate risk approach. Montreal, Quebec City, Toronto, and Vancouver City also had high AMWGS or AHMT quantiles at lower univariate RPs. Quebec City had a higher risk of PBs than Ottawa, Edmonton, and Montreal. On the other hand, Toronto had the highest risk of HACDs, followed by Montreal and Vancouver City.
- Combining bivariate pairs AMWGS-MT of EVSG-1 or AHMT-WGS of EVSG-2 at most stations indicates a higher risk of simultaneous PB and HACD events. Joint stress levels vary over different time lags and for different stations. The most stressful time lag for AMWGS-MT pairs was observed at Montreal at 0 days. In Quebec City, the joint action poses a higher risk than in Montreal and Ottawa. The bivariate AMWGS-MT events would be more stressful with a time lag of ± 1 day for both Quebec City and Ottawa. The joint action of AHMT-WGS in Toronto would be stressful at lag ± 2 days. During this time frame, the bivariate events were identifiable by lower AND-RPs (highly likely) and moderate risk levels of WGS, with higher AHMT. In Edmonton, bivariate AMWGS-MT events would be more stressful at ± 1 days. However, Montreal had a lower AND-joint RP than Edmonton, with higher AHMT and WGS design values. Therefore, Montreal has a higher risk level in joint action at a time lag of 0 days. In Regina, bivariate events AHMT-WGS may cause the most stress at ± 2 to ± 3 days, while pair AMWGS-MT would be more stressful at ± 1 days. In Vancouver, the lowest AND-joint RPs for events AHMT-WGS were observed with a time lag of ± 1 day, but the associated WGS quantiles at this time lag are lower and thus would pose a lower joint risk than other stations.
- The study examined the joint risk of AMWGS or AHMT given MT or WG events at different percentile values. The impact of the joint action would be greater in Quebec City than in Montreal. Ottawa has significantly higher conditional RPs (than Montreal or Quebec City). Similarly, higher AHMT levels were observed in Toronto. The joint distribution of AHMT events conditional on the 75th percentile (moderate threat level) and 90th percentile (higher intermediate threat level) of WGS events indicated a moderate risk level due to their combined occurrence. Conversely, Vancouver City had a low threat level for WGS events at the 75th and 90th percentiles. Still, when combined with higher AHMT events through conditional relation, the joint probability risk increases significantly. However, Vancouver City has a lower joint probability risk of AHMT-WGS bivariate events than Toronto City. Regina station has a moderate risk of WGS events, with wind gusts at the 75th and 50th percentiles. When combined with higher AHMT events through conditional joint relationships, the joint RPs were very low, indicating that joint action of AHMT-WGS would be much more stressful in Regina than in Toronto and Vancouver City. High WGS and AHMT levels in Montreal had moderately threatening effects, but the combined stress was not severe. This was because high conditional joint RPs were obtained when compounded AHMT with the 90th percentile of WGS at a 0 day time lag.

The study analysed the risks associated with different events based on historical observations. However, the proposed model did not account for changes that may occur over time due to climate change or other external factors. The model also did not consider the variation in mean and variance over time or any external covariate that could cause non-stationarity within the estimated joint exceedance probability or return periods. This limitation requires further investigation to develop a dynamic or time-varying framework for estimating bivariate distributions. Future studies should address this shortcoming and provide insights into the impact of climate change and other external factors on combined risks. Another concern is that the present study focused on maximum temperature (MT) of the day through annual maximum sampling procedure to highlight demands for air conditioning within communities during summer period without taking the accountability of relative humidity (RH). Heat stress occurs when the body faces high temperatures and RH simultaneously. High heat input can strain the body’s responses, and high RH limits sweating efficiency, making it difficult for the body to cool down. Air conditioning improves comfort by reducing humidity and enhancing cooling efficiency. The combination of temperature and relative humidity is called humidex^92^ in Canada and the heat index^93^ globally. The humidex can help us better understand how heat and humidity together impact our comfort level or demands of air-cooling. Assessing the joint probability relationship by considering RH along with extreme WGS and MT events together in a trivariate copula joint distribution can provide a more comprehensive understanding of the compounded risks in assessing the problem of power blackouts and heightened air-cooling demands. This approach would also be more effective to integrate wind gust direction accountability with WGS and temperature datasets. The highly flexible vine copula density can effectively model the compounded joint probability action of the suggested variables and their multivariate hazard risk modelling.

Minimum winter temperatures in Canada can also have significant effects, such as the impacts on public health (see, for instance, Bayentin^94^). Extreme cold spells can have devastating impacts if compounded with extreme wind gusts that lead to the destruction of the power distribution systems and the absence of heating during winter. Future research should also focus on the study of the risks associated with compound minimum temperatures and extreme wind speeds.

### Supplementary Information

Supplementary Information.

## Data Availability

The wind gust speed (WGS) and maximum temperature (MT) datasets used in this study are freely available for download from Environment and Climate Change Canada (https://climate.weather.gc.ca/). R-Studio software was used for data analysis in this study and is freely available at https://rstudio.com/products/rstudio/download/.

## Abbreviations

WGS: Wind gust speed
MT: Maximum temperature
GKDE: Gaussian kernel density estimation
PBs: Power blackouts
HACDs: Heightened air-conditioning demands
SiROT: Silverman’s rule-of-thumb
ScROT: Scott’s rule-of-thumb
STE: Solve-the-equation
DPI: Direct Plug-in
BCV: Biased cross-validation
UCV: Unbiased cross-validation
LSCV: Least square cross validation
NS: Normal scale
SCV: Smooth cross-validation
MLE: Maximum likelihood estimation
LMOM: Method-of-L-moments
MPL: Maximum pseudo likelihood
AIC: Akaike information criterion
BIC: Bayesian information criterion
HQC: Hannan-Quinn information criterion
$\theta$: Copula dependence parameter
${\Phi }^{-1}$: Inverse of generator function of Archimedean copulas
$\Phi (\cdot )$: Generator function of Archimedean copulas
