# Supplementary material for: Compounded wind gusts and maximum temperature via semiparametric copula in the risk assessments of power blackouts and air conditioning demands for major cities in Canada
Source: Sci Rep. 2024 Jul 1;14:15031. doi: 10.1038/s41598-024-65413-6 (PMC11217475; doi:10.1038/s41598-024-65413-6)
Supplement: Supplementary file 1 — Supplementary Information. [file 41598_2024_65413_MOESM1_ESM.docx]

**Supplementary** **Information (SI)**

**Compounded Wind Gusts and Maximum Temperature via Semiparametric copula in the risk assessments of power blackouts and air conditioning demands for major cities in Canada**

Shahid Latif^1*^ and Taha B.M.J. Ouarda^1^

^1^Canada Research Chair in Statistical Hydro-Climatology, Institut national de la recherche scientifique, Centre Eau Terre Environnement, INRS-ETE, 490 De la Couronne, Québec City, QC, G1K 9A9, Canada

*Corresponding author Email address: [Md_Shahid.LATIF@inrs.ca](mailto:Md_Shahid.LATIF@inrs.ca)

**This Supplementary material contains the following information:**

- **Supplementary Appendix (SA) (SA1-SA6)**
- **Supplementary Table (ST) (ST 1- ST6)**
- **Supplementary Figure (SF) (SF 1-SF 11)**

**Supplementary Appendix (SA)**

**SA1: Positively dependence parametric class 2-D copulas in the joint density modelling of bivariate extreme pairs**

This present study selected positively dependence parametric copulas includes Archimedean class copulas and their mixed version (i.e., Frank, Clayton, Gumbel, AMH (Ali–Mikhail–Haq copula), Joe, BB1, BB6, BB7 and BB8), rotated mixed Archimedean by 180 degrees (also called Survival copulas) (i.e., Survival BB1, Survival BB6, Survival BB7 and Survival BB8), rotated mono-parametric Archimedean by 180 degrees (i.e., Survival Clayton, Survival Gumbel and Survival Joe), extreme value (EV) class Tawn (Tawn type-1 and Tawn type-2) copula, rotated Tawn by 180 degrees (Survival Tawn type-1 and Survival Tawn type-2 copula) (Li 2016; Tang et al., 2015; Nikoloulopoulos et al., 2012; Reddy and Ganguli 2012; Manner 2010; Constantino 2008; Zhang and Singh 2007, 2006; Joe 1997; Joe et al., 1996; Tawn 1988). Rotation by 180 degrees is applicable to capture positive dependencies. The adequacy of Elliptical class-based Normal copula was also tested, which exhibit symmetrical tail dependence with zero upper and lower tail dependence structure (AghaKouchak et al., 2012; McNeil et al., 2005). Mixture Archimedean copulas usually possess higher flexibility than mono-parametric Archimedean class (Latif and Simonovic 2022). For instance, BB1, BB7 and BB6 copula can easily capture upper tail dependence behaviour for WGS and MT events. Besides, Survival BB1 and Survival BB7 copulas can also effectively capture the upper tail portion for WGS and MT events. Besides, Gumbel and Joe copula can accommodate the upper tail extreme portion for given bivariate pairs. The Joe copula can effectively capture joint structure in case of higher positive dependency. Clayton and Frank copula have no upper tail dependency, whereas Frank copula can accommodate the entire dependence range, $\mathrm{Frank}_{\tau_{\theta}}\in\left[ -1, 1 \right]$, while Clayton copula only accommodates a positive dependence range $\mathrm{Clayton}_{\tau_{\theta}}\in\left[ 0, 1 \right]$ and can capture lower tail dependence (Lee et al., 2013).

Mathematically, if $z_{1}$ and $z_{2}$ are the marginal vector, the rotation by 180 degrees is given by

$C^{180}=C(1-z_{1}, 1-z_{2})$ (1)

**SA2: Estimating unknown dependence parameter fitted 2-D copulas for bivariate pairs AMWGS-MT (for EVSG-1) and AHMT-WGS (for EVSG-2) at selected time lag for all selected cities.**

The selected candidate copulas' vector of unknown dependence parameters was estimated via rank-based distribution by maximizing the pseudo-log-likelihood functions, called maximum pseudo-likelihood (MPL) estimation (Latif et al., 2023; Latif and Simonovic 2022; Reddy and Ganguli 2012; Klein et al., 2010).

$Pseudo log-likelihood function= l\left( \theta\right)=\sum_{v=1}^{n} log[C_{\theta}\left\{ F_{1}\left( Z_{v,1} \right), F_{2}\left( Z_{v,2} \right) \right\}$ (1)

Where, $F_{1}\left( Z_{v,1} \right) and F_{2}\left( Z_{v,2} \right)$ are the empirical CDFs. The copula dependence parameters can be estimated by maximizing this rank-based pseudo-log-likelihood function, which is calculated by.

$\hat{\theta}=argmax[lnL_{v}\left( \theta\right)]$ (2)

**SA3: Performance evaluation of fitted 2D copula density functions via Cramer von Mises (CvM) functional test statistics** $\boldsymbol{S}_{\boldsymbol{n}}$ **with parametric bootstrapping procedure.**

The performance evaluation of fitted candidate copulas was examined using the Cramer von Mises (CvM) function test statistics S_n_ with a parametric bootstrapping procedure (Latif and Simonovic 2022; Ganguli and Reddy 2012)

$S_{n}=n\int_{{[0,1]}^{2}}\left\{ c_{n}\left( u,v \right)-c_{\theta}\left( u,v \right) \right\}^{2}dC_{n}\left( u,v \right)=\sum_{i=1}^{n} \left\{ C_{n}\left( U_{i,n},V_{i, n} \right)-C_{\theta}\left( U_{i,n},V_{i, n} \right) \right\}^{2}$ (1)

$C_{\theta}$ is the parametric 2-D copula derived under the null hypothesis, $H_{o}$, where u and v are the univariate marginal distribuiton. $c_{n}\left( u,v \right)$, are the bivariate empirical copulas estimated using n observational random pairs. Similarly, $U_{i,n},{and V}_{i, n}$ are the pseudo-observations of C transformed from $\left( X_{1}, Y_{1} \right), \left( X_{2}, Y_{2} \right),\ldots.., \left( X_{n}, Y_{n} \right)$ where the numerical value of $U_{i,n},V_{i, n}$ are estimated by

$U_{i,n}=\frac{1}{n+1}\sum_{j=1}^{n} 1(X_{j}\leq X_{i}); V_{i,n}=\frac{1}{n+1}\sum_{j=1}^{n} 1(Y_{j}\leq Y_{i}) , i\in\left\{ 1, 2,\ldots, n \right\}$ (2)

N represents the number of simulations. To incorporate CvM statistics, p-values must be estimated for each selected copula density, which is mathematically calculated by.

$P=\frac{1}{N}\sum_{i=1}^{n} 1(S_{n,t}\geq S_{n})$ (3)

The acceptance or rejection of candidate copulas is contingent on estimated S_n_ values (and p-value). To ensure a satisfactory selection and suitability of 2-D copula density, the minimum value of S_n_ statistics with p-values greater than 0.05 - based on a 5% significance level - must be attained, with any value below this threshold warranting rejection.

**SA4: Bandwidth estimation procedure of fitted GKDE models in the univariate marginal distribution of AMWGS and corresponding MT events (for EVSG-1), and AHMT and corresponding WGS (for EVSG-2).**

The mean integrated square error (MISE) statistics are used to outline the performance of fitted density, $\hat{f}$ is estimated by (Han et al., 2019; Chen 2015; Qin et al., 2011; Karmakar and Simonovic 2008).

$MISE=E\left( \int{(\hat{f}\left( z \right)-f\left( z \right))}^{2}\mathrm{dz} \right)\approx\frac{R\left( K \right)}{\mathrm{nh}}+\frac{h^{4}\mu_{2}^{2}\left( K \right)R(f^{'})}{4}$ (1)

The kernel bandwidth is usually estimated to minimize the MISE of Equation (1). The SiROT bandwidth is estimated by.

$h_{Silverman ROT}=0.9*min\left( \sigma,\frac{interquartile range \left( \mathrm{IQR} \right)}{1.35} \right)*n^{-\frac{1}{5}}$ (2)

Where $\sigma$ is the standard deviation (SD). While the Scott's ROT bandwidth is estimated by

$h_{Scott ROT}=1.06*\sigma*n^{-\frac{1}{5}}$ (3)

Both SiROT and ScROT methods are straightforward to calculate. Furthermore, the optimal bandwidth for the NS bandwidth selector is estimated by (Chen 2015; Zamborn and Dias 2013)

$h_{Normal scale}=\left( \frac{4}{3n} \right)^{0.2}*IQR$ (4)

Similarly, using Equation (9), the LSCV bandwidth selector is constructed, followed by Bowman (1984)

$LSCV=\int\hat{f}^{2}\left( z \right)d\left( z \right)-\frac{2}{n}\sum_{i} \hat{f}_{-i}(Z_{i})$ (5)

For the LSCV selector, it is required to examine the estimate of LSCV(h) from the given historical datasets and maximize it over h. the optimal bandwidth is estimated by

$h_{Least Square cross validation=}\begin{matrix} \mathrm{argmin} \\ h \end{matrix}LSCV(h)$ (6)

**SA5: Model compatibility test among the fitted 1-D marginal probability distribution.**

The AIC, BIC and HQC statistics are estimated mathematically by

$AIC=Nlog\left( \mathrm{MSE} \right)+[2*\log\left( N \right)]$ (1)

$BIC=Nlog\left( \mathrm{MSE} \right)+[k*\log\left( N \right)]$ (2)

$HQC=Nlog\left( \mathrm{MSE} \right)+[2*k*\log\left( \log\left( N \right) \right)]$ (3)

N is the sample size, and k is the number of fitted parameters. The minimum AIC, BIC, HQC, and MSE test values must indicate better performance.

**SA6: Estimating primary joint return periods for bivariate OR- and AND-hazard scenarios and conditional joint distribution, for bivariate distribution pairs AMWGS-MT (in case of EVSG-1) and AHMT-WGS (in case of EVSG-2) defined at different time lags. Estimating design quantiles corresponding to different return periods.**

Situation 1: When both AMWGS (or AHMT) and the corresponding MT (or WGS) events simultaneously exceed a specific threshold value (say, wgs or mt), i.e., $AMWGS\geq wgs AND MT\geq mt$, in case of EVSG-1 datasets group (or $AHMT\geq mt AND WGS\geq wgs, in case of EVSG-2 group)$, and called the AND-joint distribution case (or concurrence probability) and their associated RP are estimated for both extreme group EVSG-1 and EVSG-2.

$T_{EVSG-1 \left( AMWGS, MT \right)}^{\mathrm{AND}} (or T_{EVSG-2 \left( AHMT, WGS \right)}^{\mathrm{AND}}) =\frac{\mu=1 (for annual maxima-based analysis)}{1-F\left( \mathrm{AMWGS} \right)-F\left( \mathrm{MT} \right)+C(AMWGS, MT)}\left( or \frac{\mu=1 (for annual maxima-based analysis)}{1-F\left( \mathrm{WGS} \right)-F\left( \mathrm{AHMT} \right)+C(AHMT, WGS)} \right)$ (1)

Where, $F\left( \mathrm{AMWGS} \right)$and F(MT) are the marginal cdf of AMWGS, and corresponding MT events belong to EVSG-1. $F\left( \mathrm{AHMT} \right)$and F(WGS) are the marginal cdf of AHMT, and corresponding WGS events belong to the EVSG-2 group. In Equation 1, the denominator term ($F\left( \mathrm{AMWGS} \right)+F\left( \mathrm{MT} \right)-C\left( AMWGS, MT \right) (or(F\left( \mathrm{AHMT} \right)+F\left( \mathrm{WGS} \right)-C\left( AHMT, WGS \right))$, define joint non-exceedance probability (NEP) in the simultaneous occurrence of bivariate events.

Situation 2: When either WGS or MT exceeds a specific threshold value, $AMWGS\geq wgs OR MT\geq mt$ (or AHMT$\geq mt OR WGS\geq wgs)$ called the OR-joint distribution case, and their associated OR-joint RP are estimated by

$T_{EVSG-1 \left( AMWGS, MT \right)}^{\mathrm{OR}}(or T_{EVSG-2 \left( AHMT, WGS \right)}^{\mathrm{OR}})=\frac{\mu=1 (for annual maxima-based analysis)}{1-C(AMWGS, MT)}\left( or\frac{\mu=1 (for annual maxima-based analysis)}{1-C(AHMT, WGS)} \right)$ (2)

Situation 3: Understanding conditional joint distribution is crucial in bivariate hazard risk modelling. The joint probability distribution of AMWGS (or AHMT) conditional on the corresponding MT (or WGS) events were defined at different percentile values (50^th^, 75^th^, 100^th^). This study used a semiparametric copula density model to estimate the conditional joint RPs for two different situations.

- When conditional on MT events (in the case of the EVSG-1 group)

$T_{AMWGS|MT\geq mt}^{EVSG-1}=\frac{1}{(1-F\left( \mathrm{MT} \right)*(1-F\left( \mathrm{MT} \right)-F\left( \mathrm{AMWGS} \right)+C\left( F\left( \mathrm{AMWGS} \right), F\left( \mathrm{MT} \right) \right))}$ (3)

- When conditional on WGS events (in the case of the EVSG-2 group)

$T_{AHMT|WGS\geq wgs}^{EVSG-2}=\frac{1}{(1-F\left( \mathrm{WGS} \right)*(1-F\left( \mathrm{AHMT} \right)-F\left( \mathrm{WGS} \right)+C\left( F\left( \mathrm{AHMT} \right), F\left( \mathrm{WGS} \right) \right))}$ (4)

The design quantiles ($x_{T})$ corresponding to different univariate return period (RP) say T is given by.

$Exceedance probability (EP)=p=\frac{1}{T}=P(X\geq x)$ (5)

$Non-Exceedance probability\left( \mathrm{NEP} \right)=P\left( X\leq x \right)=F\left( x, \theta\right)=1-\frac{1}{T}$ (6)

$x=F^{-1}(1-\frac{1}{T};\theta)$ (7)

where, $F^{-1}$ is the inverse marginal CDF, $\theta$ is the model parameter of fitted univariate marginal distribution,

**References:**

AghaKouchak, A., Sellars, S., Sorooshian, S., (2012). Methods of Tail Dependence Estimation. Water Science and Technology Library, pp. 163–179. https://doi.org/ 10.1007/978-94-007-4479-0_6.

Chen, S. (2015). Optimal bandwidth selection for kernel density functional estimation. Journal of Probability and Statistics, 2015.

Constantino M, Larran M, Brebbia CA (2008) Computational fnance and its applications III, Volume 41 of WIT transactions on information and communication technologies, WIT Press, 2008.

Genest C, Rémillard B, Beaudoin D (2009) Goodness-of-ft tests for copulas: a review and a power study. Insur Math Econ 44:199–214.

Genest C, Rémillard B (2008) Validity of the parametric bootstrap for goodness-of-ft testing in semiparametric models. Ann L’inst Henri Poincare Prob Stat 44:1096–1127.

Han, Q., Ma, S., Wang, T., & Chu, F. (2019). Kernel density estimation model for wind speed probability distribution with applicability to wind energy assessment in China. Renewable and Sustainable Energy Reviews, 115, 109387.

Joe, H., (1997). Multivariate Models and Multivariate Dependence Concepts. CRC press.

Joe, H., Hu, T., (1996). Multivariate distributions from mixtures of max-infinitely divisible distributions. J. Multivariate Anal. 57 (2), 240–265. https://doi.org/10.1006/jmva.1996.0032.

Karmakar, S., & Simonovic, S. P. (2008). Bivariate flood frequency analysis: Part 1. Determination of marginals by parametric and nonparametric techniques. Journal of Flood Risk Management, 1(4), 190-200.

Klein, B., Pahlow, M., Hundecha, Y., Schumann, A., 2010. Probability Analysis of Hydrological Loads for the Design of Flood Control Systems Using Copulas. J. of Hydrol. Eng. 15 (5), 360–369. <https://doi.org/10.1061/(asce)he.1943-> 5584.0000204.

Latif, S., & Simonovic, S. P. (2022). Parametric Vine copula framework in the trivariate probability analysis of compound flooding events. Water, 14(14), 2214.

Latif, S., Souaissi, Z., & Ouarda, T. B. (2023). Copula-based joint modelling of extreme river temperature and low flow characteristics in the risk assessment of aquatic life. *Weather and Climate Extremes*, 100586.

Latif, S., & Simonovic, S. (2022). Trivariate probabilistic assessments of the compound flooding events using Semiparametric Fully Nested Archimedean (FNA) copula approach.

Lee, T., Modarres, R. and Ouarda, T.B.M.J. (2013). Data-based analysis of bivariate copula tail dependence for drought duration and severity. Hydrol. Process., 27(10), 1454-1463, DOI:10.1002/hyp.9233

Li F (2016) Modeling covariate-contingent correlation and taildependence with copulas. https://arxiv.org/pdf/1401.0100.pdf.

Manner H (2010) Modelling assymetric and time-varying dependence. https://cris.maastrichtuniversity.nl/portal/fles/667227/ guid-ae8195ad-cf0b%20–4744–8bb1–6a44fbe10fe7-ASSET1.0.

McNeil, J., Frey, R., Embrechts, P., (2005). Quantitative Risk Management: Concepts, Techniques and Tools. Princeton University Press, Princeton, NJ, U.S.A., 2005.Bowman, A. W. (1984). An alternative method of cross-validation for the smoothing of density estimates. Biometrika, 71(2), 353-360.

Nikoloulopoulos, A.K., Joe, H., Li, H., (2012). Vine copulas with asymmetric tail dependence and applications to financial return data. Comput. Stat. Data Anal. 56 (11), 3659–3673. https://doi.org/10.1016/j.csda.2010.07.016.

Qin, Z., Li, W., & Xiong, X. (2011). Estimating wind speed probability distribution using kernel density method. *Electric Power Systems Research*, *81*(12), 2139-2146.

Reddy MJ, Ganguli P (2012) Bivariate flood frequency analysis of Upper Godavari River flows using Archimedean copulas. Water Resour Manage. <https://doi.org/10.1007/s11269-012-0124-z>.

Tang Y, Huynh VN, Lawry J (2015) Integrated uncertainty in knowledge modelling and decision making. In: 4th International Symposium, Proceedings, LNAI 9376, Springer-Verlag.

Tawn, J.A., 1988. Bivariate extreme value theory: models and estimation. Biometrika 75 (3), 397–415. https://doi.org/10.1093/biomet/75.3.397.

Zamborn A, Dias R. A review of kernel density estimation with applications to econometrics. International Econometric Review 2013; 5:20–42.

Zhang L, Singh VP (2006) Bivariate flood frequency analysis using copula method. J Hydrol Eng 11(2):150. https://doi.org/10.1061/(ASCE)1084-0699(2006)11:2(150)

Zhang L, Singh VP (2007) Trivariate flood frequency analysis using the Gumbel-Hougaard copula. J Hydrol Eng 12(4):431–439. https://doi.org/10.1061/(ASCE)1084-0699(2007)12:4(431)

**Supplementary Table (ST)**

ST 1**:** Geographical location (with characteristics) of the selected major cities in Canada used in this study.

| Selected Geographical region of Canada | Case study cities (location of weather gauge station) | Geographical Coordinates | | Elevations (meters) | Climate ID (WMO ID) | Data availability Time interval |
| --- | --- | --- | --- | --- | --- | --- |
|  |  | Latitude (Decimal Degrees) | Longitude (Decimal Degrees) |  |  |  |
| Central Canada | Montreal (MONTREAL/PIERRE ELLIOTT TRUDEAU INTL A) | 45.47 | -73.75 | 36.00 | 7025250 (71627) | 1955-1995 |
|  | Quebec City (QUEBEC/JEAN LESAGE INTL A) | 46.8 | -71.38 | 60.00 | 701S001(71714) | 1959-1991 |
|  | Ottawa (OTTAWA MACDONALD-CARTIER INT’L A) | 45.32 | -75.67 | 114.00 | 6106000(71628) | 1955-2011 |
|  | Toronto (TORONTO ISLAND A) | 43.63 | -79.4 | 76.50 | 6158665 (71265) | 1970-2004 |
| Atlantic Canada | Halifax (HALIFAX STANFIELD INT’L A) | 44.88 | -63.5 | 145.40 | 8202250 (NA) | 1961-2012 |
| West coast (Pacific) | Vancouver (VANCOUVER INT’L A) | -123.18 | 49.2 | 4.30 | 1108447 (NA) | 1955-2013 |
| Canadian Prairies | Calgary (CALGARY INT’L A) | -114.02 | 51.11 | 1084.10 | 3031093 (NA) | 1955-2011 |
|  | Edmonton (EDMONTON CITY CENTRE A) | -113.52 | 53.57 | 670.60 | 3012208 (NA) | 1955-1991 |
|  | Regina (Regina INT’T Airport) | -104.67 | 50.43 | 577.60 | 4016560 (NA) | 1955-1995 |

ST2: Stationwise correlation measures for bivariate extreme sample of EVSG-1 and EVSG-2 (a) Montreal (b)Quebec City(c) Ottawa(d) Toronto(e) Halifax (f) Regina(g) Edmonton(h) Calgary (i) Vancouver

| 1. EVSG-1 (Montreal) | | | | |
| --- | --- | --- | --- | --- |
| PEARSON $\boldsymbol{r}$ correlation measure  (Annual maximum WGS (km/hr)) | Corresponding highest MT (Time lag = 0days) | Corresponding highest MT (Time lag = $\pm$1days) | Corresponding highest MT (Time lag = $\pm2days$) | Corresponding highest MT (Time lag = $\pm3days$) |
|  | 0.1722423 | 0.1281539 | 0.2155188 | 0.07504514 |
| KENDALL’S $\boldsymbol{\tau}$  Correlation measure  (Annual maximum WGS (km/hr)) | Corresponding highest MT (Time lag = 0day) | Corresponding highest MT (Time lag = $\pm$1days) | Corresponding highest MT (Time lag = $\pm2days$) | Corresponding highest MT (Time lag = $\pm3$days) |
|  | 0.1626238 | 0.09803922 | 0.1334206 | 0.09305405 |
| SPEARMAN’S $\boldsymbol{\rho}$  Correlation measure  (Annual maximum WGS (km/hr)) | Corresponding highest MT (Time lag = 0day) | Corresponding highest MT (Time lag = $\pm$1days) | Corresponding highest MT (Time lag = $\pm2$days) | Corresponding highest MT (Time lag = $\pm3days$) |
|  | 0.2065059 | 0.1451598 | 0.1960711 | 0.1392911 |
| EVSG-2 (Montreal region) | | | | |
| PEARSON $\boldsymbol{r}$ correlation measure  (Annual Highest MT (°C)) | Corresponding Maximum WGS (Time lag = 0day) | Corresponding Maximum WGS (Time lag = $\pm$1days) | Corresponding Maximum WGS (Time lag = $\pm2days$) | Corresponding Maximum WGS (Time lag = $\pm3days$) |
|  | 0.2628014 | 0.1037331 | 0.142345 | 0.1174951 |
| KENDALL’S $\boldsymbol{\tau}$  Correlation measure  (Annual Highest MT (°C)) | Corresponding Maximum WGS (Time lag = 0day) | Corresponding Maximum WGS (Time lag = $\pm$1days) | Corresponding Maximum WGS (Time lag = $\pm2days$) | Corresponding Maximum WGS (Time lag = $\pm3days$) |
|  | 0.1583508 | 0.03446082 | 0.02251727 | 0.002642155 |
| SPEARMAN’S $\boldsymbol{\rho}$  Correlation measure  (Annual Highest MT (°C)) | Corresponding Maximum WGS (Time lag = 0day) | Corresponding Maximum WGS (Time lag = $\pm$1days) | Corresponding Maximum WGS (Time lag = $\pm2days$) | Corresponding Maximum WGS (Time lag = $\pm3$days) |
|  | 0.2303887 | 0.04524699 | 0.02584626 | 0.006071168 |

| 1. EVSG-1 (Quebec City) | | | | |
| --- | --- | --- | --- | --- |
| PEARSON $\boldsymbol{r}$ correlation measure  (Annual maximum WGS (km/hr)) | Corresponding highest MT (Time lag = 0 day) | Corresponding highest MT (Time lag = $\pm$1 days) | Corresponding highest MT (Time lag = $\pm2 days$) | Corresponding highest MT (Time lag = $\pm3$ days) |
|  | 0.2994686 | 0.3031576 | 0.2958471 | 0.3120666 |
| KENDALL’S $\boldsymbol{\tau}$  Correlation measure  (Annual maximum WGS (km/hr)) | Corresponding highest MT (Time lag = 0 day) | Corresponding highest MT (Time lag = $\pm$1 days) | Corresponding highest MT (Time lag = $\pm2 days$) | Corresponding highest MT (Time lag = $\pm3 days$) |
|  | 0.2363122 | 0.1990347 | 0.1884619 | 0.19865 |
| SPEARMAN’S $\boldsymbol{\rho}$  Correlation measure  (Annual maximum  WGS (km/hr)) | Corresponding highest MT (Time lag = 0 day) | Corresponding highest MT (Time lag = $\pm$1 days) | Corresponding highest MT (Time lag = $\pm2 days$) | Corresponding highest MT (Time lag = $\pm3$ days) |
|  | 0.3493976 | 0.2716691 | 0.2542259 | 0.2874006 |
| EVSG-2 (Quebec City region) | | | | |
| PEARSON $\boldsymbol{r}$ correlation measure  (Annual Highest MT (°C)) | Corresponding Maximum WGS (Time lag = 0 day) | Corresponding Maximum WGS (Time lag = $\pm$1 days) | Corresponding Maximum WGS (Time lag = $\pm2 days$) | Corresponding Maximum WGS (Time lag = $\pm3 days$) |
|  | 0.002052602 | -0.05249976 | 0.0520765 | 0.1057078 |
| KENDALL’S $\boldsymbol{\tau}$  Correlation measure  (Annual Highest MT (°C)) | Corresponding Maximum WGS (Time lag = 0 day) | Corresponding Maximum WGS (Time lag = $\pm$1 days) | Corresponding Maximum WGS (Time lag = $\pm2 days$) | Corresponding Maximum WGS (Time lag = $\pm3 days$) |
|  | 0.01002006 | -0.05726039 | 0.04130371 | 0.0492187 |
| SPEARMAN’S $\boldsymbol{\rho}$  Correlation measure  (Annual Highest MT (°C)) | Corresponding Maximum WGS (Time lag = 0 day) | Corresponding Maximum WGS (Time lag = $\pm$1 days) | Corresponding Maximum WGS (Time lag = $\pm2 days$) | Corresponding Maximum WGS (Time lag = $\pm3 days$) |
|  | 0.003628695 | -0.07725314 | 0.0688848 | 0.0830957 |

| 1. EVSG-1 (Ottawa) | | | | |
| --- | --- | --- | --- | --- |
| PEARSON $\boldsymbol{r}$ correlation measure  (Annual maximum WGS (km/hr)) | Corresponding highest MT (Time lag = 0 day) | Corresponding highest MT (Time lag = $\pm$1 days) | Corresponding highest MT (Time lag = $\pm2 days$) | Corresponding highest MT (Time lag = $\pm3 days$) |
|  | 0.1152766 | 0.1858249 | 0.1678671 | 0.1276688 |
| KENDALL’S $\boldsymbol{\tau}$  Correlation measure  (Annual maximum WGS (km/hr)) | Corresponding highest MT (Time lag = 0 day) | Corresponding highest MT (Time lag = $\pm$1 days) | Corresponding highest MT (Time lag = $\pm2 days$) | Corresponding highest MT (Time lag = $\pm3 days$) |
|  | 0.07795756 | 0.1313278 | 0.1070865 | 0.08712478 |
| SPEARMAN’S $\boldsymbol{\rho}$  Correlation measure  (Annual maximum WGS (km/hr)) | Corresponding highest MT (Time lag = 0 day) | Corresponding highest MT (Time lag = $\pm$1 days) | Corresponding highest MT (Time lag = $\pm2 days$) | Corresponding highest MT (Time lag = $\pm3 days$) |
|  | 0.1190963 | 0.197487 | 0.1669102 | 0.1302859 |
| EVSG-2 (OTTAWA) | | | | |
| PEARSON $\boldsymbol{r}$ correlation measure  (Annual Highest MT (°C)) | Corresponding Maximum WGS (Time lag = 0 day) | Corresponding Maximum WGS (Time lag = $\pm$1 days) | Corresponding Maximum WGS (Time lag = $\pm2 days$) | Corresponding Maximum WGS (Time lag = $\pm3 days$) |
|  | -0.09687986 | 0.0140991 | -0.02571278 | 0.02073118 |
| KENDALL’S $\boldsymbol{\tau}$  Correlation measure  (Annual Highest MT (°C)) | Corresponding Maximum WGS (Time lag = 0 day) | Corresponding Maximum WGS (Time lag = $\pm$1 days) | Corresponding Maximum WGS (Time lag = $\pm2 days$) | Corresponding Maximum WGS (Time lag = $\pm3 days$) |
|  | -0.05147489 | 0.01294577 | -0.02398895 | 0.0155604 |
| SPEARMAN’S $\boldsymbol{\rho}$  Correlation measure  (Annual Highest MT (°C)) | Corresponding Maximum WGS (Time lag = 0 day) | Corresponding Maximum WGS (Time lag = $\pm$1 days) | Corresponding Maximum WGS (Time lag = $\pm2$ days) | Corresponding Maximum WGS (Time lag = $\pm3 days$) |
|  | -0.06450599 | 0.02940224 | -0.02602875 | 0.02271956 |

| 1. EVSG-1 (Toronto region) | | | | |
| --- | --- | --- | --- | --- |
| PEARSON $\boldsymbol{r}$ correlation measure  (Annual maximum WGS (km/hr)) | Corresponding highest MT (Time lag = 0 day) | Corresponding highest MT (Time lag = $\pm$1 days) | Corresponding highest MT (Time lag = $\pm2 days$) | Corresponding highest MT (Time lag = $\pm3 days$) |
|  | 0.07596 | 0.004108448 | -0.01565466 | 0.03832858 |
| KENDALL’S $\boldsymbol{\tau}$  Correlation measure  (Annual maximum WGS (km/hr)) | Corresponding highest MT (Time lag = 0 day) | Corresponding highest MT (Time lag = $\pm$1 days) | Corresponding highest MT (Time lag = $\pm2 days$) | Corresponding highest MT (Time lag = $\pm3 days$) |
|  | 0.05495427 | -0.001838434 | 0.01648628 | 0.04758426 |
| SPEARMAN’S $\boldsymbol{\rho}$  Correlation measure  (Annual maximum WGS (km/hr)) | Corresponding highest MT (Time lag = 0 day) | Corresponding highest MT (Time lag = $\pm$1 days) | Corresponding highest MT (Time lag = $\pm2 days$) | Corresponding highest MT (Time lag = $\pm3 days$) |
|  | 0.07505969 | 0.01533982 | 0.02598906 | 0.07466492 |
| EVSG-2 (Toronto region) | | | | |
| PEARSON $\boldsymbol{r}$ correlation measure  (Annual Highest MT (°C)) | Corresponding Maximum WGS (Time lag = 0 day) | Corresponding Maximum WGS (Time lag = $\pm$1 days) | Corresponding Maximum WGS (Time lag = $\pm2 days$) | Corresponding Maximum WGS (Time lag = $\pm3 days$) |
|  | 0.1389063 | 0.07610823 | 0.2679714 | 0.2917215 |
| KENDALL’S $\boldsymbol{\tau}$  Correlation measure  (Annual Highest MT (°C)) | Corresponding Maximum WGS (Time lag = 0 day) | Corresponding Maximum WGS (Time lag = $\pm$1 days) | Corresponding Maximum WGS (Time lag = $\pm2 days$) | Corresponding Maximum WGS (Time lag = $\pm3 days$) |
|  | 0.1054066 | 0.09957474 | 0.1883313 | 0.187026 |
| SPEARMAN’S $\boldsymbol{\rho}$  Correlation measure  (Annual Highest MT (°C)) | Corresponding Maximum WGS (Time lag = 0 day) | Corresponding Maximum WGS (Time lag = $\pm$1 days) | Corresponding Maximum WGS (Time lag = $\pm2 days$) | Corresponding Maximum WGS (Time lag = $\pm3 days$) |
|  | 0.1533575 | 0.1295283 | 0.2684093 | 0.2662181 |

| 1. EVSG-1 (Halifax region) | | | | |
| --- | --- | --- | --- | --- |
| PEARSON $\boldsymbol{r}$ correlation measure  (Annual maximum WGS (km/hr)) | Corresponding highest MT (Time lag = 0) day | Corresponding highest MT (Time lag = $\pm$1 days) | Corresponding highest MT (Time lag = $\pm2 days$) | Corresponding highest MT (Time lag = $\pm3$ days) |
|  | 0.07874638 | 0.1565295 | 0.173619 | 0.1496117 |
| KENDALL’S $\boldsymbol{\tau}$  Correlation measure  (Annual maximum WGS (km/hr)) | Corresponding highest MT (Time lag = 0 day) | Corresponding highest MT (Time lag = $\pm$1 days) | Corresponding highest MT (Time lag = $\pm2 days$) | Corresponding highest MT (Time lag = $\pm3$ days) |
|  | 0.08770344 | 0.0361174 | 0.02257338 | 0.02707586 |
| SPEARMAN’S $\boldsymbol{\rho}$  Correlation measure  (Annual maximum WGS (km/hr)) | Corresponding highest MT (Time lag = 0 day) | Corresponding highest MT (Time lag = $\pm$1 days) | Corresponding highest MT (Time lag = $\pm2 days$) | Corresponding highest MT (Time lag = $\pm3$ days) |
|  | 0.09423756 | 0.05199076 | 0.03204327 | .03785635 |
| EVSG-2 (Halifax region) | | | | |
| PEARSON $\boldsymbol{r}$ correlation measure  (Annual Highest MT (°C)) | Corresponding Maximum WGS (Time lag = 0 day) | Corresponding Maximum WGS (Time lag = $\pm$1 days) | Corresponding Maximum WGS (Time lag = $\pm2$ days) | Corresponding Maximum WGS (Time lag = $\pm3 days$) |
|  | -0.26255 | -0.4038373 | -0.3652568 | 0.09372683 |
| KENDALL’S $\boldsymbol{\tau}$  Correlation measure  (Annual Highest MT (°C)) | Corresponding Maximum WGS (Time lag = 0 day) | Corresponding Maximum WGS (Time lag = $\pm$1 days) | Corresponding Maximum WGS (Time lag = $\pm2 days$) | Corresponding Maximum WGS (Time lag = $\pm3$ days) |
|  | -0.21597 | -0.2725985 | -0.1858746 | -0.1304172 |
| SPEARMAN’S $\boldsymbol{\rho}$  Correlation measure  (Annual Highest MT (°C)) | Corresponding Maximum WGS (Time lag = 0 day) | Corresponding Maximum WGS (Time lag = $\pm$1 days) | Corresponding Maximum WGS (Time lag = $\pm2 days$) | Corresponding Maximum WGS (Time lag = $\pm3$ days) |
|  | 0.2922987 | -0.3647874 | -0.2480239 | -0.1013898 |

| 1. EVSG-1 (Regina region) | | | | |
| --- | --- | --- | --- | --- |
| PEARSON $\boldsymbol{r}$ correlation measure  (Annual maximum WGS (km/hr)) | Corresponding highest MT (Time lag = 0 day) | Corresponding highest MT (Time lag = $\pm$1 days) | Corresponding highest MT (Time lag = $\pm2 days$) | Corresponding highest MT (Time lag = $\pm3 days$) |
|  | 0.2517858 | 0.1791049 | 0.1835075 | 0.1819222 |
| KENDALL’S $\boldsymbol{\tau}$  Correlation measure  (Annual maximum WGS (km/hr)) | Corresponding highest MT (Time lag = 0 day) | Corresponding highest MT (Time lag = $\pm$1 days) | Corresponding highest MT (Time lag = $\pm2 days$) | Corresponding highest MT (Time lag = $\pm3 days$) |
|  | 0.2340558 | 0.1608912 | 0.1353199 | 0.1194038 |
| SPEARMAN’S $\boldsymbol{\rho}$  Correlation measure  (Annual maximum WGS (km/hr)) | Corresponding highest MT (Time lag = 0 day) | Corresponding highest MT (Time lag = $\pm$1 days) | Corresponding highest MT (Time lag = $\pm2 days$) | Corresponding highest MT (Time lag = $\pm3 days$) |
|  | 0.3295246 | 0.2071341 | 0.179514 | 0.1671685 |
| EVSG-2 (Regina region) | | | | |
| PEARSON $\boldsymbol{r}$ correlation measure  (Annual Highest MT (°C)) | Corresponding Maximum WGS (Time lag = 0 day) | Corresponding Maximum WGS (Time lag = $\pm$1 days) | Corresponding Maximum WGS (Time lag = $\pm2 days$) | Corresponding Maximum WGS (Time lag = $\pm3 days$) |
|  | 0.267124 | 0.196414 | 0.1732792 | 0.1262524 |
| KENDALL’S $\boldsymbol{\tau}$  Correlation measure  (Annual Highest MT (°C)) | Corresponding Maximum WGS (Time lag = 0 day) | Corresponding Maximum WGS (Time lag = $\pm$1 days) | Corresponding Maximum WGS (Time lag = $\pm2 days$) | Corresponding Maximum WGS (Time lag = $\pm3 days$) |
|  | 0.1846399 | 0.1457086 | 0.1343742 | 0.07121916 |
| SPEARMAN’S $\boldsymbol{\rho}$  Correlation measure  (Annual Highest MT (°C)) | Corresponding Maximum WGS (Time lag = 0 day) | Corresponding Maximum WGS (Time lag = $\pm$1 days) | Corresponding Maximum WGS (Time lag = $\pm2 days$) | Corresponding Maximum WGS (Time lag = $\pm3 days$) |
|  | 0.2448313 | 0.2034867 | 0.1982547 | 0.1180822 |

| 1. EVSG-1 (Edmonton region) | | | | |
| --- | --- | --- | --- | --- |
| PEARSON $\boldsymbol{r}$ correlation measure  (Annual maximum WGS (km/hr)) | Corresponding highest MT (Time lag = 0 day) | Corresponding highest MT (Time lag = $\pm$1 days) | Corresponding highest MT (Time lag = $\pm2 days$) | Corresponding highest MT (Time lag = $\pm3 days$) |
|  | 0.09779512 | 0.06421658 | -0.03298008 | -0.08054646 |
| KENDALL’S $\boldsymbol{\tau}$  Correlation measure  (Annual maximum WGS (km/hr)) | Corresponding highest MT (Time lag = 0 day) | Corresponding highest MT (Time lag = $\pm$1 days) | Corresponding highest MT (Time lag = $\pm2 days$) | Corresponding highest MT (Time lag = $\pm3 days$) |
|  | 0.1071977 | 0.07662855 | 0.06785132 | -0.001540878 |
| SPEARMAN’S $\boldsymbol{\rho}$  Correlation measure  (Annual maximum WGS (km/hr)) | Corresponding highest MT (Time lag = 0 day) | Corresponding highest MT (Time lag = $\pm$1 days) | Corresponding highest MT (Time lag = $\pm2 days$) | Corresponding highest MT (Time lag = $\pm3 days$) |
|  | 0.1291051 | 0.1033029 | 0.07249924 | -0.0127847 |
| EVSG-2 (Edmonton region) | | | | |
| PEARSON $\boldsymbol{r}$ correlation measure  (Annual Highest MT (°C)) | Corresponding Maximum WGS (Time lag = 0 day) | Corresponding Maximum WGS (Time lag = $\pm$1 days) | Corresponding Maximum WGS (Time lag = $\pm2$ days) | Corresponding Maximum WGS (Time lag = $\pm3 days$) |
|  | 0.006509779 | 0.008771324 | 0.112144 | 0.1137703 |
| KENDALL’S $\boldsymbol{\tau}$  Correlation measure  (Annual Highest MT (°C)) | Corresponding Maximum WGS (Time lag = 0 day) | Corresponding Maximum WGS (Time lag = $\pm$1 days) | Corresponding Maximum WGS (Time lag = $\pm2 days$) | Corresponding Maximum WGS (Time lag = $\pm3 days$) |
|  | 0.02545809 | -0.03577416 | 0.0357195 | 0.05128888 |
| SPEARMAN’S $\boldsymbol{\rho}$  Correlation measure  (Annual Highest MT (°C)) | Corresponding Maximum WGS (Time lag = 0 day) | Corresponding Maximum WGS (Time lag = $\pm$1 days) | Corresponding Maximum WGS (Time lag = $\pm2 days$) | Corresponding Maximum WGS (Time lag = $\pm3 days$) |
|  | 0.02204802 | -0.03207769 | 0.06027628 | 0.09439377 |

| 1. EVSG-1 (Calgary region) | | | | |
| --- | --- | --- | --- | --- |
| PEARSON $\boldsymbol{r}$ correlation measure  (Annual maximum WGS (km/hr)) | Corresponding highest MT (Time lag = 0 day) | Corresponding highest MT (Time lag = $\pm$1 days) | Corresponding highest MT (Time lag = $\pm2 days$) | Corresponding highest MT (Time lag = $\pm3 days$) |
|  | -0.1160151 | 0.07283216 | 0.05233625 | 0.0203222 |
| KENDALL’S $\boldsymbol{\tau}$  Correlation measure  (Annual maximum WGS (km/hr)) | Corresponding highest MT (Time lag = 0 day) | Corresponding highest MT (Time lag = $\pm$1 days) | Corresponding highest MT (Time lag = $\pm2 days$) | Corresponding highest MT (Time lag = $\pm3 days$) |
|  | -0.1153259 | 0.03765205 | 0.02747624 | 0.005749012 |
| SPEARMAN’S $\boldsymbol{\rho}$  Correlation measure  (Annual maximum WGS (km/hr)) | Corresponding highest MT (Time lag = 0 day) | Corresponding highest MT (Time lag = $\pm$1 days) | Corresponding highest MT (Time lag = $\pm2 days$) | Corresponding highest MT (Time lag = $\pm3 days$) |
|  | -0.1616991 | 0.05347872 | 0.0168925 | -0.01092072 |
| EVSG-2 (Calgary region) | | | | |
| PEARSON $\boldsymbol{r}$ correlation measure  (Annual Highest MT (°C) ) | Corresponding Maximum WGS (Time lag = 0 day) | Corresponding Maximum WGS (Time lag = $\pm$1 days) | Corresponding Maximum WGS (Time lag = $\pm2 days$) | Corresponding Maximum WGS (Time lag = $\pm3 days$) |
|  | 0.05991816 | 0.03534241 | -0.07960785 | -0.03550032 |
| KENDALL’S $\boldsymbol{\tau}$  Correlation measure  (Annual Highest MT (°C)) | Corresponding Maximum WGS (Time lag = 0 day) | Corresponding Maximum WGS (Time lag = $\pm$1 days) | Corresponding Maximum WGS (Time lag = $\pm2 days$) | Corresponding Maximum WGS (Time lag = $\pm3 days$) |
|  | 0.03521258 | -0.03171657 | -0.09482492 | -0.04273771 |
| SPEARMAN’S $\boldsymbol{\rho}$  Correlation measure  (Annual Highest MT (°C)) | Corresponding Maximum WGS (Time lag = 0 day) | Corresponding Maximum WGS (Time lag = $\pm$1 days) | Corresponding Maximum WGS (Time lag = $\pm2 days$) | Corresponding Maximum WGS (Time lag = $\pm3$ days) |
|  | 0.04651006 | -0.03655115 | -0.1202459 | -0.06050195 |

| 1. EVSG-1 (Vancouver region) | | | | |
| --- | --- | --- | --- | --- |
| PEARSON $\boldsymbol{r}$ correlation measure  (Annual maximum WGS (km/hr)) | Corresponding highest MT (Time lag = 0 day) | Corresponding highest MT (Time lag = $\pm$1 days) | Corresponding highest MT (Time lag = $\pm2 days$) | Corresponding highest MT (Time lag = $\pm3 days$) |
|  | -0.02034237 | 0.001599447 | -0.05506561 | -0.02555218 |
| KENDALL’S $\boldsymbol{\tau}$  Correlation measure  (Annual maximum WGS (km/hr)) | Corresponding highest MT (Time lag = 0 day) | Corresponding highest MT (Time lag = $\pm$1 days) | Corresponding highest MT (Time lag = $\pm2 days$) | Corresponding highest MT (Time lag = $\pm3 days$) |
|  | 0.04542798 | 0.0539244 | -0.0006203905 | 0.01555273 |
| SPEARMAN’S $\boldsymbol{\rho}$  Correlation measure  (Annual maximum WGS (km/hr)) | Corresponding highest MT (Time lag = 0 day) | Corresponding highest MT (Time lag = $\pm$1 days) | Corresponding highest MT (Time lag = $\pm2 days$) | Corresponding highest MT (Time lag = $\pm3 days$) |
|  | 0.07823129 | 0.07627217 | 0.01032772 | 0.02992832 |
| EVSG-2 (Vancouver region) | | | | |
| PEARSON $\boldsymbol{r}$ correlation measure  (Annual Highest MT (°C)) | Corresponding Maximum WGS (Time lag = 0 day) | Corresponding Maximum WGS (Time lag = $\pm$1 days) | Corresponding Maximum WGS (Time lag = $\pm2 days$) | Corresponding Maximum WGS (Time lag = $\pm3 days$) |
|  | 0.1038279 | 0.3024717 | 0.2346232 | 0.1614975 |
| KENDALL’S $\boldsymbol{\tau}$  Correlation measure  (Annual Highest MT (°C)) | Corresponding Maximum WGS (Time lag = 0 day) | Corresponding Maximum WGS (Time lag = $\pm$1 days) | Corresponding Maximum WGS (Time lag = $\pm2$ days) | Corresponding Maximum WGS (Time lag = $\pm3$ days) |
|  | 0.118292 | 0.2276333 | 0.1593892 | 0.07452412 |
| SPEARMAN’S $\boldsymbol{\rho}$  Correlation measure  (Annual Highest MT (°C)) | Corresponding Maximum WGS (Time lag = 0 day) | Corresponding Maximum WGS (Time lag = $\pm$1 days) | Corresponding Maximum WGS (Time lag = $\pm2$ days) | Corresponding Maximum WGS (Time lag = $\pm3$ days) |
|  | 0.1495825 | 0.2995688 | 0.2231366 | 0.1159082 |

Supplementary ST 3: Estimated 1-D parametric model distribution parameters via MLE and bandwidth of fitted GKDE model in the marginal distributions for station (a) Montreal (b) Quebec City (c) Ottawa (d) Toronto (e) Vancouver (f1 & f2) Regina (g) Edmonton

| (a )Fitted 1-D probability distribution (Montreal) | Annual maximum WGS (km/hr) | Highest MT (Time lag = 0 day) ($℃)$ | Highest MT (Time lag = ±1 days) ($℃)$ | Highest MT (Time lag = ±2 days) ($℃)$ | Highest MT (Time lag = ±3 days) ($℃)$ | Annual highest MT ($℃)$ | Corresponding maximum WGS (Time lag = 0) (km/hr) |
| --- | --- | --- | --- | --- | --- | --- | --- |
| Gamma-2p/LMOM | alpha beta  32.665693 2.525585 | alpha beta  41.1923154 0.6482398 | alpha beta  56.6620922 0.4965842 | alpha beta  67.2065451 0.4234707 | alpha beta  97.8112796 0.2948791 | alpha beta  497.51861288 0.06566086 | alpha beta  3.73569 11.24960 |
| GEV-3P/LMOM | xi alpha k  75.95258712 12.11118899 0.03830463 | xi alpha k  25.7061377 4.5261720 0.5143433 | xi alpha k  27.5260176 4.1999016 0.6672853 | xi alpha k  27.881065 3.898510 0.661041 | xi alpha k  28.0911553 3.1447967 0.4783176 | xi alpha k  32.0462754 1.3123590 0.1159485 | xi alpha k  35.002623 21.335030 0.320163 |
| Normal/LMOM | mu sigma  82.5000 14.3796 | mu sigma  26.702500 4.147883 | mu sigma  28.137500 3.729766 | mu sigma  28.460000 3.465147 | mu sigma  28.842500 2.912619 | mu sigma  32.667500 1.464206 | mu sigma  42.02500 21.02971 |
| NORMAL-2P/MLE | mean 82.50000 sd 14.32655 | mean 26.702500 sd 4.063095 | mean 28.137500 sd 3.749312 | estimate  mean 28.460000 sd 3.503484 | mean 28.842500  sd 2.854285 | estimate Std. Error  mean 32.667500 sd 1.488017 | estimate Std. Error  mean 42.02500 sd 21.28437 |
| LOGISTIC-2P/MLE | location 81.421453 scale 8.023828 | location 26.950080 scale 2.353999 | location 28.503059 scale 2.088418 | location 28.799459  scale 1.933791 | location 28.998342  scale 1.641128 | estimate Std. Error  location 32.5908773 scale 0.8076737 | estimate Std. Error  location 42.64532 scale 11.67684 |
| GEV/MLE | xi alpha kappa  76.16026317 11.86114312 0.04835383 | xi alpha kappa  25.8561500 4.4813684 0.5692258 | xi alpha kappa  27.7997405 4.3108674 0.8011786 | xi alpha kappa  28.0085833 3.9187407 0.7229846 | xi alpha kappa  28.2765202 3.1737028 0.5882701 | Estimated parameters:  location scale shape  32.0450049 1.3177168 -0.1062803 | Estimated parameters:  location scale shape  35.1630271 22.2104051 -0.3495419 |
| Gaussian KDE-Silverman Rule-of-thumb (ROT) Model | 5.379483 | 1.770864 | 1.525525 | 1.340856 | 1.244015 | 0.6021809 | 6.503554 |
| Gaussian KDE-Thumb Hand Rule by Scott (1992) Model | 6.335835 | 2.085684 | 1.796729 | 1.579231 | 1.465173 | 0.7092353 | 7.659741 |
| Gaussian KDE-Methods of Sheather & Jones (1991) via Direct Plug-in (DPI) Model | 6.718046 | 2.006226 | 1.728608 | 1.585232 | 1.563681 | 0.7324773 | 5.309888 |
| Gaussian KDE-Methods of Sheather & Jones (1991) via Solve-the-Equation (STE) Model | 6.758339 | 1.949948 | 1.716188 | 1.584848 | 1.581281 | 0.7374074 | 3.465909 |
| Gaussian KDE-Biased-cross validation (BCV) by Scott and Terrell (1987) Model | 7.904995 | 2.239163 | 2.063842 | 1.932867 | 1.573505 | 0.8215668 | 11.72254 |
| Gaussian KDE-Unbiased cross validation (UCV) by Scott and Terrell (1987) Model | 7.893545 | 2.241997 | 2.068853 | 1.933206 | 1.574981 | 0.8200262 | 1.77057 |
| Gaussian KDE-Least Square Cross Validation (LSCV) Model | 7.875403 | 2.247618 | 2.070988 | 1.935222 | 1.577171 | 0.8184734 | 1.780695 |
| Gaussian KDE-Normal Scale (NS) Model | 7.348776 | 2.084157 | 1.923203 | 1.797106 | 1.4641 | 0.7632754 | 10.91778 |
| Gaussian KDE-Smoothed cross-validation (SCV) model | 7.344594 | 2.079209 | 1.813605 | 1.700362 | 1.620667 | 0.7737903 | 6.847382 |

| (b) Fitted 1-D probability distribution (Quebec City) | Annual maximum WGS (km/hr) | Highest MT (Time lag = 0 day) ($℃)$ | Highest MT (Time lag = ±1 days) ($℃)$ | Highest MT (Time lag = ±2 days) ($℃)$ | Highest MT (Time lag = ±3 days) ($℃)$ |
| --- | --- | --- | --- | --- | --- |
| Gamma-2p/LMOM | alpha beta  13.562875 6.483831 | alpha beta  25.179049 1.018163 | alpha beta  32.9457986 0.7965333 | alpha beta  32.1994069 0.8281726 | alpha beta  60.9565273 0.4540245 |
| GEV-3P/LMOM | xi alpha k  74.9261691 14.2685598 -0.2559868 | 25.2381769 5.7911340 0.8389872 | xi alpha k  25.7909407 5.1850141 0.7970302 | xi alpha k  25.9188164 5.2772125 0.6756543 | xi alpha k  26.9024074 3.9033267 0.5578815 |
| Normal/LMOM | mu sigma  87.93939 23.65957 | 25.63636 5.08372 | mu sigma  26.242424 4.554669 | mu sigma  26.666667 4.681225 | mu sigma  27.675758 3.537522 |
| Normal-2P/MLE | mu sigma  87.93344 25.40533 | mean 25.636364 sd 5.248943 | mean 26.242424 sd 4.617689 | mean 26.66667  sd 4.66344 | mean 27.675758  sd 3.497188 |
| Logistic-2P/MLE | location 84.09916  scale 13.07670 | location 26.335797 scale 2.805511 | location 26.84940  scale 2.57238 | location 27.179159  scale 2.641887 | location 27.93362  scale 1.97438 |
| GEV/MLE | location scale shape  73.4717608 11.6247879 0.5356164 | location scale shape  25.1767067 5.7271578 -0.8060311 | location scale shape  25.7797964 5.0946503 -0.7824838 | location scale shape  25.9818139 5.2222988 -0.6881525 | location scale shape  26.9108891 3.8373497 -0.5482917 |
| Gaussian KDE-Silverman Rule-of-thumb (ROT) Model | 9.679054 | 1.835683 | 1.335042 | 1.335042 | 1.26829 |
| Gaussian KDE-Thumb Hand Rule by Scott (1992) Model | 11.39977 | 2.162026 | 1.572383 | 1.572383 | 1.493764 |
| Gaussian KDE-Methods of Sheather & Jones (1991) via Direct Plug-in (DPI) Model | 8.021532 | 1.988374 | 1.492738 | 1.507741 | 1.366206 |
| Gaussian KDE-Methods of Sheather & Jones (1991) via Solve-the-Equation (STE) Model | 6.412309 | 1.956727 | 1.458723 | 1.473639 | 1.08923 |
| Gaussian KDE-Biased-cross validation (BCV) by Scott and Terrell (1987) Model | 14.5956 | 3.019641 | 2.655193 | 2.674743 | 2.008482 |
| Gaussian KDE-Unbiased cross validation (UCV) by Scott and Terrell (1987) Model | 6.115967 | 2.499685 | 1.903539 | 1.985932 | 0.912341 |
| Gaussian KDE-Least Square Cross Validation (LSCV) Model | 6.152816 | 2.499365 | 1.905935 | 1.986197 | 0.4869091 |
| Gaussian KDE-Normal Scale (NS) Model | 13.57805 | 2.805684 | 2.468264 | 2.492719 | 1.86933 |
| Gaussian KDE-Smoothed cross-validation (SCV) model | 9.158793 | 2.19504 | 1.775433 | 1.864718 | 1.740704 |

| (c)Fitted 1-D probability distribution (Ottawa ) | Annual maximum WGS (km/hr) | Highest MT (Time lag = 0 day) ($℃)$ | Highest MT (Time lag = ±1 days) ($℃)$ | Highest MT (Time lag = ±2 days) ($℃)$ | Highest MT (Time lag = ±3 days) ($℃)$ |
| --- | --- | --- | --- | --- | --- |
| Gamma-2p/LMOM | alpha beta  33.152903 2.374961 | alpha beta  37.0323669 0.7251599 | alpha beta  55.2275310 0.5138868 | alpha beta  61.3267339 0.4703595 | alpha beta  68.6422156 0.4271833 |
| GEV-3P/LMOM | xi alpha k  72.08424428 10.55118819 -0.05132277 | xi alpha k  25.7662961 4.7793799 0.4997744 | xi alpha k  27.3668906 4.0923727 0.4617766 | xi alpha k  27.8824358 3.9586304 0.4700428 | xi alpha k  28.3256734 3.7510905 0.4285653 |
| Normal/LMOM | mu sigma  78.73684 13.62324 | mu sigma  26.85439 4.39804 | mu sigma  28.380702 3.810332 | mu sigma  28.845614 3.675954 | mu sigma  29.322807 3.532803 |
| Normal-2P/MLE | mean 78.73684  sd 14.73690 | mean 26.854386  sd 4.394337 | mean 28.380702  sd 3.768961 | mean 28.845614  sd 3.632991 | mean 29.322807  sd 3.500878 |
| Logistic-2P/MLE | location 77.235282  scale 7.427497 | location 27.11580 scale 2.46708 | location 28.578651 scale 2.145963 | estimate  location 29.039576  scale 2.071918 | location 29.46780 scale 1.98688 |
| GEV/MLE | location scale shape  72.18524649 10.67427132 0.03866296 | location scale shape  -287.323 2170.429 377.392 | location scale shape  23.32817 620.99938 162.21847 | location scale shape  -98.9492 818.2366 204.9452 | location scale shape  28.2728747 3.7061287 -0.3944096 |
| Gaussian KDE-Silverman Rule-of-thumb (ROT) Model | 4.488012 | 1.615684 | 1.196803 | 1.376324 | 1.41608 |
| Gaussian KDE-Thumb Hand Rule by Scott (1992) Model | 5.285881 | 1.902917 | 1.409568 | 1.621003 | 1.667828 |
| Gaussian KDE-Methods of Sheather & Jones (1991) via Direct Plug-in (DPI) Model | 5.558803 | 1.975858 | 1.561859 | 1.736309 | 1.749818 |
| Gaussian KDE-Methods of Sheather & Jones (1991) via Solve-the-Equation (STE) Model | 5.591767 | 1.982505 | 1.58675 | 1.748592 | 1.7582 |
| Gaussian KDE-Biased-cross validation (BCV) by Scott and Terrell (1987) Model | 6.403871 | 2.251265 | 1.92752 | 1.85995 | 1.78975 |
| Gaussian KDE-Unbiased cross validation (UCV) by Scott and Terrell (1987) Model | 6.987146 | 2.250035 | 1.9258 | 1.856734 | 1.791273 |
| Gaussian KDE-Least Square Cross Validation (LSCV) Model | 6.996564 | 2.255172 | 1.933292 | 1.862834 | 1.796645 |
| Gaussian KDE-Normal Scale (NS) Model | 7.015562 | 2.091942 | 1.794229 | 1.7295 | 1.666607 |
| Gaussian KDE-Smoothed cross-validation (SCV) model | 6.022985 | 2.126066 | 1.739159 | 1.810402 | 1.798677 |

| (d)Fitted 1-D probability distribution (Toronto) | Annual highest MT (km/hr) | Maximum WGS (Time lag = 0 day) ($℃)$ | Maximum WGS (Time lag = ±1 days) ($℃)$ | Maximum WGS (Time lag = ±2 days) ($℃)$ | Maximum WGS (Time lag = ±3 days) ($℃)$ |
| --- | --- | --- | --- | --- | --- |
| Gamma-2p/LMOM | alpha beta  239.555126 0.137166 | alpha beta  3.51847 11.43545 | alpha beta  7.819270 6.104828 | alpha beta  15.088306 3.385949 | alpha beta  25.886486 2.064443 |
| GEV-3P/LMOM | xi alpha k  32.0140600 1.9858920 0.1777739 | xi alpha k  36.5880624 23.2295233 0.6428916 | xi alpha k  45.2645980 18.9969758 0.7010037 | xi alpha k  47.7453030 14.0984874 0.4817316 | xi alpha k  49.6713768 10.2934064 0.2622138 |
| Normal/LMOM | mu sigma  32.858824 2.121889 | mu sigma  40.23529 20.70390 | mu sigma  47.73529 16.80040 | mu sigma  51.08824 13.04381 | mu sigma  53.44118 10.45305 |
| Normal-2P/MLE | mean 32.858824  sd 2.032112 | location 42.77719  scale 11.64862 | mean 47.73529 sd 18.05272 | mean 51.08824 sd 13.77416 | mean 53.44118 sd 10.25611 |
| Logistic-2P/MLE | location 32.797690 scale 1.202378 | mean 40.23529  sd 21.23823 | location 49.813529  scale 9.079661 | location 51.827485 scale 7.098693 | location 53.399093  scale 5.857564 |
| GEV/MLE | location scale shape  32.0738713 1.9004782 -0.2081928 | location scale shape  -2767.614 30217.972 1958.478 | location scale shape  -782.8801 12220.6096 613.8798 | location scale shape  -92.4421 2914.3724 106.1613 | location scale shape  49.4180158 9.7239792 -0.1938842 |
| Gaussian KDE-Silverman Rule-of-thumb (ROT) Model* | 0.9170161 | 6.220756 | 6.635474 | 4.810718 | 4.147171 |
| Gaussian KDE-Thumb Hand Rule by Scott (1992) Model | 1.080041 | 7.326669 | 7.815113 | 5.665957 | 4.884446 |
| Gaussian KDE-Methods of Sheather & Jones (1991) via Direct Plug-in (DPI) Model | 1.253195 | 5.765939 | 6.308828 | 5.256197 | 4.49265 |
| Gaussian KDE-Methods of Sheather & Jones (1991) via Solve-the-Equation (STE) Model | 1.273555 | 5.054933 | 5.631151 | 4.972076 | 4.252179 |
| Gaussian KDE-Biased-cross validation (BCV) by Scott and Terrell (1987) Model | 1.160308 | 12.13381 | 8.671543 | 7.867321 | 5.856573 |
| Gaussian KDE-Unbiased cross validation (UCV) by Scott and Terrell (1987) Model | 1.161131 | 1.266772 | 1.076769 | 6.954002 | 5.854882 |
| Gaussian KDE-Least Square Cross Validation (LSCV) Model | 1.160006 | 1.056191 | 1.04273 | 6.975771 | 5.828503 |
| Gaussian KDE-Normal Scale (NS) Model | 1.07925 | 11.27957 | 9.587756 | 7.315426 | 5.446995 |
| Gaussian KDE-Smoothed cross-validation (SCV) model | 1.304081 | 7.037465 | 7.045567 | 6.388538 | 4.957346 |

| (e)Fitted 1-D probability distribution (Vancouver) | Annual highest MT (km/hr) | Maximum WGS (Time lag = 0 day) ($℃)$ | Maximum WGS (Time lag = ±1 days) ($℃)$ | Maximum WGS (Time lag = ±2 days) ($℃)$ | Maximum WGS (Time lag = ±3 days) ($℃)$ |
| --- | --- | --- | --- | --- | --- |
| Gamma-2p/LMOM | alpha beta  272.6170459 0.1055921 | alpha beta  0.1357427 44.0742413 | alpha beta  0.9970429 22.7915357 | alpha beta  2.334617 12.539902 | alpha beta  6.178894 5.798381 |
| GEV-3P/LMOM | xi alpha k  28.03516524 1.54278830 0.09962139 | xi alpha k  0.5607632 2.0018735 -0.6869157 | xi alpha k  14.7008864 18.8745962 0.1781791 | xi alpha k  25.8014221 20.2795015 0.6127645 | xi alpha k  34.6337096 16.0895470 0.8264617 |
| Normal/LMOM | mu sigma  28.786207 1.742645 | mu sigma  5.982759 9.010778 | mu sigma  22.72414 20.16180 | mu sigma  29.27586 18.16846 | mu sigma  35.82759 14.12495 |
| Normal-2P/MLE | mean 28.786207  sd 1.771951 | mean 5.982759  sd 13.156212 | mean 22.72414  sd 20.70965 | mean 29.27586  sd 19.09847 | mean 35.82759  sd 15.79517 |
| Logistic-2P/MLE | location 28.6855486  scale 0.9684532 | location 2.481685  scale 5.928125 | location 23.04497 scale 12.99537 | location 31.70852  scale 11.11768 | location 38.388757  scale 7.801551 |
| GEV/MLE | location scale shape  28.0506814 1.5785030 -0.1178315 | location scale shape  2.424414e-12 7.570358e-12 3.078940e+00 | location scale shape  15.7410310 20.5993030 -0.3216528 | location scale shape  -414.6076 3620.0856 330.6826 | location scale shape  -757.1181 9019.8311 611.4624 |
| Gaussian KDE-Silverman Rule-of-thumb (ROT) Model | 0.633594 | 5.3023 | 8.346534 | 7.697187 | 3.20524 |
| Gaussian KDE-Thumb Hand Rule by Scott (1992) Model | 0.7462329 | 0 | 9.830362 | 9.065575 | 3.775061 |
| Gaussian KDE-Methods of Sheather & Jones (1991) via Direct Plug-in (DPI) Model | 0.7417604 | NA | 4.018702 | 4.032095 | 2.956675 |
| Gaussian KDE-Methods of Sheather & Jones (1991) via Solve-the-Equation (STE) Model | 0.7411758 | NA | 1.770976 | 2.612447 | 2.497189 |
| Gaussian KDE-Biased-cross validation (BCV) by Scott and Terrell (1987) Model | 0.9038048 | 6.705774 | 10.55037 | 6.284847 | 4.788312 |
| Gaussian KDE-Unbiased cross validation (UCV) by Scott and Terrell (1987) Model | 0.0943922 | 0.7008342 | 1.103207 | 1.017379 | 0.8414123 |
| Gaussian KDE-Least Square Cross Validation (LSCV) Model | 0.1065675 | 1.042177 | 1.044797 | 1.038086 | 1.05766 |
| Gaussian KDE-Normal Scale (NS) Model | 0.8404858 | 6.240359 | 9.823164 | 9.058937 | 7.492092 |
| Gaussian KDE-Smoothed cross-validation (SCV) model | 0.800316 | 1.743063 | 3.793809 | 3.882871 | 4.015752 |

| (f1) Fitted 1-D probability distribution (Regina) | Annual maximum WGS (km/hr) | Highest MT (Time lag = 0 day) ($℃)$ | Highest MT (Time lag = ±1 days) ($℃)$ | Highest MT (Time lag = ±2 days) ($℃)$ | Highest MT (Time lag = ±3 days) ($℃)$ |
| --- | --- | --- | --- | --- | --- |
| Gamma-2p/LMOM | alpha beta  32.822059 3.214673 | alpha beta  17.355920 1.439588 | alpha beta  30.2140259 0.9083166 | alpha beta  53.8992019 0.5404849 | alpha beta  57.1533388 0.5169235 |
| GEV-3P/LMOM | xi alpha k  97.11329875 15.36769786 0.03193009 | xi alpha k  24.2605168 6.7631027 0.7516547 | xi alpha k  26.5992273 5.5894428 0.6554358 | xi alpha k  28.2175547 4.3416149 0.5331169 | xi alpha k  28.489771 4.175908 0.453054 |
| Normal/LMOM | mu sigma  105.51220 18.34706 | mu sigma  24.985366 5.954364 | mu sigma  27.443902 4.972165 | mu sigma  29.131707 3.958841 | mu sigma  29.543902 3.899398 |
| Normal-2P/MLE | mean 105.51220  sd 18.06104 | mean 24.985366 sd 6.032819 | mean 27.443902  sd 4.984466 | mean 29.131707  sd 3.993253 | mean 29.543902  sd 3.946721 |
| Logistic-2P/MLE | location 104.09842  scale 10.33459 | location 25.725820 scale 3.352686 | location 27.937532  scale 2.777825 | location 29.407499  scale 2.197279 | location 29.734372  scale 2.173777 |
| GEV/MLE | location scale shape  97.32897925 14.74679057 -0.03071391 | location scale shape  17.73656 739.94158 83.52853 | location scale shape  -52.97727 829.31679 134.08539 | location scale shape  28.278930 4.370334 -0.558040 | location scale shape  28.5787973 4.2837901 -0.4990323 |
| Gaussian KDE-Silverman Rule-of-thumb (ROT) Model | 7.669944 | 2.364899 | 2.077276 | 1.470073 | 1.597905 |
| Gaussian KDE-Thumb Hand Rule by Scott (1992) Model | 9.033489 | 2.785326 | 2.44657 | 1.731419 | 1.881977 |
| Gaussian KDE-Methods of Sheather & Jones (1991) via Direct Plug-in (DPI) Model | 9.009623 | 2.079131 | 2.221937 | 1.512147 | 1.509538 |
| Gaussian KDE-Methods of Sheather & Jones (1991) via Solve-the-Equation (STE) Model | 9.009425 | 2.241751 | 2.177609 | 1.297293 | 1.190011 |
| Gaussian KDE-Biased-cross validation (BCV) by Scott and Terrell (1987) Model | 9.905029 | 3.226377 | 2.737113 | 2.191918 | 2.166376 |
| Gaussian KDE-Unbiased cross validation (UCV) by Scott and Terrell (1987) Model | 9.917172 | 1.922535 | 2.674007 | 1.481827 | 1.16293 |
| Gaussian KDE-Least Square Cross Validation (LSCV) Model | 9.91672 | 1.924938 | 2.674933 | 1.454664 | 1.165478 |
| Gaussian KDE-Normal Scale (NS) Model | 9.215854 | 3.078316 | 2.543382 | 2.037604 | 2.01386 |
| Gaussian KDE-Smoothed cross-validation (SCV) model | 9.375529 | 2.370865 | 2.32482 | 1.783155 | 1.646875 |

| (f2) Fitted 1-D probability distribution (Regina) | AHMT ($℃)$ | Maximum WGS (Time lag = 0 day) (km/hr) | Maximum WGS (Time lag = ±1 days) ) (km/hr) | Maximum WGS (Time lag = ±2 days) (km/hr) | Maximum WGS (Time lag = ±3 days) () (km/hr) |
| --- | --- | --- | --- | --- | --- |
| Gamma-2p/LMOM | alpha beta  427.7967314 0.0840894 | alpha beta  3.564254 12.967513 | alpha beta  13.568527 4.644896 | alpha beta  21.379719 3.150923 | alpha beta  21.260175 3.315486 |
| GEV-3P/LMOM | xi alpha k  35.5505927 1.8942808 0.5086204 | xi alpha k  40.7670326 25.9301207 0.5336698 | xi alpha k  55.49194887 14.61963525 0.06645241 | xi alpha k  61.1664063 12.8943364 0.1068805 | xi alpha k  64.4367721 14.2228778 0.1776913 |
| Normal/LMOM | mu sigma  35.973171 1.738734 | mu sigma  46.21951 23.64064 | mu sigma  63.02439 16.95287 | mu sigma  67.36585 14.48441 | mu sigma  70.48780 15.19771 |
| Normal-2P/MLE | mean 35.973171  sd 1.749986 | mean 46.21951  sd 24.55607 | mean 63.02439  sd 16.59928 | mean 67.36585 sd 14.30121 | mean 70.48780  sd 14.97335 |
| Logistic-2P/MLE | location 36.0726028  scale 0.9737473 | location 48.55108 scale 13.29913 | location 61.926497 scale 9.613457 | location 66.568656 scale 8.109956 | location 70.008335 scale 8.524193 |
| GEV/MLE | location scale shape  -486.8266 2273.8213 1518.0633 | location scale shape  -1996.968 23961.777 1328.785 | location scale shape  55.72845090 14.06168030 -0.07203714 | location scale shape  61.477939 12.858052 -0.138001 | location scale shape  64.3462065 13.6892132 -0.1456373 |
| Gaussian KDE-Silverman Rule-of-thumb (ROT) Model | 0.6711201 | 5.752458 | 7.196757 | 5.113296 | 6.491821 |
| Gaussian KDE-Thumb Hand Rule by Scott (1992) Model | 0.7904303 | 6.775117 | 8.47618 | 6.022326 | 7.645922 |
| Gaussian KDE-Methods of Sheather & Jones (1991) via Direct Plug-in (DPI) Model | 0.6457021 | 4.500569 | 8.485004 | 6.546729 | 8.313045 |
| Gaussian KDE-Methods of Sheather & Jones (1991) via Solve-the-Equation (STE) Model | 0.5530278 | 3.568276 | 8.455039 | 6.671597 | 8.410249 |
| Gaussian KDE-Biased-cross validation (BCV) by Scott and Terrell (1987) Model | 0.9611708 | 13.45714 | 9.099371 | 7.848402 | 8.213473 |
| Gaussian KDE-Unbiased cross validation (UCV) by Scott and Terrell (1987) Model | 0.4227898 | 2.631589 | 9.102503 | 7.850011 | 8.218948 |
| Gaussian KDE-Least Square Cross Validation (LSCV) Model | 0.4301787 | 2.629122 | 9.109154 | 7.825462 | 8.192864 |
| Gaussian KDE-Normal Scale (NS) Model | 0.8929507 | 12.53002 | 8.469974 | 7.29736 | 7.640324 |
| Gaussian KDE-Smoothed cross-validation (SCV) model | 0.7205828 | 6.516168 | 8.816884 | 7.554924 | 8.586653 |

| (g)Fitted 1-D probability distribution (Edmonton) | Annual maximum WGS (km/hr) | Highest MT (Time lag = 0 day) ($℃)$ | Highest MT (Time lag = ±1 days) ($℃)$ |
| --- | --- | --- | --- |
| Gamma-2p/LMOM | alpha beta  35.697517 2.521941 | alpha beta  20.000176 1.200935 | alpha beta  43.9176726 0.5974939 |
| GEV-3P/LMOM | xi alpha k  84.3446363 14.4541052 0.2226339 | xi alpha k  23.0165004 5.9649096 0.6196073 | xi alpha k  25.3922648 4.3643054 0.5654587 |
| Normal/LMOM | mu sigma  90.02703 15.01529 | mu sigma  24.018919 5.337322 | mu sigma  26.240541 3.948367 |
| Normal-2P/MLE | mean 90.02703 sd 14.36679 | mean 24.018919  sd 5.251143 | mean 26.240541 sd 3.859874 |
| Logistic-2P/MLE | location 89.733493  scale 8.589582 | location 24.509345  scale 3.025218 | location 26.530884 scale 2.229452 |
| GEV/MLE | location scale shape  85.0012475 14.0681658 -0.2887086 | location scale shape  18.52232 398.19522 69.58633 | location scale shape  25.3427309 4.1840831 -0.5250693 |
| Gaussian KDE-Silverman Rule-of-thumb (ROT) Model | **6.366658** | **2.120365** | **1.710507** |
| Gaussian KDE-Thumb Hand Rule by Scott (1992) Model | 7.498509 | 2.497319 | 2.014597 |
| Gaussian KDE-Methods of Sheather & Jones (1991) via Direct Plug-in (DPI) Model | 8.402273 | 2.208609 | 2.006177 |
| Gaussian KDE-Methods of Sheather & Jones (1991) via Solve-the-Equation (STE) Model | 8.520569 | 2.137409 | 2.004119 |
| Gaussian KDE-Biased-cross validation (BCV) by Scott and Terrell (1987) Model | 8.045186 | 2.945379 | 2.161359 |
| Gaussian KDE-Unbiased cross validation (UCV) by Scott and Terrell (1987) Model | 8.045757 | 2.702473 | 2.165582 |
| Gaussian KDE-Least Square Cross Validation (LSCV) Model | 8.035661 | 2.696633 | 2.167803 |
| Gaussian KDE-Normal Scale (NS) Model | 7.493018 | 2.738741 | 2.013122 |
| Gaussian KDE-Smoothed cross-validation (SCV) model | 8.817796 | 2.354508 | 2.078675 |

Supplementary ST 4: Summarizing most justifiable marginal probability density selected for station (a) Montreal (b) Quebec City (c) Ottawa (d) Toronto (e) Vancouver (f1 & f2) Regina (g) Edmonton

| 1. Montreal | Best fitted univariate marginal pdf |
| --- | --- |
| Annual maximum WGS (km/hr) | Gaussian KDE-Silverman Rule-of-thumb (ROT) |
| Corresponding highest MT (Time lag = 0) ($℃$) | Gaussian KDE-Silverman Rule-of-thumb (ROT) |
| Corresponding highest MT (Time lag = ±1) ($℃$) | Gaussian KDE-Silverman Rule-of-thumb (ROT) |
| Corresponding highest MT (Time lag = ±2) ($℃$) | Gaussian KDE-Silverman Rule-of-thumb (ROT) |
| Corresponding highest MT (Time lag = ±3) ($℃$) | Gaussian KDE-Silverman Rule-of-thumb (ROT) |
| Annual highest MT ($℃$) | Gaussian KDE-Silverman Rule-of-thumb (ROT) |
| Corresponding maximum WGS (Time lag = 0) (km/hr) | Gaussian KDE-Least Square Cross Validation (LSCV) |

| 1. Quebec City | Best fitted univariate marginal pdf |
| --- | --- |
| Annual maximum WGS (km/hr) | Gaussian KDE-Least Square Cross Validation (LSCV) Model |
| Corresponding highest MT (Time lag = 0) ($℃$) | Gaussian KDE-Silverman Rule-of-thumb (ROT) Model |
| Corresponding highest MT (Time lag = ±1) ($℃$) | Gaussian KDE-Silverman Rule-of-thumb (ROT) Model |
| Corresponding highest MT (Time lag = ±2) ($℃$) | Gaussian KDE-Silverman Rule-of-thumb (ROT) Model |
| Corresponding highest MT (Time lag = ±3) ($℃$) | Gaussian KDE-Least Square Cross Validation (LSCV) Model |

| 1. Ottawa | Best fitted univariate marginal pdf |
| --- | --- |
| Annual maximum WGS (km/hr) | Gaussian KDE-Silverman Rule-of-thumb (ROT) Model |
| Corresponding highest MT (Time lag = 0) ($℃$) | Gaussian KDE-Silverman Rule-of-thumb (ROT) Model |
| Corresponding highest MT (Time lag = ±1) ($℃$) | Gaussian KDE-Silverman Rule-of-thumb (ROT) Model |
| Corresponding highest MT (Time lag = ±2) ($℃$) | Gaussian KDE-Silverman Rule-of-thumb (ROT) Model |
| Corresponding highest MT (Time lag = ±3) ($℃$) | Gaussian KDE-Silverman Rule-of-thumb (ROT) Model |

| 1. Toronto | Best fitted univariate marginal pdf |
| --- | --- |
| Annual highest MT ($℃$) | Gaussian KDE-Silverman Rule-of-thumb (ROT) Model |
| Corresponding maximum WGS (Time lag = 0day) (km/hr) | Gaussian KDE-Unbiased cross validation (UCV) by Scott and Terrell (1987) Model |
| Corresponding maximum WGS (Time lag = ±1 days) (km/hr) | Gaussian KDE-Unbiased cross validation (UCV) by Scott and Terrell (1987) Model |
| Corresponding maximum WGS (Time lag = ±2 days) (km/hr) | Gaussian KDE-Silverman Rule-of-thumb (ROT) Model |
| Corresponding maximum WGS (Time lag = ±3 days) (km/hr) | Gaussian KDE-Silverman Rule-of-thumb (ROT) Model |

| 1. Vancouver | Best fitted univariate marginal pdf |
| --- | --- |
| Annual highest MT($℃$) | Gaussian KDE-Unbiased cross validation (UCV) by Scott and Terrell (1987) Model |
| Corresponding maximum WGS (Time lag = 0day) (km/hr) | Gaussian KDE-Least Square Cross Validation (LSCV) Model |
| Corresponding maximum WGS (Time lag = ±1 days) (km/hr) | Gaussian KDE-Methods of Sheather & Jones (1991) via Solve-the-Equation (STE) Model |
| Corresponding maximum WGS (Time lag = ±2 days) (km/hr) | Gaussian KDE-Methods of Sheather & Jones (1991) via Solve-the-Equation (STE) Model |
| Corresponding maximum WGS (Time lag = ±3 days) (km/hr) | Gaussian KDE-Methods of Sheather & Jones (1991) via Solve-the-Equation (STE) Model |

| 1. Regina region) | Best fitted univariate marginal pdf |  | Best fitted univariate marginal pdf |
| --- | --- | --- | --- |
| AMWGS (km/hr) | Gaussian KDE-Silverman Rule-of-thumb (ROT) Model | AHMT ($℃$) | Gaussian KDE-Least Square Cross Validation (LSCV) Model |
| Corresponding highest MT (Time lag = 0day) ($℃)$ | Gaussian KDE-Unbiased cross validation (UCV) by Scott and Terrell (1987) Model | Corresponding maximum WGS (Time lag = 0day) (km/hr) | Gaussian KDE-Least Square Cross Validation (LSCV) Model |
| Corresponding highest MT (Time lag = ±1 days) ($℃)$ | Gaussian KDE-Silverman Rule-of-thumb (ROT) Model | Corresponding maximum WGS (Time lag = ±1 days) (km/hr) | Gaussian KDE-Silverman Rule-of-thumb (ROT) Model |
| Corresponding highest MT (Time lag = ±2 days) ($℃)$ | Gaussian KDE-Methods of Sheather & Jones (1991) via Solve-the-Equation (STE) Model | Corresponding maximum WGS (Time lag = ±2 days) (km/hr) | Gaussian KDE-Silverman Rule-of-thumb (ROT) Model |
| Corresponding highest MT (Time lag = ±3 days) ($℃)$ | Gaussian KDE-Unbiased cross validation (UCV) by Scott and Terrell (1987) Model | Corresponding maximum WGS (Time lag = ±3 days) (km/hr) | Gaussian KDE-Silverman Rule-of-thumb (ROT) Model |

| 1. Edmonton | Best fitted univariate marginal pdf |
| --- | --- |
| Annual maximum WGS (km/hr) | Gaussian KDE-Silverman Rule-of-thumb (ROT) Model |
| Highest MT (Time lag = 0 day) (℃) | Gaussian KDE-Silverman Rule-of-thumb (ROT) Model |
| Highest MT (Time lag = ±1 days) (℃) | Gaussian KDE-Silverman Rule-of-thumb (ROT) Model |

Supplementary Table ST 5: Performance evaluation of fitted 1-D parametric and nonparametric models for station (a) Montreal (b) Quebec City (c) Ottawa (d) Toronto (e) Vancouver (f) Regina (g) Edmonton

| (a-1) Annual maximum WGS ($℃)$  (Montreal) | Mean Square Error (MSE) | Akaike Information Criterion (AIC) | Bayesian Information Criterion (BIC) | Hannan-Quinn Information Criterion (HQC) |
| --- | --- | --- | --- | --- |
| Gamma-2P/LMOM | 0.000996395 | -272.455 | -269.077 | -271.233 |
| GEV-3P/LMOM | 0.0005000216 | -298.034 | -292.968 | -296.202 |
| Normal-2P/LMOM | 0.001846977 | -247.768 | -244.39 | -246.547 |
| NORMAL-2P/MLE | 0.001847752 | -247.751 | -244.374 | -246.53 |
| LOGISTIC-2P/MLE | 0.001109072 | -268.169 | -264.792 | -266.948 |
| GEV-3P/MLE | 0.0004923748 | -298.651 | -293.584 | -296.819 |
| Gaussian KDE-Silverman Rule-of-thumb (ROT) Model | 0.0003410506 | -317.339 | -315.65 | -316.729 |
| Gaussian KDE-Thumb Hand Rule by Scott (1992) Model | 0.000379994 | -313.014 | -311.325 | -312.404 |
| Gaussian KDE-Methods of Sheather & Jones (1991) via Direct Plug-in (DPI) Model | 0.0003942508 | -311.541 | -309.852 | -310.93 |
| Gaussian KDE-Methods of Sheather & Jones (1991) via Solve-the-Equation (STE) Model | 0.0003956625 | -311.398 | -309.709 | -310.787 |
| Gaussian KDE-Biased-cross validation (BCV) by Scott and Terrell (1987) Model | 0.0004362031 | -307.496 | -305.807 | -306.885 |
| Gaussian KDE-Unbiased cross validation (UCV) by Scott and Terrell (1987) Model | 0.000435779 | -307.535 | -305.846 | -306.924 |
| Gaussian KDE-Least Square Cross Validation (LSCV) Model | 0.0004351233 | -307.595 | -305.906 | -306.985 |
| Gaussian KDE-Normal Scale (NS) Model | 0.0004165213 | -309.343 | -307.654 | -308.732 |
| Gaussian KDE-Smoothed cross-validation (SCV) model | 0.0004163736 | -309.357 | -307.668 | -308.746 |

| (a-2)MT (Time lag = 0 day)  (Montreal) | Mean Square Error (MSE) | Akaike Information Criterion (AIC) | Bayesian Information Criterion (BIC) | Hannan-Quinn Information Criterion (HQC) |
| --- | --- | --- | --- | --- |
| Gamma-2P/LMOM | 0.002600443 | -234.083 | -230.705 | -232.862 |
| GEV-3P/LMOM | 0.0008970005 | -274.658 | -269.592 | -272.826 |
| Normal-2P/LMOM | 0.001828529 | -248.17 | -244.792 | -246.948 |
| NORMAL-2P/MLE | 0.001918078 | -246.257 | -242.88 | -245.036 |
| LOGISTIC-2P/MLE | 0.001767874 | -249.519 | -246.141 | -248.298 |
| GEV-3P/MLE | 0.0009957284 | -270.481 | -265.415 | -268.65 |
| Gaussian KDE-Silverman Rule-of-thumb (ROT) Model | 0.0006631434 | -290.741 | -289.052 | -290.13 |
| Gaussian KDE-Thumb Hand Rule by Scott (1992) Model | 0.000741637 | -286.266 | -284.577 | -285.655 |
| Gaussian KDE-Methods of Sheather & Jones (1991) via Direct Plug-in (DPI) Model | 0.0007222528 | -287.325 | -285.637 | -286.715 |
| Gaussian KDE-Methods of Sheather & Jones (1991) via Solve-the-Equation (STE) Model | 0.0007085073 | -288.094 | -286.405 | -287.483 |
| Gaussian KDE-Biased-cross validation (BCV) by Scott and Terrell (1987) Model | 0.000780183 | -284.239 | -282.55 | -283.629 |
| Gaussian KDE-Unbiased cross validation (UCV) by Scott and Terrell (1987) Model | 0.0007808849 | -284.203 | -282.514 | -283.593 |
| Gaussian KDE-Least Square Cross Validation (LSCV) Model | 0.0007823012 | -284.131 | -282.442 | -283.52 |
| Gaussian KDE-Normal Scale (NS) Model | 0.0007412567 | -286.287 | -284.598 | -285.676 |
| Gaussian KDE-Smoothed cross-validation (SCV) model | 0.0007400227 | -286.353 | -284.664 | -285.743 |

| (a-3) MT (Time lag = ±1 day)  (Montreal) | Mean Square Error (MSE) | Akaike Information Criterion (AIC) | Bayesian Information Criterion (BIC) | Hannan-Quinn Information Criterion (HQC) |
| --- | --- | --- | --- | --- |
| Gamma-2P/LMOM | 0.003995145 | -216.907 | -213.529 | -215.686 |
| GEV-3P/LMOM | 0.0006614525 | -286.843 | -281.776 | -285.011 |
| Normal-2P/LMOM | 0.002890585 | -229.852 | -226.474 | -228.631 |
| NORMAL-2P/MLE | 0.002883439 | -229.951 | -226.573 | -228.73 |
| LOGISTIC-2P/MLE | 0.001679275 | -251.576 | -248.198 | -250.354 |
| GEV-3P/MLE | 0.001327249 | -258.986 | -253.919 | -257.154 |
| Gaussian KDE-Silverman Rule-of-thumb (ROT) Model* | 0.0007178865 | -287.568 | -285.879 | -286.957 |
| Gaussian KDE-Thumb Hand Rule by Scott (1992) Model | 0.0007591431 | -285.333 | -283.644 | -284.722 |
| Gaussian KDE-Methods of Sheather & Jones (1991) via Direct Plug-in (DPI) Model | 0.0007502021 | -285.807 | -284.118 | -285.196 |
| Gaussian KDE-Methods of Sheather & Jones (1991) via Solve-the-Equation (STE) Model | 0.0007485573 | -285.895 | -284.206 | -285.284 |
| Gaussian KDE-Biased-cross validation (BCV) by Scott and Terrell (1987) Model | 0.000797405 | -283.366 | -281.677 | -282.755 |
| Gaussian KDE-Unbiased cross validation (UCV) by Scott and Terrell (1987) Model | 0.0007982222 | -283.325 | -281.636 | -282.714 |
| Gaussian KDE-Least Square Cross Validation (LSCV) Model | 0.000798577 | -283.307 | -281.618 | -282.697 |
| Gaussian KDE-Normal Scale (NS) Model | 0.0007759513 | -284.457 | -282.768 | -283.846 |
| Gaussian KDE-Smoothed cross-validation (SCV) model | 0.0007612782 | -285.22 | -283.532 | -284.61 |

| (a-4)MT (Time lag = ±2 day)  (Montreal) | Mean Square Error (MSE) | Akaike Information Criterion (AIC) | Bayesian Information Criterion (BIC) | Hannan-Quinn Information Criterion (HQC) |
| --- | --- | --- | --- | --- |
| Gamma-2P/LMOM | 0.003989715 | -216.961 | -213.584 | -215.74 |
| GEV-3P/LMOM | 0.0007556505 | -281.517 | -276.451 | -279.685 |
| Normal-2P/LMOM | 0.002942504 | -229.14 | -225.762 | -227.918 |
| NORMAL-2P/MLE | 0.002938636 | -229.192 | -225.815 | -227.971 |
| LOGISTIC-2P/MLE | 0.001605639 | -253.369 | -249.992 | -252.148 |
| GEV-3P/MLE | 0.0009632692 | -271.807 | -266.74 | -269.975 |
| Gaussian KDE-Silverman Rule-of-thumb (ROT) Model* | 0.0007007627 | -288.534 | -286.845 | -287.923 |
| Gaussian KDE-Thumb Hand Rule by Scott (1992) Model | 0.0007342238 | -286.668 | -284.979 | -286.057 |
| Gaussian KDE-Methods of Sheather & Jones (1991) via Direct Plug-in (DPI) Model | 0.0007353328 | -286.607 | -284.919 | -285.997 |
| Gaussian KDE-Methods of Sheather & Jones (1991) via Solve-the-Equation (STE) Model | 0.0007352669 | -286.611 | -284.922 | -286 |
| Gaussian KDE-Biased-cross validation (BCV) by Scott and Terrell (1987) Model | 0.0007989293 | -283.29 | -281.601 | -282.679 |
| Gaussian KDE-Unbiased cross validation (UCV) by Scott and Terrell (1987) Model | 0.000798996 | -283.286 | -281.597 | -282.676 |
| Gaussian KDE-Least Square Cross Validation (LSCV) Model | 0.0007993639 | -283.268 | -281.579 | -282.657 |
| Gaussian KDE-Normal Scale (NS) Model | 0.0007737935 | -284.568 | -282.879 | -283.958 |
| Gaussian KDE-Smoothed cross-validation (SCV) model | 0.0007561069 | -285.493 | -283.804 | -284.882 |

| (a-5) MT (Time lag = 3 day)  (Montreal) | Mean Square Error (MSE) | Akaike Information Criterion (AIC) | Bayesian Information Criterion (BIC) | Hannan-Quinn Information Criterion (HQC) |
| --- | --- | --- | --- | --- |
| Gamma-2P/LMOM | 0.001843327 | -247.847 | -244.47 | -246.626 |
| GEV-3P/LMOM | 0.0007623217 | -281.166 | -276.099 | -279.334 |
| Normal-2P/LMOM | 0.001374921 | -259.574 | -256.197 | -258.353 |
| NORMAL-2P/MLE | 0.00141137 | -258.528 | -255.15 | -257.306 |
| LOGISTIC-2P/MLE | 0.001177514 | -265.774 | -262.396 | -264.553 |
| GEV-3P/MLE | 0.001116661 | -265.896 | -260.83 | -264.065 |
| Gaussian KDE-Silverman Rule-of-thumb (ROT) Model | 0.0007762683 | -284.44 | -282.752 | -283.83 |
| Gaussian KDE-Thumb Hand Rule by Scott (1992) Model | 0.0008410335 | -281.235 | -279.546 | -280.625 |
| Gaussian KDE-Methods of Sheather & Jones (1991) via Direct Plug-in (DPI) Model | 0.0008629512 | -280.206 | -278.517 | -279.595 |
| Gaussian KDE-Methods of Sheather & Jones (1991) via Solve-the-Equation (STE) Model | 0.0008663874 | -280.047 | -278.358 | -279.436 |
| Gaussian KDE-Biased-cross validation (BCV) by Scott and Terrell (1987) Model | 0.0008648516 | -280.118 | -278.429 | -279.507 |
| Gaussian KDE-Unbiased cross validation (UCV) by Scott and Terrell (1987) Model | 0.00086514 | -280.105 | -278.416 | -279.494 |
| Gaussian KDE-Least Square Cross Validation (LSCV) Model | 0.0008655703 | -280.085 | -278.396 | -279.474 |
| Gaussian KDE-Normal Scale (NS) Model | 0.000840772 | -281.248 | -279.559 | -280.637 |
| Gaussian KDE-Smoothed cross-validation (SCV) model | 0.0008738836 | -279.703 | -278.014 | -279.092 |

| (a-6) AHMT Montreal | Mean Square Error (MSE) | Akaike Information Criterion (AIC) | Bayesian Information Criterion (BIC) | Hannan-Quinn Information Criterion (HQC) |
| --- | --- | --- | --- | --- |
| Gamma-2p/LMOM | 0.001398627 | -258.891 | -255.513 | -257.669 |
| GEV-3P/LMOM | 0.001132898 | -265.319 | -260.252 | -263.487 |
| Normal-2P/LMOM | 0.00154412 | -254.932 | -251.554 | -253.711 |
| Weinull-3P/LMOM | 0.001339067 | -258.631 | -253.565 | -256.799 |
| NORMAL-2P/MLE | 0.00161337 | -253.177 | -249.799 | -251.956 |
| LOGISTIC-2P/MLE | 0.001031432 | -271.072 | -267.695 | -269.851 |
| GEV-3P/MLE | 0.002146739 | -239.752 | -234.686 | -237.92 |
| Gaussian KDE-Silverman Rule-of-thumb (ROT) Model* | 0.0008232041 | -282.092 | -280.403 | -281.482 |
| Gaussian KDE-Thumb Hand Rule by Scott (1992) Model | 0.0008528227 | -280.678 | -278.989 | -280.068 |
| Gaussian KDE-Methods of Sheather & Jones (1991) via Direct Plug-in (DPI) Model | 0.0008604041 | -280.324 | -278.635 | -279.714 |
| Gaussian KDE-Methods of Sheather & Jones (1991) via Solve-the-Equation (STE) Model | 0.0008619787 | -280.251 | -278.562 | -279.641 |
| Gaussian KDE-Biased-cross validation (BCV) by Scott and Terrell (1987) Model | 0.0008859639 | -279.153 | -277.464 | -278.543 |
| Gaussian KDE-Unbiased cross validation (UCV) by Scott and Terrell (1987) Model | 0.0008855779 | -279.171 | -277.482 | -278.56 |
| Gaussian KDE-Least Square Cross Validation (LSCV) Model | 0.0008851864 | -279.188 | -277.5 | -278.578 |
| Gaussian KDE-Normal Scale (NS) Model | 0.0008698294 | -279.889 | -278.2 | -279.278 |
| Gaussian KDE-Smoothed cross-validation (SCV) model | 0.0008729894 | -279.743 | -278.055 | -279.133 |

| (a-7) WGS (Time interval = 0) Montreal | Mean Square Error (MSE) | Akaike Information Criterion (AIC) | Bayesian Information Criterion (BIC) | Hannan-Quinn Information Criterion (HQC) |
| --- | --- | --- | --- | --- |
| Gamma-2p/LMOM | 0.00761529 | -191.104 | -187.726 | -189.883 |
| GEV-3P/LMOM | 0.003582991 | -219.262 | -214.196 | -217.43 |
| Normal-2P/LMOM | 0.003390214 | -223.474 | -220.097 | -222.253 |
| Weinull-3P/LMOM | 0.003475639 | -220.479 | -215.412 | -218.647 |
| NORMAL-2P/MLE | 0.003480378 | -222.425 | -219.047 | -221.203 |
| LOGISTIC-2P/MLE | 0.002430611 | -236.785 | -233.407 | -235.563 |
| GEV-3P/MLE | 0.01031964 | -176.948 | -171.882 | -175.116 |
| Gaussian KDE-Silverman Rule-of-thumb (ROT) Model* | 0.001014914 | -273.718 | -272.029 | -273.107 |
| Gaussian KDE-Thumb Hand Rule by Scott (1992) Model | 0.001106862 | -270.249 | -268.56 | -269.638 |
| Gaussian KDE-Methods of Sheather & Jones (1991) via Direct Plug-in (DPI) Model | 0.0008675104 | -279.995 | -278.306 | -279.385 |
| Gaussian KDE-Methods of Sheather & Jones (1991) via Solve-the-Equation (STE) Model | 0.0005752172 | -296.431 | -294.742 | -295.82 |
| Gaussian KDE-Biased-cross validation (BCV) by Scott and Terrell (1987) Model | 0.001655126 | -254.155 | -252.466 | -253.544 |
| Gaussian KDE-Unbiased cross validation (UCV) by Scott and Terrell (1987) Model | 0.04886876 | -118.745 | -117.056 | -118.134 |
| Gaussian KDE-Least Square Cross Validation (LSCV) Model | 0.0004072259 | -310.246 | -308.557 | -309.635 |
| Gaussian KDE-Normal Scale (NS) Model | 0.001573384 | -256.181 | -254.492 | -255.57 |
| Gaussian KDE-Smoothed cross-validation (SCV) model | 0.001096822 | -270.614 | -268.925 | -270.003 |

| (b1) AMWGS ($℃)$  (QUEBEC CITY) | Mean Square Error (MSE) | Akaike Information Criterion (AIC) | Bayesian Information Criterion (BIC) | Hannan-Quinn Information Criterion (HQC) |
| --- | --- | --- | --- | --- |
| Gamma-2P/LMOM | 0.004769243 | -172.404 | -169.411 | -171.397 |
| GEV-3P/LMOM | 0.001370802 | -211.548 | -207.058 | -210.037 |
| Normal-2P/LMOM | 0.007525321 | -157.353 | -154.36 | -156.346 |
| NORMAL-2P/MLE | 0.00756722 | -157.17 | -154.177 | -156.163 |
| LOGISTIC-2P/MLE | 0.003858349 | -179.398 | -176.405 | -178.391 |
| GEV-3P/MLE | 0.02326228 | -118.11 | -113.621 | -116.6 |
| Gaussian KDE-Silverman Rule-of-thumb (ROT) Model* | 0.001247997 | -218.645 | -217.149 | -218.142 |
| Gaussian KDE-Thumb Hand Rule by Scott (1992) Model | 0.001674121 | -208.951 | -207.455 | -208.448 |
| Gaussian KDE-Methods of Sheather & Jones (1991) via Direct Plug-in (DPI) Model | 0.0009334595 | -228.228 | -226.732 | -227.725 |
| Gaussian KDE-Methods of Sheather & Jones (1991) via Solve-the-Equation (STE) Model | 0.0007857414 | -233.913 | -232.417 | -233.41 |
| Gaussian KDE-Biased-cross validation (BCV) by Scott and Terrell (1987) Model | 0.002269326 | -198.913 | -197.416 | -198.409 |
| Gaussian KDE-Unbiased cross validation (UCV) by Scott and Terrell (1987) Model | 0.0008150665 | -232.704 | -231.207 | -232.2 |
| Gaussian KDE-Least Square Cross Validation (LSCV) Model | 0.0005418739 | -246.176 | -244.679 | -245.672 |
| Gaussian KDE-Normal Scale (NS) Model | 0.00207271 | -201.904 | -200.407 | -201.4 |
| Gaussian KDE-Smoothed cross-validation (SCV) model | 0.001069372 | -223.743 | -222.246 | -223.239 |

| (b-2) MT (Time lag = 0 day) ($℃)$QUEBEC CITY | Mean Square Error (MSE) | Akaike Information Criterion (AIC) | Bayesian Information Criterion (BIC) | Hannan-Quinn Information Criterion (HQC) |
| --- | --- | --- | --- | --- |
| Gamma-2P/LMOM | 0.008183708 | -154.585 | -151.592 | -153.578 |
| GEV-3P/LMOM | 0.0009791226 | -222.652 | -218.163 | -221.142 |
| Normal-2P/LMOM | 0.00561458 | -167.019 | -164.026 | -166.012 |
| NORMAL-2P/MLE | 0.005613846 | -167.023 | -164.03 | -166.016 |
| LOGISTIC-2P/MLE | 0.002516254 | -193.504 | -190.511 | -192.497 |
| GEV-3P/MLE | 0.02294066 | -118.57 | -114.08 | -117.059 |
| Gaussian KDE-Silverman Rule-of-thumb (ROT) Model* | 0.0005503003 | -245.667 | -244.17 | -245.163 |
| Gaussian KDE-Thumb Hand Rule by Scott (1992) Model | 0.0005762067 | -244.148 | -242.652 | -243.645 |
| Gaussian KDE-Methods of Sheather & Jones (1991) via Direct Plug-in (DPI) Model | 0.0005611518 | -245.022 | -243.526 | -244.519 |
| Gaussian KDE-Methods of Sheather & Jones (1991) via Solve-the-Equation (STE) Model | 0.000558809 | -245.16 | -243.664 | -244.657 |
| Gaussian KDE-Biased-cross validation (BCV) by Scott and Terrell (1987) Model | 0.0007009338 | -237.682 | -236.186 | -237.179 |
| Gaussian KDE-Unbiased cross validation (UCV) by Scott and Terrell (1987) Model | 0.0006117333 | -242.174 | -240.678 | -241.671 |
| Gaussian KDE-Least Square Cross Validation (LSCV) Model | 0.0006116937 | -242.176 | -240.68 | -241.673 |
| Gaussian KDE-Normal Scale (NS) Model | 0.000657144 | -239.811 | -238.315 | -239.308 |
| Gaussian KDE-Smoothed cross-validation (SCV) model | 0.0005792892 | -243.972 | -242.476 | -243.469 |

| (b-3) MT (Time lag = ±1 days) ($℃)$  (QUEBEC CITY) | Mean Square Error (MSE) | Akaike Information Criterion (AIC) | Bayesian Information Criterion (BIC) | Hannan-Quinn Information Criterion (HQC) |
| --- | --- | --- | --- | --- |
| Gamma-2P/LMOM | 0.009567129 | -149.431 | -146.438 | -148.424 |
| GEV-3P/LMOM | 0.001520993 | -208.117 | -203.627 | -206.606 |
| Normal-2P/LMOM | 0.007171831 | -158.941 | -155.948 | -157.934 |
| NORMAL-2P/MLE | 0.007097939 | -159.282 | -156.289 | -158.275 |
| LOGISTIC-2P/MLE | 0.004169242 | -176.841 | -173.848 | -175.834 |
| GEV-3P/MLE | 0.02381549 | -117.335 | -112.845 | -115.824 |
| Gaussian KDE-Silverman Rule-of-thumb (ROT) Model | 0.0009727474 | -226.868 | -225.371 | -226.364 |
| Gaussian KDE-Thumb Hand Rule by Scott (1992) Model | 0.001031868 | -224.921 | -223.424 | -224.417 |
| Gaussian KDE-Methods of Sheather & Jones (1991) via Direct Plug-in (DPI) Model | 0.001009743 | -225.636 | -224.139 | -225.132 |
| Gaussian KDE-Methods of Sheather & Jones (1991) via Solve-the-Equation (STE) Model | 0.001000715 | -225.932 | -224.436 | -225.429 |
| Gaussian KDE-Biased-cross validation (BCV) by Scott and Terrell (1987) Model | 0.001285339 | -217.672 | -216.176 | -217.169 |
| Gaussian KDE-Unbiased cross validation (UCV) by Scott and Terrell (1987) Model | 0.001122787 | -222.134 | -220.638 | -221.631 |
| Gaussian KDE-Least Square Cross Validation (LSCV) Model | 0.001123384 | -222.117 | -220.62 | -221.613 |
| Gaussian KDE-Normal Scale (NS) Model | 0.001241494 | -218.818 | -217.321 | -218.314 |
| Gaussian KDE-Smoothed cross-validation (SCV) model | 0.001089042 | -223.141 | -221.645 | -222.638 |

| (b-4) MT (Time lag = ±2 days) ($℃)$  QUEBEC CITY | Mean Square Error (MSE) | Akaike Information Criterion (AIC) | Bayesian Information Criterion (BIC) | Hannan-Quinn Information Criterion (HQC) |
| --- | --- | --- | --- | --- |
| Gamma-2P/LMOM | 0.007808847 | -156.132 | -153.139 | -155.125 |
| GEV-3P/LMOM | 0.001889454 | -200.958 | -196.469 | -199.448 |
| Normal-2P/LMOM | 0.005601384 | -167.096 | -164.103 | -166.089 |
| NORMAL-2P/MLE | 0.005617978 | -166.999 | -164.006 | -165.992 |
| LOGISTIC-2P/MLE | 0.003406652 | -183.507 | -180.514 | -182.5 |
| GEV-3P/MLE | 0.02104785 | -121.412 | -116.922 | -119.901 |
| Gaussian KDE-Silverman Rule-of-thumb (ROT) Model | 0.0006910209 | -238.152 | -236.656 | -237.649 |
| Gaussian KDE-Thumb Hand Rule by Scott (1992) Model | 0.0007740957 | -234.406 | -232.909 | -233.902 |
| Gaussian KDE-Methods of Sheather & Jones (1991) via Direct Plug-in (DPI) Model | 0.0007545656 | -235.249 | -233.753 | -234.746 |
| Gaussian KDE-Methods of Sheather & Jones (1991) via Solve-the-Equation (STE) Model | 0.0007431602 | -235.752 | -234.255 | -235.248 |
| Gaussian KDE-Biased-cross validation (BCV) by Scott and Terrell (1987) Model | 0.0009399795 | -227.999 | -226.502 | -227.495 |
| Gaussian KDE-Unbiased cross validation (UCV) by Scott and Terrell (1987) Model | 0.0008507521 | -231.29 | -229.793 | -230.786 |
| Gaussian KDE-Least Square Cross Validation (LSCV) Model | 0.0008507803 | -231.289 | -229.792 | -230.785 |
| Gaussian KDE-Normal Scale (NS) Model | 0.0009074666 | -229.16 | -227.664 | -228.657 |
| Gaussian KDE-Smoothed cross-validation (SCV) model | 0.0008353582 | -231.892 | -230.396 | -231.389 |

| (b-5)MT (Time lag = ±3 days) ($℃)$  (QUEBEC CITY) | Mean Square Error (MSE) | Akaike Information Criterion (AIC) | Bayesian Information Criterion (BIC) | Hannan-Quinn Information Criterion (HQC) |
| --- | --- | --- | --- | --- |
| Gamma-2P/LMOM | 0.003910242 | -178.957 | -175.964 | -177.95 |
| GEV-3P/LMOM | 0.001888553 | -200.974 | -196.485 | -199.464 |
| Normal-2P/LMOM | 0.002981587 | -187.905 | -184.912 | -186.898 |
| NORMAL-2P/MLE | 0.002994353 | -187.764 | -184.771 | -186.757 |
| LOGISTIC-2P/MLE | 0.00220708 | -197.831 | -194.838 | -196.824 |
| GEV-3P/MLE | 0.01461905 | -133.439 | -128.95 | -131.929 |
| Gaussian KDE-Silverman Rule-of-thumb (ROT) Model | 0.000898232 | -229.498 | -228.001 | -228.994 |
| Gaussian KDE-Thumb Hand Rule by Scott (1992) Model | 0.001044517 | -224.519 | -223.022 | -224.015 |
| Gaussian KDE-Methods of Sheather & Jones (1991) via Direct Plug-in (DPI) Model | 0.0009643885 | -227.153 | -225.656 | -226.649 |
| Gaussian KDE-Methods of Sheather & Jones (1991) via Solve-the-Equation (STE) Model | 0.0007742625 | -234.399 | -232.902 | -233.895 |
| Gaussian KDE-Biased-cross validation (BCV) by Scott and Terrell (1987) Model | 0.001284672 | -217.689 | -216.193 | -217.186 |
| Gaussian KDE-Unbiased cross validation (UCV) by Scott and Terrell (1987) Model | 0.0006577978 | -239.778 | -238.282 | -239.275 |
| Gaussian KDE-Least Square Cross Validation (LSCV) Model | 0.0003848849 | -257.465 | -255.968 | -256.961 |
| Gaussian KDE-Normal Scale (NS) Model | 0.001230922 | -219.1 | -217.603 | -218.596 |
| Gaussian KDE-Smoothed cross-validation (SCV) model | 0.001174914 | -220.636 | -219.14 | -220.133 |

| (c-1)AMWGS (km/hr)  Ottawa | Mean Square Error (MSE) | Akaike Information Criterion (AIC) | Bayesian Information Criterion (BIC) | Hannan-Quinn Information Criterion (HQC) |
| --- | --- | --- | --- | --- |
| Gamma-2P/LMOM | 0.001796821 | -356.339 | -352.253 | -354.751 |
| GEV-3P/LMOM | 0.0008269454 | -398.573 | -392.444 | -396.191 |
| Normal-2P/LMOM | 0.002921716 | -328.628 | -324.542 | -327.04 |
| NORMAL-2P/MLE | 0.003577015 | -317.094 | -313.008 | -315.506 |
| LOGISTIC-2P/MLE | 0.0007445574 | -406.555 | -402.469 | -404.967 |
| GEV-3P/MLE | 0.0006834666 | -409.435 | -403.306 | -407.053 |
| Gaussian KDE-Silverman Rule-of-thumb (ROT) Model | 0.0003290388 | -455.102 | -453.059 | -454.308 |
| Gaussian KDE-Thumb Hand Rule by Scott (1992) Model | 0.0003561864 | -450.583 | -448.54 | -449.789 |
| Gaussian KDE-Methods of Sheather & Jones (1991) via Direct Plug-in (DPI) Model | 0.0003688469 | -448.592 | -446.549 | -447.798 |
| Gaussian KDE-Methods of Sheather & Jones (1991) via Solve-the-Equation (STE) Model | 0.0003705447 | -448.331 | -446.288 | -447.537 |
| Gaussian KDE-Biased-cross validation (BCV) by Scott and Terrell (1987) Model | 0.0004211549 | -441.033 | -438.99 | -440.239 |
| Gaussian KDE-Unbiased cross validation (UCV) by Scott and Terrell (1987) Model | 0.000463588 | -435.561 | -433.518 | -434.767 |
| Gaussian KDE-Least Square Cross Validation (LSCV) Model | 0.0004642693 | -435.478 | -433.435 | -434.684 |
| Gaussian KDE-Normal Scale (NS) Model | 0.0004656221 | -435.312 | -433.269 | -434.518 |
| Gaussian KDE-Smoothed cross-validation (SCV) model | 0.0003953202 | -444.641 | -442.598 | -443.847 |

| (c-2)MT (Time lag = 0 day) ($℃)$  Ottawa | Mean Square Error (MSE) | Akaike Information Criterion (AIC) | Bayesian Information Criterion (BIC) | Hannan-Quinn Information Criterion (HQC) |
| --- | --- | --- | --- | --- |
| Gamma-2P/LMOM | 0.002116936 | -346.994 | -342.908 | -345.406 |
| GEV-3P/LMOM | 0.0005265448 | -424.303 | -418.174 | -421.921 |
| Normal-2P/LMOM | 0.001205818 | -379.074 | -374.988 | -377.486 |
| NORMAL-2P/MLE | 0.001205156 | -379.105 | -375.019 | -377.517 |
| LOGISTIC-2P/MLE | 0.0007939031 | -402.897 | -398.811 | -401.309 |
| GEV-3P/MLE | 0.332958 | -56.6851 | -50.556 | -54.3031 |
| Gaussian KDE-Silverman Rule-of-thumb (ROT) Model | 0.0002507622 | -470.587 | -468.544 | -469.793 |
| Gaussian KDE-Thumb Hand Rule by Scott (1992) Model | 0.0002898806 | -462.324 | -460.281 | -461.53 |
| Gaussian KDE-Methods of Sheather & Jones (1991) via Direct Plug-in (DPI) Model | 0.0002996858 | -460.428 | -458.385 | -459.634 |
| Gaussian KDE-Methods of Sheather & Jones (1991) via Solve-the-Equation (STE) Model | 0.0003005626 | -460.262 | -458.219 | -459.468 |
| Gaussian KDE-Biased-cross validation (BCV) by Scott and Terrell (1987) Model | 0.0003354334 | -454.005 | -451.962 | -453.211 |
| Gaussian KDE-Unbiased cross validation (UCV) by Scott and Terrell (1987) Model | 0.0003352721 | -454.032 | -451.989 | -453.238 |
| Gaussian KDE-Least Square Cross Validation (LSCV) Model | 0.0003359443 | -453.918 | -451.875 | -453.124 |
| Gaussian KDE-Normal Scale (NS) Model | 0.0003150905 | -457.571 | -455.528 | -456.777 |
| Gaussian KDE-Smoothed cross-validation (SCV) model | 0.0003195384 | -456.772 | -454.729 | -455.978 |

| (c-3) MT (Time lag = ±1 day) ($℃)$  Ottawa | Mean Square Error (MSE) | Akaike Information Criterion (AIC) | Bayesian Information Criterion (BIC) | Hannan-Quinn Information Criterion (HQC) |
| --- | --- | --- | --- | --- |
| Gamma-2P/LMOM | 0.002212254 | -344.483 | -340.397 | -342.895 |
| GEV-3P/LMOM | 0.0007366733 | -405.162 | -399.033 | -402.78 |
| Normal-2P/LMOM | 0.001410178 | -370.15 | -366.064 | -368.562 |
| NORMAL-2P/MLE | 0.001407119 | -370.274 | -366.188 | -368.686 |
| LOGISTIC-2P/MLE | 0.0009359899 | -393.513 | -389.427 | -391.925 |
| GEV-3P/MLE | 0.10776 | -120.987 | -114.858 | -118.605 |
| Gaussian KDE-Silverman Rule-of-thumb (ROT) Model | 0.0003034065 | -459.725 | -457.682 | -458.931 |
| Gaussian KDE-Thumb Hand Rule by Scott (1992) Model | 0.0003224862 | -456.249 | -454.206 | -455.455 |
| Gaussian KDE-Methods of Sheather & Jones (1991) via Direct Plug-in (DPI) Model | 0.0003370344 | -453.734 | -451.691 | -452.94 |
| Gaussian KDE-Methods of Sheather & Jones (1991) via Solve-the-Equation (STE) Model | 0.00033945 | -453.326 | -451.283 | -452.532 |
| Gaussian KDE-Biased-cross validation (BCV) by Scott and Terrell (1987) Model | 0.0003707281 | -448.302 | -446.259 | -447.508 |
| Gaussian KDE-Unbiased cross validation (UCV) by Scott and Terrell (1987) Model | 0.0003705832 | -448.325 | -446.282 | -447.531 |
| Gaussian KDE-Least Square Cross Validation (LSCV) Model | 0.0003712191 | -448.227 | -446.184 | -447.433 |
| Gaussian KDE-Normal Scale (NS) Model | 0.0003589865 | -450.137 | -448.094 | -449.343 |
| Gaussian KDE-Smoothed cross-validation (SCV) model | 0.0003539034 | -450.95 | -448.907 | -450.156 |

| (c-4)Highest MT (Time lag = ±2 day) ($℃)$  AMWGS (  (EVSG-1) (Ottawa region) | Mean Square Error (MSE) | Akaike Information Criterion (AIC) | Bayesian Information Criterion (BIC) | Hannan-Quinn Information Criterion (HQC) |
| --- | --- | --- | --- | --- |
| Gamma-2P/LMOM | 0.001818783 | -355.646 | -351.56 | -354.058 |
| GEV-3P/LMOM | 0.0004561549 | -432.483 | -426.353 | -430.101 |
| Normal-2P/LMOM | 0.00113181 | -382.684 | -378.598 | -381.096 |
| NORMAL-2P/MLE | 0.001135898 | -382.479 | -378.393 | -380.891 |
| LOGISTIC-2P/MLE | 0.0007935133 | -402.925 | -398.839 | -401.337 |
| GEV-3P/MLE | 0.332958 | -56.6851 | -50.556 | -54.3031 |
| Gaussian KDE-Silverman Rule-of-thumb (ROT) Model | 0.0002707735 | -466.211 | -464.168 | -465.417 |
| Gaussian KDE-Thumb Hand Rule by Scott (1992) Model | 0.0002958723 | -461.158 | -459.115 | -460.364 |
| Gaussian KDE-Methods of Sheather & Jones (1991) via Direct Plug-in (DPI) Model | 0.0003064801 | -459.15 | -457.107 | -458.356 |
| Gaussian KDE-Methods of Sheather & Jones (1991) via Solve-the-Equation (STE) Model | 0.0003075652 | -458.949 | -456.906 | -458.155 |
| Gaussian KDE-Biased-cross validation (BCV) by Scott and Terrell (1987) Model | 0.0003169825 | -457.23 | -455.187 | -456.436 |
| Gaussian KDE-Unbiased cross validation (UCV) by Scott and Terrell (1987) Model | 0.0003167171 | -457.278 | -455.235 | -456.484 |
| Gaussian KDE-Least Square Cross Validation (LSCV) Model | 0.0003172202 | -457.187 | -455.144 | -456.393 |
| Gaussian KDE-Normal Scale (NS) Model | 0.0003058768 | -459.263 | -457.22 | -458.469 |
| Gaussian KDE-Smoothed cross-validation (SCV) model | 0.0003129366 | -457.962 | -455.919 | -457.168 |

| (c-5) MT (Time lag = ±3 day) ($℃)$  Ottawa | Mean Square Error (MSE) | Akaike Information Criterion (AIC) | Bayesian Information Criterion (BIC) | Hannan-Quinn Information Criterion (HQC) |
| --- | --- | --- | --- | --- |
| Gamma-2P/LMOM | 0.001098335 | -384.396 | -380.31 | -382.808 |
| GEV-3P/LMOM | 0.0003870459 | -441.847 | -435.718 | -439.465 |
| Normal-2P/LMOM | 0.000661518 | -413.295 | -409.209 | -411.707 |
| NORMAL-2P/MLE | 0.0006603231 | -413.399 | -409.312 | -411.811 |
| LOGISTIC-2P/MLE | 0.0005986566 | -418.987 | -414.901 | -417.399 |
| GEV-3P/MLE | 0.007841605 | -270.354 | -264.225 | -267.972 |
| Gaussian KDE-Silverman Rule-of-thumb (ROT) Model* | 0.0002487905 | -471.037 | -468.994 | -470.243 |
| Gaussian KDE-Thumb Hand Rule by Scott (1992) Model | 0.0002747522 | -465.38 | -463.336 | -464.586 |
| Gaussian KDE-Methods of Sheather & Jones (1991) via Direct Plug-in (DPI) Model | 0.0002834522 | -463.603 | -461.56 | -462.809 |
| Gaussian KDE-Methods of Sheather & Jones (1991) via Solve-the-Equation (STE) Model | 0.0002843341 | -463.426 | -461.383 | -462.632 |
| Gaussian KDE-Biased-cross validation (BCV) by Scott and Terrell (1987) Model | 0.0002877406 | -462.747 | -460.704 | -461.953 |
| Gaussian KDE-Unbiased cross validation (UCV) by Scott and Terrell (1987) Model | 0.0002879031 | -462.715 | -460.671 | -461.921 |
| Gaussian KDE-Least Square Cross Validation (LSCV) Model | 0.0002884694 | -462.603 | -460.559 | -461.809 |
| Gaussian KDE-Normal Scale (NS) Model | 0.0002746227 | -465.406 | -463.363 | -464.612 |
| Gaussian KDE-Smoothed cross-validation (SCV) model | 0.0002886905 | -462.559 | -460.516 | -461.765 |

| (d-0) AHMT ($℃)$ (TORONTO) | Mean Square Error (MSE) | Akaike Information Criterion (AIC) | Bayesian Information Criterion (BIC) | Hannan-Quinn Information Criterion (HQC) |
| --- | --- | --- | --- | --- |
| Gamma-2P/LMOM | 0.0005526813 | -251.025 | -247.972 | -249.984 |
| GEV-3P/LMOM | 0.0004826761 | -253.63 | -249.051 | -252.068 |
| Normal-2P/LMOM | 0.0006867739 | -243.639 | -240.586 | -242.598 |
| NORMAL-2P/MLE | 0.0008989338 | -234.486 | -231.434 | -233.445 |
| LOGISTIC-2P/MLE | 0.0009513059 | -232.561 | -229.508 | -231.52 |
| GEV-3P/MLE | 0.002595928 | -196.43 | -191.85 | -194.868 |
| Gaussian KDE-Silverman Rule-of-thumb (ROT) Model | 0.000340521 | -269.491 | -267.965 | -268.971 |
| Gaussian KDE-Thumb Hand Rule by Scott (1992) Model | 0.0003840402 | -265.402 | -263.876 | -264.881 |
| Gaussian KDE-Methods of Sheather & Jones (1991) via Direct Plug-in (DPI) Model | 0.0004343234 | -261.219 | -259.692 | -260.698 |
| Gaussian KDE-Methods of Sheather & Jones (1991) via Solve-the-Equation (STE) Model | 0.0004400796 | -260.771 | -259.245 | -260.25 |
| Gaussian KDE-Biased-cross validation (BCV) by Scott and Terrell (1987) Model | 0.0004073951 | -263.395 | -261.868 | -262.874 |
| Gaussian KDE-Unbiased cross validation (UCV) by Scott and Terrell (1987) Model | 0.0004076378 | -263.374 | -261.848 | -262.854 |
| Gaussian KDE-Least Square Cross Validation (LSCV) Model | 0.0004073067 | -263.402 | -261.876 | -262.882 |
| Gaussian KDE-Normal Scale (NS) Model | 0.0003838185 | -265.422 | -263.895 | -264.901 |
| Gaussian KDE-Smoothed cross-validation (SCV) model | 0.0004486263 | -260.117 | -258.591 | -259.596 |

| (d-2) WGS (Time lag = 0 day) km/hr  TORONTO | Mean Square Error (MSE) | Akaike Information Criterion (AIC) | Bayesian Information Criterion (BIC) | Hannan-Quinn Information Criterion (HQC) |
| --- | --- | --- | --- | --- |
| Gamma-2P/LMOM | 0.01658137 | -135.382 | -132.329 | -134.341 |
| GEV-3P/LMOM | 0.003145851 | -189.897 | -185.318 | -188.335 |
| Normal-2P/LMOM | 0.007357095 | -163.011 | -159.958 | -161.97 |
| NORMAL-2P/MLE | 0.007406435 | -162.784 | -159.731 | -161.743 |
| LOGISTIC-2P/MLE | 0.003846026 | -185.064 | -182.012 | -184.023 |
| GEV-3P/MLE | 0.3326766 | -31.4199 | -26.8408 | -29.8583 |
| Gaussian KDE-Silverman Rule-of-thumb (ROT) Model* | 0.001286566 | -224.296 | -222.77 | -223.776 |
| Gaussian KDE-Thumb Hand Rule by Scott (1992) Model | 0.001298717 | -223.977 | -222.451 | -223.456 |
| Gaussian KDE-Methods of Sheather & Jones (1991) via Direct Plug-in (DPI) Model | 0.001495969 | -219.169 | -217.643 | -218.649 |
| Gaussian KDE-Methods of Sheather & Jones (1991) via Solve-the-Equation (STE) Model | 0.001404549 | -221.313 | -219.787 | -220.793 |
| Gaussian KDE-Biased-cross validation (BCV) by Scott and Terrell (1987) Model | 0.001602754 | -216.825 | -215.299 | -216.305 |
| Gaussian KDE-Unbiased cross validation (UCV) by Scott and Terrell (1987) Model | 0.0008656413 | -237.769 | -236.243 | -237.249 |
| Gaussian KDE-Least Square Cross Validation (LSCV) Model | 0.00133826 | -222.957 | -221.431 | -222.437 |
| Gaussian KDE-Normal Scale (NS) Model | 0.001632852 | -216.193 | -214.666 | -215.672 |
| Gaussian KDE-Smoothed cross-validation (SCV) model | 0.001291816 | -224.158 | -222.632 | -223.637 |

| (d-3) WGS (Time lag = ±1 days ) km/hr  TORONTO | Mean Square Error (MSE) | Akaike Information Criterion (AIC) | Bayesian Information Criterion (BIC) | Hannan-Quinn Information Criterion (HQC) |
| --- | --- | --- | --- | --- |
| Gamma-2P/LMOM | 0.01014015 | -152.103 | -149.05 | -151.062 |
| GEV-3P/LMOM | 0.003409311 | -187.162 | -182.583 | -185.601 |
| Normal-2P/LMOM | 0.005713445 | -171.608 | -168.555 | -170.567 |
| NORMAL-2P/MLE | 0.006491799 | -167.265 | -164.213 | -166.224 |
| LOGISTIC-2P/MLE | 0.002396364 | -201.149 | -198.097 | -200.108 |
| GEV-3P/MLE | 0.3326766 | -31.4199 | -26.8408 | -29.8583 |
| Gaussian KDE-Silverman Rule-of-thumb (ROT) Model* | 0.001394398 | -221.56 | -220.034 | -221.039 |
| Gaussian KDE-Thumb Hand Rule by Scott (1992) Model | 0.001489241 | -219.323 | -217.796 | -218.802 |
| Gaussian KDE-Methods of Sheather & Jones (1991) via Direct Plug-in (DPI) Model | 0.001392306 | -221.611 | -220.085 | -221.09 |
| Gaussian KDE-Methods of Sheather & Jones (1991) via Solve-the-Equation (STE) Model | 0.001834923 | -212.226 | -210.699 | -211.705 |
| Gaussian KDE-Biased-cross validation (BCV) by Scott and Terrell (1987) Model | 0.001452832 | -220.164 | -218.638 | -219.644 |
| Gaussian KDE-Unbiased cross validation (UCV) by Scott and Terrell (1987) Model | 0.001248086 | -225.329 | -223.803 | -224.808 |
| Gaussian KDE-Least Square Cross Validation (LSCV) Model | 0.001628102 | -216.292 | -214.765 | -215.771 |
| Gaussian KDE-Normal Scale (NS) Model | 0.001497878 | -219.126 | -217.6 | -218.605 |
| Gaussian KDE-Smoothed cross-validation (SCV) model | 0.001450677 | -220.215 | -218.688 | -219.694 |

| (d-4) WGS (Time lag = ±2 days ) km/hr  TORONTO | Mean Square Error (MSE) | Akaike Information Criterion (AIC) | Bayesian Information Criterion (BIC) | Hannan-Quinn Information Criterion (HQC) |
| --- | --- | --- | --- | --- |
| Gamma-2P/LMOM | 0.003305593 | -190.213 | -187.16 | -189.172 |
| GEV-3P/LMOM | 0.001808492 | -208.719 | -204.14 | -207.157 |
| Normal-2P/LMOM | 0.00194269 | -208.285 | -205.232 | -207.244 |
| NORMAL-2P/MLE | 0.002368432 | -201.548 | -198.495 | -200.507 |
| LOGISTIC-2P/MLE | 0.00123857 | -223.589 | -220.536 | -222.548 |
| GEV-3P/MLE | 0.3326766 | -31.4199 | -26.8408 | -29.8583 |
| Gaussian KDE-Silverman Rule-of-thumb (ROT) Model | 0.0007603964 | -242.177 | -240.65 | -241.656 |
| Gaussian KDE-Thumb Hand Rule by Scott (1992) Model | 0.0009461958 | -234.744 | -233.218 | -234.224 |
| Gaussian KDE-Methods of Sheather & Jones (1991) via Direct Plug-in (DPI) Model | 0.0007812041 | -241.259 | -239.733 | -240.738 |
| Gaussian KDE-Methods of Sheather & Jones (1991) via Solve-the-Equation (STE) Model | 0.0008813096 | -237.159 | -235.633 | -236.639 |
| Gaussian KDE-Biased-cross validation (BCV) by Scott and Terrell (1987) Model | 0.001048628 | -231.249 | -229.723 | -230.729 |
| Gaussian KDE-Unbiased cross validation (UCV) by Scott and Terrell (1987) Model | 0.0008891221 | -236.859 | -235.333 | -236.339 |
| Gaussian KDE-Least Square Cross Validation (LSCV) Model | 0.001017643 | -232.269 | -230.743 | -231.749 |
| Gaussian KDE-Normal Scale (NS) Model | 0.001047469 | -231.287 | -229.761 | -230.766 |
| Gaussian KDE-Smoothed cross-validation (SCV) model | 0.0008337261 | -239.047 | -237.52 | -238.526 |

| (d-5) WGS (Time lag = ±3 days) km/hr  (TORONTO | Mean Square Error (MSE) | Akaike Information Criterion (AIC) | Bayesian Information Criterion (BIC) | Hannan-Quinn Information Criterion (HQC) |
| --- | --- | --- | --- | --- |
| Gamma-2P/LMOM | 0.00201429 | -207.055 | -204.002 | -206.014 |
| GEV-3P/LMOM | 0.001601156 | -212.859 | -208.28 | -211.297 |
| Normal-2P/LMOM | 0.001458529 | -218.031 | -214.978 | -216.99 |
| NORMAL-2P/MLE | 0.001458759 | -218.026 | -214.973 | -216.985 |
| LOGISTIC-2P/MLE | 0.001597277 | -214.941 | -211.889 | -213.9 |
| GEV-3P/MLE | 0.004391513 | -178.555 | -173.976 | -176.993 |
| Gaussian KDE-Silverman Rule-of-thumb (ROT) Model | 0.0008669458 | -237.718 | -236.192 | -237.198 |
| Gaussian KDE-Thumb Hand Rule by Scott (1992) Model | 0.0009594024 | -234.273 | -232.746 | -233.752 |
| Gaussian KDE-Methods of Sheather & Jones (1991) via Direct Plug-in (DPI) Model | 0.000914725 | -235.894 | -234.368 | -235.374 |
| Gaussian KDE-Methods of Sheather & Jones (1991) via Solve-the-Equation (STE) Model | 0.0008823954 | -237.118 | -235.591 | -236.597 |
| Gaussian KDE-Biased-cross validation (BCV) by Scott and Terrell (1987) Model | 0.001051843 | -231.145 | -229.619 | -230.625 |
| Gaussian KDE-Unbiased cross validation (UCV) by Scott and Terrell (1987) Model | 0.001051687 | -231.15 | -229.624 | -230.63 |
| Gaussian KDE-Least Square Cross Validation (LSCV) Model | 0.001049286 | -231.228 | -229.702 | -230.707 |
| Gaussian KDE-Normal Scale (NS) Model | 0.001014018 | -232.39 | -230.864 | -231.87 |
| Gaussian KDE-Smoothed cross-validation (SCV) model | 0.0009668951 | -234.008 | -232.482 | -233.488 |

| (e-!) AHMT $℃$(Vancouver) | Mean Square Error (MSE) | Akaike Information Criterion (AIC) | Bayesian Information Criterion (BIC) | Hannan-Quinn Information Criterion (HQC) |
| --- | --- | --- | --- | --- |
| Gamma-2P/LMOM | 0.00145642 | -374.843 | -370.722 | -373.238 |
| GEV-3P/LMOM | 0.001091198 | -389.588 | -383.406 | -387.18 |
| Normal-2P/LMOM | 0.001683908 | -366.425 | -362.304 | -364.82 |
| NORMAL-2P/MLE | 0.001756813 | -363.967 | -359.846 | -362.362 |
| LOGISTIC-2P/MLE | 0.0009933256 | -397.038 | -392.917 | -395.433 |
| GEV-3P/MLE | 0.002149317 | -350.271 | -344.09 | -347.863 |
| Gaussian KDE-Silverman Rule-of-thumb (ROT) Model* | 0.0006859234 | -420.515 | -418.455 | -419.713 |
| Gaussian KDE-Thumb Hand Rule by Scott (1992) Model | 0.0007601084 | -414.559 | -412.498 | -413.756 |
| Gaussian KDE-Methods of Sheather & Jones (1991) via Direct Plug-in (DPI) Model | 0.0007576811 | -414.744 | -412.684 | -413.942 |
| Gaussian KDE-Methods of Sheather & Jones (1991) via Solve-the-Equation (STE) Model | 0.0007573667 | -414.768 | -412.708 | -413.966 |
| Gaussian KDE-Biased-cross validation (BCV) by Scott and Terrell (1987) Model | 0.0008283532 | -409.572 | -407.512 | -408.77 |
| Gaussian KDE-Unbiased cross validation (UCV) by Scott and Terrell (1987) Model | 0.0002724777 | -474.061 | -472.001 | -473.259 |
| Gaussian KDE-Least Square Cross Validation (LSCV) Model | 0.0003515623 | -459.281 | -457.221 | -458.479 |
| Gaussian KDE-Normal Scale (NS) Model | 0.0008049125 | -411.237 | -409.177 | -410.434 |
| Gaussian KDE-Smoothed cross-validation (SCV) model | 0.0007875843 | -412.499 | -410.439 | -411.697 |

| (e-2) WGS (Time lag = 0 day) km/hr  (VANCOUVER | Mean Square Error (MSE) | Akaike Information Criterion (AIC) | Bayesian Information Criterion (BIC) | Hannan-Quinn Information Criterion (HQC) |
| --- | --- | --- | --- | --- |
| Gamma-2P/LMOM | 0.1894491 | -92.4908 | -88.3699 | -90.8857 |
| GEV-3P/LMOM | 0.0688825 | -149.17 | -142.989 | -146.763 |
| Normal-2P/LMOM | 0.07004814 | -150.197 | -146.076 | -148.592 |
| NORMAL-2P/MLE | 0.05479122 | -164.445 | -160.324 | -162.84 |
| LOGISTIC-2P/MLE | 0.04881671 | -171.142 | -167.021 | -169.536 |
| GEV-3P/MLE | 0.06217118 | -155.116 | -148.935 | -152.708 |
| Gaussian KDE-Silverman Rule-of-thumb (ROT) Model* | 0.04767019 | -174.52 | -172.46 | -173.717 |
| Gaussian KDE-Thumb Hand Rule by Scott (1992) Model | NA | NA | NA | NA |
| Gaussian KDE-Methods of Sheather & Jones (1991) via Direct Plug-in (DPI) Model | NA | NA | NA | NA |
| Gaussian KDE-Methods of Sheather & Jones (1991) via Solve-the-Equation (STE) Model | NA | NA | NA | NA |
| Gaussian KDE-Biased-cross validation (BCV) by Scott and Terrell (1987) Model | 0.04741746 | -174.828 | -172.768 | -174.026 |
| Gaussian KDE-Unbiased cross validation (UCV) by Scott and Terrell (1987) Model | 0.04774938 | -174.424 | -172.363 | -173.621 |
| Gaussian KDE-Least Square Cross Validation (LSCV) Model | 0.04703725 | -175.295 | -173.235 | -174.493 |
| Gaussian KDE-Normal Scale (NS) Model | 0.05153728 | -169.996 | -167.936 | -169.194 |
| Gaussian KDE-Smoothed cross-validation (SCV) model | 0.04977493 | -172.014 | -169.954 | -171.212 |

| (e-3)WGS (Time lag = ±1 DAYS) km/hr  (VANCOUVER ) | Mean Square Error (MSE) | Akaike Information Criterion (AIC) | Bayesian Information Criterion (BIC) | Hannan-Quinn Information Criterion (HQC) |
| --- | --- | --- | --- | --- |
| Gamma-2P/LMOM | 0.03997943 | -182.725 | -178.604 | -181.119 |
| GEV-3P/LMOM | 0.01739226 | -229 | -222.819 | -226.593 |
| Normal-2P/LMOM | 0.01596092 | -235.981 | -231.861 | -234.376 |
| NORMAL-2P/MLE | 0.01513238 | -239.073 | -234.952 | -237.468 |
| LOGISTIC-2P/MLE | 0.014115 | -243.11 | -238.989 | -241.505 |
| GEV-3P/MLE | 0.02010915 | -220.582 | -214.4 | -218.174 |
| Gaussian KDE-Silverman Rule-of-thumb (ROT) Model* | 0.007656587 | -280.587 | -278.527 | -279.784 |
| Gaussian KDE-Thumb Hand Rule by Scott (1992) Model | 0.009401933 | -268.677 | -266.616 | -267.874 |
| Gaussian KDE-Methods of Sheather & Jones (1991) via Direct Plug-in (DPI) Model | 0.008348579 | -275.569 | -273.508 | -274.766 |
| Gaussian KDE-Methods of Sheather & Jones (1991) via Solve-the-Equation (STE) Model | 0.0067796 | -287.643 | -285.582 | -286.84 |
| Gaussian KDE-Biased-cross validation (BCV) by Scott and Terrell (1987) Model | 0.009427123 | -268.522 | -266.461 | -267.719 |
| Gaussian KDE-Unbiased cross validation (UCV) by Scott and Terrell (1987) Model | 0.02070297 | -222.894 | -220.833 | -222.091 |
| Gaussian KDE-Least Square Cross Validation (LSCV) Model | 0.007489277 | -281.868 | -279.808 | -281.066 |
| Gaussian KDE-Normal Scale (NS) Model | 0.009401586 | -268.679 | -266.618 | -267.876 |
| Gaussian KDE-Smoothed cross-validation (SCV) model | 0.007251106 | -283.743 | -281.682 | -282.94 |

| (e-4) WGS (Time lag = ±2 DAYS) km/hr  (VANCOUVER | Mean Square Error (MSE) | Akaike Information Criterion (AIC) | Bayesian Information Criterion (BIC) | Hannan-Quinn Information Criterion (HQC) |
| --- | --- | --- | --- | --- |
| Gamma-2P/LMOM | 0.02965164 | -200.058 | -195.937 | -198.453 |
| GEV-3P/LMOM | 0.008346949 | -271.58 | -265.399 | -269.172 |
| Normal-2P/LMOM | 0.01578884 | -236.61 | -232.489 | -235.005 |
| NORMAL-2P/MLE | 0.01495626 | -239.752 | -235.631 | -238.147 |
| LOGISTIC-2P/MLE | 0.0113821 | -255.591 | -251.47 | -253.986 |
| GEV-3P/MLE | 0.3329649 | -57.7837 | -51.6023 | -55.3759 |
| Gaussian KDE-Silverman Rule-of-thumb (ROT) Model* | 0.002352338 | -349.036 | -346.976 | -348.233 |
| Gaussian KDE-Thumb Hand Rule by Scott (1992) Model | 0.003192389 | -331.325 | -329.265 | -330.523 |
| Gaussian KDE-Methods of Sheather & Jones (1991) via Direct Plug-in (DPI) Model | 0.002317276 | -349.907 | -347.847 | -349.104 |
| Gaussian KDE-Methods of Sheather & Jones (1991) via Solve-the-Equation (STE) Model | 0.02147317 | -220.775 | -218.715 | -219.973 |
| Gaussian KDE-Biased-cross validation (BCV) by Scott and Terrell (1987) Model | 0.01622802 | -237.019 | -234.958 | -236.216 |
| Gaussian KDE-Unbiased cross validation (UCV) by Scott and Terrell (1987) Model | 0.001990144 | -358.734 | -356.673 | -357.931 |
| Gaussian KDE-Least Square Cross Validation (LSCV) Model | 0.002112633 | -355.27 | -353.209 | -354.467 |
| Gaussian KDE-Normal Scale (NS) Model | 0.003189435 | -331.379 | -329.318 | -330.576 |
| Gaussian KDE-Smoothed cross-validation (SCV) model | 0.002291832 | -350.547 | -348.487 | -349.745 |

| (e-5) WGS (Time lag = ±3 DAYS) km/hr  (VANCOUVER) | Mean Square Error (MSE) | Akaike Information Criterion (AIC) | Bayesian Information Criterion (BIC) | Hannan-Quinn Information Criterion (HQC) |
| --- | --- | --- | --- | --- |
| Gamma-2P/LMOM | 0.02093688 | -220.242 | -216.121 | -218.637 |
| GEV-3P/LMOM | 0.004767078 | -304.069 | -297.888 | -301.662 |
| Normal-2P/LMOM | 0.0124927 | -250.191 | -246.071 | -248.586 |
| NORMAL-2P/MLE | 0.01327865 | -246.653 | -242.532 | -245.048 |
| LOGISTIC-2P/MLE | 0.005415512 | -298.672 | -294.551 | -297.067 |
| GEV-3P/MLE | 0.3329649 | -57.7837 | -51.6023 | -55.3759 |
| Gaussian KDE-Silverman Rule-of-thumb (ROT) Model* | 0.0006746263 | -421.478 | -419.418 | -420.676 |
| Gaussian KDE-Thumb Hand Rule by Scott (1992) Model | 0.0007618307 | -414.428 | -412.367 | -413.625 |
| Gaussian KDE-Methods of Sheather & Jones (1991) via Direct Plug-in (DPI) Model | 0.0006451797 | -424.067 | -422.006 | -423.264 |
| Gaussian KDE-Methods of Sheather & Jones (1991) via Solve-the-Equation (STE) Model | 0.0005914494 | -429.11 | -427.05 | -428.308 |
| Gaussian KDE-Biased-cross validation (BCV) by Scott and Terrell (1987) Model | 0.001883024 | -361.943 | -359.882 | -361.14 |
| Gaussian KDE-Unbiased cross validation (UCV) by Scott and Terrell (1987) Model | 0.01258662 | -251.757 | -249.697 | -250.954 |
| Gaussian KDE-Least Square Cross Validation (LSCV) Model | 0.000666861 | -422.15 | -420.089 | -421.347 |
| Gaussian KDE-Normal Scale (NS) Model | 0.0009061392 | -404.366 | -402.306 | -403.564 |
| Gaussian KDE-Smoothed cross-validation (SCV) model | 0.0007715968 | -413.689 | -411.628 | -412.886 |

| (f-1) AMWGS km/hr (Regina) | Mean Square Error (MSE) | Akaike Information Criterion (AIC) | Bayesian Information Criterion (BIC) | Hannan-Quinn Information Criterion (HQC) |
| --- | --- | --- | --- | --- |
| Gamma-2P/LMOM | 0.001014542 | -278.626 | -275.199 | -277.378 |
| GEV-3P/LMOM | 0.0003796063 | -316.931 | -311.791 | -315.059 |
| Normal-2P/LMOM | 0.001944813 | -251.946 | -248.519 | -250.698 |
| NORMAL-2P/MLE | 0.001979593 | -251.219 | -247.792 | -249.971 |
| LOGISTIC-2P/MLE | 0.001195647 | -271.892 | -268.465 | -270.644 |
| GEV-3P/MLE | 0.0004473289 | -310.201 | -305.06 | -308.329 |
| Gaussian KDE-Silverman Rule-of-thumb (ROT) Model | 0.000356584 | -323.497 | -321.783 | -322.873 |
| Gaussian KDE-Thumb Hand Rule by Scott (1992) Model | 0.0003891355 | -319.915 | -318.201 | -319.291 |
| Gaussian KDE-Methods of Sheather & Jones (1991) via Direct Plug-in (DPI) Model | 0.0003884773 | -319.984 | -318.271 | -319.36 |
| Gaussian KDE-Methods of Sheather & Jones (1991) via Solve-the-Equation (STE) Model | 0.0003884719 | -319.985 | -318.271 | -319.361 |
| Gaussian KDE-Biased-cross validation (BCV) by Scott and Terrell (1987) Model | 0.000415215 | -317.255 | -315.542 | -316.631 |
| Gaussian KDE-Unbiased cross validation (UCV) by Scott and Terrell (1987) Model | 0.0004155986 | -317.217 | -315.504 | -316.593 |
| Gaussian KDE-Least Square Cross Validation (LSCV) Model | 0.0004155843 | -317.219 | -315.505 | -316.595 |
| Gaussian KDE-Normal Scale (NS) Model | 0.0003942067 | -319.384 | -317.67 | -318.76 |
| Gaussian KDE-Smoothed cross-validation (SCV) model | 0.0003988791 | -318.901 | -317.187 | -318.277 |

| (f-2) MT (Time lag = 0 day) ($℃)$  (Regina) | Mean Square Error (MSE) | Akaike Information Criterion (AIC) | Bayesian Information Criterion (BIC) | Hannan-Quinn Information Criterion (HQC) |
| --- | --- | --- | --- | --- |
| Gamma-2P/LMOM | 0.008629513 | -190.855 | -187.428 | -189.607 |
| GEV-3P/LMOM | 0.001159457 | -271.152 | -266.011 | -269.28 |
| Normal-2P/LMOM | 0.005609947 | -208.512 | -205.085 | -207.264 |
| NORMAL-2P/MLE | 0.005569593 | -208.808 | -205.381 | -207.56 |
| LOGISTIC-2P/MLE | 0.003125498 | -232.495 | -229.067 | -231.247 |
| GEV-3P/MLE | 0.07537391 | -99.9971 | -94.8563 | -98.1251 |
| Gaussian KDE-Silverman Rule-of-thumb (ROT) Model | 0.0004676256 | -312.382 | -310.668 | -311.758 |
| Gaussian KDE-Thumb Hand Rule by Scott (1992) Model | 0.0005430765 | -306.249 | -304.535 | -305.625 |
| Gaussian KDE-Methods of Sheather & Jones (1991) via Direct Plug-in (DPI) Model | 0.0004338076 | -315.459 | -313.746 | -314.835 |
| Gaussian KDE-Methods of Sheather & Jones (1991) via Solve-the-Equation (STE) Model | 0.0004501443 | -313.944 | -312.23 | -313.32 |
| Gaussian KDE-Biased-cross validation (BCV) by Scott and Terrell (1987) Model | 0.0006368744 | -299.716 | -298.003 | -299.092 |
| Gaussian KDE-Unbiased cross validation (UCV) by Scott and Terrell (1987) Model | 0.0004278711 | -316.03 | -314.32 | -315.4 |
| Gaussian KDE-Least Square Cross Validation (LSCV) Model | 0.0004278782 | -316.02 | -314.31 | -315.3 |
| Gaussian KDE-Normal Scale (NS) Model | 0.0006036986 | -301.91 | -300.196 | -301.286 |
| Gaussian KDE-Smoothed cross-validation (SCV) model | 0.000468533 | -312.302 | -310.588 | -311.678 |

| (f-3) MT (Time lag = ±1 days) ($℃)$  (Regina) | Mean Square Error (MSE) | Akaike Information Criterion (AIC) | Bayesian Information Criterion (BIC) | Hannan-Quinn Information Criterion (HQC) |
| --- | --- | --- | --- | --- |
| Gamma-2P/LMOM | 0.01047056 | -182.9266985 | -179.5 | -181.679 |
| GEV-3P/LMOM | 0.005037016 | -210.9285989 | -205.788 | -209.057 |
| Normal-2P/LMOM | 0.008428258 | -191.822772 | -188.396 | -190.575 |
| NORMAL-2P/MLE | 0.008420162 | -191.8621747 | -188.435 | -190.614 |
| LOGISTIC-2P/MLE | 0.006718196 | -201.1203601 | -197.693 | -199.872 |
| GEV-3P/MLE | 0.3327984 | -39.10895349 | -33.9682 | -37.237 |
| Gaussian KDE-Silverman Rule-of-thumb (ROT) Model | 0.005003527 | -215.2021008 | -213.489 | -214.578 |
| Gaussian KDE-Thumb Hand Rule by Scott (1992) Model | 0.005086768 | -214.5256174 | -212.812 | -213.902 |
| Gaussian KDE-Methods of Sheather & Jones (1991) via Direct Plug-in (DPI) Model | 0.005037219 | -214.9269465 | -213.213 | -214.303 |
| Gaussian KDE-Methods of Sheather & Jones (1991) via Solve-the-Equation (STE) Model | 0.005027002 | -215.0101914 | -213.297 | -214.386 |
| Gaussian KDE-Biased-cross validation (BCV) by Scott and Terrell (1987) Model | 0.005149988 | -214.0191967 | -212.306 | -213.395 |
| Gaussian KDE-Unbiased cross validation (UCV) by Scott and Terrell (1987) Model | 0.005136334 | -214.128043 | -212.414 | -213.504 |
| Gaussian KDE-Least Square Cross Validation (LSCV) Model | 0.005136534 | -214.1264466 | -212.413 | -213.502 |
| Gaussian KDE-Normal Scale (NS) Model | 0.005108116 | -214.3539099 | -212.64 | -213.73 |
| Gaussian KDE-Smoothed cross-validation (SCV) model | 0.005060005 | -214.7419001 | -213.028 | -214.118 |

| (f-4) MT (Time lag = ±2 days) ($℃)$  (Regina) | Mean Square Error (MSE) | Akaike Information Criterion (AIC) | Bayesian Information Criterion (BIC) | Hannan-Quinn Information Criterion (HQC) |
| --- | --- | --- | --- | --- |
| Gamma-2P/LMOM | 0.005028854 | -212.9950893 | -209.568 | -211.747 |
| GEV-3P/LMOM | 0.003187581 | -229.6882112 | -224.547 | -227.816 |
| Normal-2P/LMOM | 0.004131819 | -221.0505389 | -217.623 | -219.803 |
| NORMAL-2P/MLE | 0.004156989 | -220.8015349 | -217.374 | -219.554 |
| LOGISTIC-2P/MLE | 0.003194136 | -231.6039847 | -228.177 | -230.356 |
| GEV-3P/MLE | 0.015742 | -164.2083421 | -159.068 | -162.336 |
| Gaussian KDE-Silverman Rule-of-thumb (ROT) Model | 0.002522272 | -243.2864031 | -241.573 | -242.662 |
| Gaussian KDE-Thumb Hand Rule by Scott (1992) Model | 0.002610074 | -241.8834449 | -240.17 | -241.259 |
| Gaussian KDE-Methods of Sheather & Jones (1991) via Direct Plug-in (DPI) Model | 0.002534877 | -243.0820167 | -241.368 | -242.458 |
| Gaussian KDE-Methods of Sheather & Jones (1991) via Solve-the-Equation (STE) Model | 0.002474421 | -244.0717028 | -242.358 | -243.448 |
| Gaussian KDE-Biased-cross validation (BCV) by Scott and Terrell (1987) Model | 0.00280644 | -238.9093786 | -237.196 | -238.285 |
| Gaussian KDE-Unbiased cross validation (UCV) by Scott and Terrell (1987) Model | 0.002525704 | -243.2306532 | -241.517 | -242.607 |
| Gaussian KDE-Least Square Cross Validation (LSCV) Model | 0.002517642 | -243.3617337 | -241.648 | -242.738 |
| Gaussian KDE-Normal Scale (NS) Model | 0.002737624 | -239.9272604 | -238.214 | -239.303 |
| Gaussian KDE-Smoothed cross-validation (SCV) model | 0.00263009 | -241.5702257 | -239.857 | -240.946 |

| (f-5) MT (Time lag = ±3 days) ($℃)$  (Regina) | Mean Square Error (MSE) | Akaike Information Criterion (AIC) | Bayesian Information Criterion (BIC) | Hannan-Quinn Information Criterion (HQC) |
| --- | --- | --- | --- | --- |
| Gamma-2P/LMOM | 0.003911503 | -223.2971768 | -219.87 | -222.049 |
| GEV-3P/LMOM | 0.003157449 | -230.0776251 | -224.937 | -228.206 |
| Normal-2P/LMOM | 0.003428174 | -228.7048284 | -225.278 | -227.457 |
| NORMAL-2P/MLE | 0.003455424 | -228.3802145 | -224.953 | -227.132 |
| LOGISTIC-2P/MLE | 0.003199014 | -231.5414183 | -228.114 | -230.293 |
| GEV-3P/MLE | 0.01332775 | -171.034185 | -165.893 | -169.162 |
| Gaussian KDE-Silverman Rule-of-thumb (ROT) Model | 0.002328797 | -246.5585416 | -244.845 | -245.935 |
| Gaussian KDE-Thumb Hand Rule by Scott (1992) Model | 0.002444871 | -244.5642796 | -242.851 | -243.94 |
| Gaussian KDE-Methods of Sheather & Jones (1991) via Direct Plug-in (DPI) Model | 0.002295431 | -247.1502208 | -245.437 | -246.526 |
| Gaussian KDE-Methods of Sheather & Jones (1991) via Solve-the-Equation (STE) Model | 0.002174439 | -249.3703677 | -247.657 | -248.746 |
| Gaussian KDE-Biased-cross validation (BCV) by Scott and Terrell (1987) Model | 0.002579949 | -242.3594116 | -240.646 | -241.735 |
| Gaussian KDE-Unbiased cross validation (UCV) by Scott and Terrell (1987) Model | 0.002163851 | -249.5704967 | -247.857 | -248.947 |
| Gaussian KDE-Least Square Cross Validation (LSCV) Model | 0.002164808 | -249.5523678 | -247.839 | -248.928 |
| Gaussian KDE-Normal Scale (NS) Model | 0.002505583 | -243.5585873 | -241.845 | -242.935 |
| Gaussian KDE-Smoothed cross-validation (SCV) model | 0.002347688 | -246.2272949 | -244.514 | -245.603 |

| (f-6) AHMT ($℃)$  (Regina) | Mean Square Error (MSE) | Akaike Information Criterion (AIC) | Bayesian Information Criterion (BIC) | Hannan-Quinn Information Criterion (HQC) |
| --- | --- | --- | --- | --- |
| Gamma-2P/LMOM | 0.003063527 | -233.316 | -229.889 | -232.068 |
| GEV-3P/LMOM | 0.001909533 | -250.697 | -245.556 | -248.825 |
| Normal-2P/LMOM | 0.002802769 | -236.963 | -233.536 | -235.715 |
| NORMAL-2P/MLE | 0.002812107 | -236.827 | -233.4 | -235.579 |
| LOGISTIC-2P/MLE | 0.00231834 | -244.743 | -241.316 | -243.495 |
| GEV-3P/MLE | 0.3327984 | -39.109 | -33.9682 | -37.237 |
| Gaussian KDE-Silverman Rule-of-thumb (ROT) Model | 0.00124556 | -272.215 | -270.501 | -271.591 |
| Gaussian KDE-Thumb Hand Rule by Scott (1992) Model | 0.001331989 | -269.464 | -267.751 | -268.84 |
| Gaussian KDE-Methods of Sheather & Jones (1991) via Direct Plug-in (DPI) Model | 0.001230054 | -272.729 | -271.015 | -272.105 |
| Gaussian KDE-Methods of Sheather & Jones (1991) via Solve-the-Equation (STE) Model | 0.0011728 | -274.683 | -272.969 | -274.059 |
| Gaussian KDE-Biased-cross validation (BCV) by Scott and Terrell (1987) Model | 0.001453343 | -265.889 | -264.176 | -265.265 |
| Gaussian KDE-Unbiased cross validation (UCV) by Scott and Terrell (1987) Model | 0.001125506 | -276.37 | -274.657 | -275.746 |
| Gaussian KDE-Least Square Cross Validation (LSCV) Model | 0.001120629 | -276.548 | -274.835 | -275.924 |
| Gaussian KDE-Normal Scale (NS) Model | 0.001407465 | -267.205 | -265.491 | -266.581 |
| Gaussian KDE-Smoothed cross-validation (SCV) model | 0.001279392 | -271.116 | -269.403 | -270.492 |

| (f-7) WGS (Time lag = 0 day)  (Regina) | Mean Square Error (MSE) | Akaike Information Criterion (AIC) | Bayesian Information Criterion (BIC) | Hannan-Quinn Information Criterion (HQC) |
| --- | --- | --- | --- | --- |
| Gamma-2P/LMOM | 0.01667497 | -163.848 | -160.421 | -162.6 |
| GEV-3P/LMOM | 0.006096033 | -203.105 | -197.964 | -201.233 |
| Normal-2P/LMOM | 0.008186442 | -193.016 | -189.589 | -191.768 |
| NORMAL-2P/MLE | 0.008358819 | -192.162 | -188.735 | -190.914 |
| LOGISTIC-2P/MLE | 0.005395902 | -210.107 | -206.68 | -208.859 |
| GEV-3P/MLE | 0.05371412 | -113.887 | -108.747 | -112.015 |
| Gaussian KDE-Silverman Rule-of-thumb (ROT) Model | 0.0008096159 | -289.877 | -288.163 | -289.253 |
| Gaussian KDE-Thumb Hand Rule by Scott (1992) Model | 0.001013016 | -280.688 | -278.974 | -280.064 |
| Gaussian KDE-Methods of Sheather & Jones (1991) via Direct Plug-in (DPI) Model | 0.0007361631 | -293.776 | -292.063 | -293.152 |
| Gaussian KDE-Methods of Sheather & Jones (1991) via Solve-the-Equation (STE) Model | 0.0007032168 | -295.654 | -293.94 | -295.03 |
| Gaussian KDE-Biased-cross validation (BCV) by Scott and Terrell (1987) Model | 0.001785098 | -257.46 | -255.746 | -256.836 |
| Gaussian KDE-Unbiased cross validation (UCV) by Scott and Terrell (1987) Model | 0.0006829051 | -296.855 | -295.142 | -296.231 |
| Gaussian KDE-Least Square Cross Validation (LSCV) Model | 0.0006798779 | -297.037 | -295.324 | -296.414 |
| Gaussian KDE-Normal Scale (NS) Model | 0.001715101 | -259.1 | -257.386 | -258.476 |
| Gaussian KDE-Smoothed cross-validation (SCV) model | 0.0008190699 | -289.401 | -287.687 | -288.777 |

| (f-8) WGS (Time lag = ±1 days)  (Regina) | Mean Square Error (MSE) | Akaike Information Criterion (AIC) | Bayesian Information Criterion (BIC) | Hannan-Quinn Information Criterion (HQC) |
| --- | --- | --- | --- | --- |
| Gamma-2P/LMOM | 0.0007936319 | -288.695 | -285.267 | -287.447 |
| GEV-3P/LMOM | 0.000661109 | -294.185 | -289.045 | -292.313 |
| Normal-2P/LMOM | 0.001805695 | -254.989 | -251.562 | -253.741 |
| NORMAL-2P/MLE | 0.001892088 | -253.073 | -249.646 | -251.825 |
| LOGISTIC-2P/MLE | 0.00153659 | -261.606 | -258.179 | -260.358 |
| GEV-3P/MLE | 0.0008060734 | -286.057 | -280.916 | -284.185 |
| Gaussian KDE-Silverman Rule-of-thumb (ROT) Model | 0.0004228357 | -316.51 | -314.796 | -315.886 |
| Gaussian KDE-Thumb Hand Rule by Scott (1992) Model | 0.000475409 | -311.705 | -309.991 | -311.081 |
| Gaussian KDE-Methods of Sheather & Jones (1991) via Direct Plug-in (DPI) Model | 0.0004758096 | -311.67 | -309.957 | -311.046 |
| Gaussian KDE-Methods of Sheather & Jones (1991) via Solve-the-Equation (STE) Model | 0.0004744498 | -311.788 | -310.074 | -311.164 |
| Gaussian KDE-Biased-cross validation (BCV) by Scott and Terrell (1987) Model | 0.0005058189 | -309.163 | -307.449 | -308.539 |
| Gaussian KDE-Unbiased cross validation (UCV) by Scott and Terrell (1987) Model | 0.0005059773 | -309.15 | -307.436 | -308.526 |
| Gaussian KDE-Least Square Cross Validation (LSCV) Model | 0.0005063181 | -309.122 | -307.409 | -308.498 |
| Gaussian KDE-Normal Scale (NS) Model | 0.0004751275 | -311.729 | -310.015 | -311.105 |
| Gaussian KDE-Smoothed cross-validation (SCV) model | 0.0004916149 | -310.33 | -308.617 | -309.706 |

| (f-9) WGS (Time lag = ±2 days)  (Regina) | Mean Square Error (MSE) | Akaike Information Criterion (AIC) | Bayesian Information Criterion (BIC) | Hannan-Quinn Information Criterion (HQC) |
| --- | --- | --- | --- | --- |
| Gamma-2P/LMOM | 0.0008350428 | -286.609 | -283.182 | -285.361 |
| GEV-3P/LMOM | 0.0006983483 | -291.938 | -286.798 | -290.067 |
| Normal-2P/LMOM | 0.001553613 | -261.154 | -257.727 | -259.906 |
| NORMAL-2P/MLE | 0.001552976 | -261.171 | -257.744 | -259.923 |
| LOGISTIC-2P/MLE | 0.001146552 | -273.611 | -270.184 | -272.363 |
| GEV-3P/MLE | 0.001541094 | -259.486 | -254.345 | -257.614 |
| Gaussian KDE-Silverman Rule-of-thumb (ROT) Model | 0.0004565586 | -313.364 | -311.65 | -312.74 |
| Gaussian KDE-Thumb Hand Rule by Scott (1992) Model | 0.0005115146 | -308.704 | -306.99 | -308.08 |
| Gaussian KDE-Methods of Sheather & Jones (1991) via Direct Plug-in (DPI) Model | 0.0005394557 | -306.523 | -304.809 | -305.899 |
| Gaussian KDE-Methods of Sheather & Jones (1991) via Solve-the-Equation (STE) Model | 0.0005456173 | -306.057 | -304.344 | -305.433 |
| Gaussian KDE-Biased-cross validation (BCV) by Scott and Terrell (1987) Model | 0.00059716 | -302.356 | -300.643 | -301.732 |
| Gaussian KDE-Unbiased cross validation (UCV) by Scott and Terrell (1987) Model | 0.0005972232 | -302.352 | -300.638 | -301.728 |
| Gaussian KDE-Least Square Cross Validation (LSCV) Model | 0.0005962418 | -302.419 | -300.706 | -301.795 |
| Gaussian KDE-Normal Scale (NS) Model | 0.0005745812 | -303.937 | -302.223 | -303.313 |
| Gaussian KDE-Smoothed cross-validation (SCV) model | 0.0005854346 | -303.169 | -301.456 | -302.545 |

| (f-10) WGS (Time lag = ±3 days)  (Regina) | Mean Square Error (MSE) | Akaike Information Criterion (AIC) | Bayesian Information Criterion (BIC) | Hannan-Quinn Information Criterion (HQC) |
| --- | --- | --- | --- | --- |
| Gamma-2P/LMOM | 0.0005302792 | -305.226 | -301.799 | -303.978 |
| GEV-3P/LMOM | 0.0005171858 | -304.251 | -299.111 | -302.379 |
| Normal-2P/LMOM | 0.0006241373 | -298.545 | -295.118 | -297.297 |
| NORMAL-2P/MLE | 0.0006309214 | -298.101 | -294.674 | -296.854 |
| LOGISTIC-2P/MLE | 0.0006706743 | -295.596 | -292.169 | -294.348 |
| GEV-3P/MLE | 0.001865204 | -251.66 | -246.519 | -249.788 |
| Gaussian KDE-Silverman Rule-of-thumb (ROT) Model | 0.0003521335 | -324.012 | -322.298 | -323.388 |
| Gaussian KDE-Thumb Hand Rule by Scott (1992) Model | 0.0004001296 | -318.773 | -317.059 | -318.149 |
| Gaussian KDE-Methods of Sheather & Jones (1991) via Direct Plug-in (DPI) Model | 0.000426021 | -316.202 | -314.488 | -315.578 |
| Gaussian KDE-Methods of Sheather & Jones (1991) via Solve-the-Equation (STE) Model | 0.0004297271 | -315.847 | -314.133 | -315.223 |
| Gaussian KDE-Biased-cross validation (BCV) by Scott and Terrell (1987) Model | 0.0004221407 | -316.577 | -314.863 | -315.953 |
| Gaussian KDE-Unbiased cross validation (UCV) by Scott and Terrell (1987) Model | 0.0004223531 | -316.556 | -314.843 | -315.932 |
| Gaussian KDE-Least Square Cross Validation (LSCV) Model | 0.0004213431 | -316.655 | -314.941 | -316.031 |
| Gaussian KDE-Normal Scale (NS) Model | 0.0003999118 | -318.795 | -317.081 | -318.171 |
| Gaussian KDE-Smoothed cross-validation (SCV) model | 0.0004365002 | -315.206 | -313.492 | -314.582 |

| (g-1) AMWGS (km/hr) EDMONTON | Mean Square Error (MSE) | Akaike Information Criterion (AIC) | Bayesian Information Criterion (BIC) | Hannan-Quinn Information Criterion (HQC) |
| --- | --- | --- | --- | --- |
| Gamma-2P/LMOM | 0.0007552551 | -261.973 | -258.751 | -260.837 |
| GEV-3P/LMOM | 0.0006822619 | -263.734 | -258.901 | -262.03 |
| Normal-2P/LMOM | 0.0009754521 | -252.507 | -249.285 | -251.371 |
| NORMAL-2P/MLE | 0.001298589 | -241.92 | -238.698 | -240.784 |
| LOGISTIC-2P/MLE | 0.001382541 | -239.602 | -236.38 | -238.466 |
| GEV-3P/MLE | 0.004273085 | -195.851 | -191.018 | -194.147 |
| Gaussian KDE-Silverman Rule-of-thumb (ROT) Model | 0.0003423414 | -293.249 | -291.638 | -292.681 |
| Gaussian KDE-Thumb Hand Rule by Scott (1992) Model | 0.0003736586 | -290.01 | -288.399 | -289.442 |
| Gaussian KDE-Methods of Sheather & Jones (1991) via Direct Plug-in (DPI) Model | 0.0004044834 | -287.077 | -285.466 | -286.509 |
| Gaussian KDE-Methods of Sheather & Jones (1991) via Solve-the-Equation (STE) Model | 0.0004090136 | -286.665 | -285.054 | -286.097 |
| Gaussian KDE-Biased-cross validation (BCV) by Scott and Terrell (1987) Model | 0.0003915315 | -288.281 | -286.671 | -287.714 |
| Gaussian KDE-Unbiased cross validation (UCV) by Scott and Terrell (1987) Model | 0.0003915515 | -288.28 | -286.669 | -287.712 |
| Gaussian KDE-Least Square Cross Validation (LSCV) Model | 0.0003911995 | -288.313 | -286.702 | -287.745 |
| Gaussian KDE-Normal Scale (NS) Model | 0.0003734947 | -290.026 | -288.416 | -289.459 |
| Gaussian KDE-Smoothed cross-validation (SCV) model | 0.0004206651 | -285.626 | -284.015 | -285.058 |

| (g-2) MT (Time lag = 0 day) EDMONTON | Mean Square Error (MSE) | Akaike Information Criterion (AIC) | Bayesian Information Criterion (BIC) | Hannan-Quinn Information Criterion (HQC) |
| --- | --- | --- | --- | --- |
| Gamma-2P/LMOM | 0.005726277 | -187.02 | -183.798 | -185.884 |
| GEV-3P/LMOM | 0.0007735718 | -259.086 | -254.253 | -257.382 |
| Normal-2P/LMOM | 0.003476472 | -205.484 | -202.262 | -204.348 |
| NORMAL-2P/MLE | 0.003544466 | -204.768 | -201.546 | -203.632 |
| LOGISTIC-2P/MLE | 0.002185937 | -222.651 | -219.429 | -221.515 |
| GEV-3P/MLE | 0.06914207 | -92.8489 | -88.0161 | -91.1451 |
| Gaussian KDE-Silverman Rule-of-thumb (ROT) Model* | 0.0003697261 | -290.402 | -288.791 | -289.834 |
| Gaussian KDE-Thumb Hand Rule by Scott (1992) Model | 0.0004256534 | -285.19 | -283.579 | -284.622 |
| Gaussian KDE-Methods of Sheather & Jones (1991) via Direct Plug-in (DPI) Model | 0.0003810555 | -289.285 | -287.674 | -288.717 |
| Gaussian KDE-Methods of Sheather & Jones (1991) via Solve-the-Equation (STE) Model | 0.0003718141 | -290.193 | -288.582 | -289.625 |
| Gaussian KDE-Biased-cross validation (BCV) by Scott and Terrell (1987) Model | 0.0005136688 | -278.235 | -276.625 | -277.668 |
| Gaussian KDE-Unbiased cross validation (UCV) by Scott and Terrell (1987) Model | 0.0004636336 | -282.027 | -280.416 | -281.459 |
| Gaussian KDE-Least Square Cross Validation (LSCV) Model | 0.0004624806 | -282.12 | -280.509 | -281.552 |
| Gaussian KDE-Normal Scale (NS) Model | 0.0004708388 | -281.457 | -279.846 | -280.889 |
| Gaussian KDE-Smoothed cross-validation (SCV) model | 0.0004022387 | -287.283 | -285.672 | -286.715 |

| (g-3) MT (Time lag = ±1 days) $℃$ EDMONTON | Mean Square Error (MSE) | Akaike Information Criterion (AIC) | Bayesian Information Criterion (BIC) | Hannan-Quinn Information Criterion (HQC) |
| --- | --- | --- | --- | --- |
| Gamma-2P/LMOM | 0.00327024 | -207.747 | -204.525 | -206.611 |
| GEV-3P/LMOM | 0.0006848892 | -263.591 | -258.759 | -261.888 |
| Normal-2P/LMOM | 0.002174788 | -222.84 | -219.619 | -221.705 |
| NORMAL-2P/MLE | 0.002251937 | -221.551 | -218.329 | -220.415 |
| LOGISTIC-2P/MLE | 0.0015245 | -235.985 | -232.763 | -234.849 |
| GEV-3P/MLE | 0.0127149 | -155.504 | -150.672 | -153.801 |
| Gaussian KDE-Silverman Rule-of-thumb (ROT) Model | 0.0006506702 | -269.488 | -267.877 | -268.92 |
| Gaussian KDE-Thumb Hand Rule by Scott (1992) Model | 0.0007190854 | -265.789 | -264.178 | -265.221 |
| Gaussian KDE-Methods of Sheather & Jones (1991) via Direct Plug-in (DPI) Model | 0.0007175198 | -265.869 | -264.258 | -265.301 |
| Gaussian KDE-Methods of Sheather & Jones (1991) via Solve-the-Equation (STE) Model | 0.0007171375 | -265.889 | -264.278 | -265.321 |
| Gaussian KDE-Biased-cross validation (BCV) by Scott and Terrell (1987) Model | 0.0007438476 | -264.536 | -262.925 | -263.968 |
| Gaussian KDE-Unbiased cross validation (UCV) by Scott and Terrell (1987) Model | 0.0007445073 | -264.503 | -262.892 | -263.935 |
| Gaussian KDE-Least Square Cross Validation (LSCV) Model | 0.0007448526 | -264.486 | -262.875 | -263.918 |
| Gaussian KDE-Normal Scale (NS) Model | 0.0007188115 | -265.803 | -264.192 | -265.235 |
| Gaussian KDE-Smoothed cross-validation (SCV) model | 0.0007303829 | -265.212 | -263.601 | -264.644 |

Supplementary Table ST 6: Model performance evaluation of the fitted 2-D copulas via CvM functional statistics S_n_ with parametric bootstrapping procedure together with estimating copula dependence parameter via MPL estimator (a) Montreal (b) Quebec City (c) Ottawa (d) Toronto (e) Vancouver (f) Regina (g) Edmonton

| (a-1) | | | | | |
| --- | --- | --- | --- | --- | --- |
| 2-D Copula function (Montreal) | Estimated copula dependence parameter via Maximum pseudo likelihood (MPL) estimation (Annual maximum WGS-Corresponding highest MT (Time lag = 0 day) | Estimated copula dependence parameter via Maximum pseudo likelihood (MPL) estimation (Annual maximum WGS-Corresponding highest MT (Time lag =$\pm1$days) | Estimated copula dependence parameter via Maximum pseudo likelihood (MPL) estimation (Annual maximum WGS-Corresponding highest MT (Time lag =$\pm2$days) | Estimated copula dependence parameter via Maximum pseudo likelihood (MPL) estimation (Annual maximum WGS-Corresponding highest MT (Time lag =$\pm3$days) | Estimated copula dependence parameter via Maximum pseudo likelihood (MPL) estimation (Annual highest MT-Corresponding maximum WGS (Time lag = 0 day) |
| Normal copula | 0.2827 | rho.1  0.2256 | rho.1  0.2835 | rho.1  0.1592 | 0.2969 |
| Frank copula | 1.341 | alpha  0.9547 | alpha  1.246 | alpha  0.8319 | 1.325 |
| Clayton | 0.4155 | alpha  0.4657 | alpha  0.55 | alpha  0.1927 | 0.5527 |
| Gumbel | 1.167 | alpha  1.088 | alpha  1.118 | alpha  1.054 | 1.1 |
| Joe | 1.197 | alpha  1.014 | alpha  1.049 | alpha  1.028 | 2 |
| Plackett copula | 1.936 | alpha  1.649 | alpha  1.835 | alpha  1.456 | alpha  1.736 |
| AMH Copula | 0.5935 | alpha  0.6486 | alpha  0.999 | alpha  0.4009 | alpha  0.7793 |
| BB1 copula | theta delta  0.4155 1.0000 | theta delta  0.4656 1.0000 | theta delta  0.55 1.00 | theta delta  0.1928 1.0000 | theta delta  0.5527 1.0000 |
| BB6 copula | theta delta  1.000 1.167 | theta delta  1.0 1.1 | theta delta  1.000 1.118 | theta delta  1.000 1.054 | theta delta  1.0 1.1 |
| BB7 copula | theta delta  1.0000 0.4155 | theta delta  1.0000 0.4657 | theta delta  1.00 0.55 | theta delta  1.0000 0.2001 | theta delta  1.0000 0.5528 |
| BB8 copula | par = 6, par2 = 0.22 | par = 6, par2 = 0.15 | par = 6, par2 = 0.19 | par = 6, par2 = 0.14 | par = 6, par2 = 0.2 |
| Survival Clayton (Rotated Clayton 180 degrees) | theta  0.2917 | theta  0.09977 | theta  0.1703 | theta  0.1335 | theta  0.1055 |
| Survival Gumbel (Rotated Clayton 180 degrees) | theta  1.21 | theta  1.211 | theta  1.248 | theta  1.084 | theta  1.224 |
| Survival Joe (Rotated Joe 180 degrees) | theta  1.317 | theta  1.383 | theta  1.443 | theta  1.112 | theta  1.411 |
| Survival BB1 (Rotated BB1 180 degrees) | theta delta  3.145e-08 1.210e+00 | theta delta  4.419e-10 1.211e+00 | theta delta  8.763e-09 1.248e+00 | theta delta  0.006003 1.081442 | theta delta  1.025e-08 1.224e+00 |
| Survival BB6 (Rotated BB6 180 degrees) | theta delta  1.192 1.077 | theta delta  1.383 1.000 | theta delta  1.443 1.000 | theta delta  1.000 1.084 | theta delta  1.411 1.000 |
| Survival BB7 (Rotated BB7 180 degrees) | theta delta  1.2789 0.1165 | theta delta  1.383e+00 2.020e-09 | theta delta  1.443e+00 5.269e-08 | theta delta  1.06453 0.08614 | theta delta  1.411e+00 2.287e-08 |
| Survival BB8 (Rotated BB8 180 degrees) | theta delta  1.317 1.000 | par = 1.38, par2 = 1 | par = 1.44, par2 = 1 | par = 2.33, par2 = 0.44 | theta delta  1.411 1.000 |
| Tawn type-1 copula | par = 2.47, par2 = 0.3 | par = 1.84, par2 = 0.19, | par = 5.31, par2 = 0.1 | par = 5.3, par2 = 0.1 | param1 param2  1.9652 0.1 |
| Tawn type-2 copula | par = 2.95, par2 = 0.06 | par = 20, par2 = 0.05 | par = 20, par2 = 0.05 | par = 12.43, par2 = 0.05 | param1 param2  1.82 0.18 |
| Survival Tawn type 1 copula (180 degrees rotation) | par = 1.19, par2 = 0.36 | par = 1.28, par2 = 0.3 | par = 1.38, par2 = 0.33, | par = 2, par2 = 0.5 | param1 param2  2.7198 0.2155 |
| Survival Tawn type 2 copula (180 degrees rotation) | par = 2.21, par2 = 0.35 | par = 1.54, par2 = 0.3 | par = 1.55, par2 = 0.33 | par = 11.93, par2 = 0.1 | param1 param2  4.13 0.19 |

| (a-2) | | | | | |
| --- | --- | --- | --- | --- | --- |
| 2-D Copula function (Montreal) | Cramer von Mises (CvM) functional test statistics $\boldsymbol{S}_{\boldsymbol{n}}$ (estimated p-value≥0.05) with parametric bootstrap procedure, (No. of Bootstrapping samples, N=1000)  (Annual maximum WGS-Corresponding highest MT (Time lag = 0 day) | Cramer von Mises (CvM) functional test statistics $\boldsymbol{S}_{\boldsymbol{n}}$ (estimated p-value≥0.05) with parametric bootstrap procedure, (No. of Bootstrapping samples, N=1000)  (Annual maximum WGS-Corresponding highest MT (Time lag = ±1 days) | Cramer von Mises (CvM) functional test statistics $\boldsymbol{S}_{\boldsymbol{n}}$ (estimated p-value≥0.05) with parametric bootstrap procedure, (No. of Bootstrapping samples, N=1000)  (Annual maximum WGS-Corresponding highest MT (Time lag = ±2 days) | Cramer von Mises (CvM) functional test statistics $\boldsymbol{S}_{\boldsymbol{n}}$ (estimated p-value≥0.05) with parametric bootstrap procedure, (No. of Bootstrapping samples, N=1000)  (Annual maximum WGS-Corresponding highest MT (Time lag = ±3 days) | Cramer von Mises (CvM) functional test statistics $\boldsymbol{S}_{\boldsymbol{n}}$ (estimated p-value≥0.05) with parametric bootstrap procedure, (No. of Bootstrapping samples, N=1000)  (Annual highest MT-Corresponding maximum WGS (Time lag = 0 day) |
| Normal copula | statistic = 0.040041 | statistic = 0.023299 | statistic = 0.022393 | statistic = 0.027989 | statistic = 0.025314 |
| Frank copula | statistic = 0.04801 | statistic = 0.029062 | statistic = 0.029028 | statistic = 0.029978 | statistic = 0.033124 |
| Clayton | statistic = 0.048052 | statistic = 0.021769 | statistic = 0.01978 | statistic = 0.03153 | statistic = 0.01835 |
| Gumbel | statistic = 0.047582 | statistic = 0.033758 | statistic = 0.035433 | statistic = 0.039724k | statistic = 0.04587 |
| Joe | statistic = 0.060762 | statistic = 0.054523 | statistic = 0.05752 | statistic = 0.050453 | statistic = 0.049719 |
| Plackett copula | statistic = 0.048038 | statistic = 0.028099 | statistic = 0.029333 | statistic = 0.031719 | statistic = 0.037267 |
| AMH Copula | statistic = 0.046908 | statistic = 0.022252 | statistic = 0.021264 | statistic = 0.028423 | statistic = 0.020466 |
| BB1 copula | statistic = 0.048052 | statistic = 0.021769 | statistic = 0.01973 | statistic = 0.031525 | statistic = 0.018346 |
| BB6 copula | statistic = 0.047626 | statistic = 0.031664 | statistic = 0.0354 | statistic = 0.039647 | statistic = 0.045831 |
| BB7 copula | statistic = 0.048052 | statistic = 0.021769 | statistic = 0.019726 | statistic = 0.030926 | statistic = 0.018344 |
| BB8 copula | statistic = 0.048814 | statistic = 0.03771 | statistic = 0.031945 | statistic = 0.0312 | statistic = 0.036635 |
| Survival Clayton (Rotated Clayton 180 degrees) | statistic = 0.051999 | statistic = 0.042265 | statistic = 0.042414 | statistic = 0.036751 | statistic = 0.057458 |
| Survival Gumbel (Rotated Clayton 180 degrees) | statistic = 0.044871 | statistic = 0.020972 | statistic = 0.020049 | statistic = 0.033481 | statistic = 0.023248 |
| Survival Joe (Rotated Joe 180 degrees) | statistic = 0.053429 | statistic = 0.022253 | statistic = 0.020438 | statistic = 0.038035 | statistic = 0.020736 |
| Survival BB1 (Rotated BB1 180 degrees) | statistic = 0.044871 | statistic = 0.020972 | statistic = 0.020049 | statistic = 0.033302 | statistic = 0.023248 |
| Survival BB6 (Rotated BB6 180 degrees) | statistic = 0.04936 | statistic = 0.022253 | statistic = 0.020438 | statistic = 0.033481 | statistic = 0.020736 |
| Survival BB7 (Rotated BB7 180 degrees) | statistic = 0.044168 | statistic = 0.022253 | statistic = 0.020438 | statistic = 0.033948 | statistic = 0.020736 |
| Survival BB8 (Rotated BB8 180 degrees) | statistic = 0.053429 | statistic = 0.022281 | statistic = 0.020489 | statistic = 0.029517 | statistic = 0.020736 |
| Tawn type-1 copula | statistic = 0.01648 | statistic = 0.020733 | statistic = 0.034789 | statistic = 0.024966 | statistic = 0.051693 |
| Tawn type-2 copula | statistic = 0.075113 | statistic = 0.039734 | statistic = 0.048461 | statistic = 0.037755 | statistic = 0.043257 |
| Survival Tawn type 1 copula (180 degrees rotation) | statistic = 0.072639 | statistic = 0.031004 | statistic = 0.029149 | statistic = 0.032173 | statistic = 0.023098 |
| Survival Tawn type 2 copula (180 degrees rotation) | statistic = 0.05023 | statistic = 0.026211 | statistic = 0.02539 | statistic = 0.037309 | statistic = 0.024056 |

| (b-1) | | | | |
| --- | --- | --- | --- | --- |
| 2D -Copula function Quebec City) | Estimated dependence parameter via Maximum pseudo likelihood (MPL) estimation (annual maximum WGS- corresponding highest MT (Time lag = 0 day) | Estimated dependence parameter via Maximum pseudo likelihood (MPL) estimation (annual maximum WGS- corresponding highest MT (Time lag = ±1days) | Estimated dependence parameter via Maximum pseudo likelihood (MPL) estimation [annual maximum WGS- corresponding highest MT (Time lag = ±2days)] | Estimated dependence parameter via Maximum pseudo likelihood [(MPL) estimation (annual maximum WGS- corresponding highest MT (Time lag = ±3days)] |
| Normal copula | rho.1  0.3657 | rho.1  0.3035 | rho.1  0.2746 | rho.1  0.3087 |
| Frank copula | alpha  2.173 | alpha  1.672 | alpha  1.531 | alpha  1.731 |
| Clayton | alpha  0.3949 | alpha  0.2596 | alpha  0.2312 | alpha  0.3012 |
| Gumbel | alpha  1.306 | alpha  1.241 | alpha  1.21 | alpha  1.233 |
| Joe | alpha  1.483 | alpha  1.402 | alpha  1.348 | alpha  1.37 |
| Plackett copula | alpha  2.614 | alpha  2.114 | alpha  1.966 | alpha  2.126 |
| AMH Copula | alpha  0.6245 | alpha  0.5096 | alpha  0.4821 | alpha  0.5428 |
| BB1 copula | theta delta  2.894e-09 1.306e+00 | theta delta  1.742e-09 1.241e+00 | theta delta  1.753e-09 1.210e+00 | theta delta  1.098e-09 1.233e+00 |
| BB6 copula | theta delta  1.078 1.247 | theta delta  1.401 1.000 | theta delta  1.297 1.027 | theta delta  1.048 1.198 |
| BB7 copula | theta delta  1.483e+00 6.879e-08 | theta delta  1.402e+00 5.772e-08 | theta delta  1.348e+00 8.899e-09 | theta delta  1.370e+00 1.103e-08 |
| BB8 copula | theta delta  3.2811 0.5902 | par = 1.99, par2 = 0.8 | par = 2.15, par2 = 0.71 | par = 2.69, par2 = 0.61 |
| Survival Clayton (Rotated Clayton 180 degrees) | theta  0.5709 | theta  0.4853 | theta  0.4264 | theta  0.4613 |
| Survival Gumbel (Rotated Clayton 180 degrees) | theta  1.234 | theta  1.156 | theta  1.129 | theta  1.172 |
| Survival Joe (Rotated Joe 180 degrees) | theta  1.251 | theta  1.114 | theta  1.081 | theta  1.166 |
| Survival BB1 (Rotated BB1 180 degrees) | theta delta  0.5709 1.0000 | theta delta  0.4853 1.0000 | theta delta  0.4262 1.0000 | theta delta  0.4613 1.0000 |
| Survival BB6 (Rotated BB6 180 degrees) | theta delta  1.000 1.234 | theta delta  1.000 1.156 | theta delta  1.000 1.129 | theta delta  1.000 1.172 |
| Survival BB7 (Rotated BB7 180 degrees) | theta delta  1.0000 0.5709 | theta delta  1.0000 0.4853 | theta delta  1.0000 0.4262 | theta delta  1.0000 0.4613 |
| Survival BB8 (Rotated BB8 180 degrees) | par = 6, par2 = 0.32 | par = 6, par2 = 0.25 | par = 6, par2 = 0.23 | par = 6, par2 = 0.26 |
| Tawn type-1 copula | par = 1.44, par2 = 0.44 | par = 1.29, par2 = 0.4 | par = 1.22, par2 = 0.39 | par = 1.23, par2 = 0.4 |
| Tawn type-2 copula | par = 1.49, par2 = 0.44 | par = 1.54, par2 = 0.4 | par = 1.65, par2 = 0.37 | par = 1.81, par2 = 0.35 |
| Survival Tawn type 1 copula (180 degrees rotation) | par = 2.47, par2 = 0.14 | par = 7.87, par2 = 0.1 | par = 10.45, par2 = 0.11 | par = 2.57, par2 = 0.15 |
| Survival Tawn type 2 copula (180 degrees rotation) | par = 1.28, par2 = 0.44 | par = 1.12, par2 = 0.4 | par = 1.15, par2 = 0.09 | par = 1.2, par2 = 0.1 |

| (b-2) | | | | |
| --- | --- | --- | --- | --- |
| 2-D Copula function (QUEBEC CITY) | Cramer von Mises functional test statistics $S_{n}$ (estimated p-value≥0.05) with parametric bootstrap procedure, (No. of Bootstrapping samples, N=1000)  (Annual maximum WGS- Corresponding highest MT (Time lag = 0 day) | Cramer von Mises functional test statistics $S_{n}$ (estimated p-value≥0.05) with parametric bootstrap procedure, (No. of Bootstrapping samples, N=1000)  (Annual maximum WGS- Corresponding highest MT (Time lag = ±1days) | Cramer von Mises functional test statistics $S_{n}$ (estimated p-value≥0.05) with parametric bootstrap procedure, (No. of Bootstrapping samples, N=1000)  [Annual maximum WGS- Corresponding highest MT (Time lag = ±2days)] | Cramer von Mises functional test statistics $S_{n}$ (estimated p-value≥0.05) with parametric bootstrap procedure, (No. of Bootstrapping samples, N=1000)  (Annual maximum WGS- Corresponding highest MT (Time lag = ±3days)] |
| Normal copula | statistic = 0.033219 | statistic = 0.037969 | statistic = 0.042644 | statistic = 0.031193 |
| Frank copula | statistic = 0.033199 | statistic = 0.040737 | statistic = 0.044246 | statistic = 0.0332 |
| Clayton | statistic = 0.06237 | statistic = 0.06736 | statistic = 0.069594 | statistic = 0.054522 |
| Gumbel | statistic = 0.029526 | statistic = 0.034543 | statistic = 0.040083 | statistic = 0.030108 |
| Joe | statistic = 0.03221 | statistic = 0.0348 | statistic = 0.040593 | statistic = 0.032237 |
| Plackett copula | statistic = 0.03804 | statistic = 0.044924 | statistic = 0.048945 | statistic = 0.037956 |
| AMH Copula | statistic = 0.053709 | statistic = 0.056863 | statistic = 0.058488 | statistic = 0.046529 |
| BB1 copula | statistic = 0.029489 | statistic = 0.034493 | statistic = 0.040023 | statistic = 0.030057 |
| BB6 copula | statistic = 0.029356 | statistic = 0.034852 | statistic = 0.040147 | statistic = 0.030024 |
| BB7 copula | statistic = 0.032202 | statistic = 0.034754 | statistic = 0.040558 | statistic = 0.032199 |
| BB8 copula | statistic = 0.028416 | statistic = 0.033128 | statistic = 0.038587 | statistic = 0.029501 |
| Survival Clayton (Rotated Clayton 180 degrees) | statistic = 0.030261 | statistic = 0.03244 | statistic = 0.037795 | statistic = 0.028980 |
| Survival Gumbel (Rotated Clayton 180 degrees) | statistic = 0.051707 | statistic = 0.059145 | statistic = 0.064481 | statistic = 0.047958 |
| Survival Joe (Rotated Joe 180 degrees) | statistic = 0.076126 | statistic = 0.086363 | statistic = 0.090343 | statistic = 0.068008 |
| Survival BB1 (Rotated BB1 180 degrees) | statistic = 0.030261 | statistic = 0.032436 | statistic = 0.037813 | statistic = 0.028985 |
| Survival BB6 (Rotated BB6 180 degrees) | statistic = 0.051707 | statistic = 0.059145 | statistic = 0.064481 | statistic = 0.047958 |
| Survival BB7 (Rotated BB7 180 degrees) | statistic = 0.030261 | statistic = 0.032441 | statistic = 0.037813 | statistic = 0.028976 |
| Survival BB8 (Rotated BB8 180 degrees) | statistic = 0.037819 | statistic = 0.046015 | statistic = 0.04964 | statistic = 0.037569 |
| Tawn type-1 copula | statistic = 0.045311 | statistic = 0.055844 | statistic = 0.064332 | statistic = 0.05501 |
| Tawn type-2 copula | statistic = 0.041863 | statistic = 0.038458 | statistic = 0.039554 | statistic = 0.031936 |
| Survival Tawn type 1 copula (180 degrees rotation) | statistic = 0.073411 | statistic = 0.07095 | statistic = 0.064613 | statistic = 0.049559 |
| Survival Tawn type 2 copula (180 degrees rotation) | statistic = 0.067152 | statistic = 0.082663 | statistic = 0.094791 | statistic = 0.082258 |

| (c-1) | | | | |
| --- | --- | --- | --- | --- |
| 2D -Copula function  EVSG-1 (Ottawa) | Estimated dependence parameter via Maximum pseudo likelihood (MPL) estimation (annual maximum WGS- corresponding highest MT (Time lag = 0 day) | Estimated dependence parameter via Maximum pseudo likelihood (MPL) estimation (annual maximum WGS- corresponding highest MT (Time lag = ±1days) | Estimated dependence parameter via Maximum pseudo likelihood (MPL) estimation [annual maximum WGS- corresponding highest MT (Time lag = ±2days)] | Estimated dependence parameter via Maximum pseudo likelihood [(MPL) estimation (annual maximum WGS- corresponding highest MT (Time lag = ±3days)] |
| Normal copula | rho.1  0.128 | rho.1  0.2118 | rho.1  0.1926 | rho.1  0.1485 |
| Frank copula | alpha  0.6848 | alpha  1.17 | alpha  0.9813 | alpha  0.7642 |
| Clayton | alpha  0.1365 | alpha  9.002e-18 | alpha  2.386e-17 | alpha  3.15e-18 |
| Gumbel | alpha  1.044 | alpha  1.178 | alpha  1.147 | alpha  1.099 |
| Joe | alpha  1.028 | alpha  1.32 | alpha  1.253 | alpha  1.158 |
| Plackett copula | alpha  1.353 | alpha  1.689 | alpha  1.552 | alpha  1.413 |
| AMH Copula | alpha  0.3294 | alpha  0.3702 | alpha  0.3499 | alpha  0.3135 |
| BB1 copula | theta delta  0.1375 1.0000 | theta delta  3.009e-10 1.178e+00 | theta delta  1.497e-09 1.147e+00 | theta delta  1.754e-08 1.099e+00 |
| BB6 copula | theta delta  1.000 1.044 | theta delta  1.32 1.00 | theta delta  1.253 1.000 | theta delta  1.158 1.000 |
| BB7 copula | theta delta  1.0000 0.1364 | theta delta  1.320e+00 1.099e-08 | theta delta  1.253e+00 2.631e-08 | theta delta  1.158e+00 1.514e-07 |
| BB8 copula | par = 6, par2 = 0.12 | par = 1.32, par2 = 1 | par = 1.25, par2 = 1 | par = 1.16, par2 = 1 |
| Survival Clayton (Rotated Clayton 180 degrees) | theta  0.1036 | theta  0.4019 | theta  0.3292 | theta  0.2134 |
| Survival Gumbel (Rotated Clayton 180 degrees) | theta  1.062 | theta  1.065 | theta  1.059 | theta  1.05 |
| Survival Joe (Rotated Joe 180 degrees) | theta  1.077 | theta  2 | theta  1 | theta  1.011 |
| Survival BB1 (Rotated BB1 180 degrees) | theta delta  0.01815 1.05461 | theta delta  0.4019 1.0000 | theta delta  0.3293 1.0000 | theta delta  0.2134 1.0000 |
| Survival BB6 (Rotated BB6 180 degrees) | theta delta  1.000 1.062 | theta delta  1.000 1.065 | theta delta  1.000 1.059 | theta delta  1.00 1.05 |
| Survival BB7 (Rotated BB7 180 degrees) | theta delta  1.04192 0.07288 | theta delta  1.0000 0.4019 | theta delta  1.0000 0.3297 | theta delta  1.0000 0.2134 |
| Survival BB8 (Rotated BB8 180 degrees) | par = 6, par2 = 0.12 | par = 6, par2 = 0.18 | par = 6, par2 = 0.16, | par = 6, par2 = 0.13 |
| Tawn type-1 copula | par = 1.56, par2 = 0.07 | par = 1.4, par2 = 0.33 | par = 1.33, par2 = 0.31 | par = 1.18, par2 = 0.29 |
| Tawn type-2 copula | par = 20, par2 = 0.02 | par = 1.29, par2 = 0.33 | par = 1.27, par2 = 0.31 | par = 1.24, par2 = 0.29 |
| Survival Tawn type 1 copula (180 degrees rotation) | par = 4.69, par2 = 0.08 | par = 1.77, par2 = 0.03 | par = 1.94, par2 = 0.08 | par = 1.74, par2 = 0.05 |
| Survival Tawn type 2 copula (180 degrees rotation) | par = 2, par2 = 0.5 | par = 1.48, par2 = 0.15 | par = 1.52, par2 = 0.12 | par = 1.45, par2 = 0.12 |

| (c-2) | | | | |
| --- | --- | --- | --- | --- |
| 2-D Copula function (Ottawa) | Cramer von Mises functional test statistics $S_{n}$ (estimated p-value≥0.05) with parametric bootstrap procedure, (No. of Bootstrapping samples, N=1000)  (Annual maximum WGS- Corresponding highest MT (Time lag = 0 day) | Cramer von Mises functional test statistics $S_{n}$ (estimated p-value≥0.05) with parametric bootstrap procedure, (No. of Bootstrapping samples, N=1000)  (Annual maximum WGS- Corresponding highest MT (Time lag = ±1days) | Cramer von Mises functional test statistics $S_{n}$ (estimated p-value≥0.05) with parametric bootstrap procedure, (No. of Bootstrapping samples, N=1000)  [Annual maximum WGS- Corresponding highest MT (Time lag = ±2days)] | Cramer von Mises functional test statistics $S_{n}$ (estimated p-value≥0.05) with parametric bootstrap procedure, (No. of Bootstrapping samples, N=1000)  (Annual maximum WGS- Corresponding highest MT (Time lag = ±3days)] |
| Normal copula | statistic = 0.019072 | statistic = 0.032485 | statistic = 0.02272 | statistic = 0.022096 |
| Frank copula | statistic = 0.020319 | statistic = 0.034157 | statistic = 0.025336 | statistic = 0.024003, |
| Clayton | statistic = 0.023774 | statistic = 31.471 | statistic = 31.576 | statistic = 31.646 |
| Gumbel | statistic = 0.027365 | statistic = 0.023577 | statistic = 0.018066 | statistic = 0.021558 |
| Joe | statistic = 0.034882 | statistic = 0.018246 | statistic = 0.015672 | statistic = 0.022348, |
| Plackett copula | statistic = 0.02193, | statistic = 0.036431 | statistic = 0.026993 | statistic = 0.025491 |
| AMH Copula | statistic = 0.019717 | statistic = 0.043752 | statistic = 0.030155 | statistic = 0.025993 |
| BB1 copula | statistic = 0.023691 | statistic = 0.023553 | statistic = 0.018071 | statistic = 0.021587 |
| BB6 copula | statistic = 0.02731 | statistic = 0.018262 | statistic = 0.015654 | statistic = 0.022367 |
| BB7 copula | statistic = 0.02378 | statistic = 0.018262 | statistic = 0.015654 | statistic = 0.022367 |
| BB8 copula | statistic = 0.020834 | statistic = 0.018262 | statistic = 0.015846 | statistic = 0.022177 |
| Survival Clayton (Rotated Clayton 180 degrees) | statistic = 0.025369 | statistic = 0.017652 | statistic = 0.014642 | statistic = 0.02016 |
| Survival Gumbel (Rotated Clayton 180 degrees) | statistic = 0.024467 | statistic = 0.054799 | statistic = 0.038392 | statistic = 0.033483 |
| Survival Joe (Rotated Joe 180 degrees) | statistic = 0.028685 | statistic = 0.083471 | statistic = 0.077515 | statistic = 0.04602 |
| Survival BB1 (Rotated BB1 180 degrees) | statistic = 0.023764 | statistic = 0.017641 | statistic = 0.014638 | statistic = 0.02014 |
| Survival BB6 (Rotated BB6 180 degrees) | statistic = 0.024467 | statistic = 0.054799 | statistic = 0.038392 | statistic = 0.033483 |
| Survival BB7 (Rotated BB7 180 degrees) | statistic = 0.023972 | statistic = 0.017660 | statistic = 0.014622 | statistic = 0.02017 |
| Survival BB8 (Rotated BB8 180 degrees) | statistic = 0.020923 | statistic = 0.037802 | statistic = 0.027111 | statistic = 0.025114 |
| Tawn type-1 copula | statistic = 0.025909 | statistic = 0.025238 | statistic = 0.020637 | statistic = 0.026471 |
| Tawn type-2 copula | statistic = 0.034213 | statistic = 0.031083 | statistic = 0.02222 | statistic = 0.023251 |
| Survival Tawn type 1 copula (180 degrees rotation) | statistic = 0.020955 | statistic = 0.0678 | statistic = 0.037218 | statistic = 0.0358 |
| Survival Tawn type 2 copula (180 degrees rotation) | statistic = 0.041124 | statistic = 0.051188 | statistic = 0.034788 | statistic = 0.028536 |

| (d-1) | | | | |
| --- | --- | --- | --- | --- |
| 2D -Copula function  (Toronto) | Estimated dependence parameter via Maximum pseudo likelihood (MPL) estimation (Annual highest MT-corresponding maximum WGS (Time lag = 0 day) | Estimated dependence parameter via Maximum pseudo likelihood (MPL) estimation (Annual highest MT-corresponding maximum WGS (Time lag = ±1days) | Estimated dependence parameter via Maximum pseudo likelihood (MPL) estimation [Annual highest MT-corresponding maximum WGS (Time lag = ±2days)] | Estimated dependence parameter via Maximum pseudo likelihood [(MPL) estimation (Annual highest MT-corresponding maximum WGS (Time lag = ±3days)] |
| Normal copula | rho.1  0.1843 | rho.1  0.1263 | rho.1  0.3424 | rho.1  0.3705 |
| Frank copula | alpha  1.042 | alpha  0.9081 | alpha  1.843 | alpha  1.751 |
| Clayton | alpha  0.2948 | alpha  0.1481 | alpha  0.3533 | alpha  0.4133 |
| Gumbel | alpha  1.16 | alpha  1.156 | alpha  1.303 | alpha  1.291 |
| Joe | alpha  1.238 | alpha  1.267 | alpha  1.49 | alpha  1.45 |
| Plackett copula | alpha  1.741 | alpha  1.667 | alpha  2.518 | alpha  2.301 |
| AMH Copula | alpha  0.4591 | alpha  0.3336 | alpha  0.5868 | alpha  0.6312 |
| BB1 copula | theta delta  0.06999 1.13390 | theta delta  3.865e-09 1.156e+00 | theta delta  9.531e-10 1.303e+00 | theta delta  0.00333 1.28943 |
| BB6 copula | theta delta  1.065 1.114 | theta delta  1.267 1.000 | theta delta  1.49 1.00 | theta delta  1.259 1.110 |
| BB7 copula | theta delta  1.1836 0.1648 | theta delta  1.283e+00 1.863e-09 | theta delta  1.489161 0.002697 | theta delta  1.3956 0.1703 |
| BB8 copula | theta delta  1.238 1.000 | theta delta  1.267 1.000 | theta delta  1.49 1.00 | theta delta  1.45 1.00 |
| Survival Clayton (Rotated Clayton 180 degrees) | theta  0.2471 | theta  0.2575 | theta  0.5818 | theta  0.5536 |
| Survival Gumbel (Rotated Clayton 180 degrees) | theta  1.161 | theta  1.104 | theta  1.226 | theta  1.24 |
| Survival Joe (Rotated Joe 180 degrees) | theta  1.255 | theta  1.127 | theta  1.252 | theta  1.297 |
| Survival BB1 (Rotated BB1 180 degrees) | theta delta  0.0271 1.1484 | theta delta  0.236 1.013 | theta delta  0.5818 1.0000 | theta delta  0.4703 1.0505 |
| Survival BB6 (Rotated BB6 180 degrees) | theta delta  1.054 1.124 | theta delta  1.0 1.1 | theta delta  1.000 1.226 | theta delta  1.000 1.225 |
| Survival BB7 (Rotated BB7 180 degrees) | theta delta  1.210 0.106 | theta delta  1.0261 0.2415 | theta delta  1.0000 0.5817 | theta delta  1.1110 0.4983 |
| Survival BB8 (Rotated BB8 180 degrees) | theta delta  1.7472 0.6995 | par = 6, par2 = 0.15 | par = 6, par2 = 0.28 | par = 6, par2 = 0.27, |
| Tawn type-1 copula | par = 1.46, par2 = 0.31 | par = 1.38, par2 = 0.3 | par = 1.4, par2 = 0.39 | par = 1.36, par2 = 0.39 |
| Tawn type-2 copula | par = 1.14, par2 = 0.31 | par = 1.13, par2 = 0.3, | par = 1.58, par2 = 0.39 | par = 6.36, par2 = 0.17 |
| Survival Tawn type 1 copula (180 degrees rotation) | par = 1.26, par2 = 0.31 | par = 1.16, par2 = 0.3 | par = 1.43, par2 = 0.39 | par = 3.25, par2 = 0.16 |
| Survival Tawn type 2 copula (180 degrees rotation) | par = 1.27, par2 = 0.31 | par = 1.19, par2 = 0.3 | par = 1.3, par2 = 0.39 | par = 1.3, par2 = 0.39 |

| (d-2) | | | | |
| --- | --- | --- | --- | --- |
| 2-D Copula function (Toronto) | Cramer von Mises functional test statistics $S_{n}$ (estimated p-value≥0.05) with parametric bootstrap procedure, (No. of Bootstrapping samples, N=1000)  (Annual maximum WGS- Corresponding highest MT (Time lag = 0 day) | Cramer von Mises functional test statistics $S_{n}$ (estimated p-value≥0.05) with parametric bootstrap procedure, (No. of Bootstrapping samples, N=1000)  (Annual maximum WGS- Corresponding highest MT (Time lag = ±1days) | Cramer von Mises functional test statistics $S_{n}$ (estimated p-value≥0.05) with parametric bootstrap procedure, (No. of Bootstrapping samples, N=1000)  [Annual maximum WGS- Corresponding highest MT (Time lag = ±2days)] | Cramer von Mises functional test statistics $S_{n}$ (estimated p-value≥0.05) with parametric bootstrap procedure, (No. of Bootstrapping samples, N=1000)  (Annual maximum WGS- Corresponding highest MT (Time lag = ±3days)] |
| Normal copula | statistic = 0.03014 | statistic = 0.039415 | statistic = 0.027586 | statistic = 0.027912 |
| Frank copula | statistic = 0.03133d | statistic = 0.034606 | statistic = 0.032173 | statistic = 0.035903 |
| Clayton | statistic = 0.030704 | statistic = 0.045854 | statistic = 0.049181 | statistic = 0.044804 |
| Gumbel | statistic = 0.025258 | statistic = 0.023394 | statistic = 0.01953 | statistic = 0.024295 |
| Joe | statistic = 0.027738 | statistic = 0.021117 | statistic = 0.017628 | statistic = 0.024087 |
| Plackett copula | statistic = 0.029864 | statistic = 0.031732 | statistic = 0.031063 | statistic = 0.036479 |
| AMH Copula | statistic = 0.031344 | statistic = 0.039789 | statistic = 0.043012 | statistic = 0.041462 |
| BB1 copula | statistic = 0.024191 | statistic = 0.023394 | statistic = 0.019559 | statistic = 0.024309 |
| BB6 copula | statistic = 0.025516 | statistic = 0.02116 | statistic = 0.01763 | statistic = 0.023482 |
| BB7 copula | statistic = 0.022619 | statistic = 0.019789 | statistic = 0.017596 | statistic = 0.022375 |
| BB8 copula | statistic = 0.027725 | statistic = 0.02116 | statistic = 0.01763 | statistic = 0.024067 |
| Survival Clayton (Rotated Clayton 180 degrees) | statistic = 0.030164, | statistic = 0.026616 | statistic = 0.017779 | statistic = 0.023298 |
| Survival Gumbel (Rotated Clayton 180 degrees) | statistic = 0.027965 | statistic = 0.03874 | statistic = 0.039021 | statistic = 0.038011 |
| Survival Joe (Rotated Joe 180 degrees) | statistic = 0.031482 | statistic = 0.046901 | statistic = 0.055638 | statistic = 0.05036 |
| Survival BB1 (Rotated BB1 180 degrees) | statistic = 0.027173 | statistic = 0.026587 | statistic = 0.017779 | statistic = 0.022994 |
| Survival BB6 (Rotated BB6 180 degrees) | statistic = 0.028515 | statistic = 0.039431 | statistic = 0.039021 | statistic = 0.039354 |
| Survival BB7 (Rotated BB7 180 degrees) | statistic = 0.026212 | statistic = 0.026349 | statistic = 0.017784 | statistic = 0.022633 |
| Survival BB8 (Rotated BB8 180 degrees) | statistic = 0.031691 | statistic = 0.036603 | statistic = 0.0353 | statistic = 0.038274 |
| Tawn type-1 copula | statistic = 0.021076 | statistic = 0.024621 | statistic = 0.034116 | statistic = 0.039461 |
| Tawn type-2 copula | statistic = 0.040364 | statistic = 0.043488 | statistic = 0.026157 | statistic = 0.044621 |
| Survival Tawn type 1 copula (180 degrees rotation) | statistic = 0.035987 | statistic = 0.045029 | statistic = 0.042649 | statistic = 0.045066 |
| Survival Tawn type 2 copula (180 degrees rotation) | statistic = 0.035526 | statistic = 0.042684 | statistic = 0.050078 | statistic = 0.048146 |

| (e-1) | | | | |
| --- | --- | --- | --- | --- |
| 2D -Copula function  (Vancouver) | Estimated dependence parameter via Maximum pseudo likelihood (MPL) estimation (Annual highest MT-corresponding maximum WGS (Time lag = 0 day) | Estimated dependence parameter via Maximum pseudo likelihood (MPL) estimation (Annual highest MT-corresponding maximum WGS (Time lag = ±1days) | Estimated dependence parameter via Maximum pseudo likelihood (MPL) estimation [Annual highest MT-corresponding maximum WGS (Time lag = ±2days)] | Estimated dependence parameter via Maximum pseudo likelihood [(MPL) estimation (Annual highest MT-corresponding maximum WGS (Time lag = ±3days)] |
| Normal copula | rho.1  0.2535 | rho.1  0.32 | rho.1  0.2435 | rho.1  0.1349 |
| Frank copula | alpha  1.333 | alpha  2.105 | alpha  1.453 | alpha  0.7466 |
| Clayton | alpha  0.3995 | alpha  0.6053 | alpha  0.3657 | alpha  0.2674 |
| Gumbel | alpha  1.072 | alpha  1.255 | alpha  1.191 | alpha  1.078 |
| Joe | alpha  1.051 | alpha  1.336 | alpha  1.273 | alpha  1.077 |
| Plackett copula | alpha  1.775 | alpha  3.123 | alpha  2.104 | alpha  1.468 |
| AMH Copula | alpha  0.6698 | alpha  0.7451 | alpha  0.5444 | alpha  0.4243 |
| BB1 copula | theta delta  0.3994 1.0000 | theta delta  0.2966 1.1545 | theta delta  0.02884 1.18015 | theta delta  0.2674 1.0000 |
| BB6 copula | theta delta  1.000 1.072 | theta delta  1.000 1.255 | theta delta  1.000 1.191 | theta delta  1.000 1.078 |
| BB7 copula | theta delta  1.0000 0.3994 | theta delta  1.1668 0.4648 | theta delta  1.2032 0.1888 | theta delta  1.0000 0.2674 |
| BB8 copula | par = 6, par2 = 0.21 | par = 6, par2 = 0.32 | par = 2.1, par2 = 0.68 | par = 6, par2 = 0.13 |
| Survival Clayton (Rotated Clayton 180 degrees) | theta  0.1386 | theta  0.4089 | theta  0.3144 | theta  0.0623 |
| Survival Gumbel (Rotated Clayton 180 degrees) | theta  1.176 | theta  1.337 | theta  1.205 | theta  1.123 |
| Survival Joe (Rotated Joe 180 degrees) | theta  1.245 | theta  1.546 | theta  1.309 | theta  1.225 |
| Survival BB1 (Rotated BB1 180 degrees) | theta delta  1.485e-08 1.176e+00 | theta delta  1.340e-08 1.337e+00 | theta delta  0.07906 1.16418 | theta delta  2.231e-08 1.123e+00 |
| Survival BB6 (Rotated BB6 180 degrees) | theta delta  1.000 1.176 | theta delta  1.000 1.337 | theta delta  1.0 1.2 | theta delta  1.225 1.000 |
| Survival BB7 (Rotated BB7 180 degrees) | theta delta  1.20602 0.07435 | theta delta  1.4362 0.1693 | theta delta  1.1940 0.1995 | theta delta  1.225e+00 1.699e-07 |
| Survival BB8 (Rotated BB8 180 degrees) | theta delta  2.6539 0.5719 | theta delta  3.9002 0.5029 | par = 1.17, par2 = 0.36 | theta delta  1.225 1.000 |
| Tawn type-1 copula | par = 2.84, par2 = 0.06 | par = 1.2, par2 = 0.43 | par = 1.18, par2 = 0.36 | par = 1, par2 = 0 |
| Tawn type-2 copula | par = 20, par2 = 0.02 | par = 1.84, par2 = 0.43 | par = 1.57, par2 = 0.36 | par = 1.21, par2 = 0.27 |
| Survival Tawn type 1 copula (180 degrees rotation) | par = 1.18, par2 = 0.32 | par = 2, par2 = 0.43 | par = 1.84, par2 = 0.36 | par = 1.35, par2 = 0.27 |
| Survival Tawn type 2 copula (180 degrees rotation) | par = 2.53, par2 = 0.09 | par = 1.28, par2 = 0.43 | par = 1.17, par2 = 0.36 | par = 1.17, par2 = 0.27 |

| (e-2) | | | | |
| --- | --- | --- | --- | --- |
| 2-D Copula function (Vancouver) | Cramer von Mises functional test statistics $S_{n}$ (estimated p-value≥0.05) with parametric bootstrap procedure, (No. of Bootstrapping samples, N=1000)  (Annual highest MT-corresponding maximum WGS(Time lag = 0 day) | Cramer von Mises functional test statistics $S_{n}$ (estimated p-value≥0.05) with parametric bootstrap procedure, (No. of Bootstrapping samples, N=1000)  (Annual highest MT-corresponding maximum WGS(Time lag = ±1days) | Cramer von Mises functional test statistics $S_{n}$ (estimated p-value≥0.05) with parametric bootstrap procedure, (No. of Bootstrapping samples, N=1000)  [Annual highest MT-corresponding maximum WGS(Time lag = ±2days)] | Cramer von Mises functional test statistics $S_{n}$ (estimated p-value≥0.05) with parametric bootstrap procedure, (No. of Bootstrapping samples, N=1000)  (Annual highest MT-corresponding maximum WGS(Time lag = ±3days)] |
| Normal copula | statistic = 0.012939 | statistic = 0.047429 | statistic = 0.027257 | statistic = 0.025295 |
| Frank copula | statistic = 0.017529 | statistic = 0.035957 | statistic = 0.025854 | statistic = 0.025895 |
| Clayton | statistic = 0.023747 | statistic = 0.047006 | statistic = 0.032603 | statistic = 0.018574 |
| Gumbel | statistic = 0.031329 | statistic = 0.048564 | statistic = 0.025416 | statistic = 0.029354 |
| Joe | statistic = 0.044894 | statistic = 0.072149 | statistic = 0.033634 | statistic = 0.036512 |
| Plackett copula | statistic = 0.02127 | statistic = 0.029401 | statistic = 0.024459 | statistic = 0.025463 |
| AMH Copula | statistic = 0.015673 | statistic = 0.044881 | statistic = 0.030545 | statistic = 0.021785 |
| BB1 copula | statistic = 0.023749 | statistic = 0.036384 | statistic = 0.024401 | statistic = 0.018575 |
| BB6 copula | statistic = 0.031267 | statistic = 0.048586 | statistic = 0.025361 | statistic = 0.029312 |
| BB7 copula | statistic = 0.023749 | statistic = 0.038414 | statistic = 0.023224 | statistic = 0.018575 |
| BB8 copula | statistic = 0.018655 | statistic = 0.037722 | statistic = 0.025902 | statistic = 0.026642 |
| Survival Clayton (Rotated Clayton 180 degrees) | statistic = 0.029975 | statistic = 0.065104 | statistic = 0.032709 | statistic = 0.039284 |
| Survival Gumbel (Rotated Clayton 180 degrees) | statistic = 0.021538 | statistic = 0.036602 | statistic = 0.026999 | statistic = 0.020281 |
| Survival Joe (Rotated Joe 180 degrees) | statistic = 0.033889 | statistic = 0.048982 | statistic = 0.035475 | statistic = 0.018838 |
| Survival BB1 (Rotated BB1 180 degrees) | statistic = 0.021538 | statistic = 0.036602 | statistic = 0.024617 | statistic = 0.020281 |
| Survival BB6 (Rotated BB6 180 degrees) | statistic = 0.021538 | statistic = 0.036602 | statistic = 0.02763 | statistic = 0.018838 |
| Survival BB7 (Rotated BB7 180 degrees) | statistic = 0.024821 | statistic = 0.039906 | statistic = 0.024564 | statistic = 0.018838 |
| Survival BB8 (Rotated BB8 180 degrees) | statistic = 0.016702 | statistic = 0.034358 | statistic = 0.094095 | statistic = 0.018838 |
| Tawn type-1 copula | statistic = 0.026472 | statistic = 0.11126 | statistic = 0.026904 | statistic = 0.059999 |
| Tawn type-2 copula | statistic = 0.047507 | statistic = 0.042585 | statistic = 0.028069 | statistic = 0.02909 |
| Survival Tawn type 1 copula (180 degrees rotation) | statistic = 0.041148 | statistic = 0.024086 | statistic = 0.016107 | statistic = 0.020035 |
| Survival Tawn type 2 copula (180 degrees rotation) | statistic = 0.04699 | statistic = 0.090316 | statistic = 0.053655 | statistic = 0.027568 |

| (f-1) | | | | |
| --- | --- | --- | --- | --- |
| 2D -Copula function  (Regina) | Estimated dependence parameter via Maximum pseudo likelihood (MPL) estimation (Annual maximum WGS-Corresponding highest MT Time lag = 0 day) | Estimated dependence parameter via Maximum pseudo likelihood (MPL) estimation (Annual maximum WGS-Corresponding highest MT Time lag ±1days) | Estimated dependence parameter via Maximum pseudo likelihood (MPL) estimation (Annual maximum WGS-Corresponding highest MT Time lag ±2 days) | Estimated dependence parameter via Maximum pseudo likelihood (MPL) estimation [Annual maximum WGS-Corresponding highest MT Time lag ±3 days) |
| Normal copula | rho.1  0.3494 | rho.1  0.1987 | rho.1  0.2024 | rho.1  0.1948 |
| Frank copula | alpha  2.227 | alpha  1.471 | alpha  1.218 | alpha  1.063 |
| Clayton | par = 0.3 | par = 0.18 | alpha  0.3312 | par = 0.27 |
| Gumbel | alpha  1.324 | alpha  1.198 | alpha  1.115 | alpha  1.086 |
| Joe | alpha  1.537 | alpha  1.323 | alpha  1.097 | alpha  1.058 |
| Plackett copula | alpha  3.034 | alpha  2.288 | alpha  1.913 | alpha  1.674 |
| AMH Copula | alpha  0.5777 | alpha  0.4639 | alpha  0.5614 | alpha  0.4756 |
| BB1 copula | theta delta  2.242e-08 1.324e+00 | theta delta  5.676e-08 1.198e+00 | theta delta  0.3312 1.0000 | theta delta  0.2697 1.0000 |
| BB6 copula | theta delta  1.537 1.000 | theta delta  1.323 1.000 | theta delta  1.000 1.115 | theta delta  1.000 1.086 |
| BB7 copula | theta delta  1.537e+00 1.992e-08 | theta delta  1.3234923 0.0000001 | theta delta  1.0000 0.3317 | theta delta  1.0000 0.2693 |
| BB8 copula | theta delta  1.9696 0.8956 | theta delta  1.7615 0.8372 | par = 6, par2 = 0.2 | par = 6, par2 = 0.18 |
| Survival Clayton (Rotated Clayton 180 degrees) | theta  0.6356 | theta  0.3742 | theta  0.1728 | theta  0.1771 |
| Survival Gumbel (Rotated Clayton 180 degrees) | theta  1.235 | theta  1.152 | theta  1.164 | theta  1.136 |
| Survival Joe (Rotated Joe 180 degrees) | theta  1.204 | theta  1.158 | theta  1.259 | theta  1.206 |
| Survival BB1 (Rotated BB1 180 degrees) | theta delta  0.6356 1.0000 | theta delta  0.3742 1.0000 | theta delta  4.950e-08 1.164e+00 | theta delta  8.319e-08 1.136e+00 |
| Survival BB6 (Rotated BB6 180 degrees) | theta delta  1.000 1.235 | theta delta  1.000 1.152 | theta delta  1.218 1.024 | theta delta  1.023 1.120 |
| Survival BB7 (Rotated BB7 180 degrees) | theta delta  1.0000 0.6356 | theta delta  1.000 0.374 | theta delta  1.259e+00 3.495e-07 | theta delta  1.19488 0.02683 |
| Survival BB8 (Rotated BB8 180 degrees) | par = 6, par2 = 0.31 | par = 6, par2 = 0.23 | par = 1.55, par2 = 0.87 | par = 1.73, par2 = 0.72 |
| Tawn type-1 copula | par = 1.53, par2 = 0.43 | par = 1.15, par2 = 0.36 | par = 1, par2 = 0.04 | par = 3.34, par2 = 0.05 |
| Tawn type-2 copula | par = 1.43, par2 = 0.43 | par = 1.62, par2 = 0.33, | par = 6.92, par2 = 0.09 | par = 7.97, par2 = 0.07 |
| Survival Tawn type 1 copula (180 degrees rotation) | par = 1.36, par2 = 0.43 | par = 1.34, par2 = 0.36, | par = 1.57, par2 = 0.26 | par = 1.96, par2 = 0.2 |
| Survival Tawn type 2 copula (180 degrees rotation) | par = 1.32, par2 = 0.4 | par = 1.16, par2 = 0.36 | par = 1.19, par2 = 0.34 | par = 11.39, par2 = 0.04 |

| (f-2) | | | | |
| --- | --- | --- | --- | --- |
| 2-D Copula function (Regina region ) | Cramer von Mises functional test statistics $S_{n}$ (estimated p-value≥0.05) with parametric bootstrap procedure, (No. of Bootstrapping samples, N=1000)  (Annual maximum WGS-Corresponding highest MT Time lag= 0 days) | Cramer von Mises functional test statistics $S_{n}$ (estimated p-value≥0.05) with parametric bootstrap procedure, (No. of Bootstrapping samples, N=1000)  (Annual maximum WGS-Corresponding highest MT Time lag ±1 days)) | Cramer von Mises functional test statistics $S_{n}$ (estimated p-value≥0.05) with parametric bootstrap procedure, (No. of Bootstrapping samples, N=1000)  (Annual maximum WGS-Corresponding highest MT Time lag ±2 days)) | Cramer von Mises functional test statistics $S_{n}$ (estimated p-value≥0.05) with parametric bootstrap procedure, (No. of Bootstrapping samples, N=1000)  [Annual maximum WGS-Corresponding highest MT Time lag ±3 days) |
| Normal copula | statistic = 0.039899 | statistic = 0.038989 | statistic = 0.030031 | statistic = 0.025633 |
| Frank copula | statistic = 0.036172 | statistic = 0.028801 | statistic = 0.028126 | statistic = 0.02653 |
| Clayton | statistic = 21.459 | statistic = 22.047 | statistic = 0.029933 | statistic = 0.029317 |
| Gumbel | statistic = 0.028214 | statistic = 0.024828 | statistic = 0.036437 | statistic = 0.03616 |
| Joe | statistic = 0.027171 | statistic = 0.025837 | statistic = 0.054191 | statistic = 0.051744 |
| Plackett copula | statistic = 0.034365 | statistic = 0.023803 | statistic = 0.0257 | statistic = 0.027039, |
| AMH Copula | statistic = 0.064564 | statistic = 0.042159 | statistic = 0.025967 | statistic = 0.026237 |
| BB1 copula | statistic = 0.028198 | statistic = 0.024853 | statistic = 0.029934 | statistic = 0.029314 |
| BB6 copula | statistic = 0.027161 | statistic = 0.025891 | statistic = 0.036517 | statistic = 0.036236 |
| BB7 copula | statistic = 0.027161 | statistic = 0.025837 | statistic = 0.029897 | statistic = 0.029343 |
| BB8 copula | statistic = 0.023989 | statistic = 0.022162 | statistic = 0.029342 | statistic = 0.02712 |
| Survival Clayton (Rotated Clayton 180 degrees) | statistic = 0.025664 | statistic = 0.025629 | statistic = 0.044219 | statistic = 0.035515 |
| Survival Gumbel (Rotated Clayton 180 degrees) | statistic = 0.057235 | statistic = 0.041064 | statistic = 0.028613 | statistic = 0.027959 |
| Survival Joe (Rotated Joe 180 degrees) | statistic = 0.092364 | statistic = 0.059669 | statistic = 0.033737 | statistic = 0.032815 |
| Survival BB1 (Rotated BB1 180 degrees) | statistic = 0.025664 | statistic = 0.025629 | statistic = 0.028613 | statistic = 0.027959 |
| Survival BB6 (Rotated BB6 180 degrees) | statistic = 0.057235 | statistic = 0.041064 | statistic = 0.032639 | statistic = 0.028318 |
| Survival BB7 (Rotated BB7 180 degrees) | statistic = 0.025664 | statistic = 0.025649 | statistic = 0.033737 | statistic = 0.030976 |
| Survival BB8 (Rotated BB8 180 degrees) | statistic = 0.044344 | statistic = 0.032146 | statistic = 0.028263 | statistic = 0.027206 |
| Tawn type-1 copula | statistic = 0.039963 | statistic = 0.058129 | statistic = 0.078083 | statistic = 0.050239 |
| Tawn type-2 copula | statistic = 0.047962 | statistic = 0.025394 | statistic = 0.053341 | statistic = 0.045596 |
| Survival Tawn type 1 copula (180 degrees rotation) | statistic = 0.068419 | statistic = 0.040203 | statistic = 0.026317 | statistic = 0.022948 |
| Survival Tawn type 2 copula (180 degrees rotation) | statistic = 0.075036 | statistic = 0.059336 | statistic = 0.04326 | statistic = 0.0508 |

| (f-3) | | | | |
| --- | --- | --- | --- | --- |
| 2D -Copula function  (Regina) | Estimated dependence parameter via Maximum pseudo likelihood (MPL) estimation (Annual highest MT-Corresponding maximum WGS Time lag = 0 day) | Estimated dependence parameter via Maximum pseudo likelihood (MPL) estimation (Annual highest MT-Corresponding maximum WGS Time lag ±1days) | Estimated dependence parameter via Maximum pseudo likelihood (MPL) estimation (Annual highest MT-Corresponding maximum WGS Time lag ±2 days) | Estimated dependence parameter via Maximum pseudo likelihood (MPL) estimation [Annual highest MT-Corresponding maximum WGS Time lag = ±3 days) |
| Normal copula | rho.1  0.3104 | rho.1  0.2073 | rho.1  0.239 | rho.1  0.1679 |
| Frank copula | alpha  1.548 | alpha  1.284 | alpha  1.156 | alpha  0.7022 |
| Clayton | alpha  0.4549 | alpha  0.3858 | alpha  0.3671 | alpha  0.3138 |
| Gumbel | alpha  1.192 | alpha  1.107 | alpha  1.092 | alpha  1.022 |
| Joe | alpha  1.24 | alpha  1.044 | alpha  1.017 | alpha  1 |
| Plackett copula | alpha  2.013 | alpha  1.833 | alpha  1.647 | alpha  1.371 |
| AMH Copula | alpha  0.6411 | alpha  0.6405 | alpha  0.6066 | alpha  0.5085 |
| BB1 copula | theta delta  0.4158 1.0230 | theta delta  0.386 1.000 | theta delta  0.3671 1.0000 | theta delta  0.3139 1.0000 |
| BB6 copula | theta delta  1.0 1.2 | theta delta  1.000 1.107 | heta delta  1.0 1.1 | theta delta  1.000 1.022 |
| BB7 copula | theta delta  1.0323 0.4365 | theta delta  1.0000 0.3858 | theta delta  1.0000 0.3671 | theta delta  1.0000 0.3138 |
| BB8 copula | par = 6, par2 = 0.25 | par = 6, par2 = 0.2 | par = 6, par2 = 0.18 | par = 6, par2 = 0.11 |
| Survival Clayton (Rotated Clayton 180 degrees) | theta  0.3418 | theta  0.1132 | theta  0.1106 | par = 0 |
| Survival Gumbel (Rotated Clayton 180 degrees) | theta  1.228 | theta  1.178 | theta  1.154 | theta  1.117 |
| Survival Joe (Rotated Joe 180 degrees) | theta  1.347 | theta  1.305 | theta  1.259 | theta  1.229 |
| Survival BB1 (Rotated BB1 180 degrees) | theta delta  0.02476 1.21692 | theta delta  7.967e-09 1.178e+00 | theta delta  2.317e-08 1.154e+00 | theta delta  3.106e-08 1.117e+00 |
| Survival BB6 (Rotated BB6 180 degrees) | theta delta  1.001 1.228 | theta delta  1.305 1.000 | theta delta  1.262 1.000 | theta delta  1.229 1.000 |
| Survival BB7 (Rotated BB7 180 degrees) | theta delta  1.2793 0.1646 | theta delta  1.289e+00 4.352e-08 | theta delta  1.259e+00 4.235e-08 | theta delta  1.229e+00 3.249e-08 |
| Survival BB8 (Rotated BB8 180 degrees) | par = 4.48, par2 = 0.34 | par = 1.6, par2 = 0.88 | par = 1.33, par2 = 0.94 | par = 1.33, par2 = 0.94 |
| Tawn type-1 copula | par = 1.77, par2 = 0.25 | par = 5.07, par2 = 0.05 | (par = 20, par2 = 0.04 | par = 4.29, par2 = 0.05 |
| Tawn type-2 copula | par = 1.25, par2 = 0.38 | par = 1, par2 = 0.05 | par = 1.24, par2 = 0.28 | par = 20, par2 = 0.03 |
| Survival Tawn type 1 copula (180 degrees rotation) | par = 1.71, par2 = 0.32 | par = 1.17, par2 = 0.35 | par = 20, par2 = 0.11 | par = 6.85, par2 = 0.12 |
| Survival Tawn type 2 copula (180 degrees rotation) | par = 1.35, par2 = 0.38 | par = 19.16, par2 = 0.11 | par = 1.89, par2 = 0.09 | par = 20, par2 = 0.05 |

| (f-4) | | | | |
| --- | --- | --- | --- | --- |
| 2-D Copula function (Regina) | Cramer von Mises functional test statistics $S_{n}$ (estimated p-value≥0.05) with parametric bootstrap procedure, (No. of Bootstrapping samples, N=1000)  (Annual highest MT-Corresponding maximum WGS Time lag= 0 days) | Cramer von Mises functional test statistics $S_{n}$ (estimated p-value≥0.05) with parametric bootstrap procedure, (No. of Bootstrapping samples, N=1000)  (Annual highest MT-Corresponding maximum WGS Time lag ±1 days)) | Cramer von Mises functional test statistics $S_{n}$ (estimated p-value≥0.05) with parametric bootstrap procedure, (No. of Bootstrapping samples, N=1000)  (Annual highest MT-Corresponding maximum WGS Time lag ±2 days)) | Cramer von Mises functional test statistics $S_{n}$ (estimated p-value≥0.05) with parametric bootstrap procedure, (No. of Bootstrapping samples, N=1000)  [Annual highest MT-Corresponding maximum WGS Time lag ±3 days) |
| Normal copula | statistic = 0.022522 | statistic = 0.023125 | statistic = 0.01692 | statistic = 0.018718 |
| Frank copula | statistic = 0.029829 | statistic = 0.022036 | statistic = 0.02201 | statistic = 0.022937 |
| Clayton | statistic = 0.026087 | statistic = 0.01893 | statistic = 0.018562 | statistic = 0.015534 |
| Gumbel | statistic = 0.030579 | statistic = 0.031557 | statistic = 0.030027 | statistic = 0.032674 |
| Joe | statistic = 0.042332 | statistic = 0.053401 | statistic = 0.052052 | statistic = 0.037835 |
| Plackett copula | statistic = 0.032605 | statistic = 0.023172 | statistic = 0.025006 | statistic = 0.02398 |
| AMH Copula | statistic = 0.027977 | statistic = 0.016749 | statistic = 0.0171 | statistic = 0.017342 |
| BB1 copula | statistic = 0.024514 | statistic = 0.018918 | statistic = 0.018563 | statistic = 0.015532 |
| BB6 copula | statistic = 0.029349 | statistic = 0.03156 | statistic = 0.028487 | statistic = 0.032712 |
| BB7 copula | statistic = 0.024368 | statistic = 0.018928 | statistic = 0.018563 | statistic = 0.015535 |
| BB8 copula | statistic = 0.030815 | statistic = 0.024545 | statistic = 0.024414 | statistic = 0.02461 |
| Survival Clayton (Rotated Clayton 180 degrees) | statistic = 0.035582 | statistic = 0.043945 | statistic = 0.038418 | statistic = 0.041097 |
| Survival Gumbel (Rotated Clayton 180 degrees) | statistic = 0.025182 | statistic = 0.019997 | statistic = 0.020814 | statistic = 0.018252 |
| Survival Joe (Rotated Joe 180 degrees) | statistic = 0.030448 | statistic = 0.021888 | statistic = 0.022869 | statistic = 0.016909 |
| Survival BB1 (Rotated BB1 180 degrees) | statistic = 0.024509 | statistic = 0.019997 | statistic = 0.020814 | statistic = 0.018252 |
| Survival BB6 (Rotated BB6 180 degrees) | statistic = 0.025098 | statistic = 0.021571 | statistic = 0.022672 | statistic = 0.016909 |
| Survival BB7 (Rotated BB7 180 degrees) | statistic = 0.023057 | statistic = 0.022614 | statistic = 0.022869 | statistic = 0.016909 |
| Survival BB8 (Rotated BB8 180 degrees) | statistic = 0.02959 | statistic = 0.018812 | statistic = 0.024656 | statistic = 0.017341 |
| Tawn type-1 copula | statistic = 0.03136 | statistic = 0.043703 | statistic = 0.043094 | statistic = 0.028456 |
| Tawn type-2 copula | statistic = 0.043603 | statistic = 0.06283 | statistic = 0.029807 | statistic = 0.031479 |
| Survival Tawn type 1 copula (180 degrees rotation) | statistic = 0.0272 | statistic = 0.037523 | statistic = 0.024766 | statistic = 0.014593 |
| Survival Tawn type 2 copula (180 degrees rotation) | statistic = 0.035481 | statistic = 0.038885 | statistic = 0.032125 | statistic = 0.025115 |

| (g-1) | | |
| --- | --- | --- |
| 2D -Copula function  (Edmonton) | Estimated dependence parameter via Maximum pseudo likelihood (MPL) estimation (Annual maximum WGS-corresponding highest MT(Time lag = 0 day) | Estimated dependence parameter via Maximum pseudo likelihood (MPL) estimation (Annual maximum WGS-corresponding highest MT(Time lag = ±1 days) |
| Normal copula | rho.1  0.1225 | rho.1  0.09559 |
| Frank copula | alpha  0.8923 | alpha  0.7434 |
| Clayton | alpha  0.2704 | alpha  0.3832 |
| Gumbel | alpha  1.107 | alpha  1.048 |
| Joe | alpha  1.12 | alpha  1 |
| Plackett copula | alpha  1.676 | alpha  1.591 |
| AMH Copula | alpha  0.4003 | alpha  0.4505 |
| BB1 copula | theta delta  0.2704 1.0000 | theta delta  0.3832 1.0000 |
| BB6 copula | theta delta  1.000 1.107 | theta delta  1.000 1.048 |
| BB7 copula | theta delta  1.0000 0.2704 | theta delta  1.0000 0.3833 |
| BB8 copula | par = 6, par2 = 0.16 | par = 6, par2 = 0.12 |
| Survival Clayton (Rotated Clayton 180 degrees) | theta  0.06209 | par = 1 |
| Survival Gumbel (Rotated Clayton 180 degrees) | theta  1.136 | theta  1.176 |
| Survival Joe (Rotated Joe 180 degrees) | theta  1.225 | theta  1.345 |
| Survival BB1 (Rotated BB1 180 degrees) | theta delta  3.458e-09 1.136e+00 | par = 0, par2 = 1.18 |
| Survival BB6 (Rotated BB6 180 degrees) | theta delta  1.225 1.000 | par = 1.34, par2 = 1 |
| Survival BB7 (Rotated BB7 180 degrees) | theta delta  1.225e+00 4.086e-08 | par = 1.34, par2 = 0 |
| Survival BB8 (Rotated BB8 180 degrees) | theta delta  1.225 1.000 | ar = 1.34, par2 = 1 |
| Tawn type-1 copula | par = 1, par2 = 0.01 | par = 1.13, par2 = 0.28 |
| Tawn type-2 copula | par = 1.14, par2 = 0.31 | par = 1, par2 = 0 |
| Survival Tawn type 1 copula (180 degrees rotation) | par = 1.15, par2 = 0.31 | par = 1.16, par2 = 0.28 |
| Survival Tawn type 2 copula (180 degrees rotation) | par = 1.36, par2 = 0.31, | par = 1.57, par2 = 0.28 |

| (g-2)) | | |
| --- | --- | --- |
| 2-D Copula function (Edmonton) | Cramer von Mises functional test statistics $S_{n}$ (estimated p-value≥0.05) with parametric bootstrap procedure, (No. of Bootstrapping samples, N=1000)  ((Annual maximum WGS-corresponding highest MT (Time lag = 0 day) | Cramer von Mises functional test statistics $S_{n}$ (estimated p-value≥0.05) with parametric bootstrap procedure, (No. of Bootstrapping samples, N=1000)  (Annual maximum WGS-corresponding highest MT (Time lag = ±1 days) |
| Normal copula | statistic = 0.041574 | statistic = 0.032085 |
| Frank copula | statistic = 0.03605 | statistic = 0.027565 |
| Clayton | statistic = 0.037351 | statistic = 0.017311 |
| Gumbel |  | statistic = 0.035624 |
| Joe | statistic = 0.041445 |  |
| Plackett copula | statistic = 0.032241 | statistic = 0.02384 |
| AMH Copula | statistic = 0.03693 | statistic = 0.022343 |
| BB1 copula | statistic = 0.037353 | statistic = 0.017311 |
| BB6 copula | statistic = 0.033427 | statistic = 0.035626 |
| BB7 copula | statistic = 0.037353 | statistic = 0.017309 |
| BB8 copula | statistic = 0.035391 | statistic = 0.029456 |
| Survival Clayton (Rotated Clayton 180 degrees) | statistic = 0.055141 | statistic = 0.038629, |
| Survival Gumbel (Rotated Clayton 180 degrees) | statistic = 0.034495 | statistic = 0.017197 |
| Survival Joe (Rotated Joe 180 degrees) | statistic = 0.039466 | statistic = 0.017094 |
| Survival BB1 (Rotated BB1 180 degrees) | statistic = 0.034495 | statistic = 0.016951 |
| Survival BB6 (Rotated BB6 180 degrees) | statistic = 0.039466 | statistic = 0.017199 |
| Survival BB7 (Rotated BB7 180 degrees) | statistic = 0.039466 | statistic = 0.017199 |
| Survival BB8 (Rotated BB8 180 degrees) | statistic = 0.039466 | statistic = 0.017199 |
| Tawn type-1 copula | statistic = 0.067974 | statistic = 0.033173 |
| Tawn type-2 copula | statistic = 0.044436 | statistic = 0.025546 |
| Survival Tawn type 1 copula (180 degrees rotation) | statistic = 0.046948 | statistic = 0.032094 |
| Survival Tawn type 2 copula (180 degrees rotation) | statistic = 0.033631 | statistic = 0.021428 |

**Supplementary Figure (SF)**


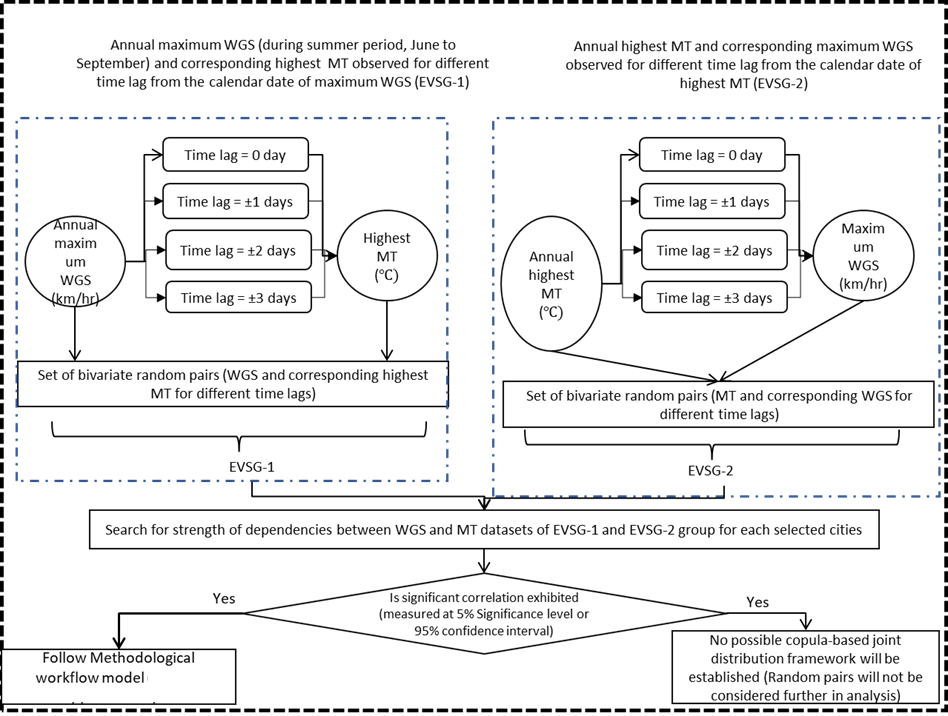


**Supplementary SF1**: Flowchart in the delineation of extreme value sample groups (EVSG) for defining bivariate extreme pairs in compounded WGS and MT events


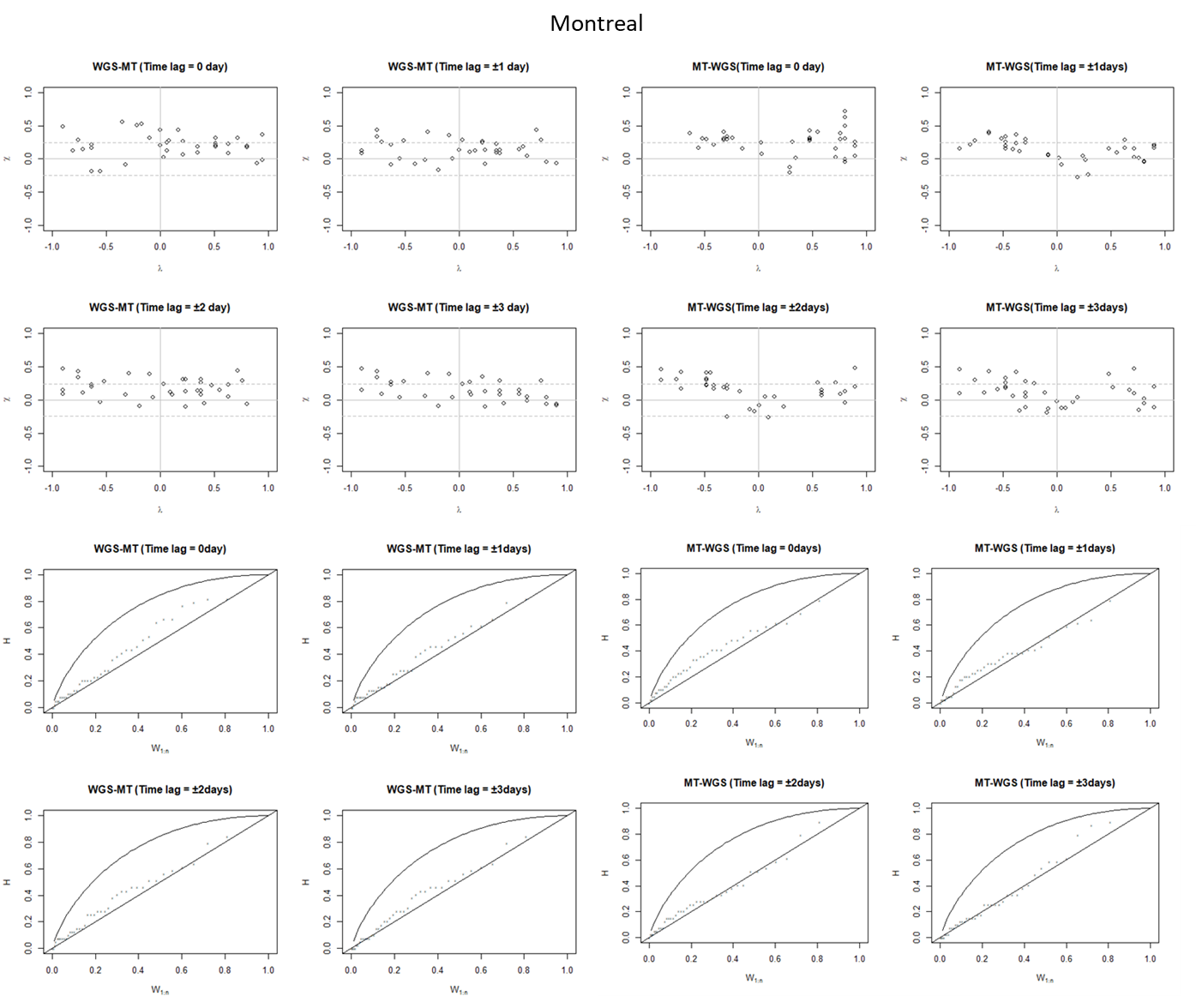
 (a-1)


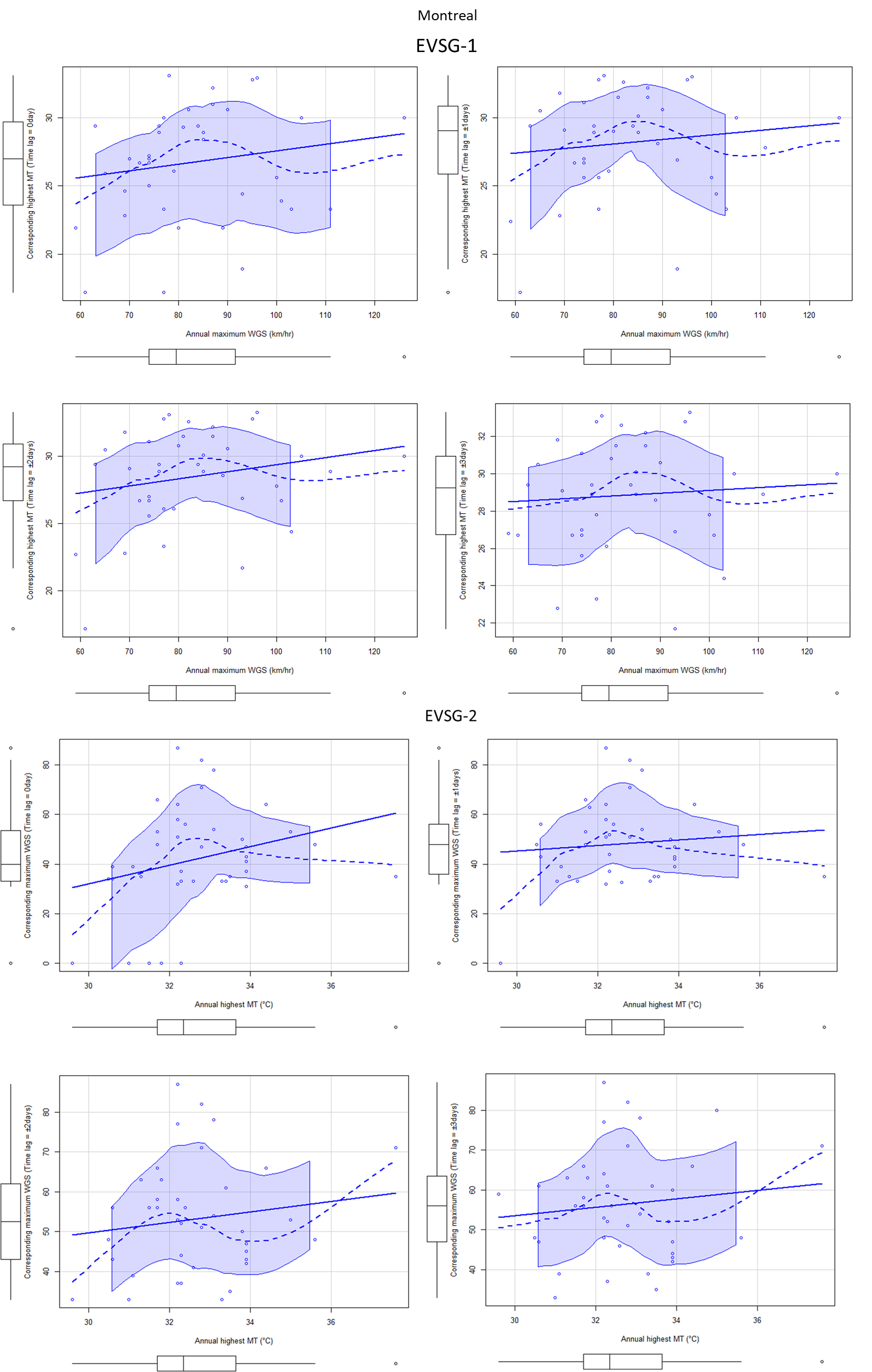
 (a-2)


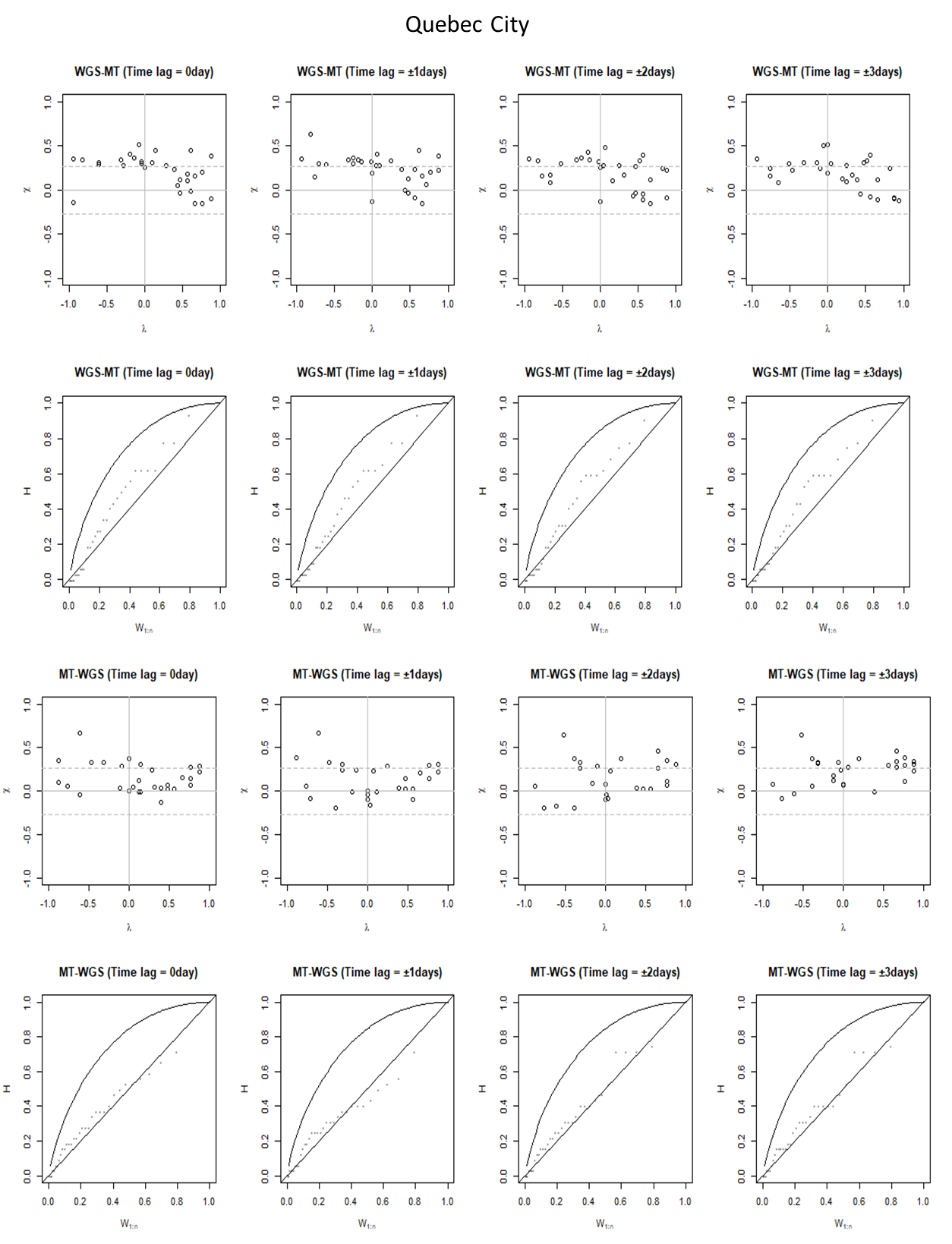
(b-1)


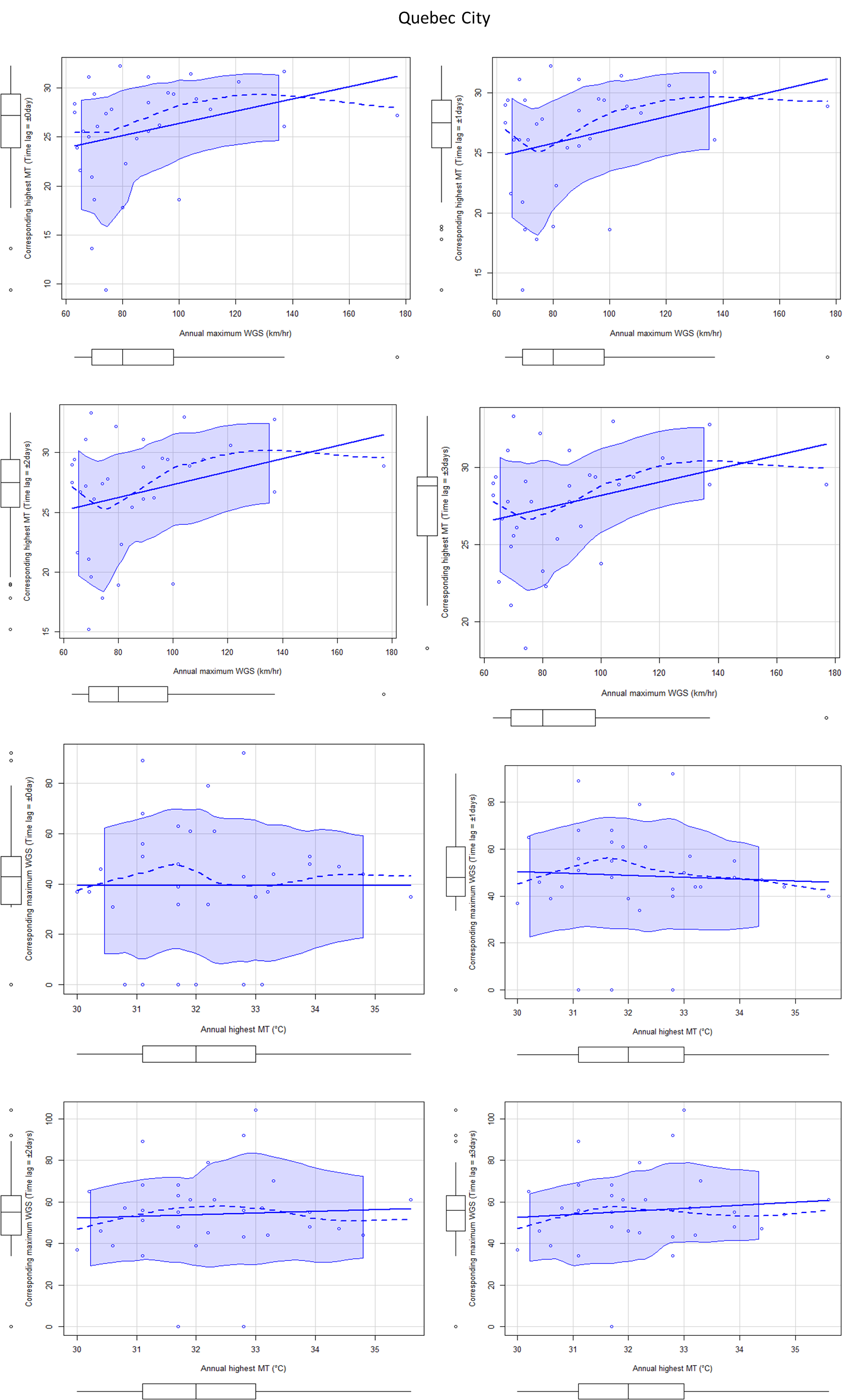
(b-2)


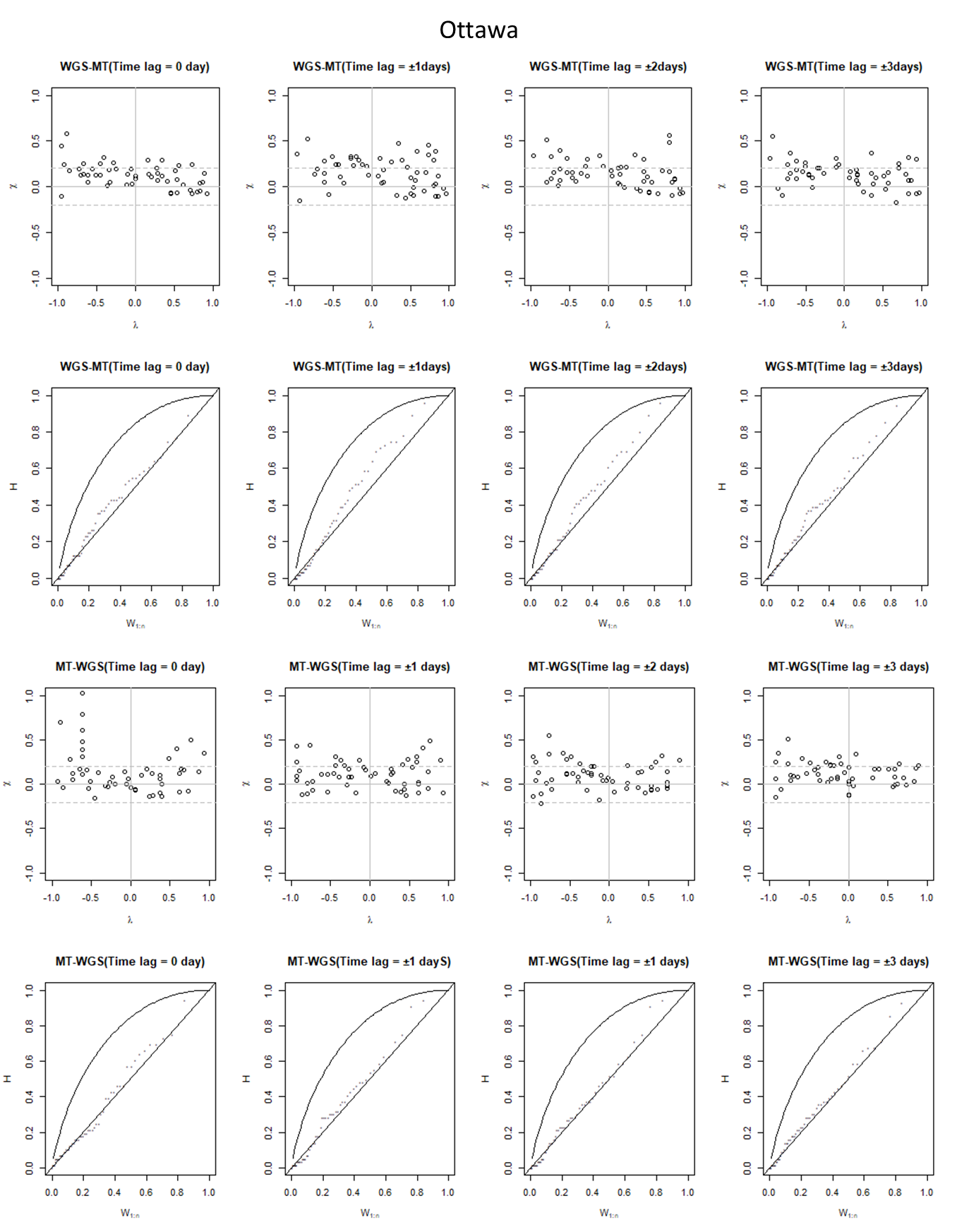
(c-1)


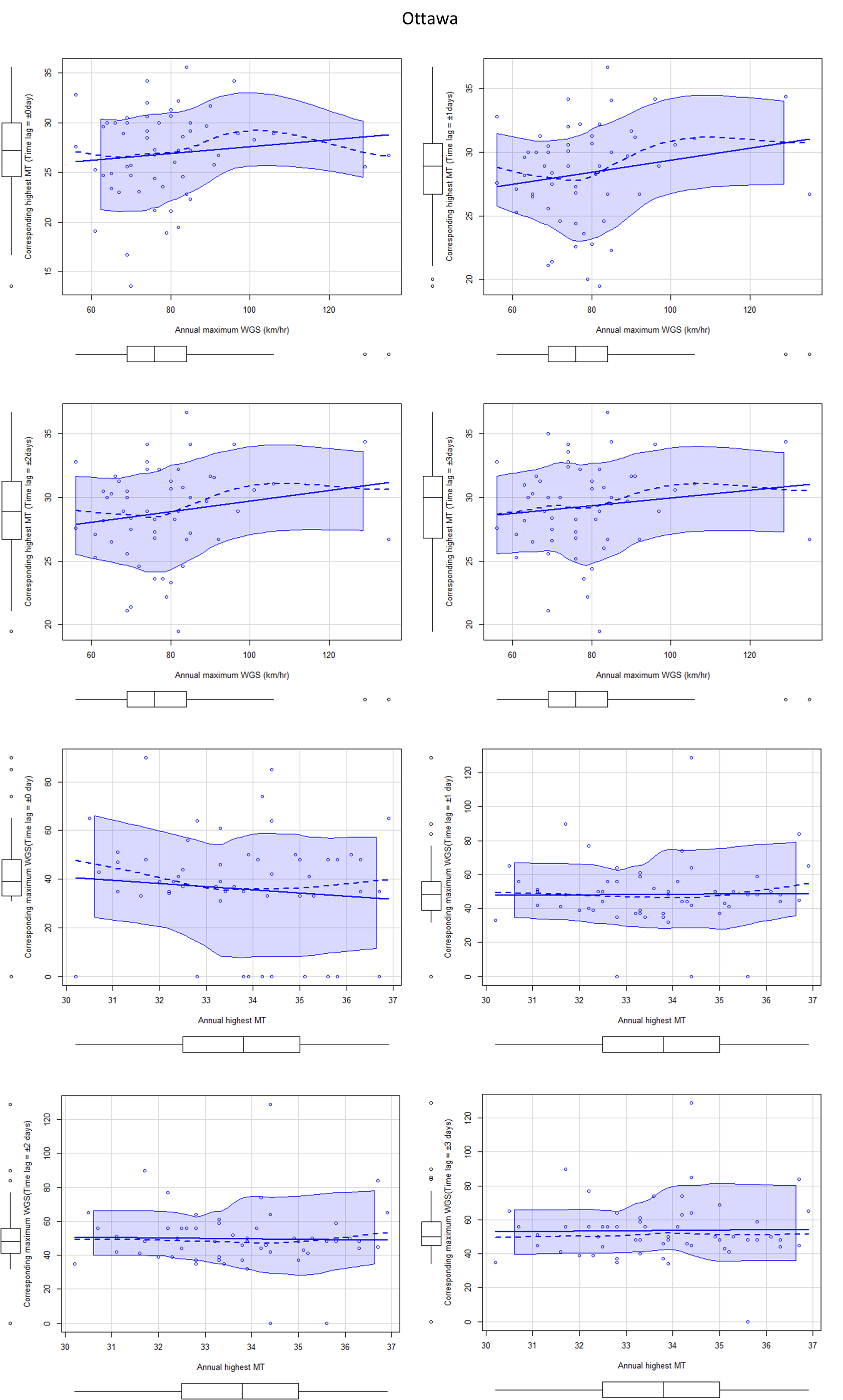
(c-2)


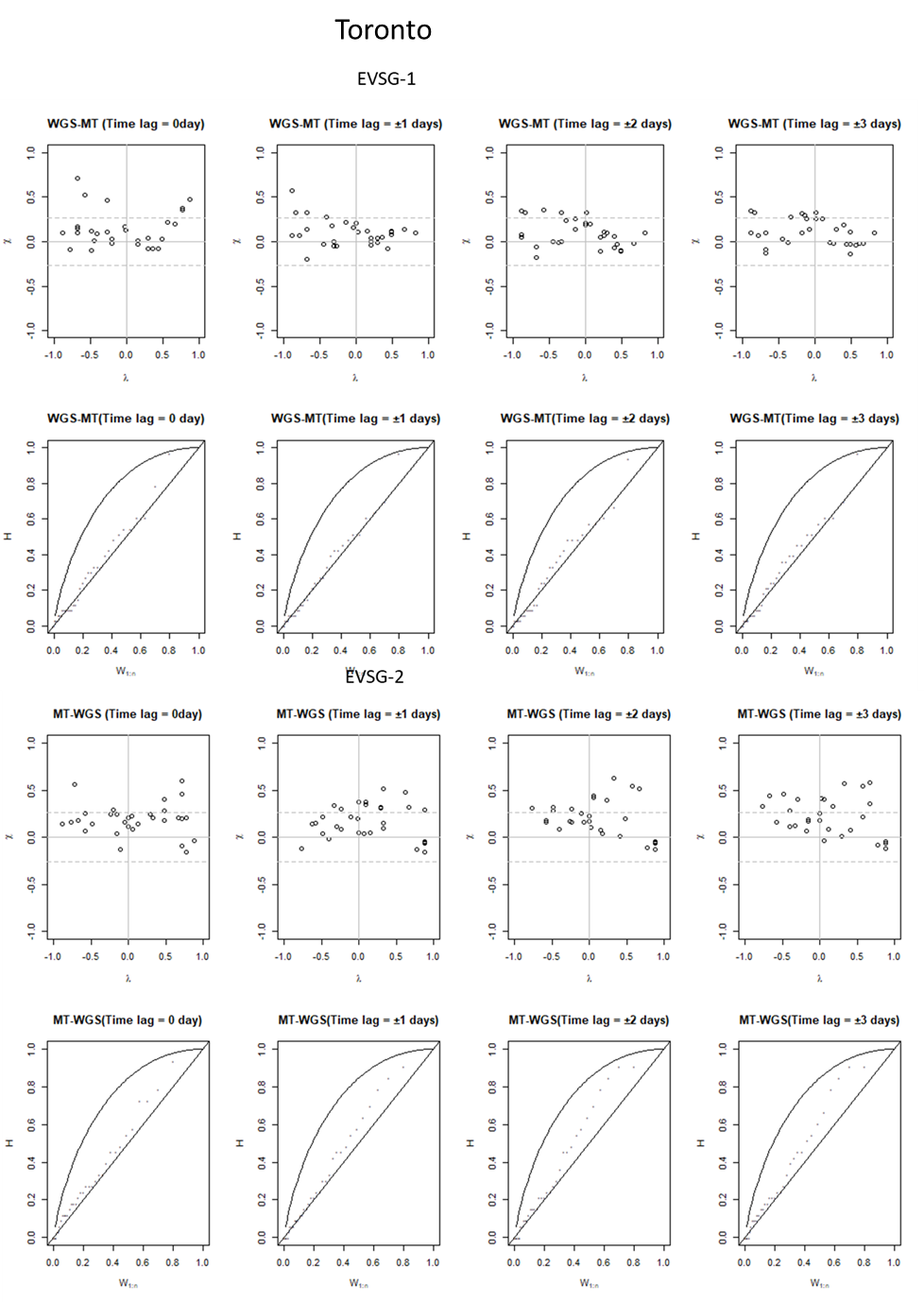
(d-1)


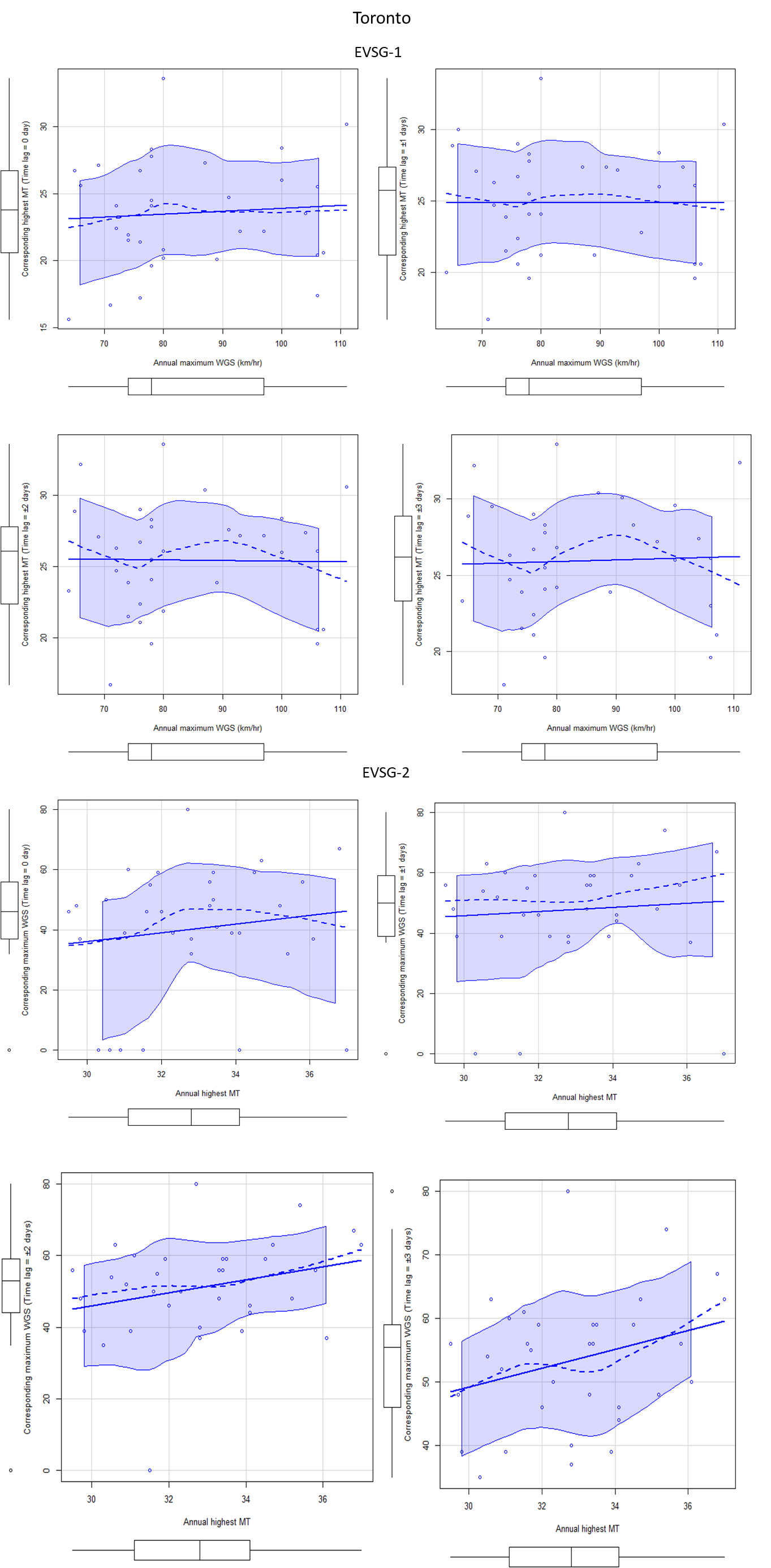
(d-2)


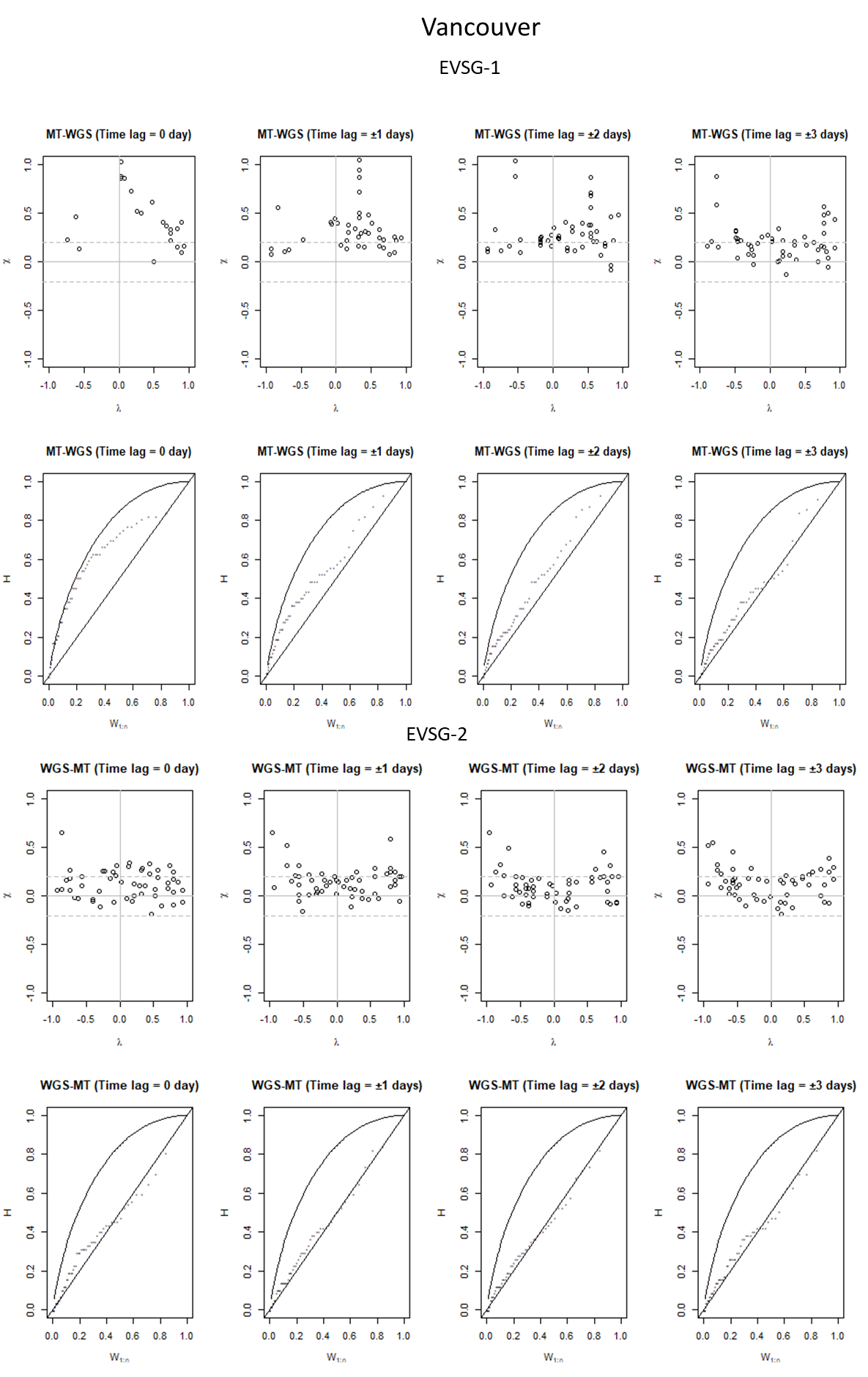
(e-1)


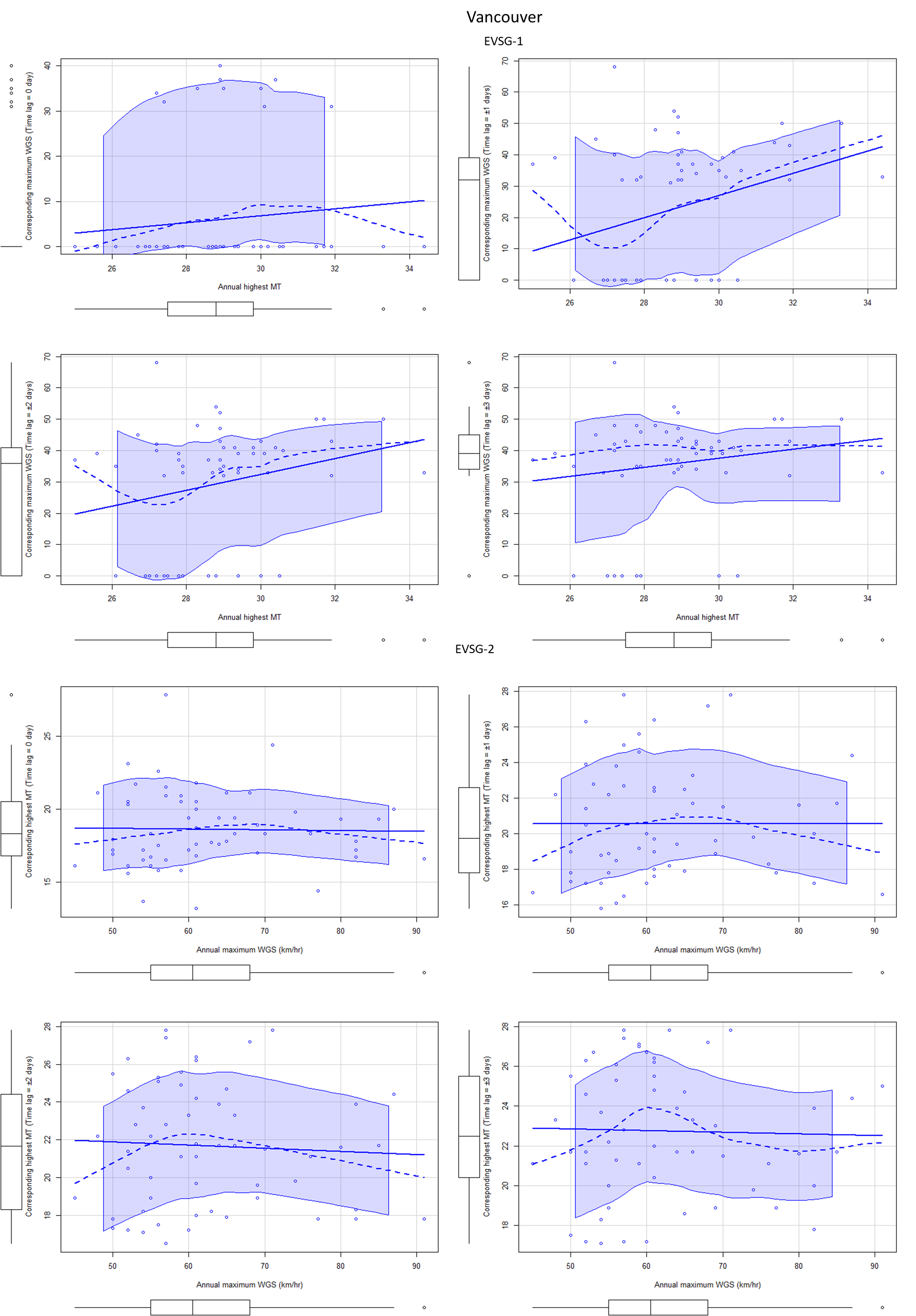
(e-2)


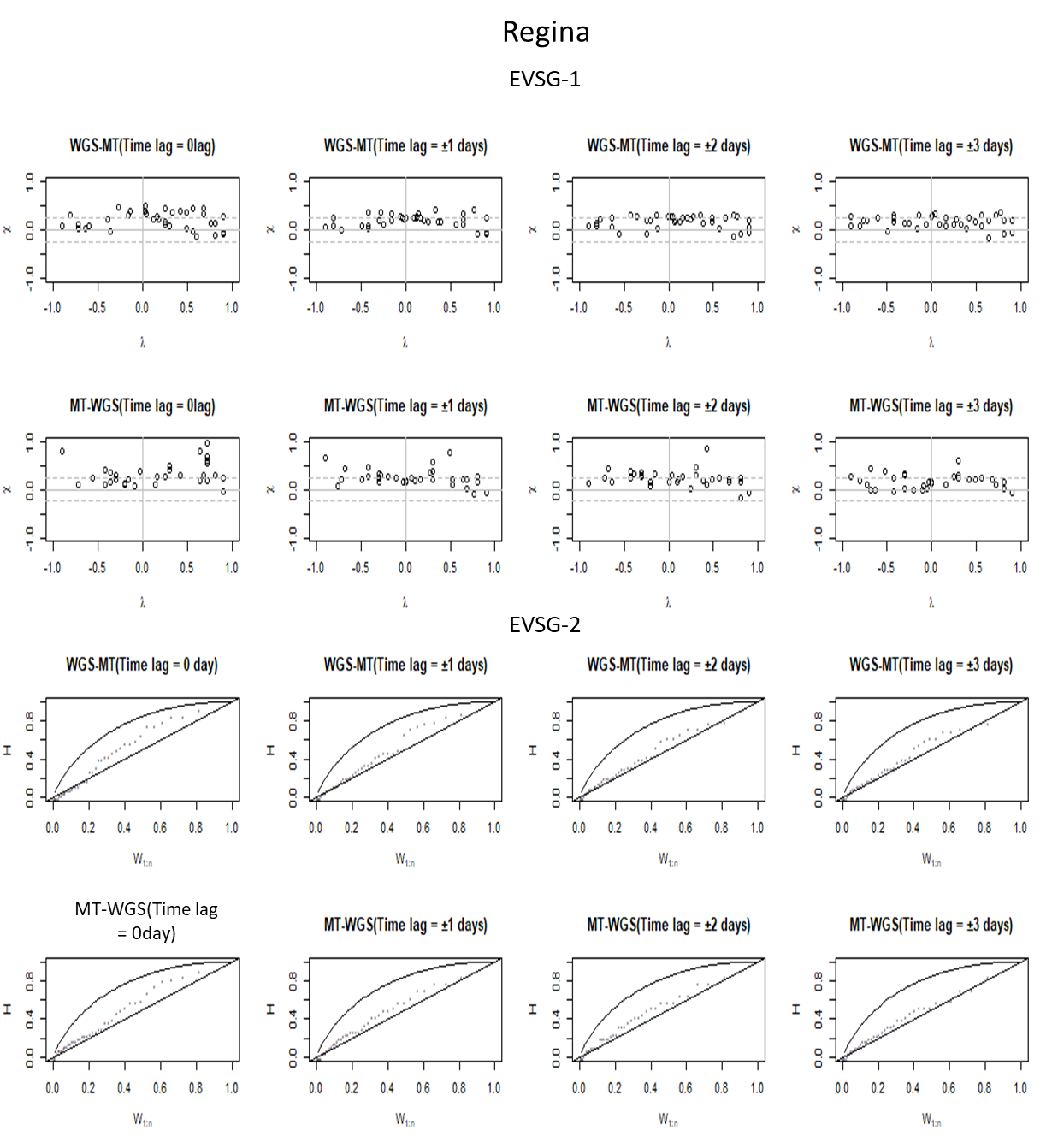
(f-1)


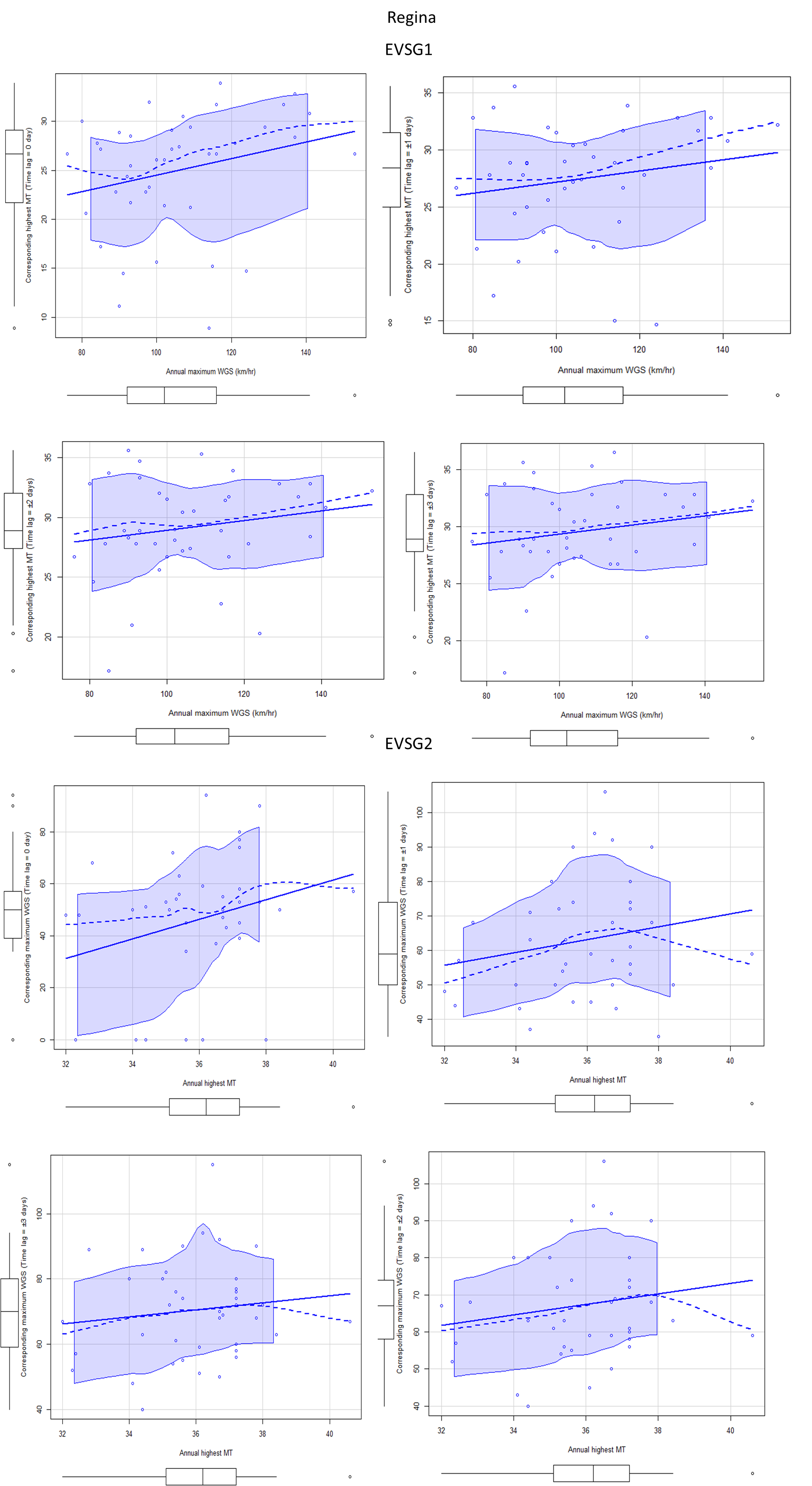
(f-2)


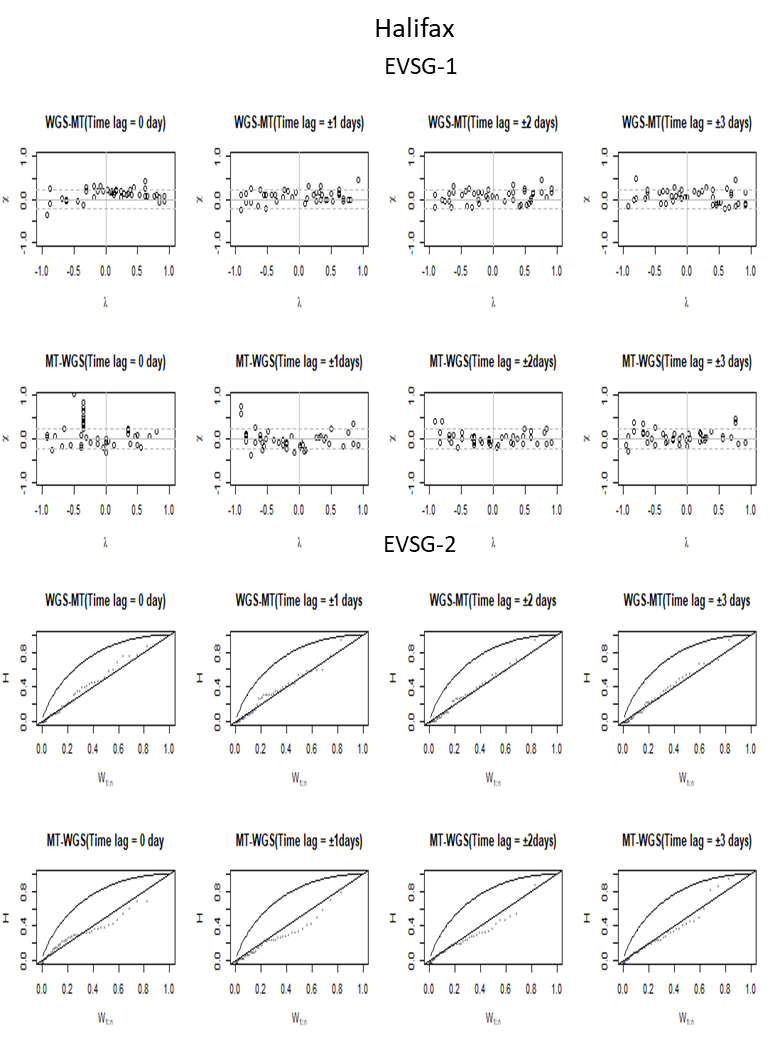
(g-1)


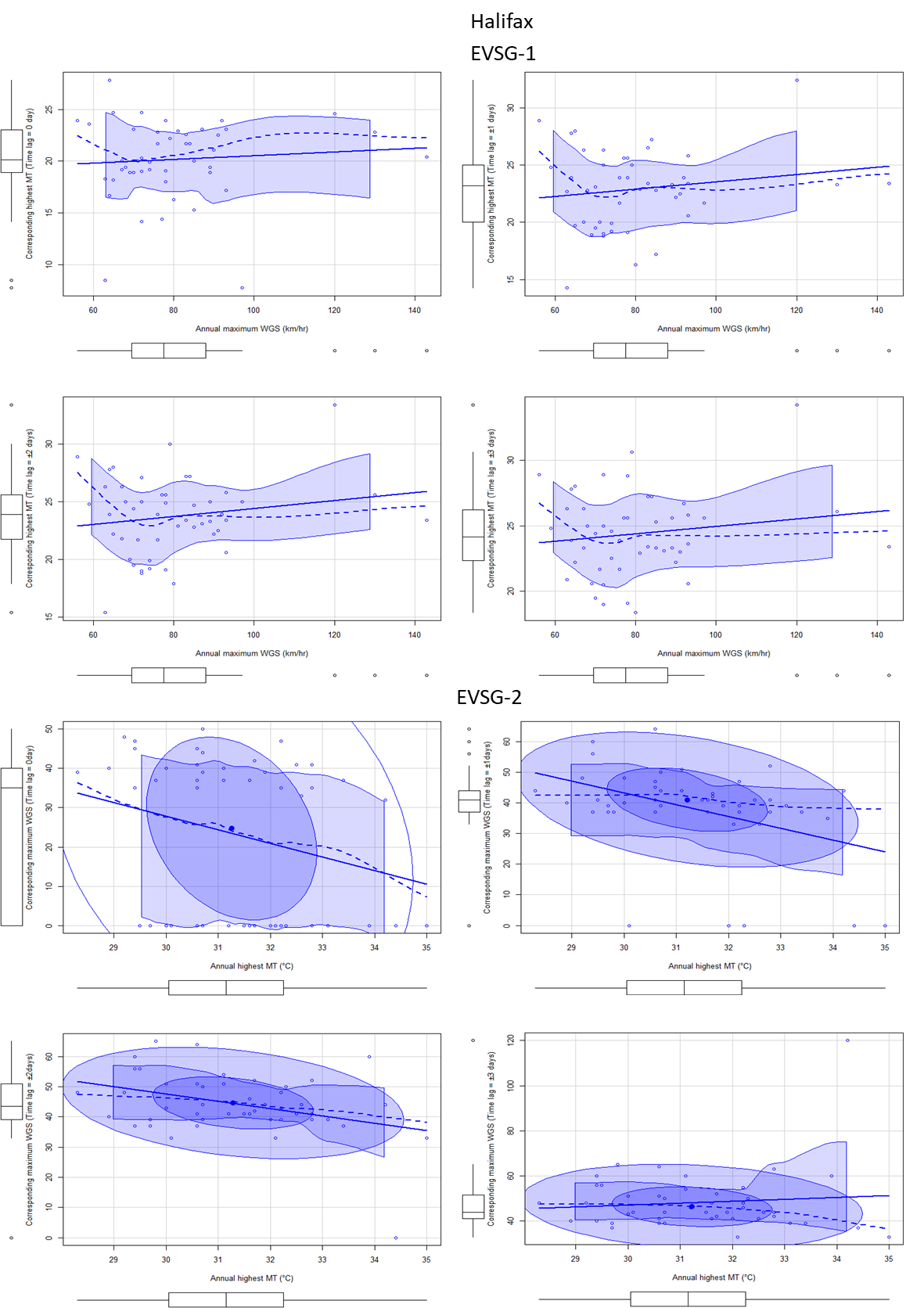
(g-2)


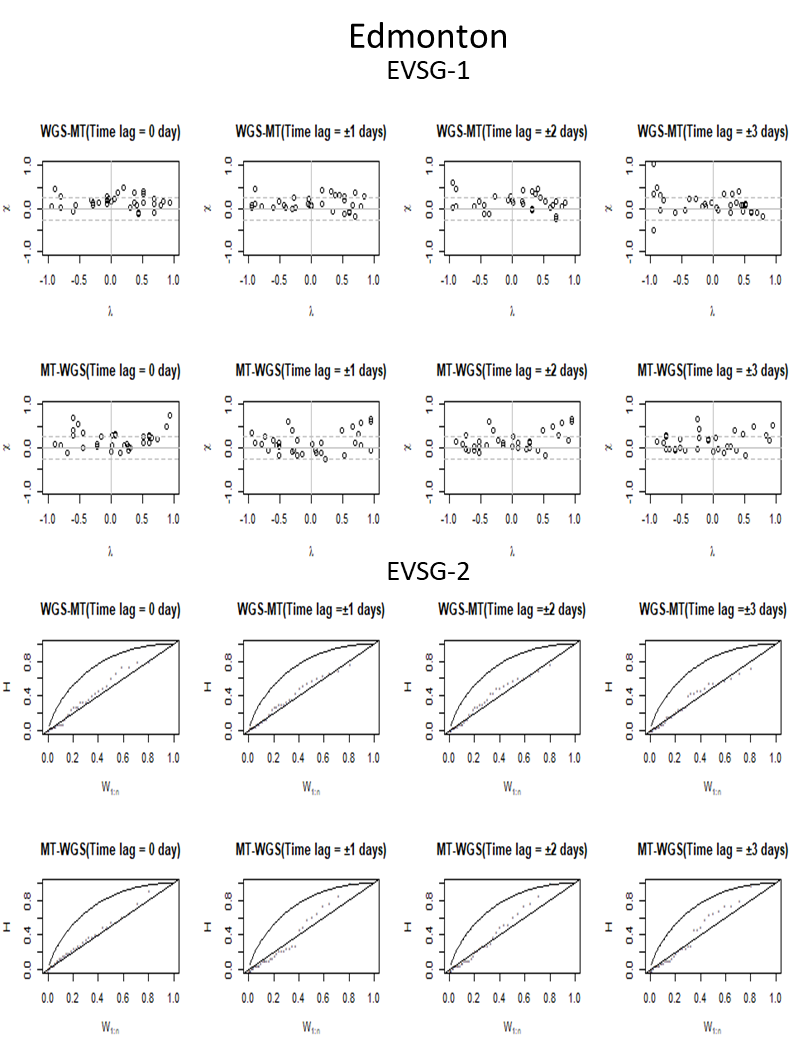
(h-1)


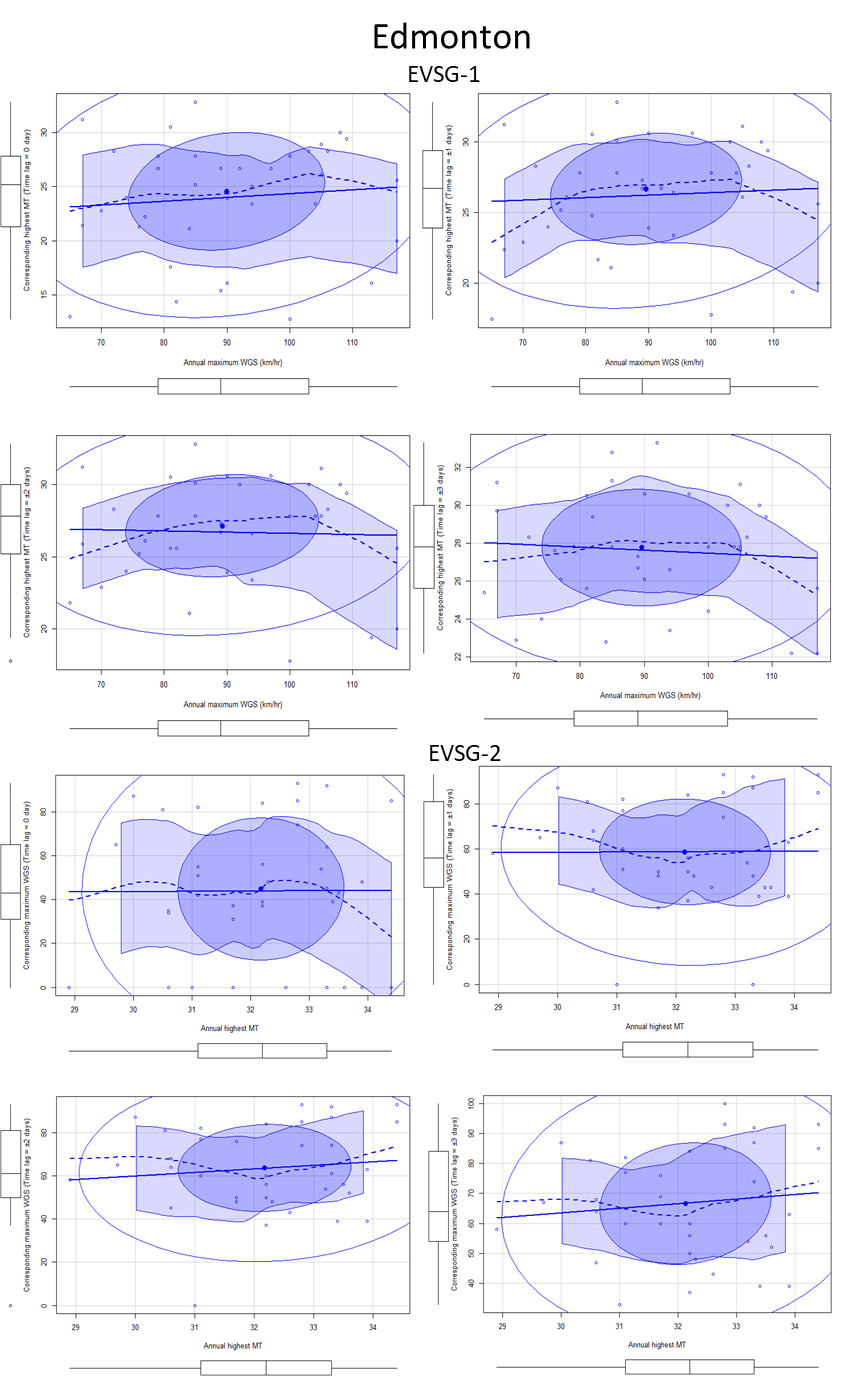
(h-2)


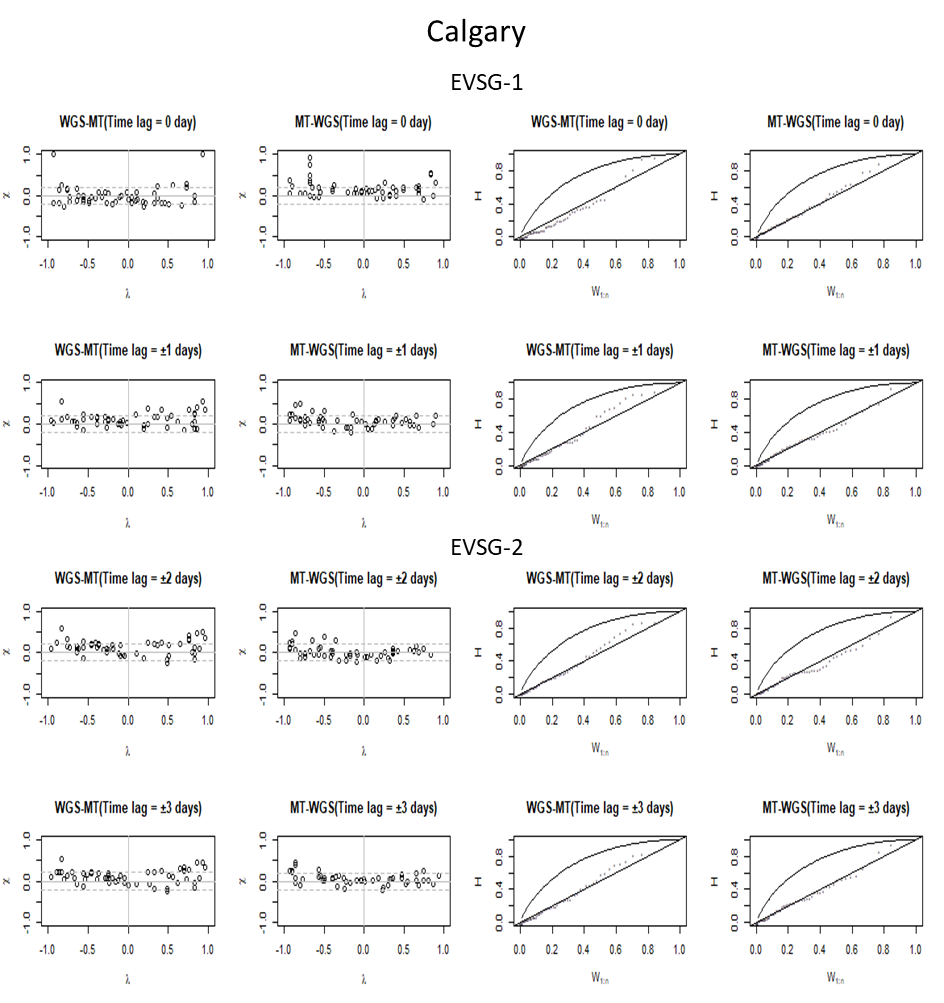
(i-1)


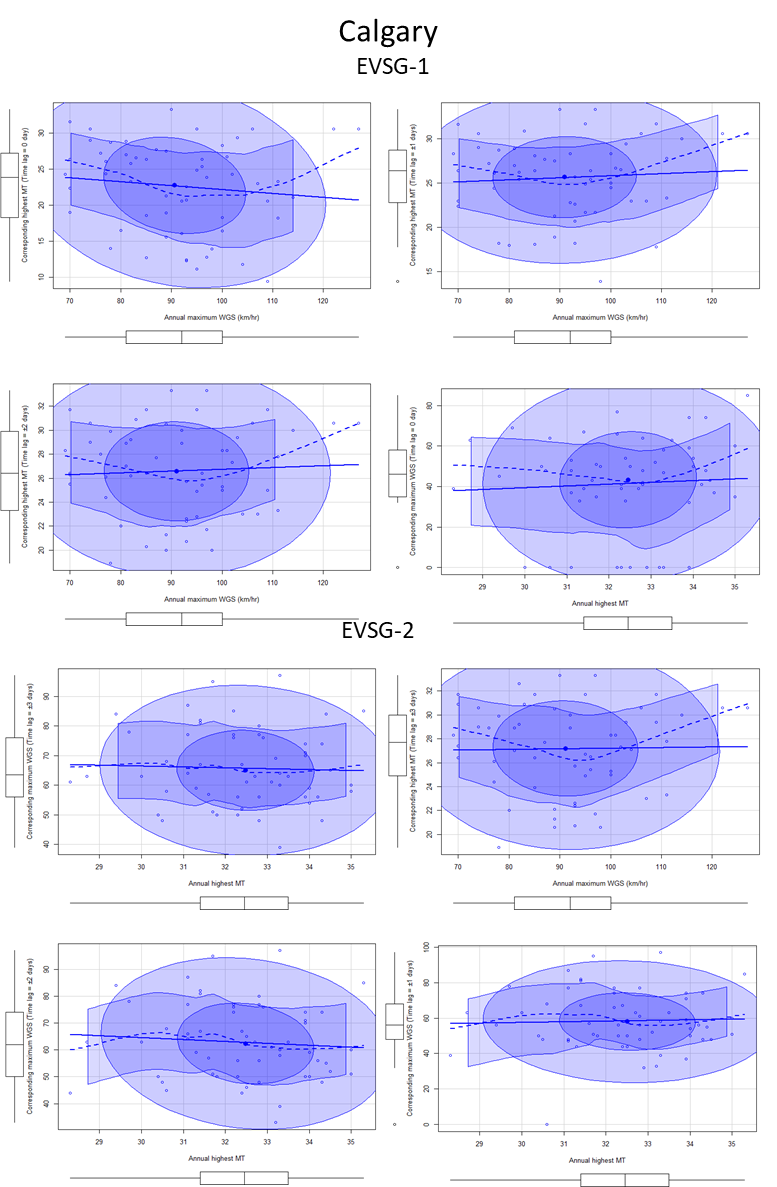
(i-2)

Supplementary SF 2: Stationwise visualizing strength of dependencies via 2-D Chi-plots, 2-D Kendall’s (K) plots and 2-D scatter plots (a-1 and a-2) Montreal (b-1 and b-2) Quebec city (c-1 and c-2) Ottawa(d-1 and d-2) Toronto(e-1 and e-2) Vancouver (f-1 and f-2) Regina (g-1 and g-2) Halifax (h-1 and h-2) Edmonton (i-1 and i-2) Calgary


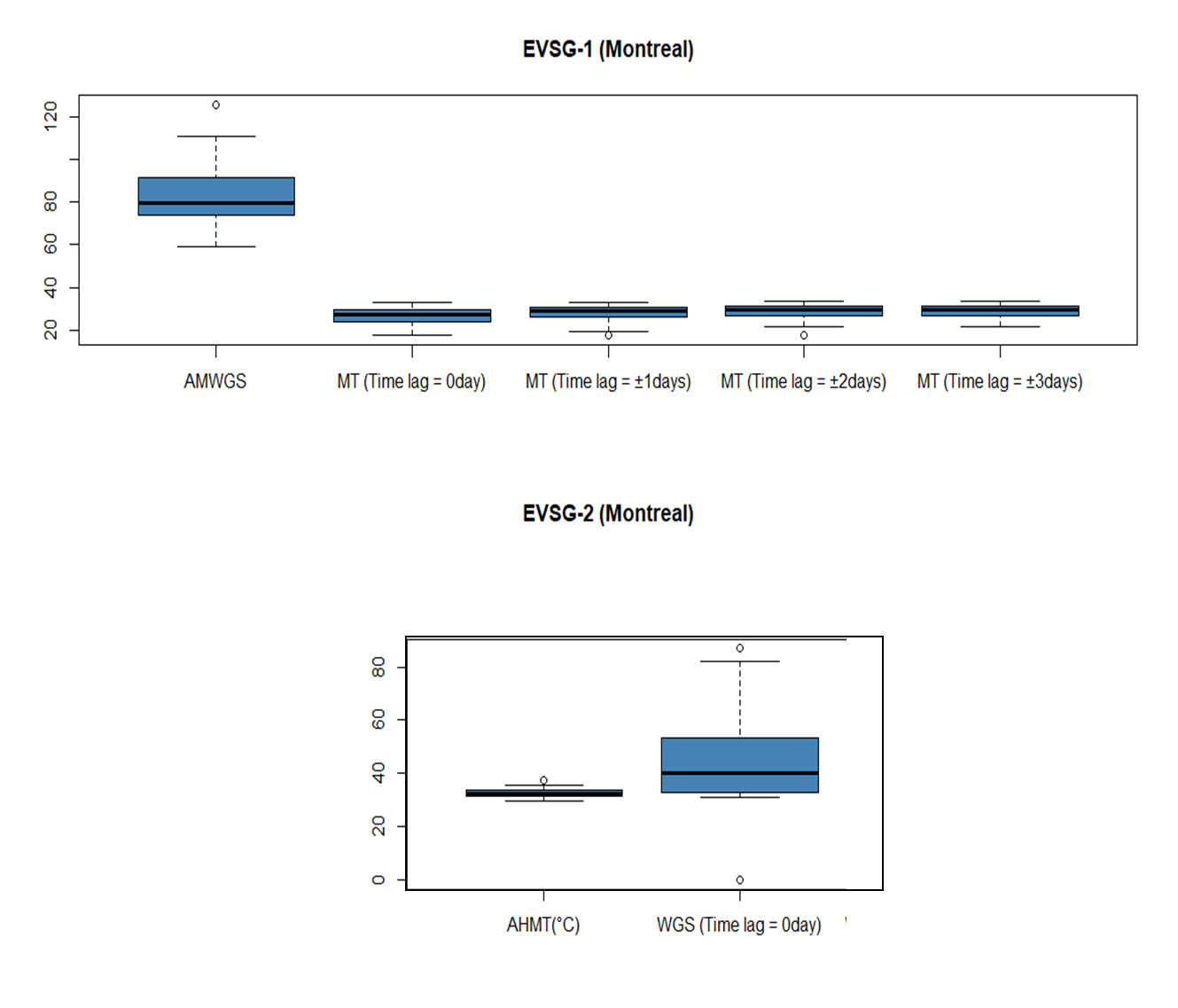
(a)

**
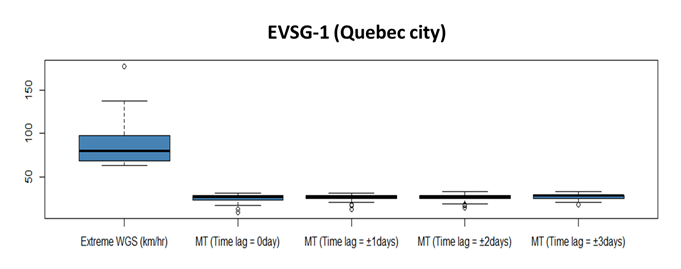
**(b)


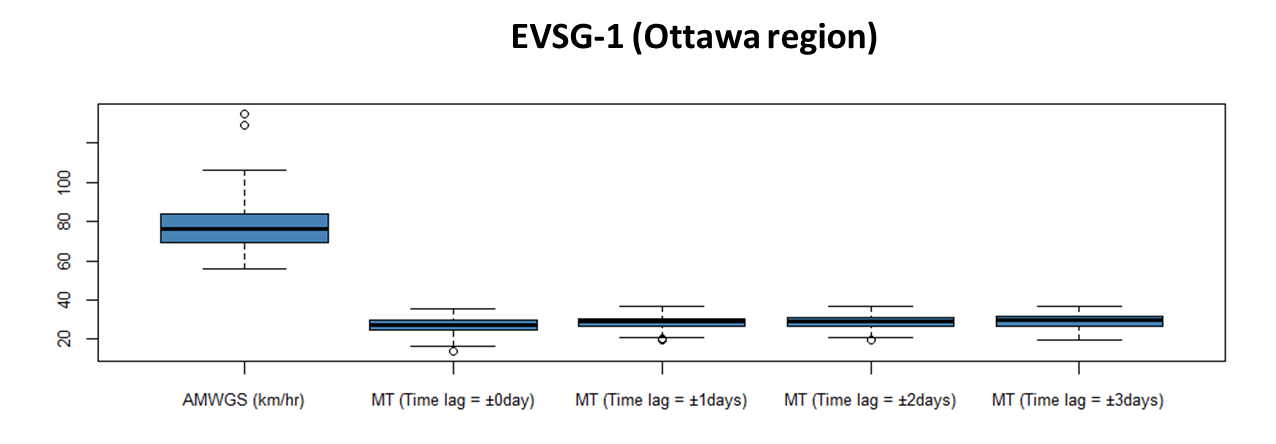
(C)


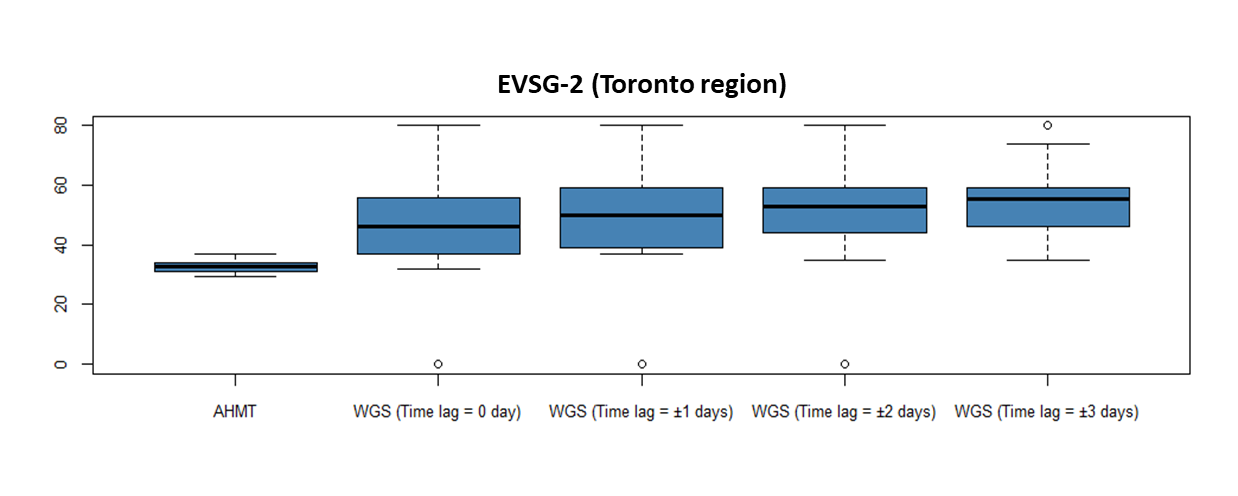
(d)


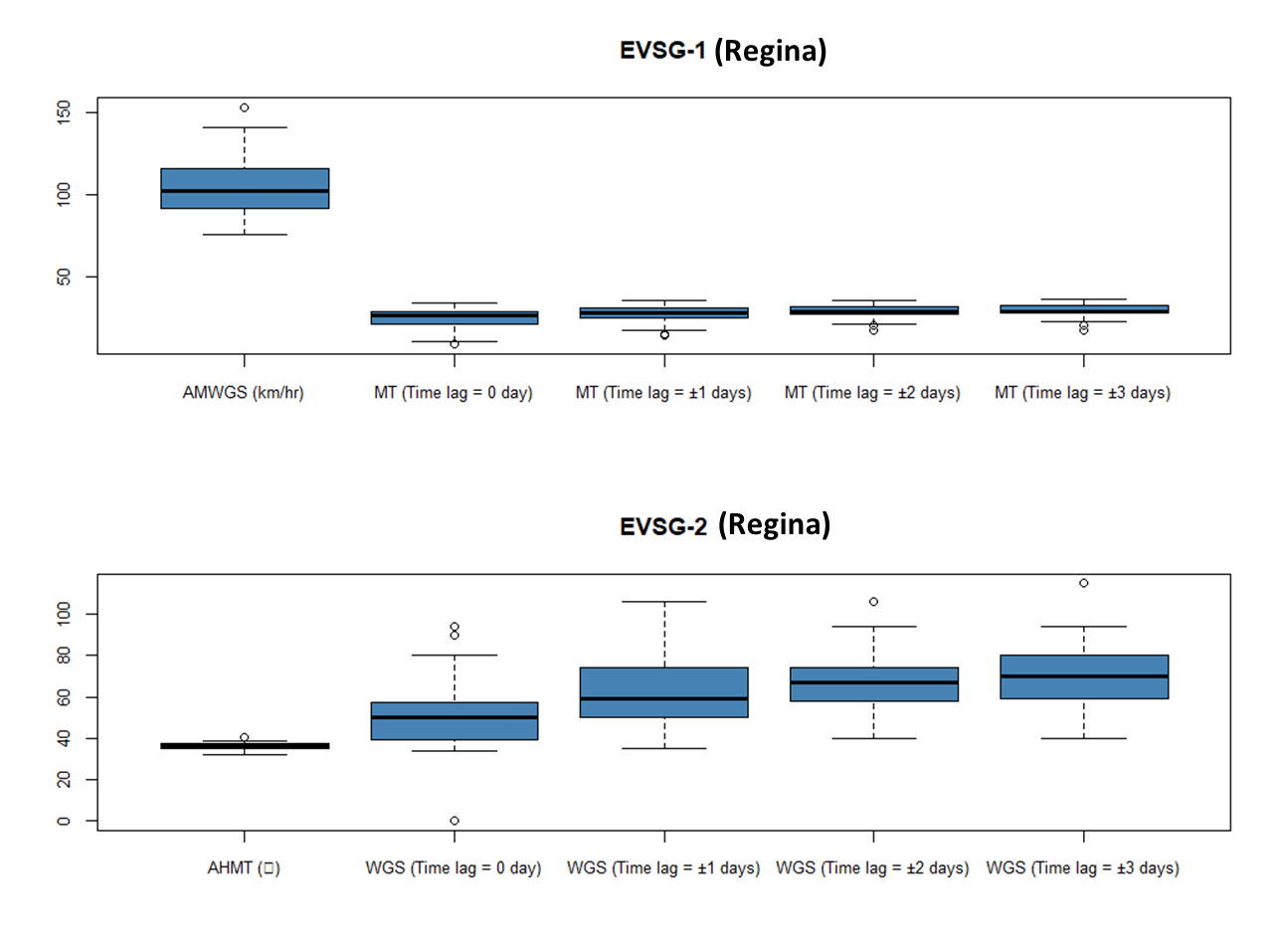
(e)


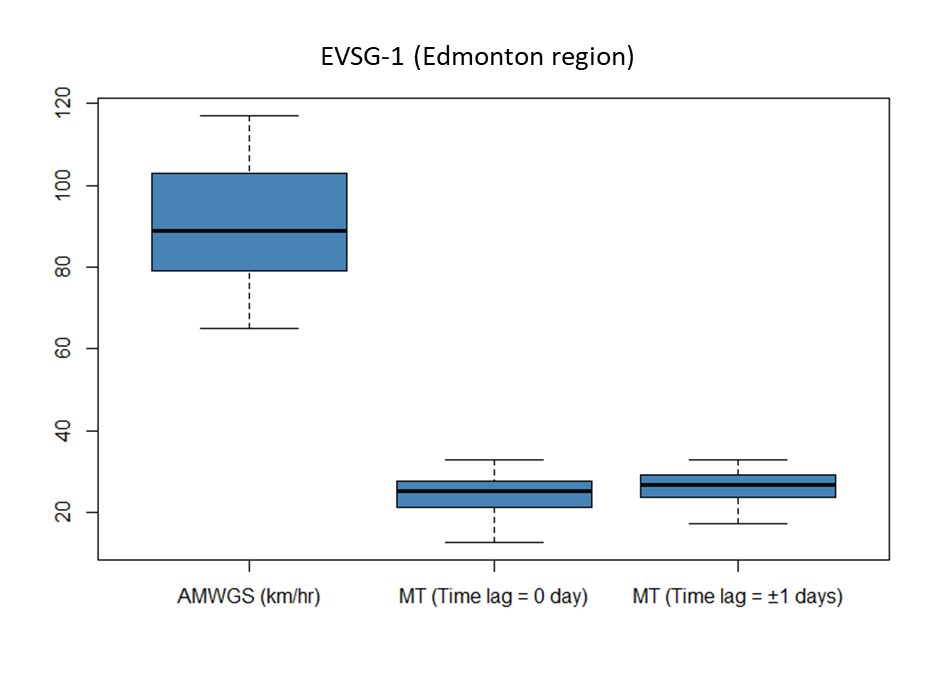
 (f)


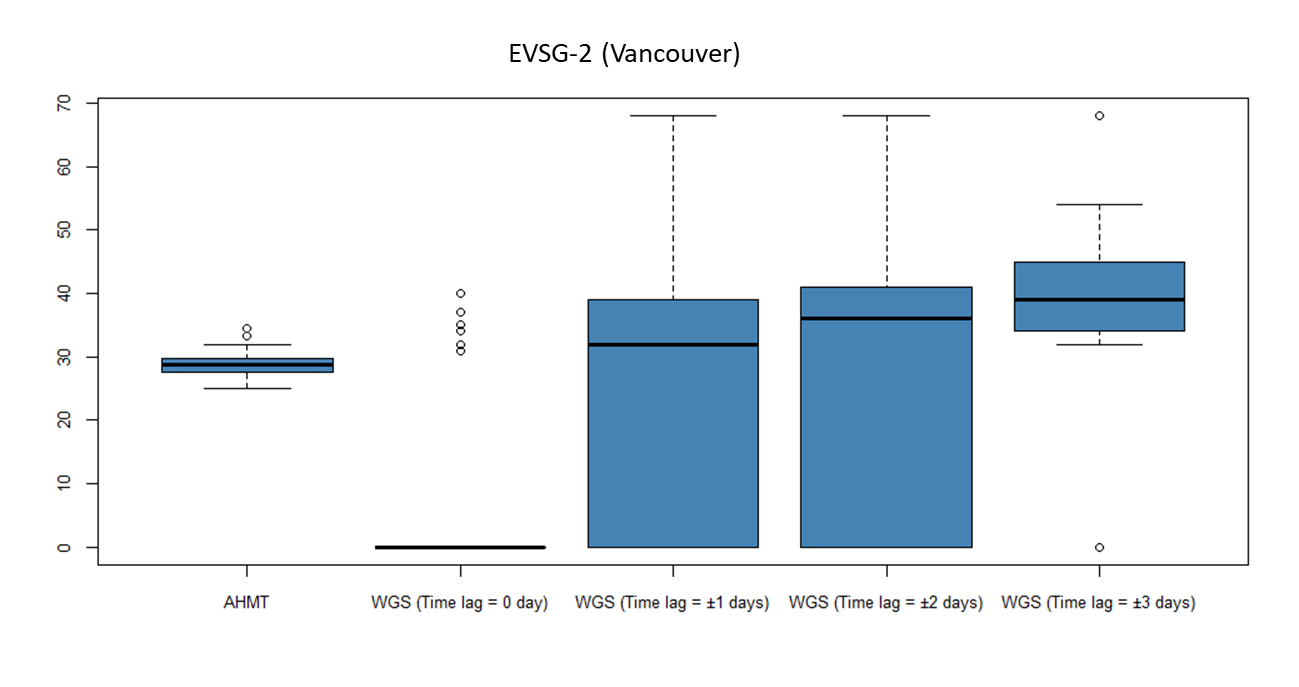
(g)

Supplementary SF 3: Stationwise Box-whisker plots (a) Montreal (b) Quebec City (c) Ottawa (d) Toronto (e) Regina (f) Edmonton (g) Vancouver


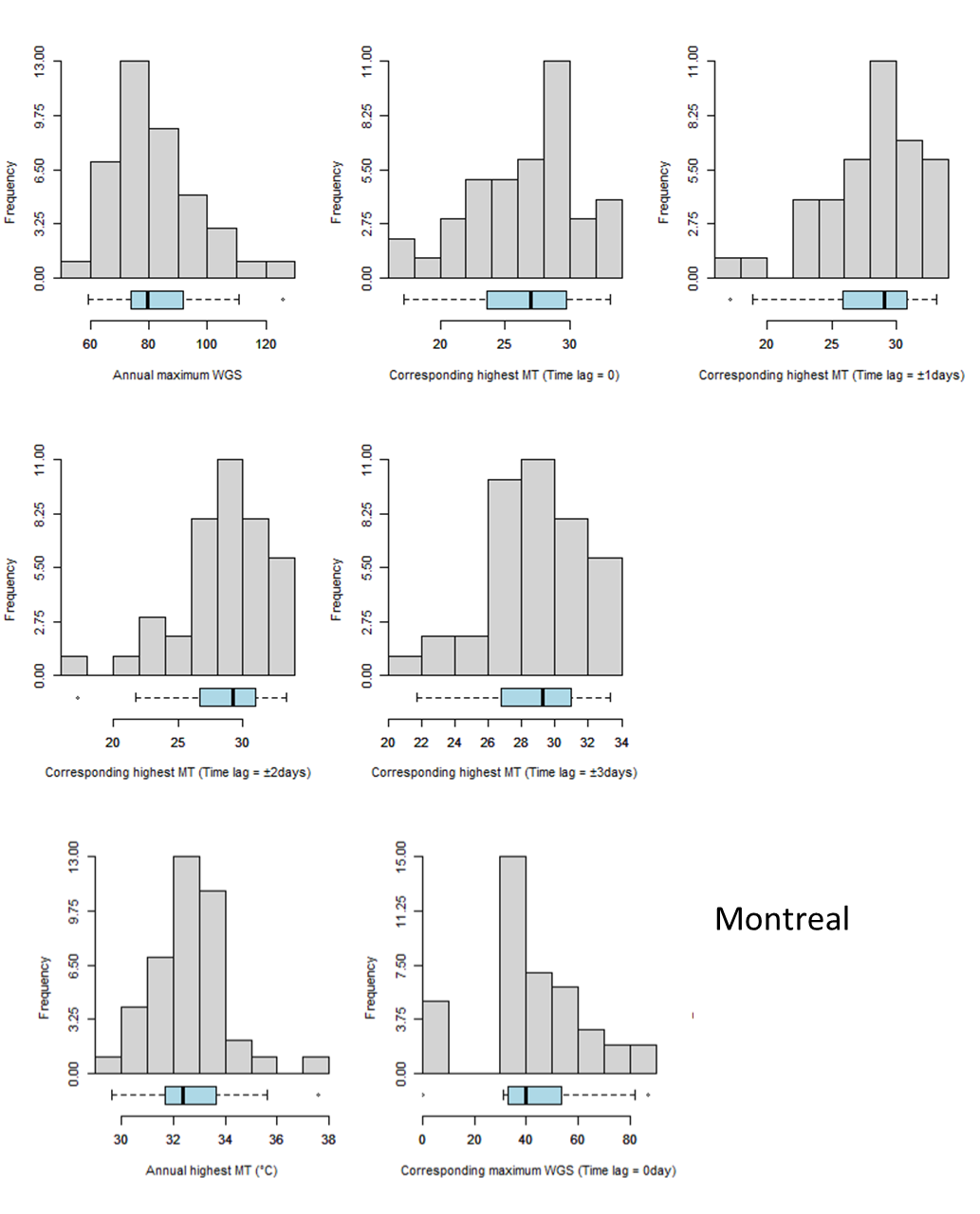
(a)


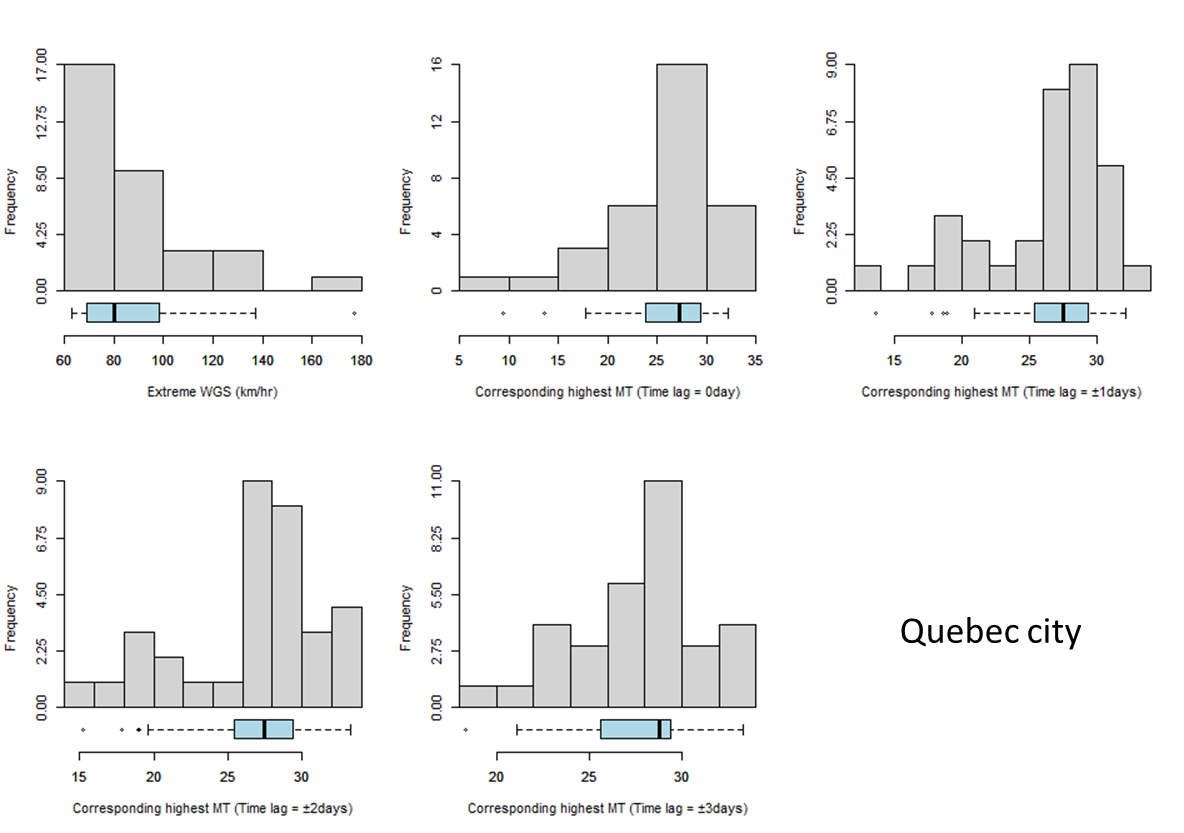
(b)


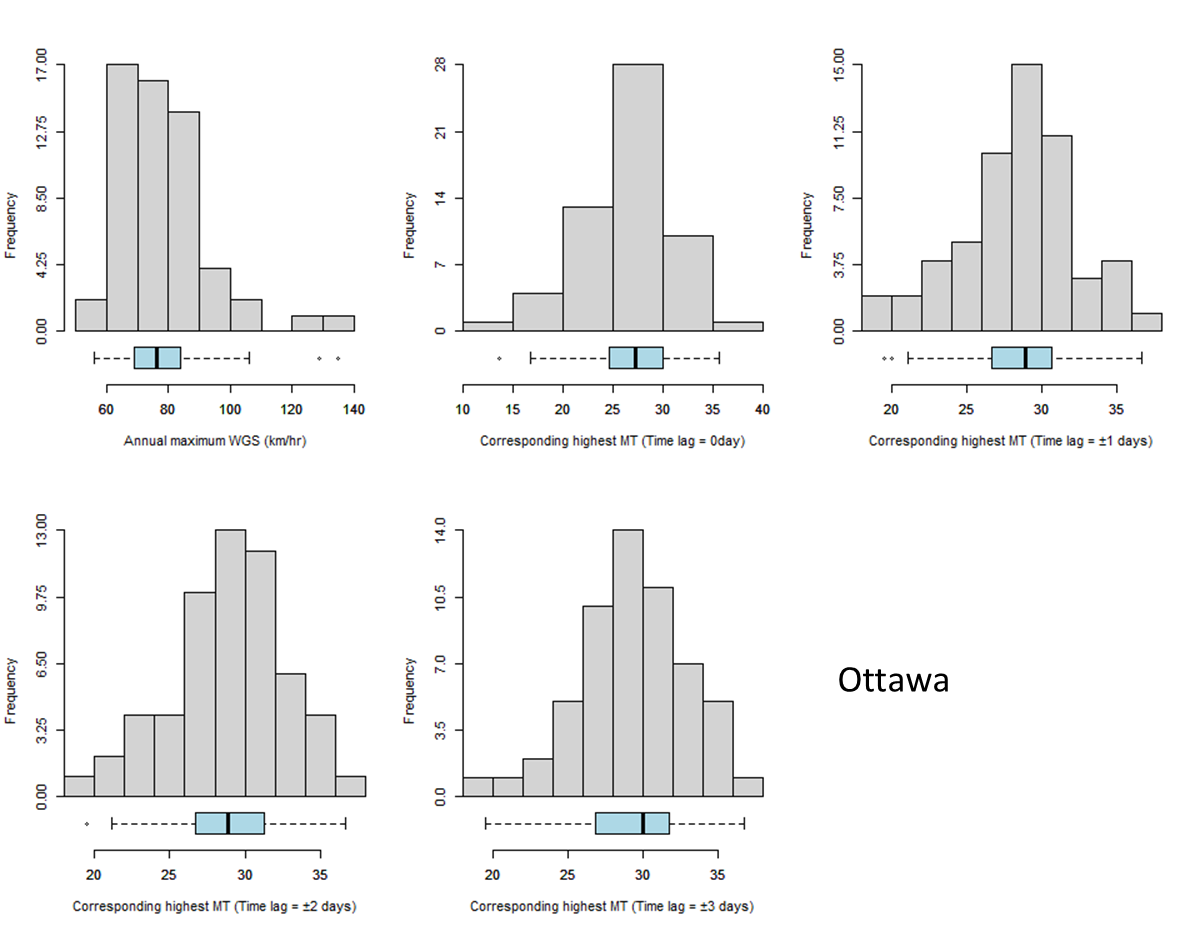
(c)


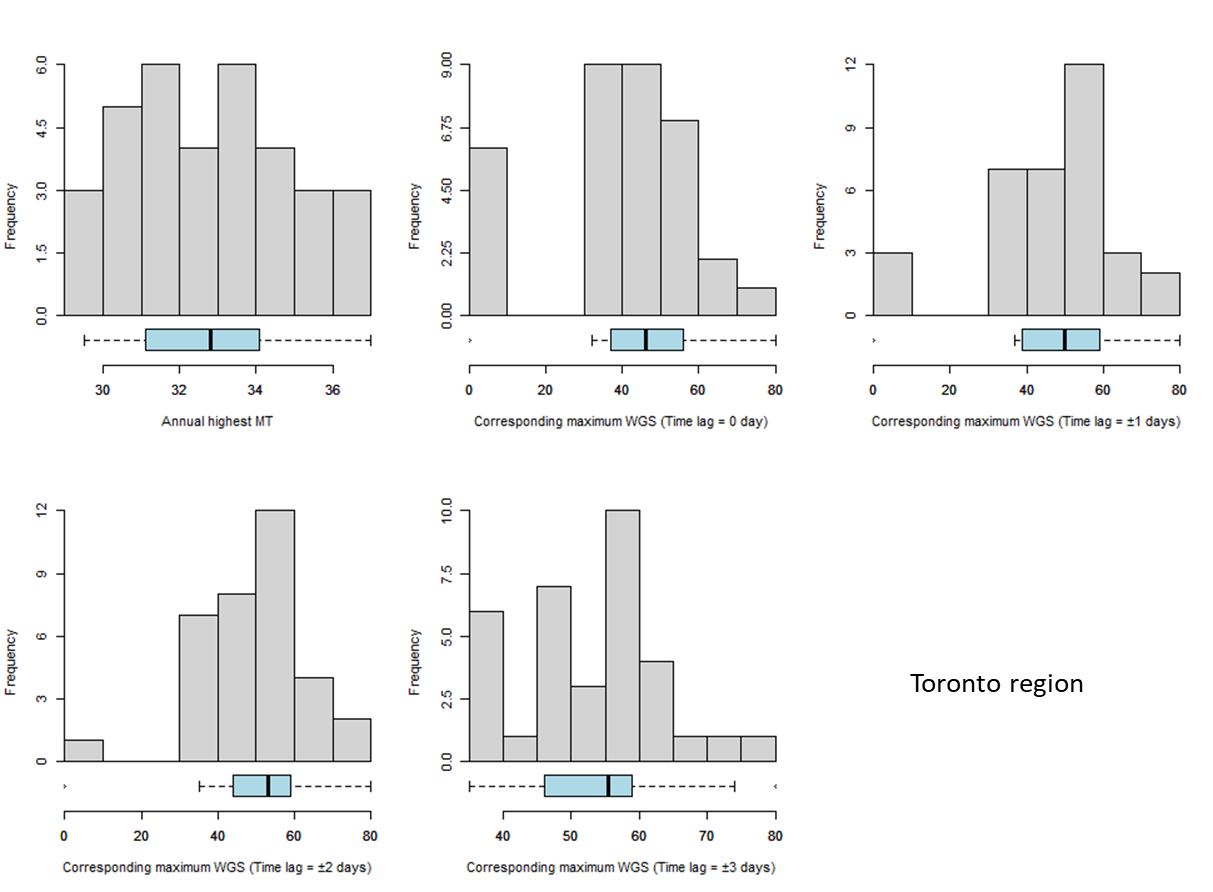
 (d)


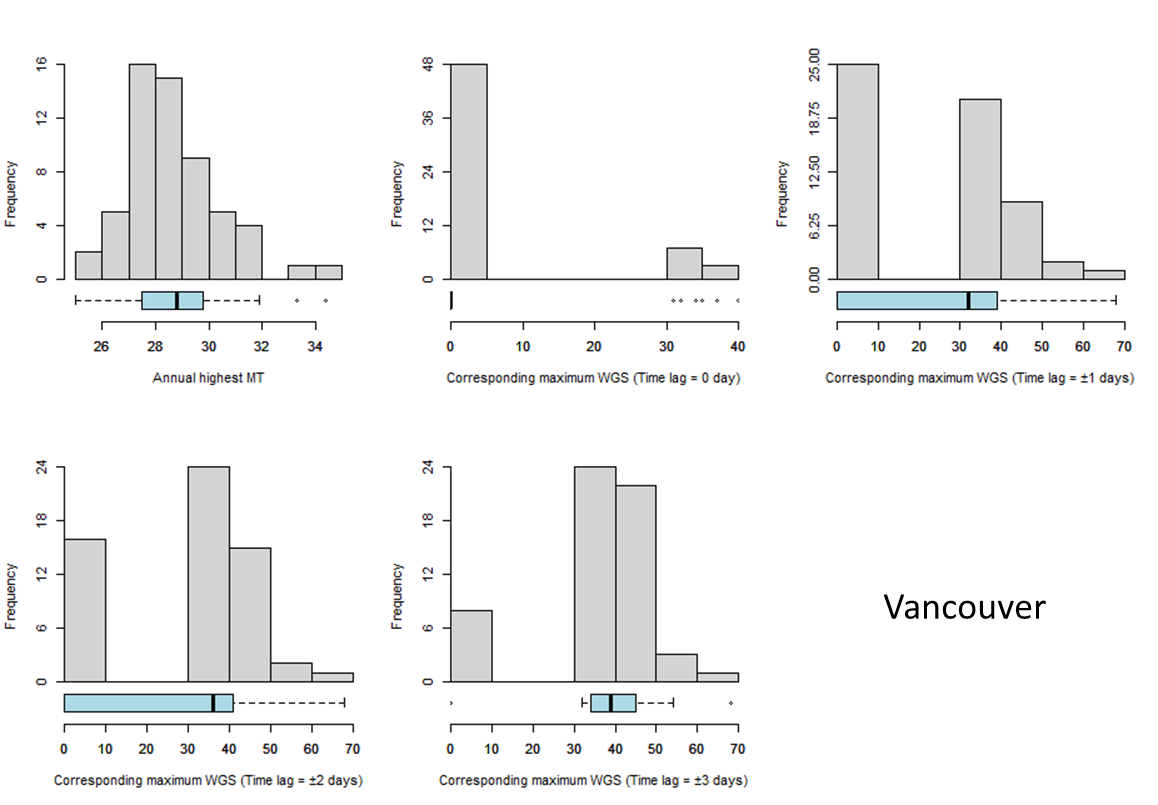
 (e)


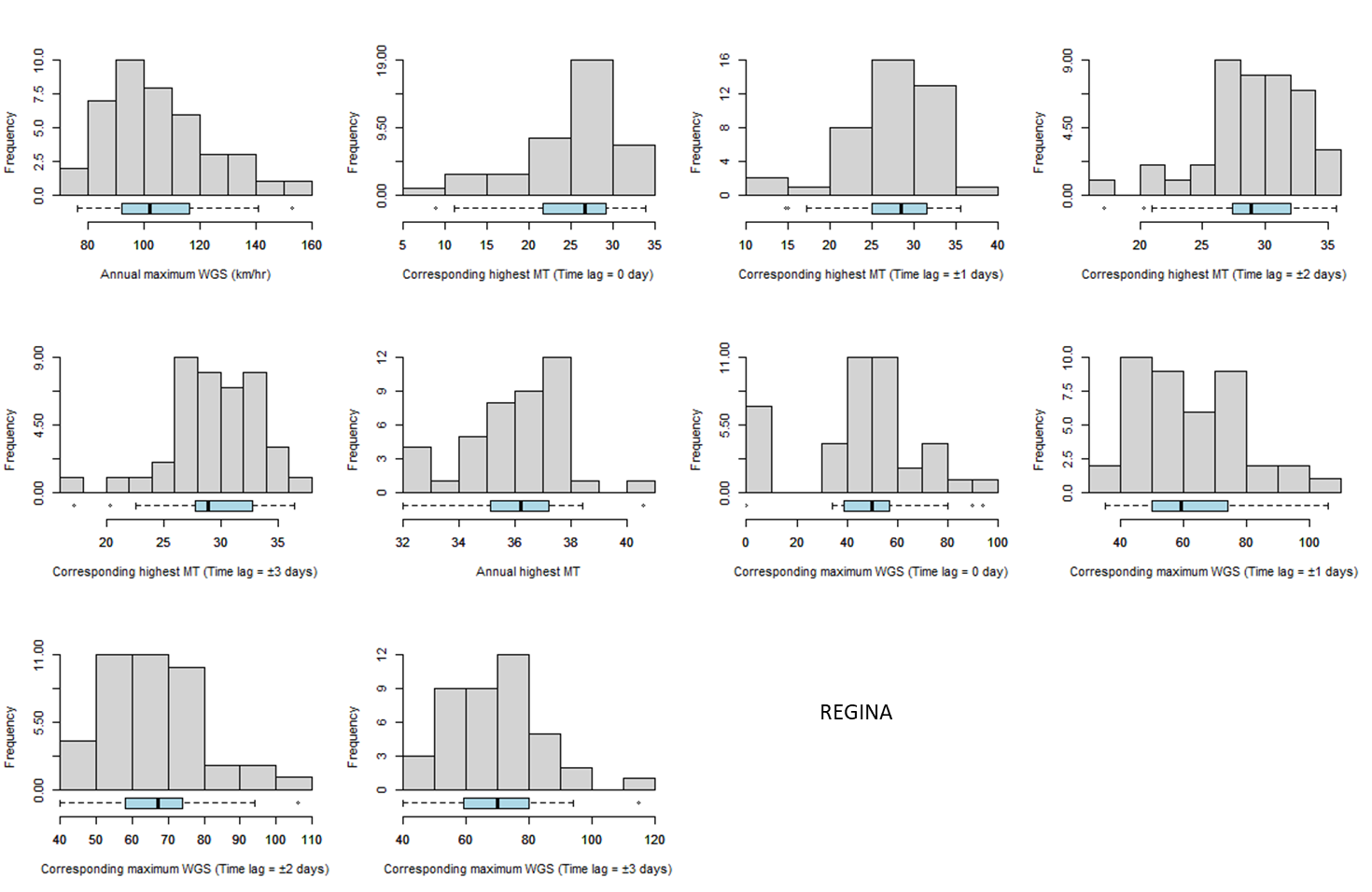
(f)


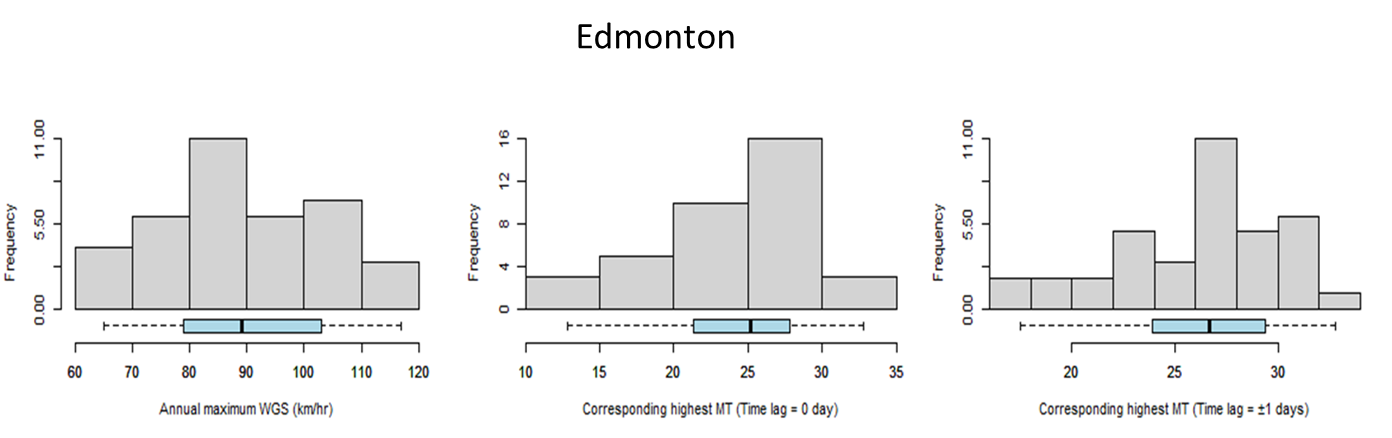
(g)

Supplementary SF 4.1: Stationwise histogram distribution plots (a) Montreal (b) Quebec City (c) Ottawa (d) Toronto (e) Vancouver (f) Regina (g) Edmonton


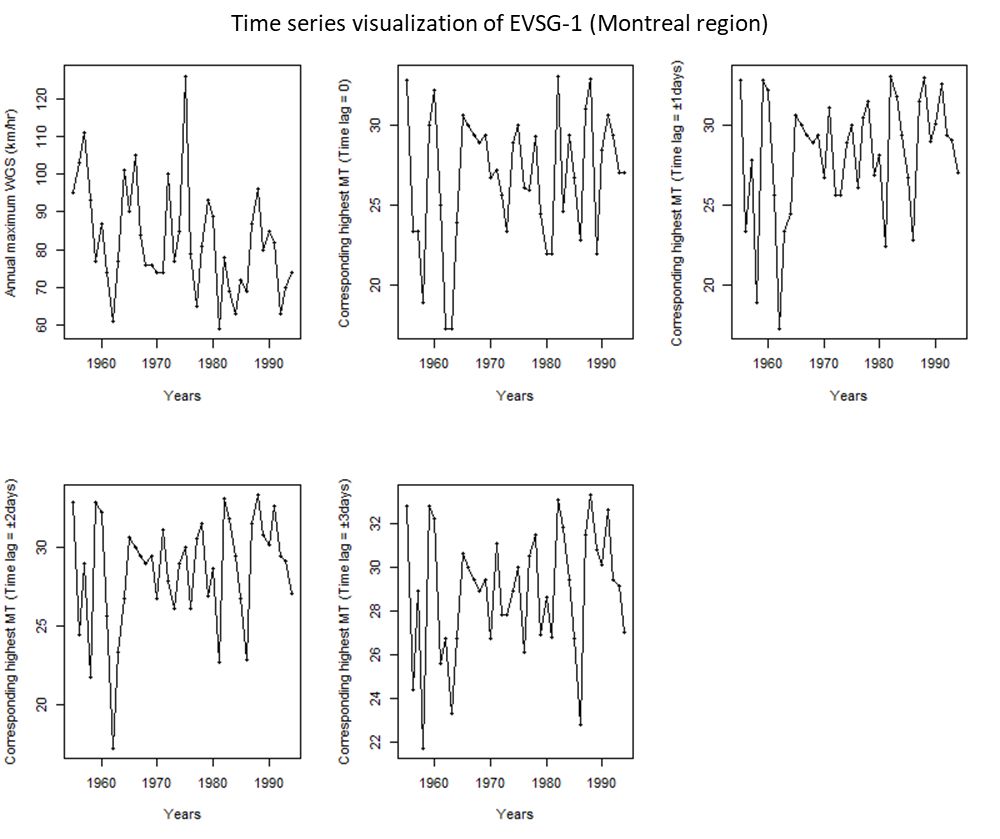
(a-1)


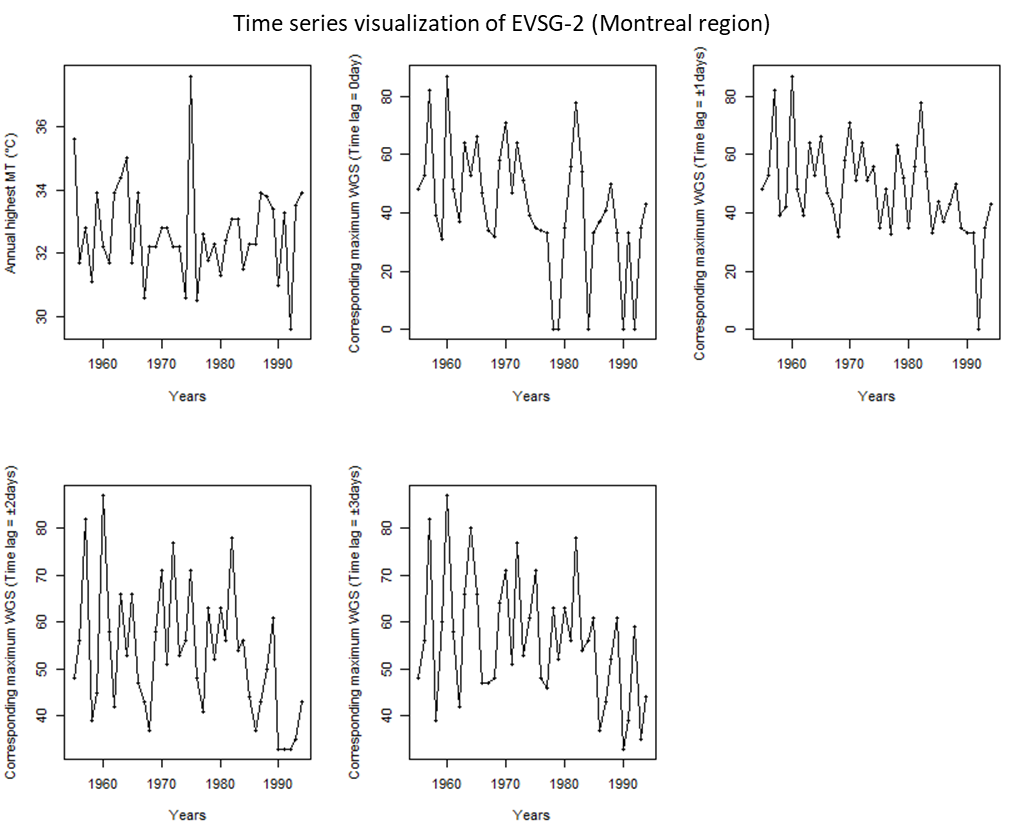
(a-2)


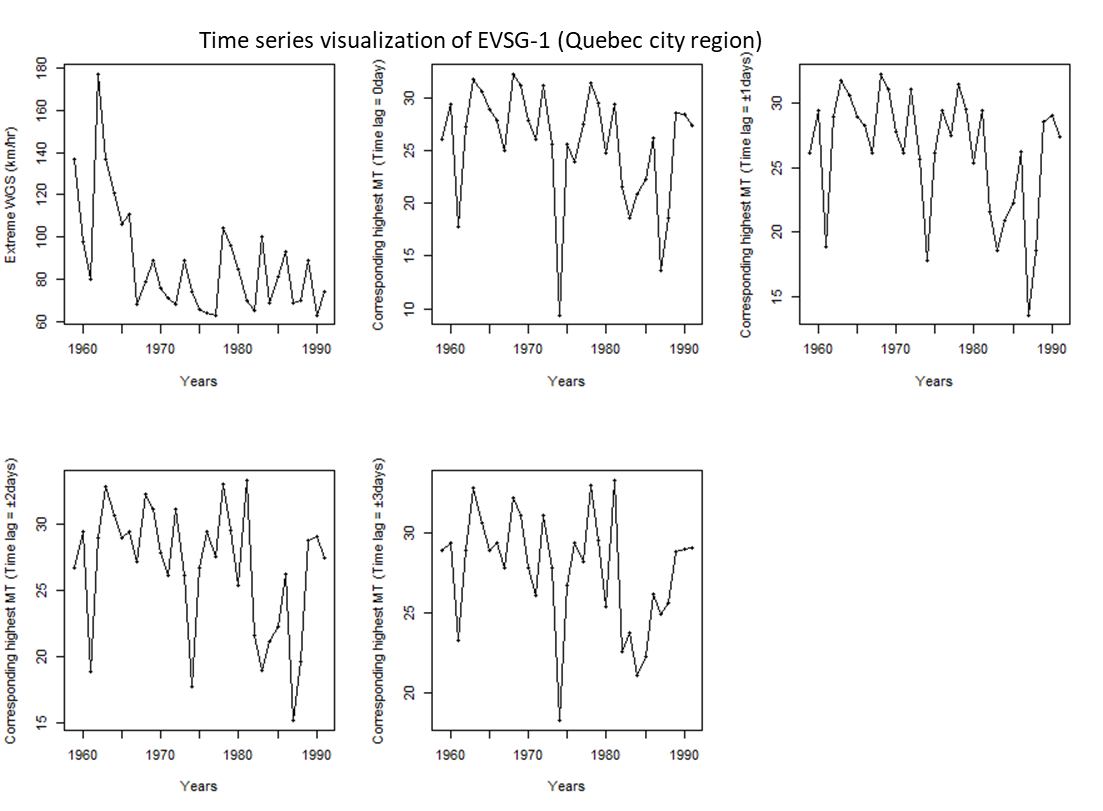
(b-1)


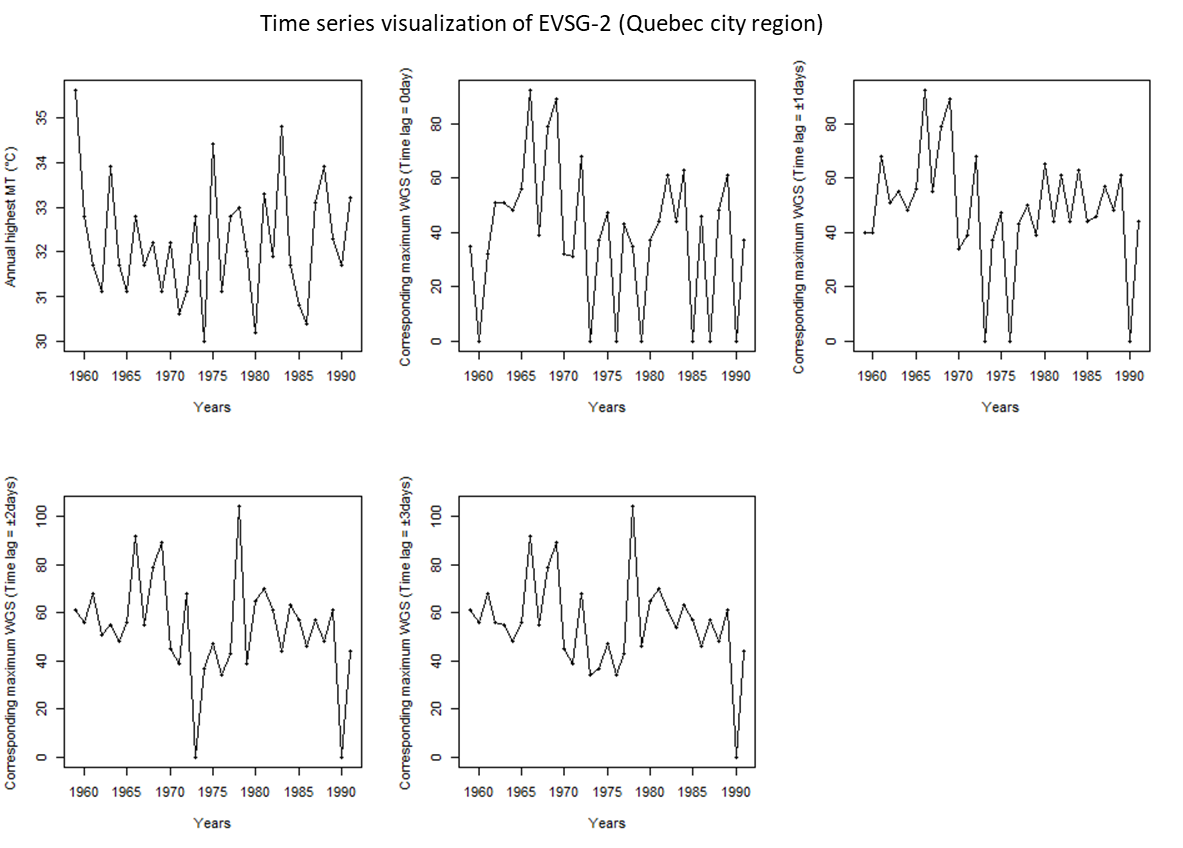
(b-2)


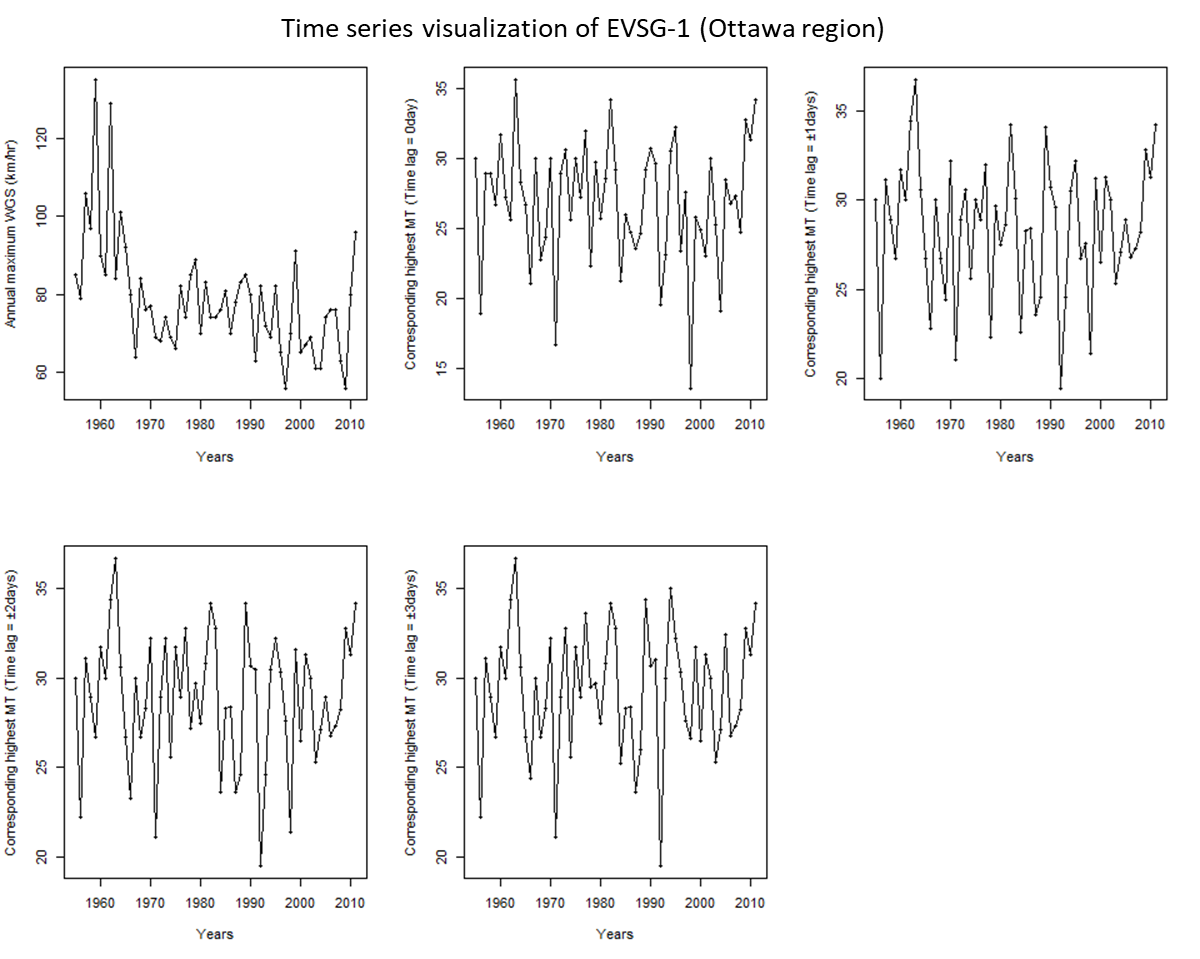
(c-1)


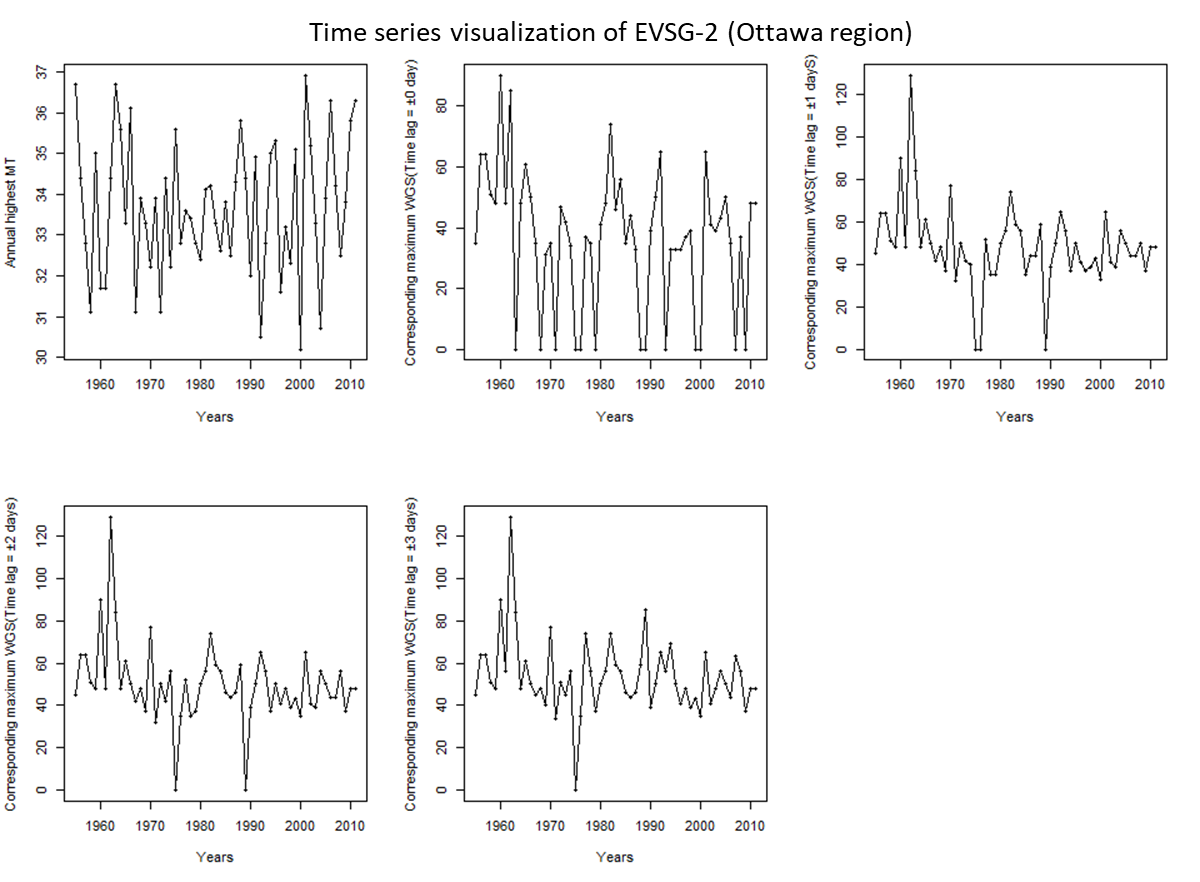
 (c-2)


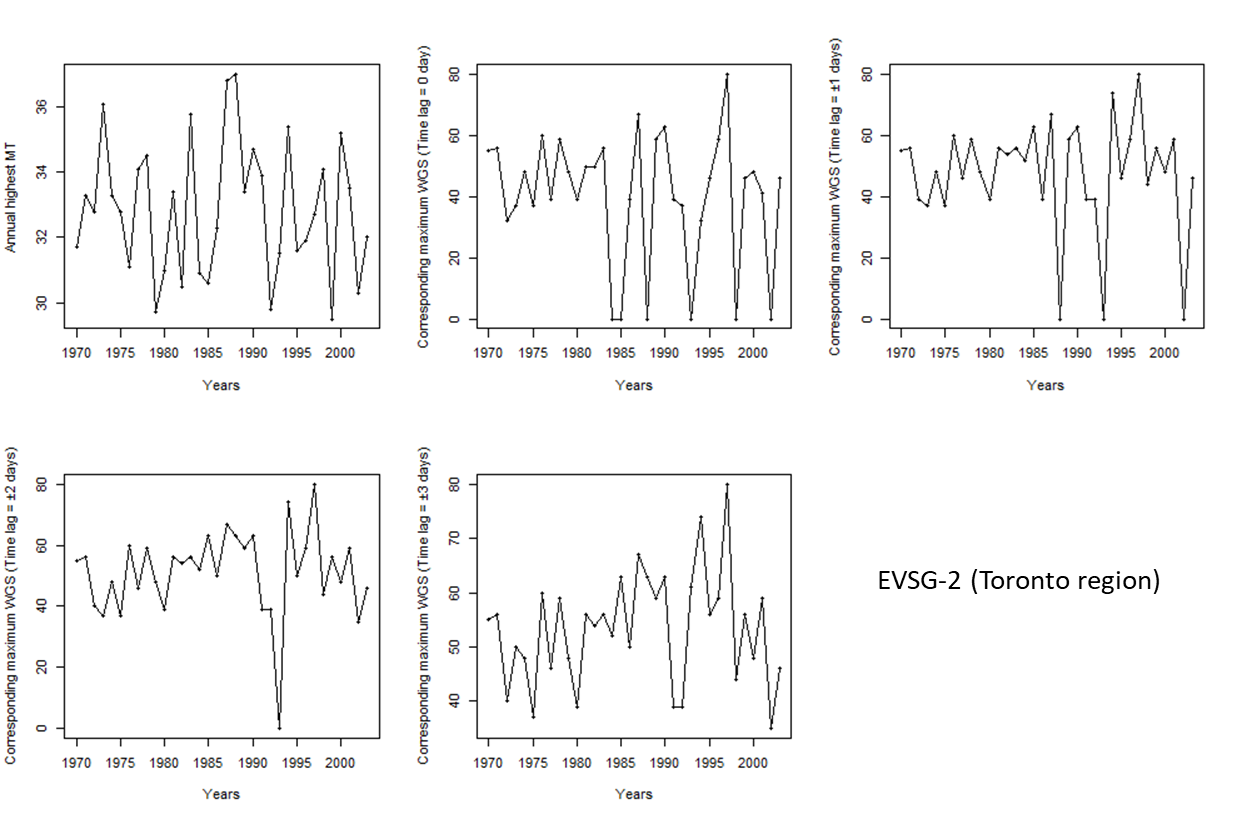


(d)


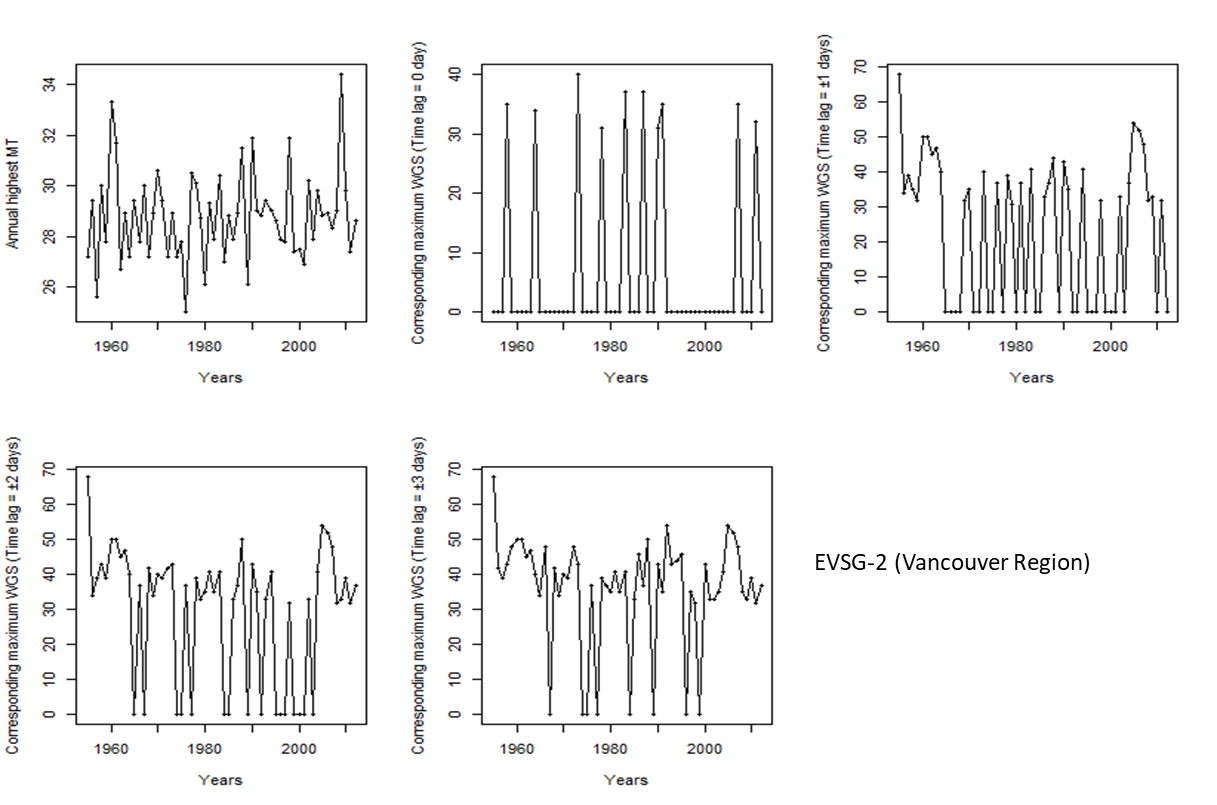
 (e)


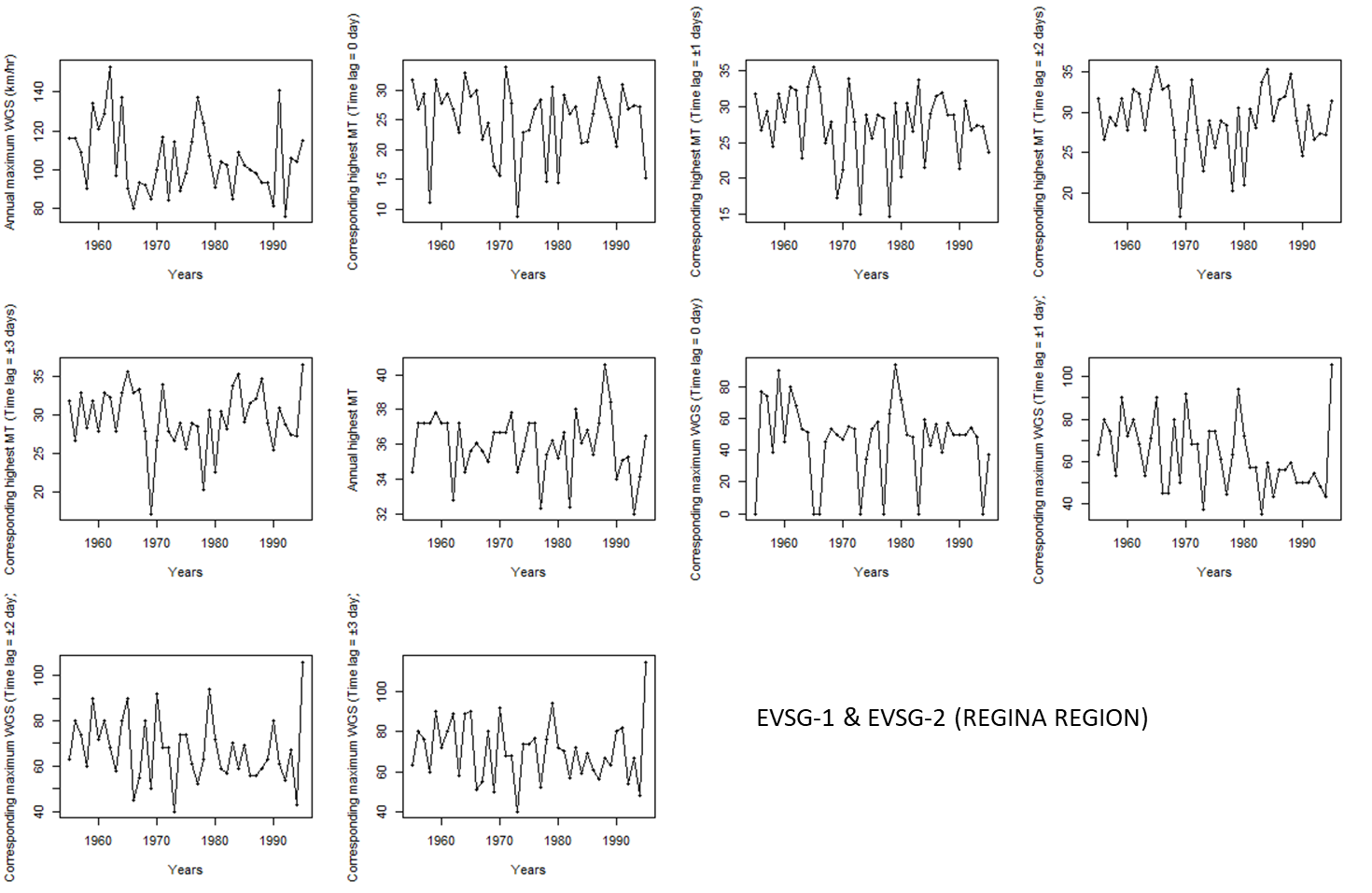
 (f)


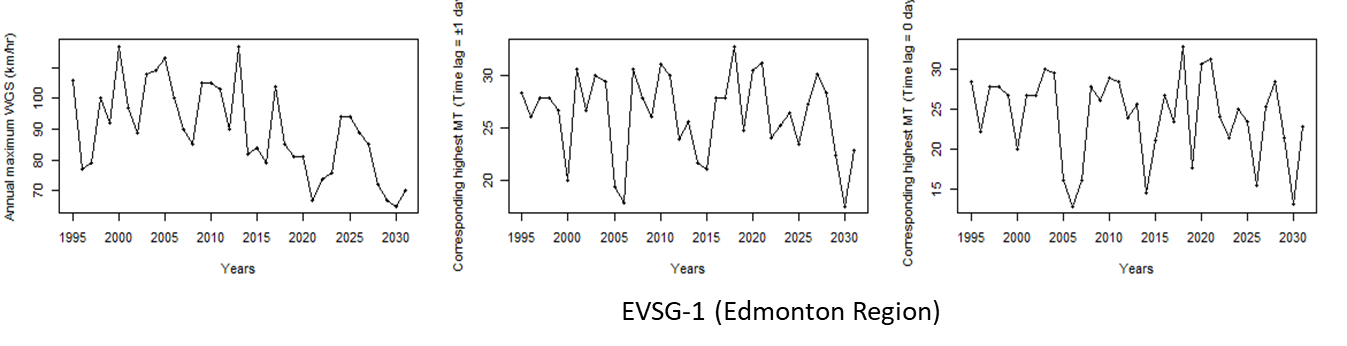
 (g)

Supplementary SF 4.2: Stationwise time series plots (a-1, a-2) Montreal (b) Quebec City (c) Ottawa (d) Toronto (e) Vancouver (f) Regina (g) Edmonton


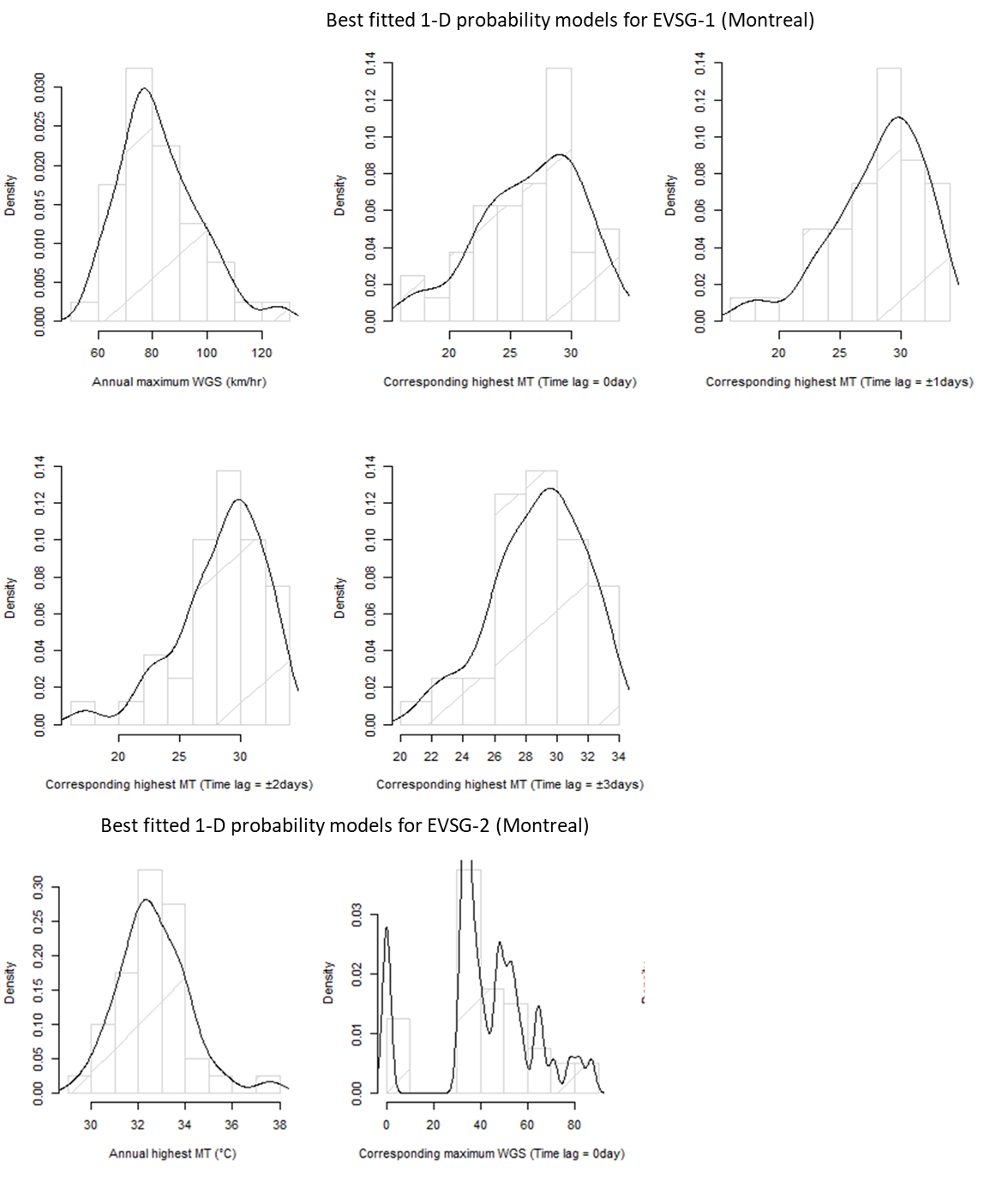
 (a)


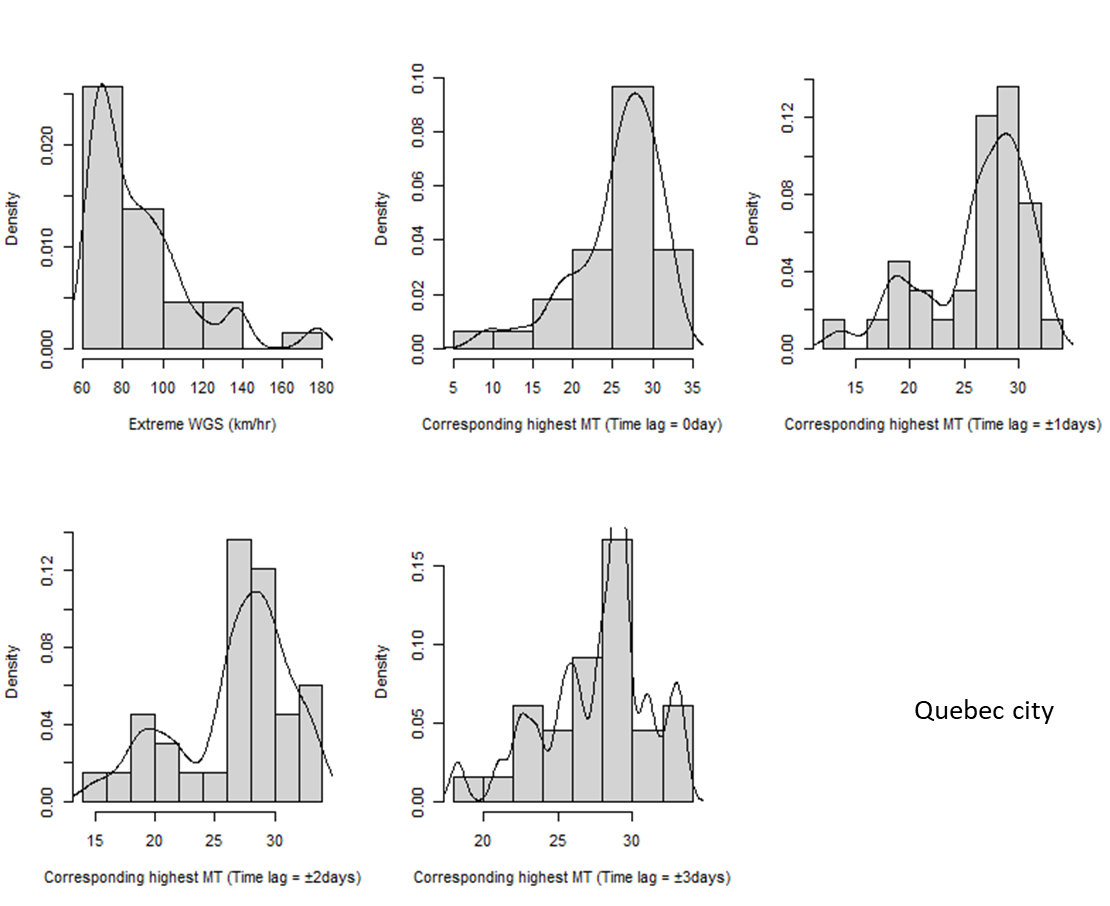
(b)


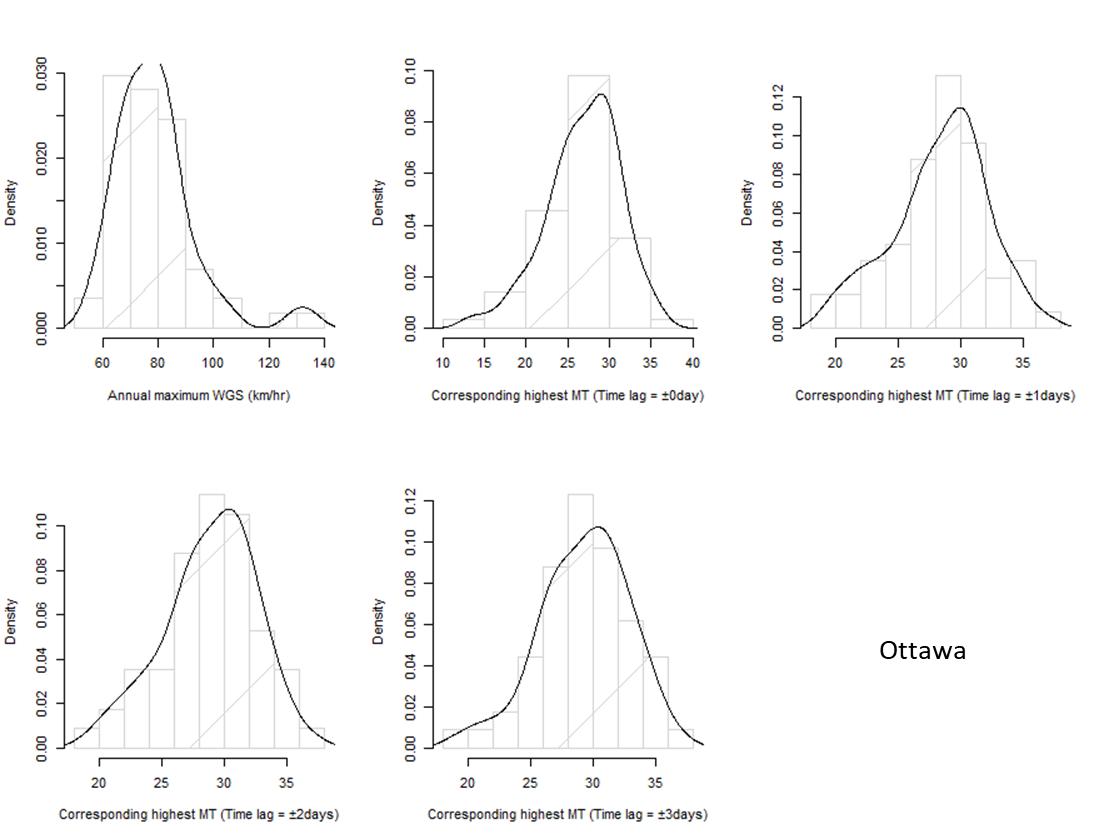
(c)


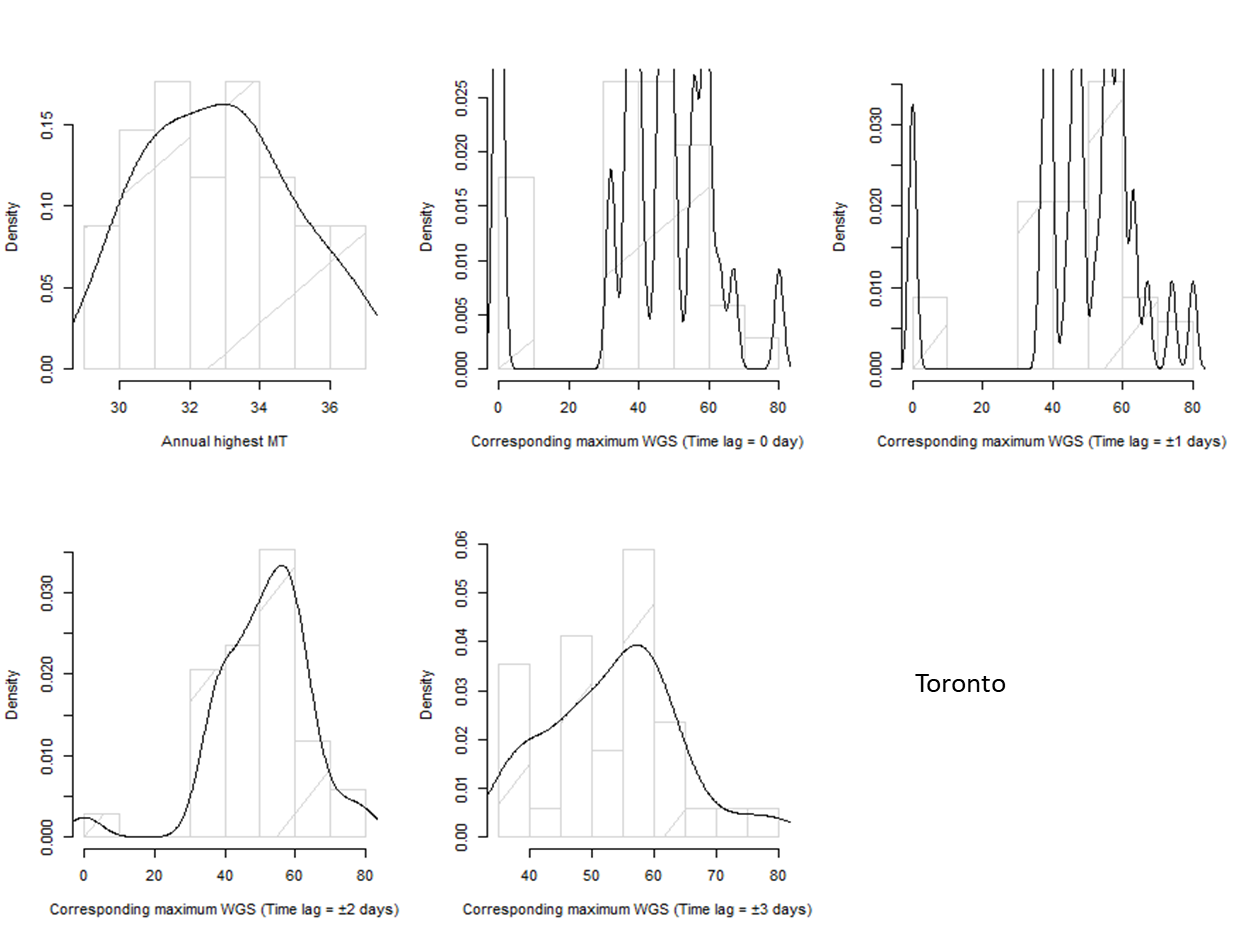
(d)


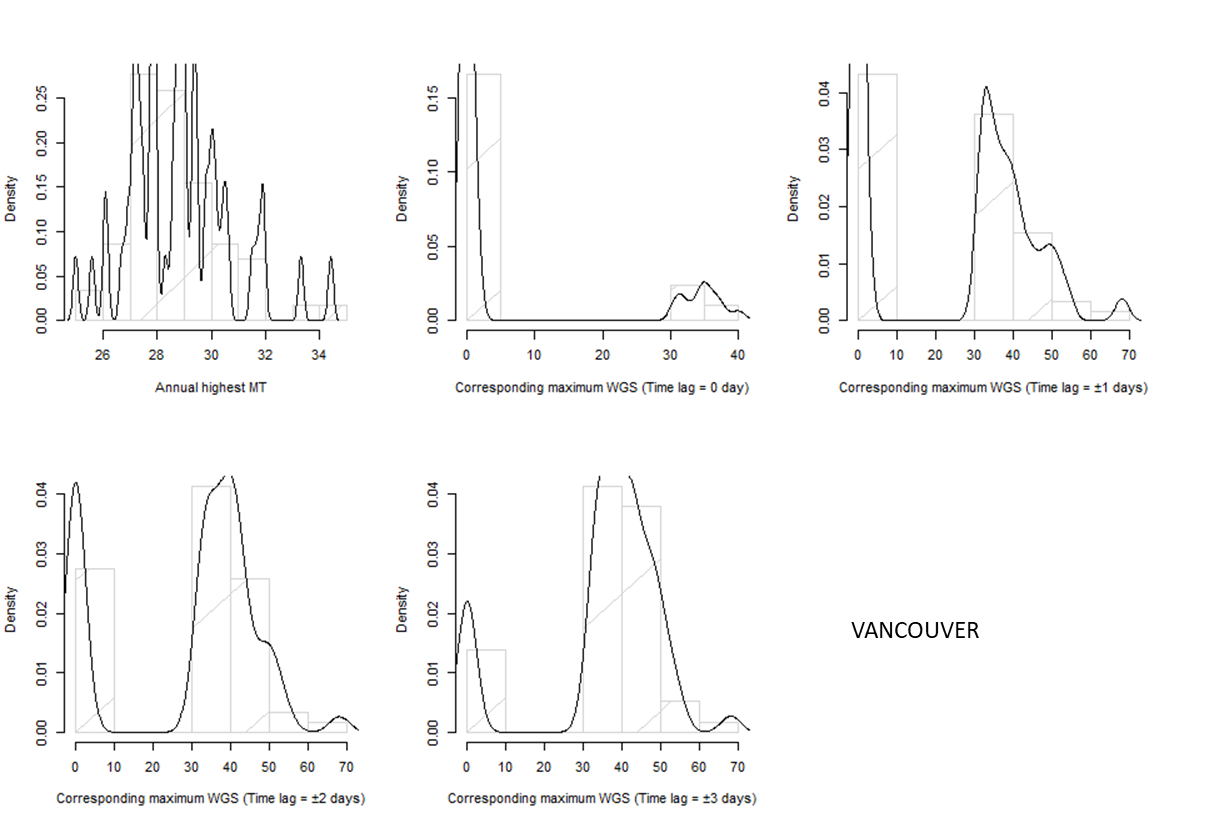
(e)


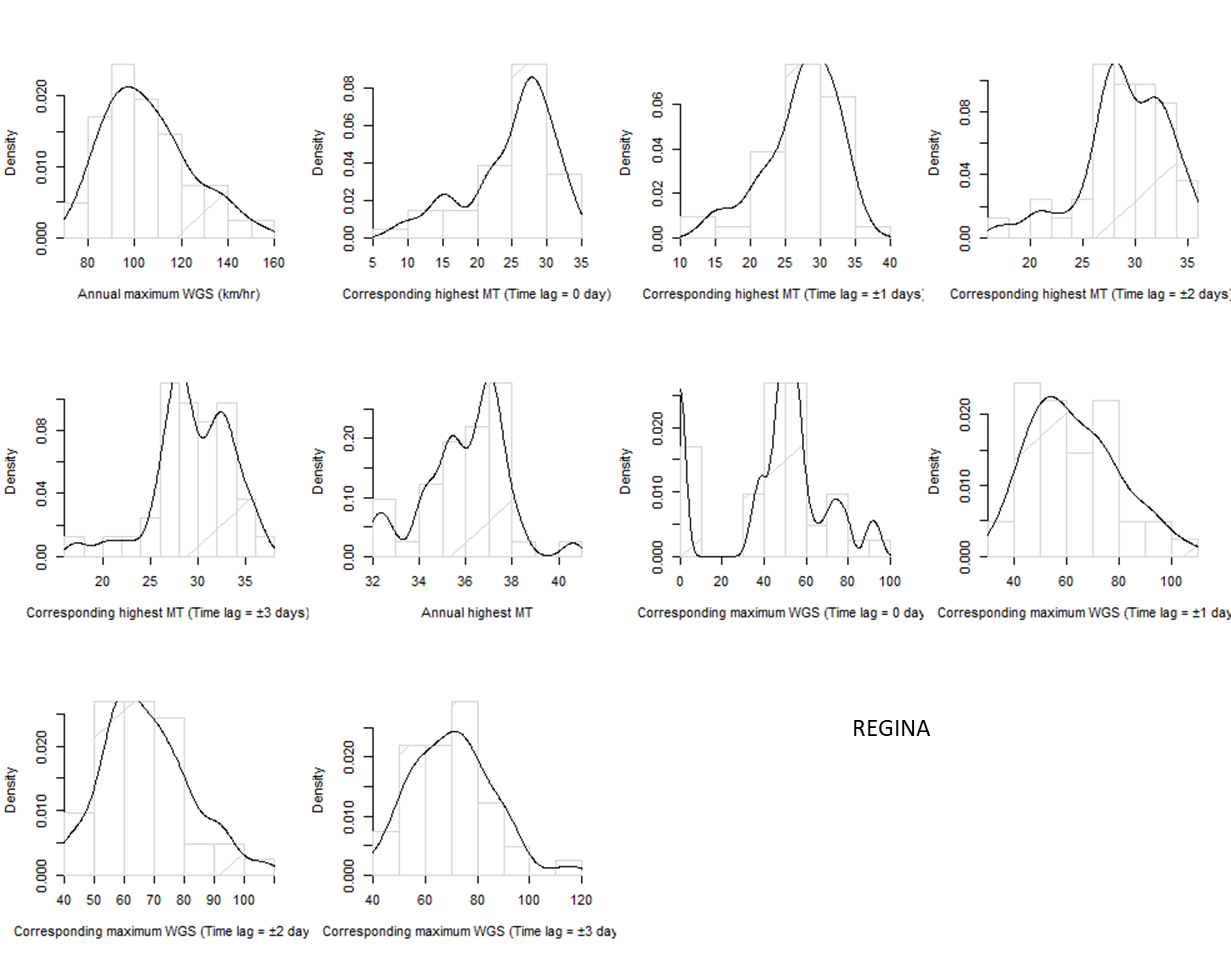
(f)


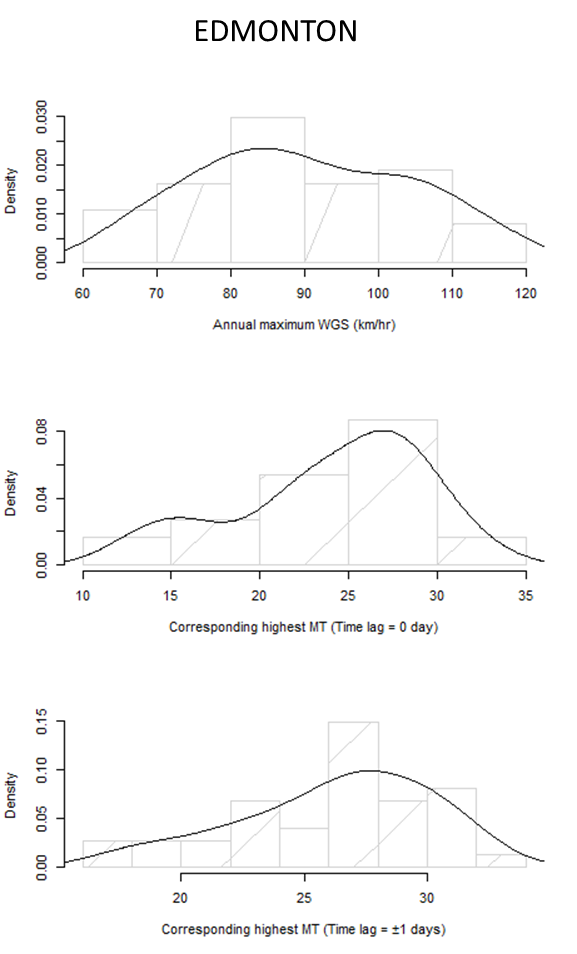
(g)

**Supplementary SF 5:** Probability density functions (PDFs) of the most justifiable nonparametric GKDE models fitted to selected univariate series of EVSG-1/2 at (a) Montreal (b) Quebec City (c) Ottawa (d) Toronto (e) Vancouver (f) Regina (g) Edmonton


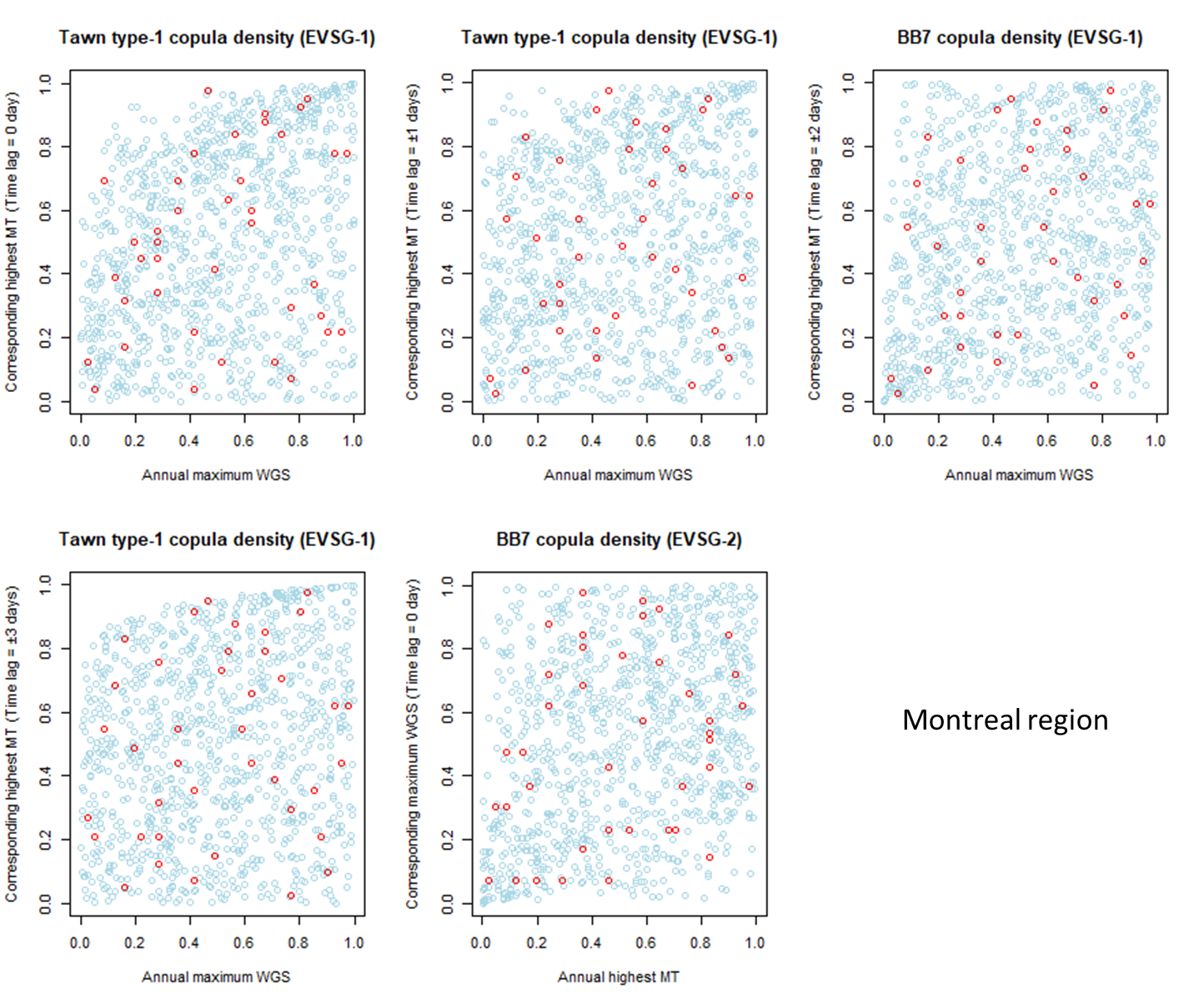
(a)


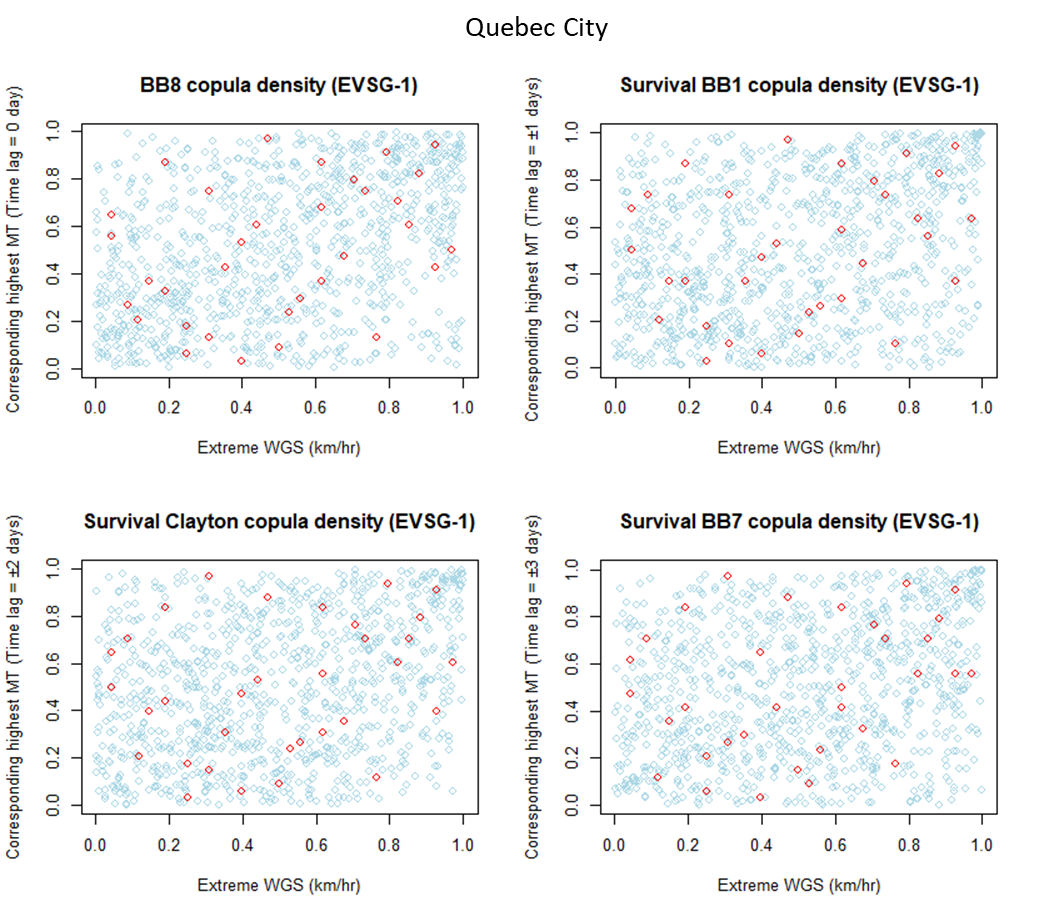
 (b)


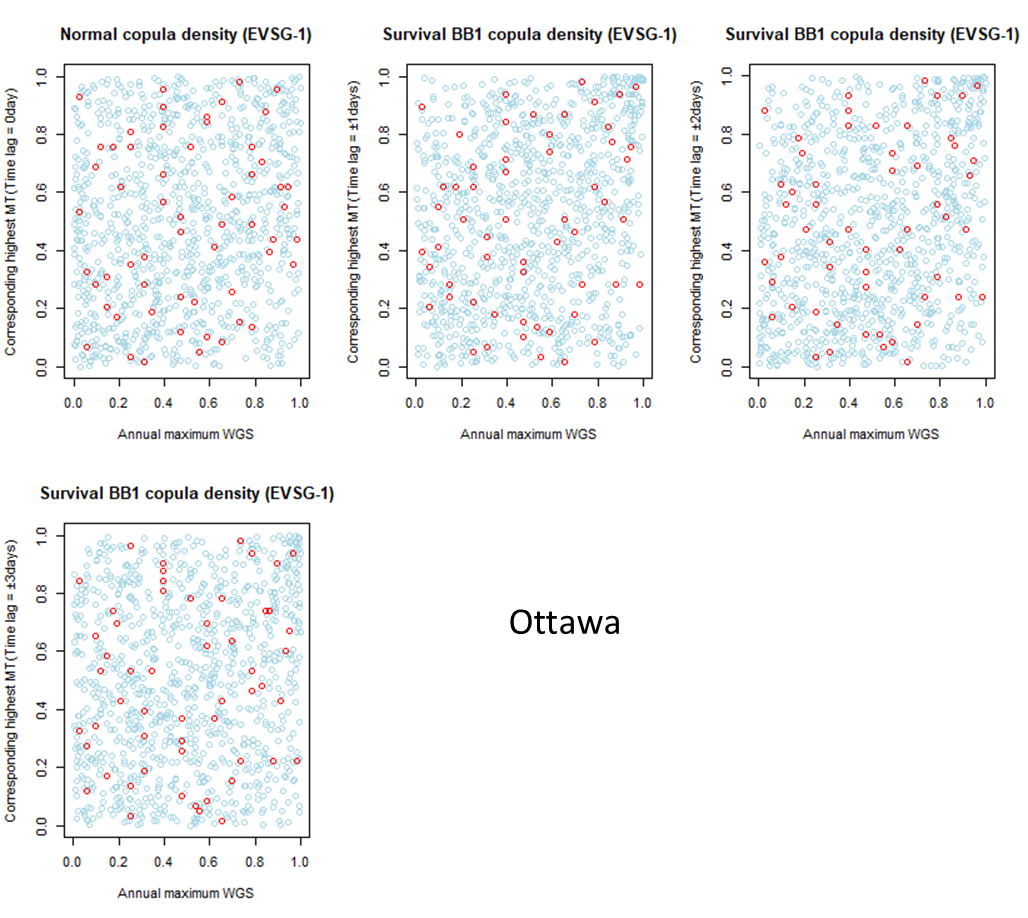
(c)


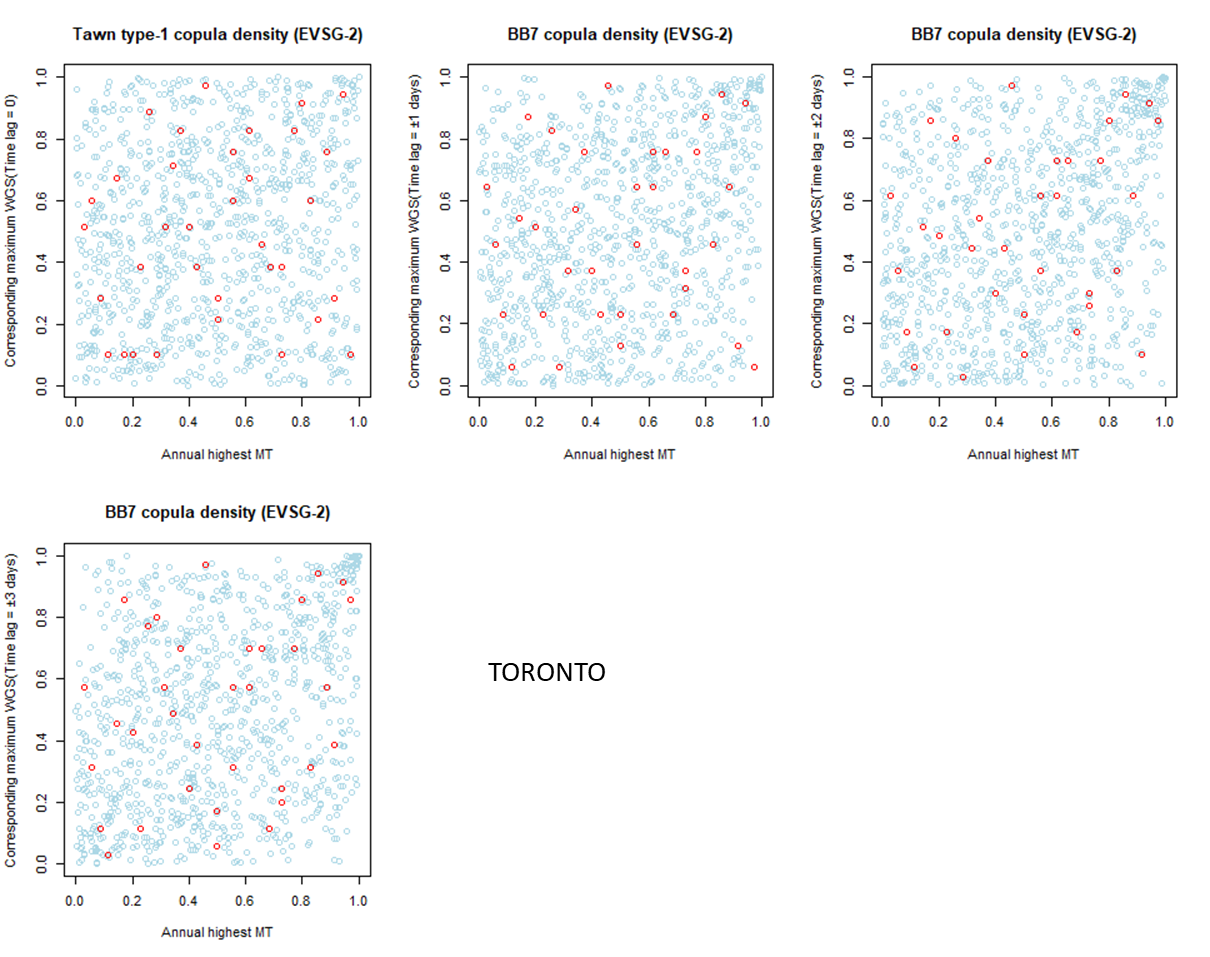
(d)


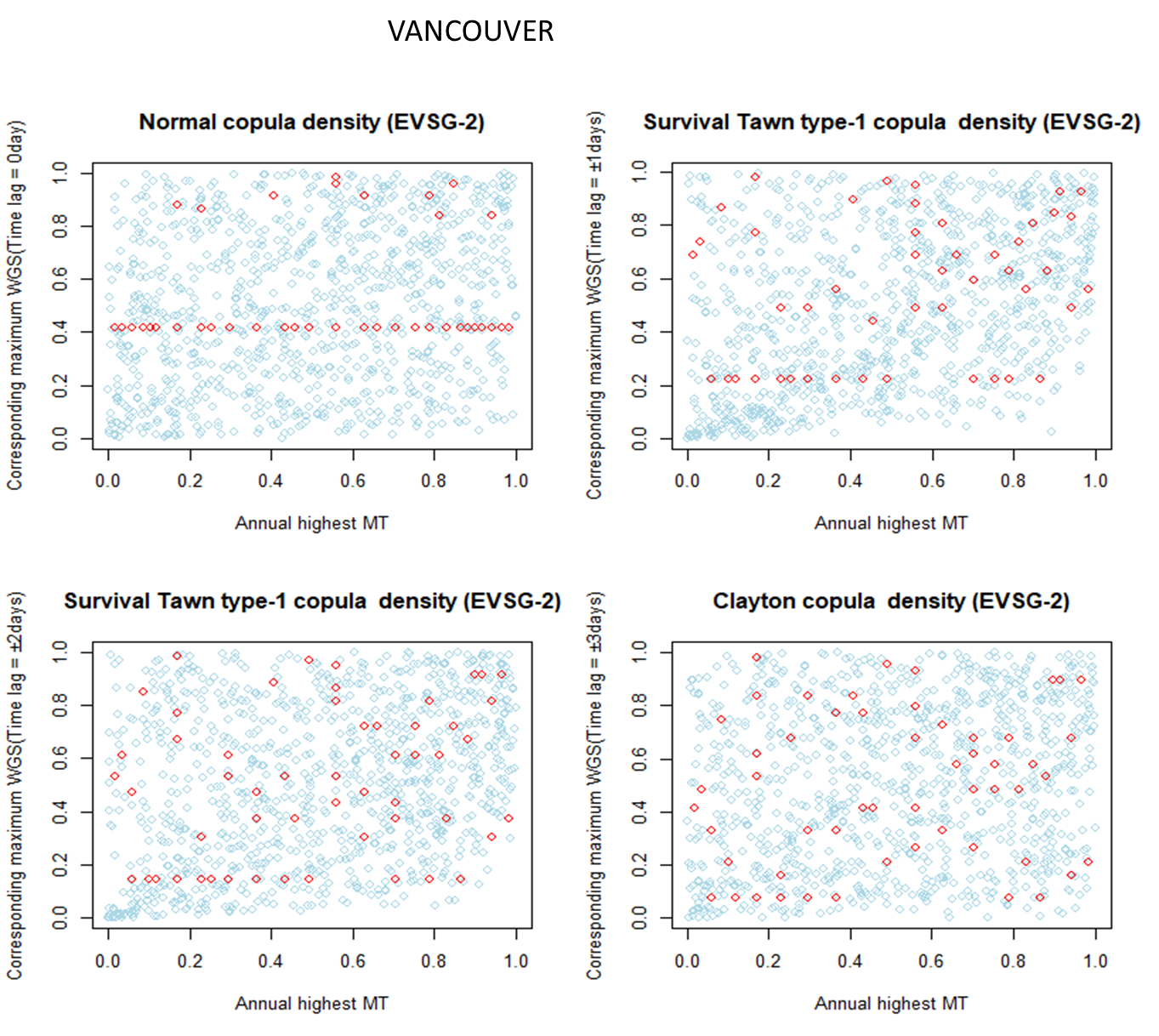
(e)


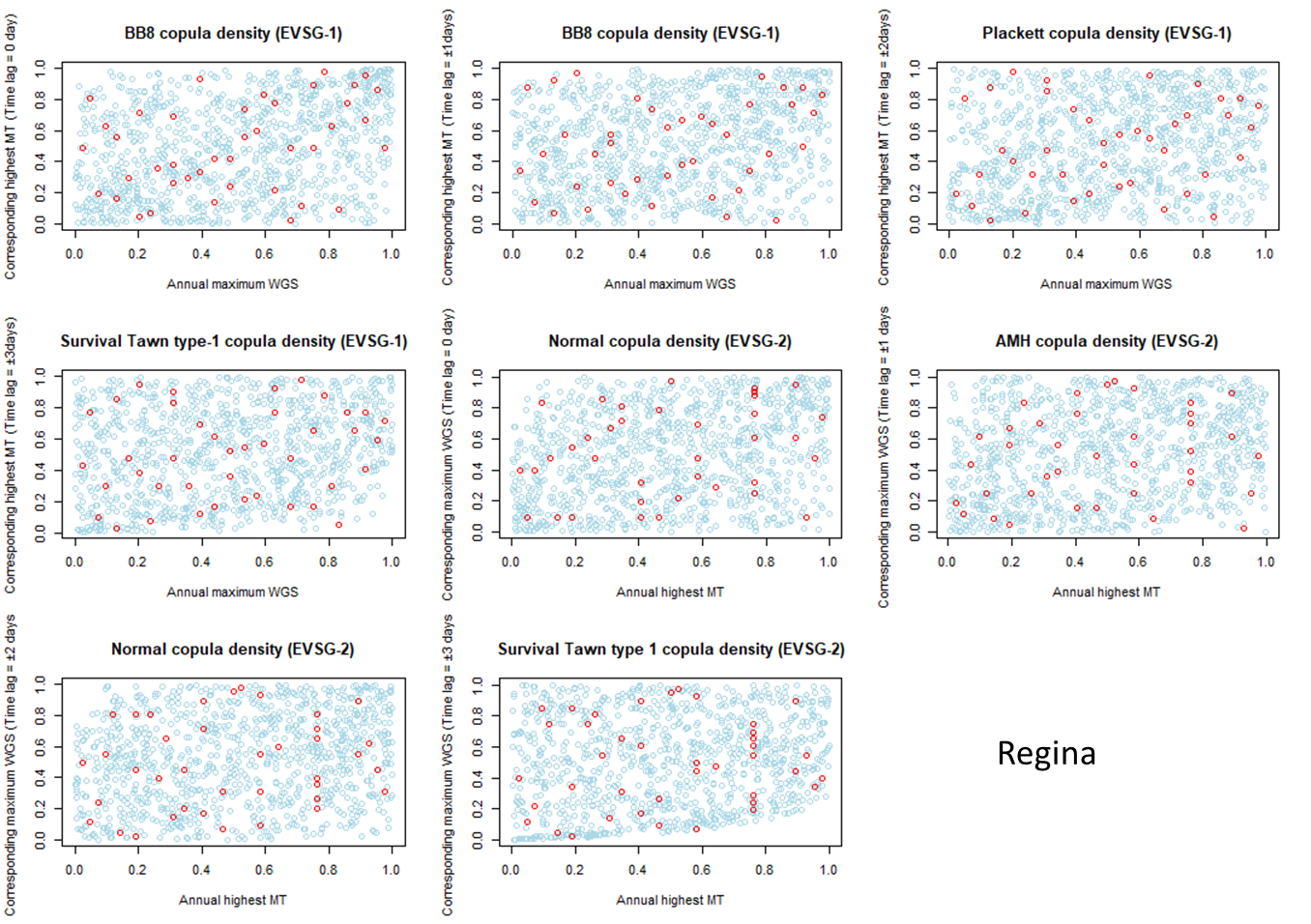


(f)


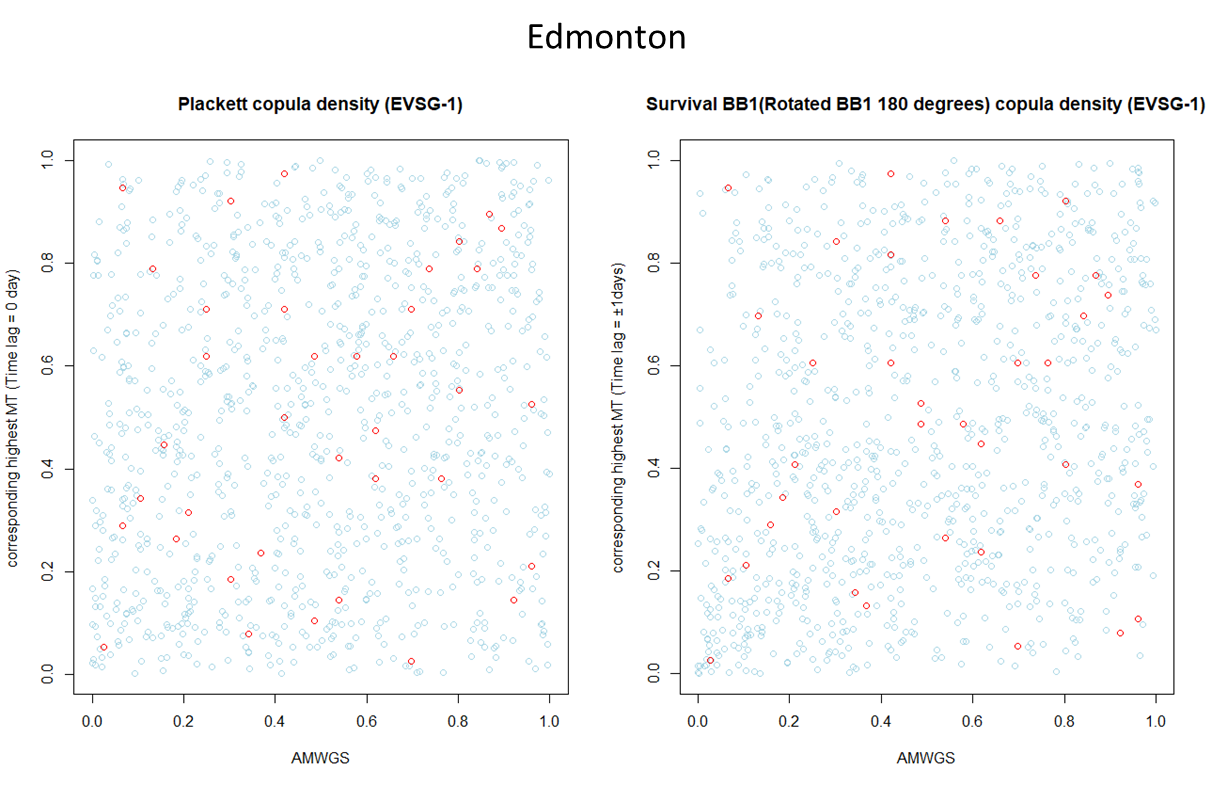
(g)

**Supplementary SF 6:** Visual comparison based on the 2-D scatter plot between bivariate observed (red colour) extreme pairs with set of 1000 random observations simulated (light blue colour) from best-fitted bivariate 2-D copulas for each selected bivariate extreme pairs of selected cities using the MPL estimator. For station (a) Montreal (b) Quebec City (c) Ottawa (d) Toronto (e) Vancouver (f) Regina (g) Edmonton


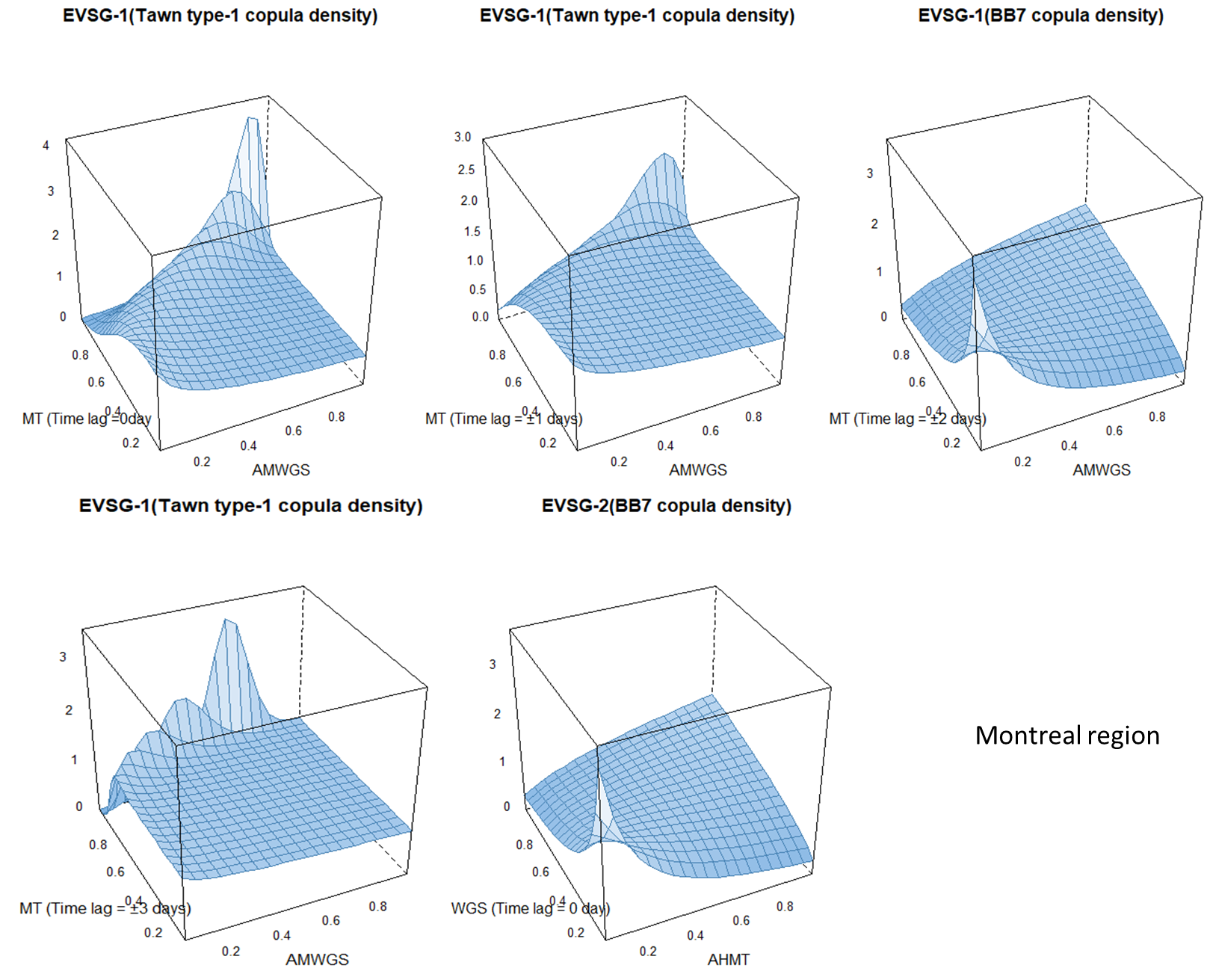
(a)


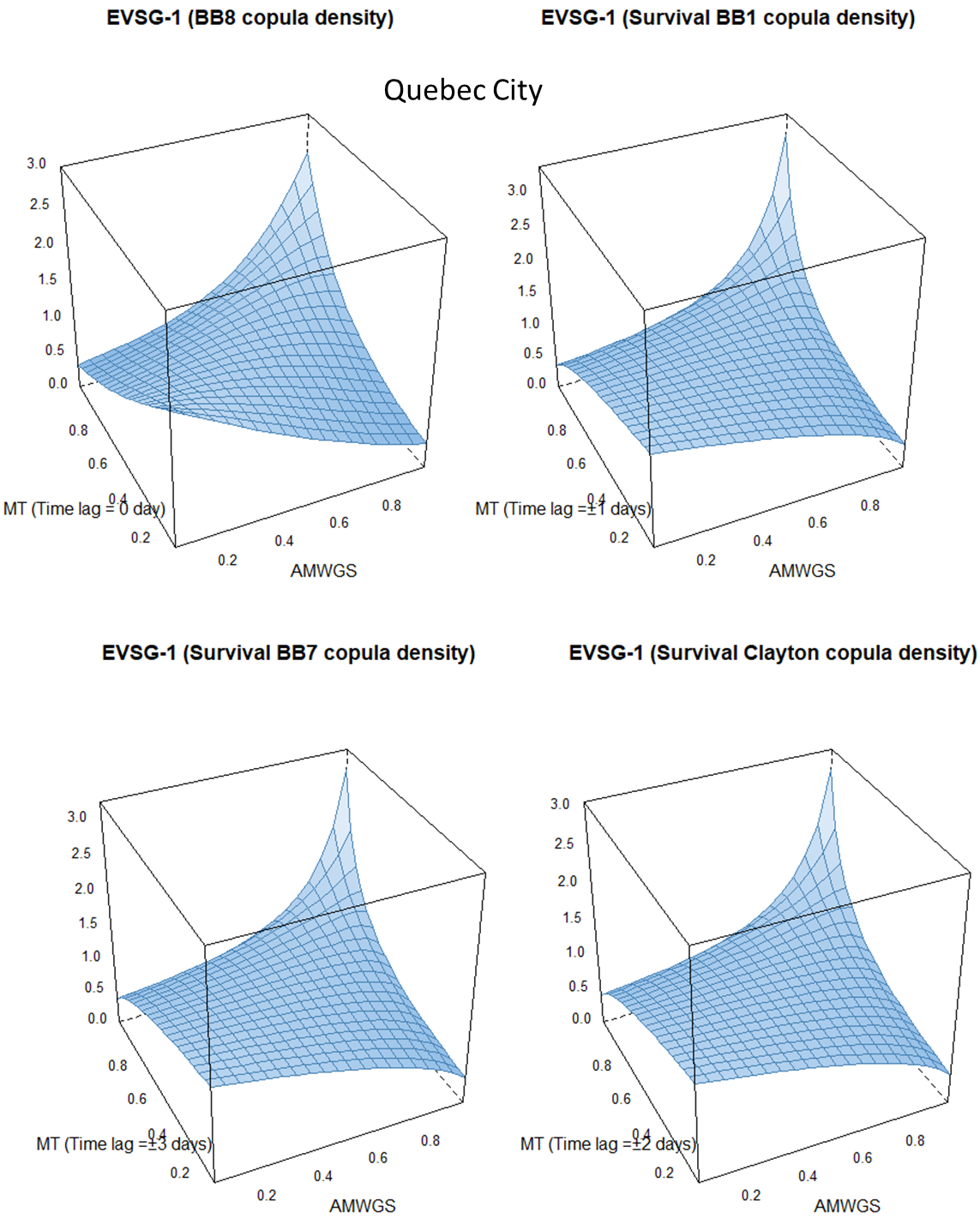
 (b)


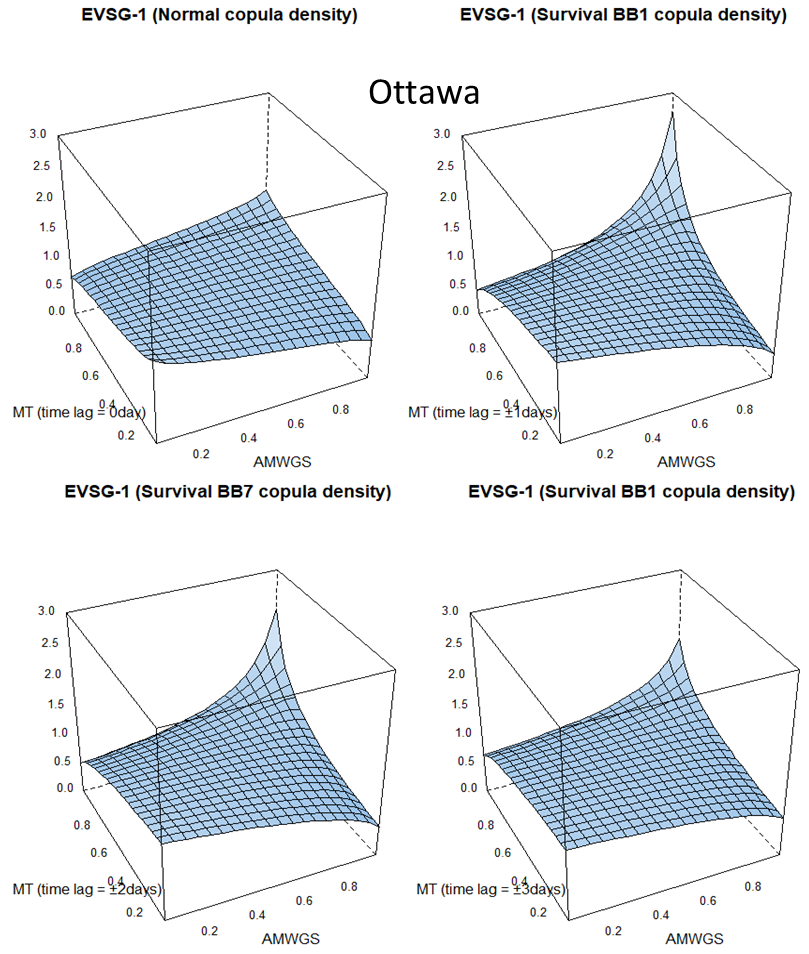
(c)


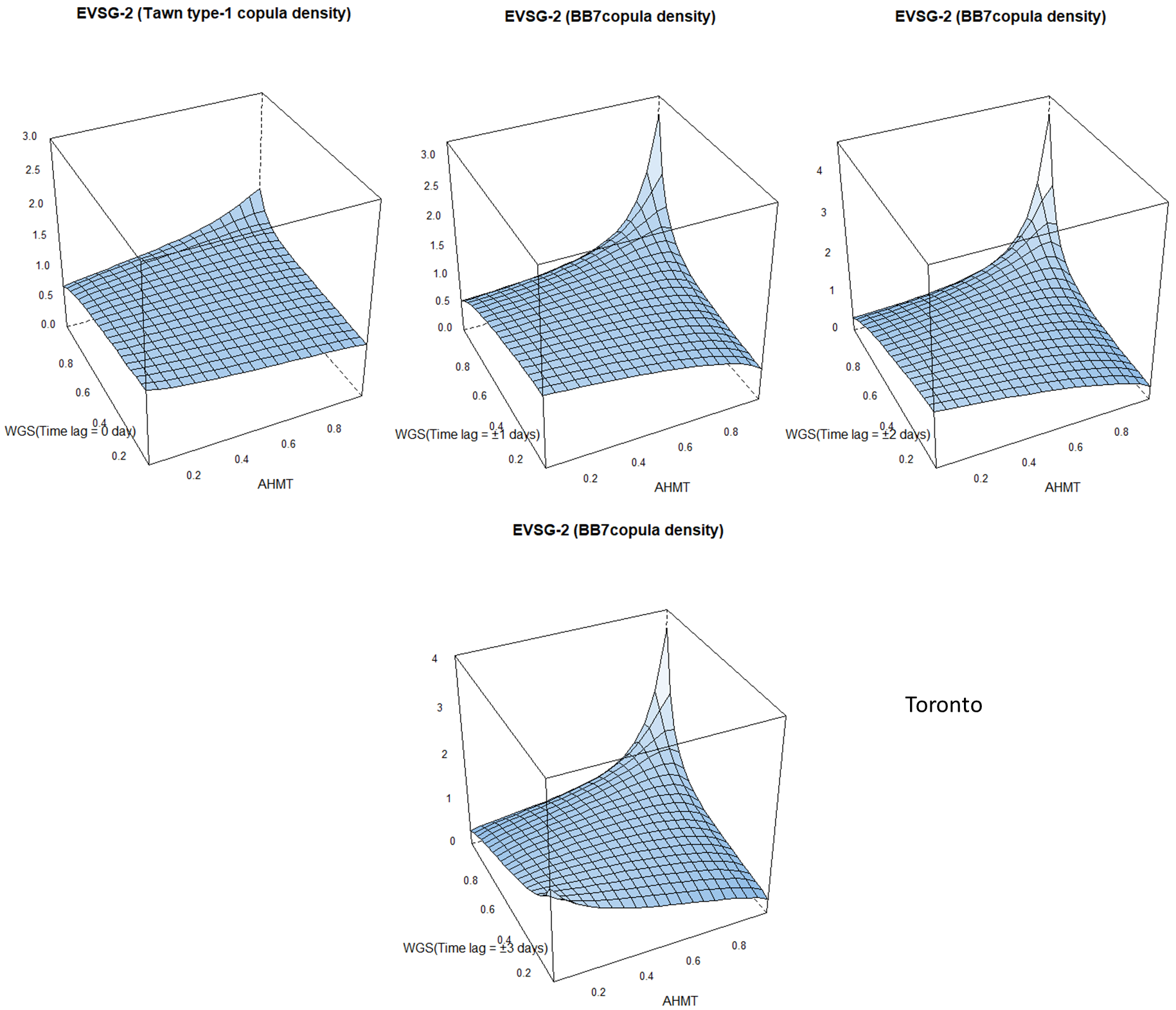
 (d)


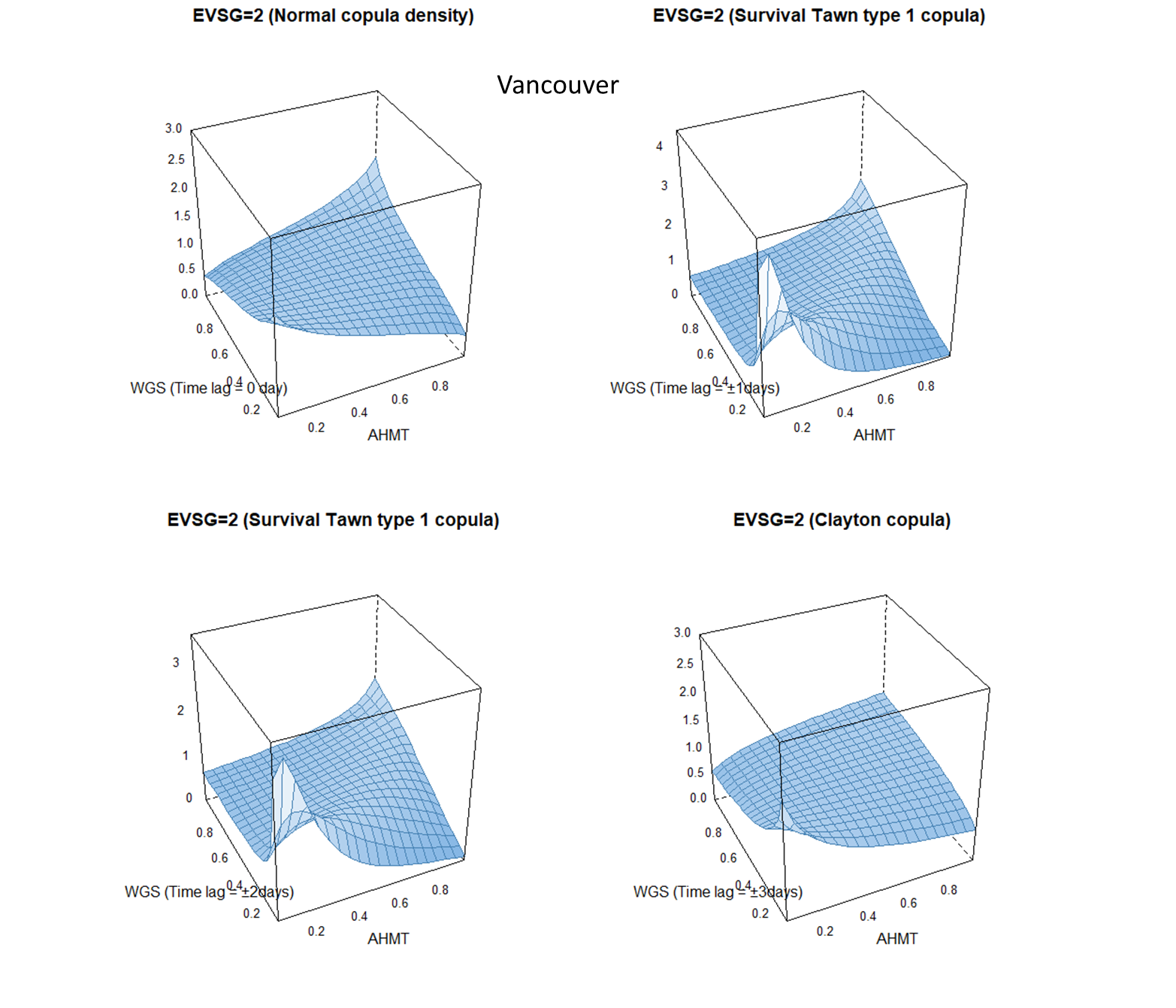
(e)


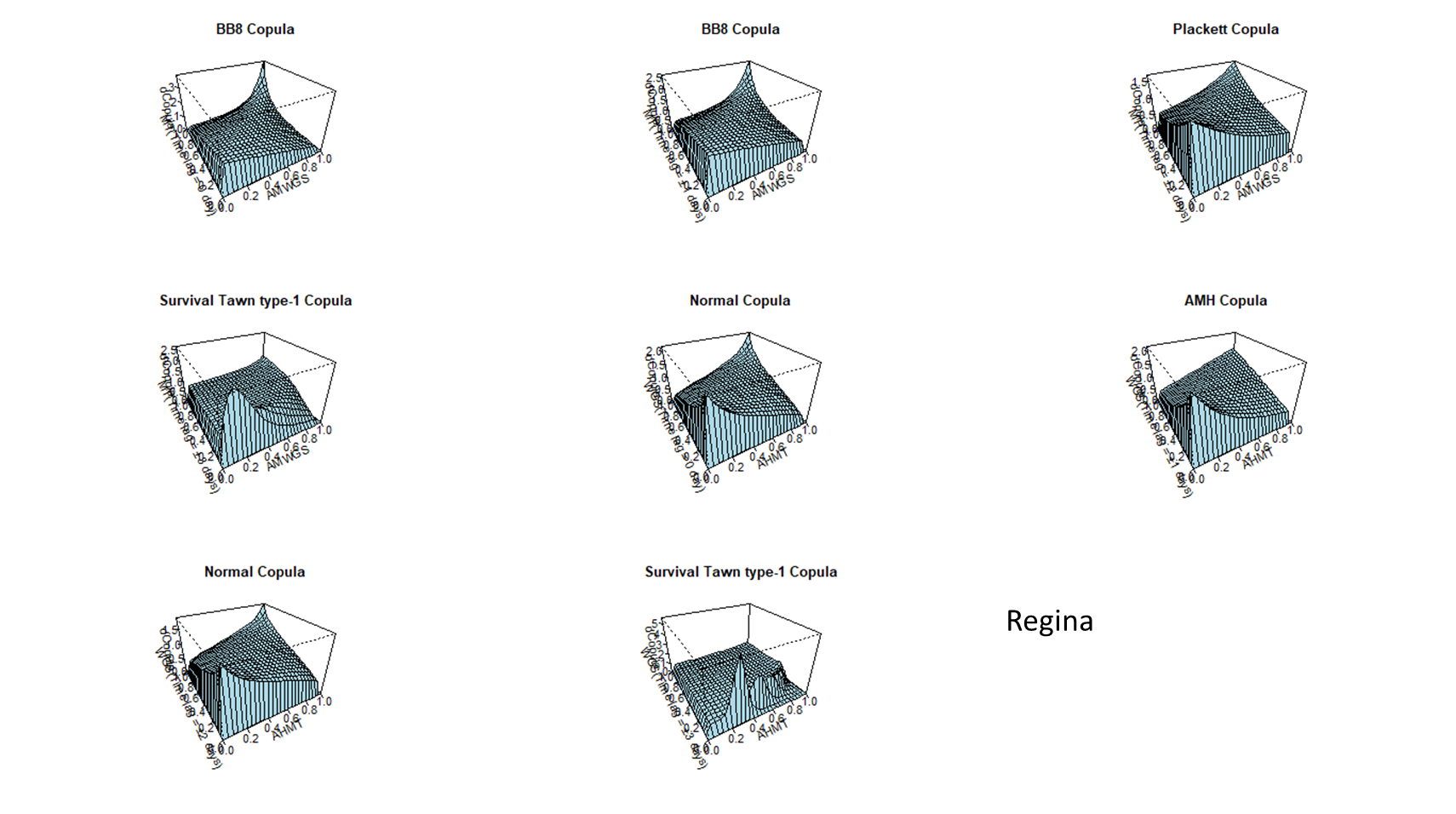
(f)


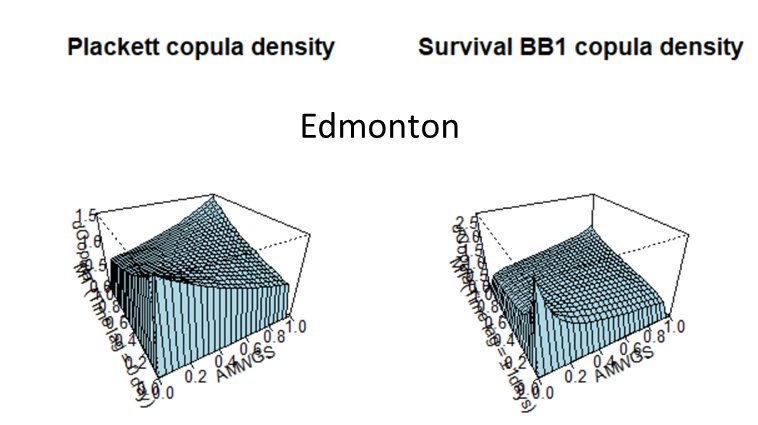
 (g)

**Supplementary SF7:** Illustrating surface density of best-fitted copula density selected for station (a) Montreal (b) Quebec City (c) Ottawa (d) Toronto (e) Vancouver (f) Regina (g) Edmonton


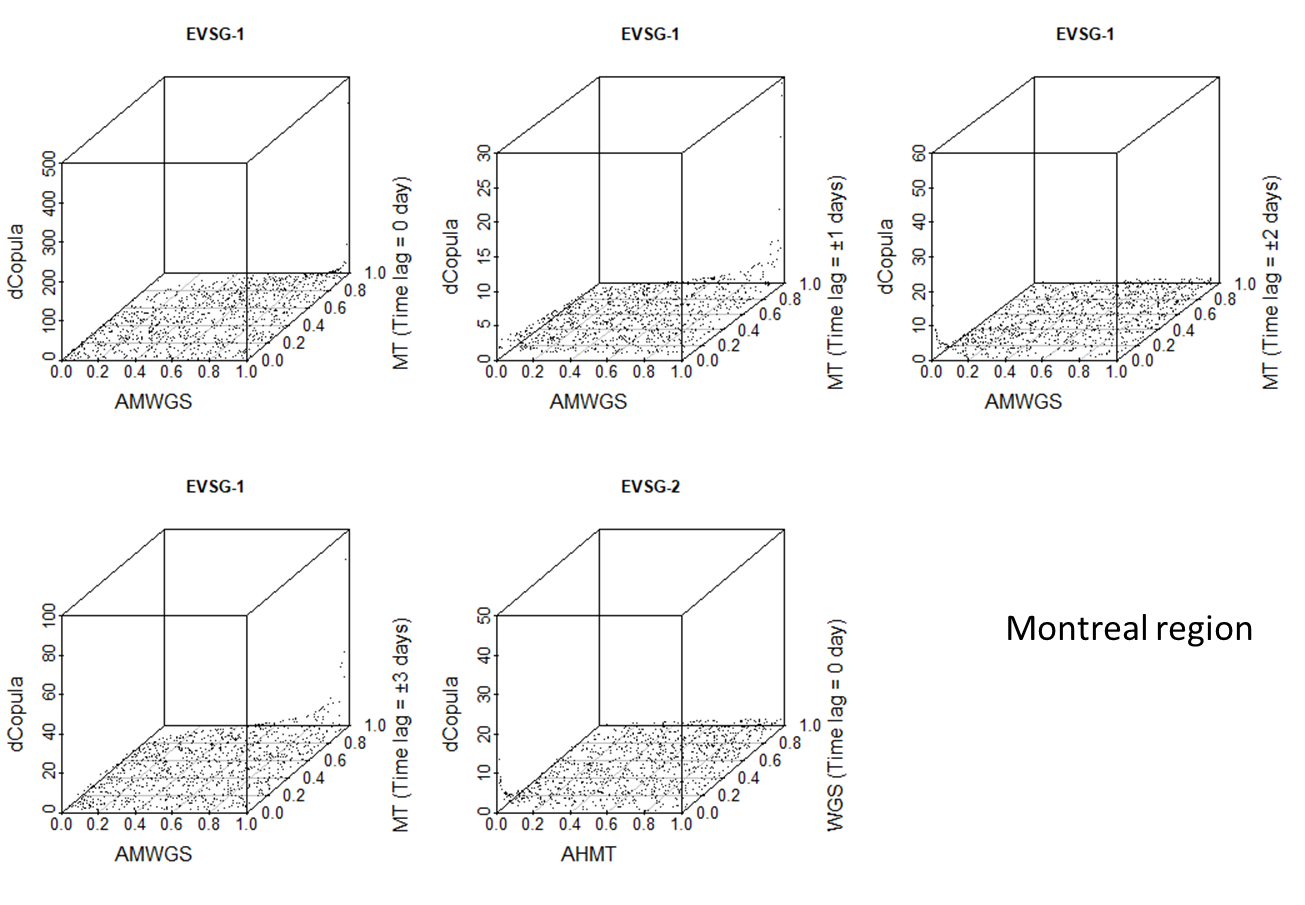
(a)


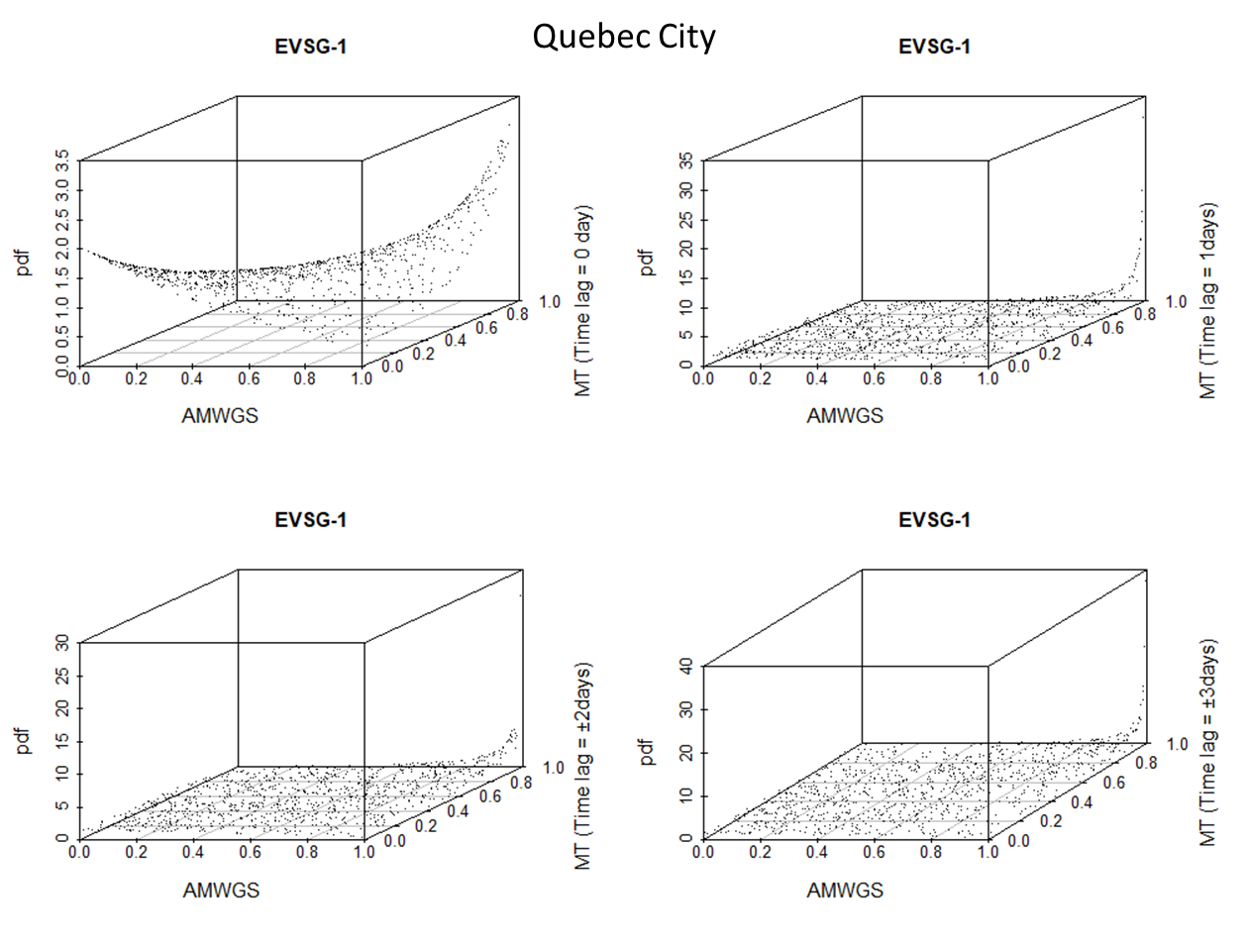
 (b)


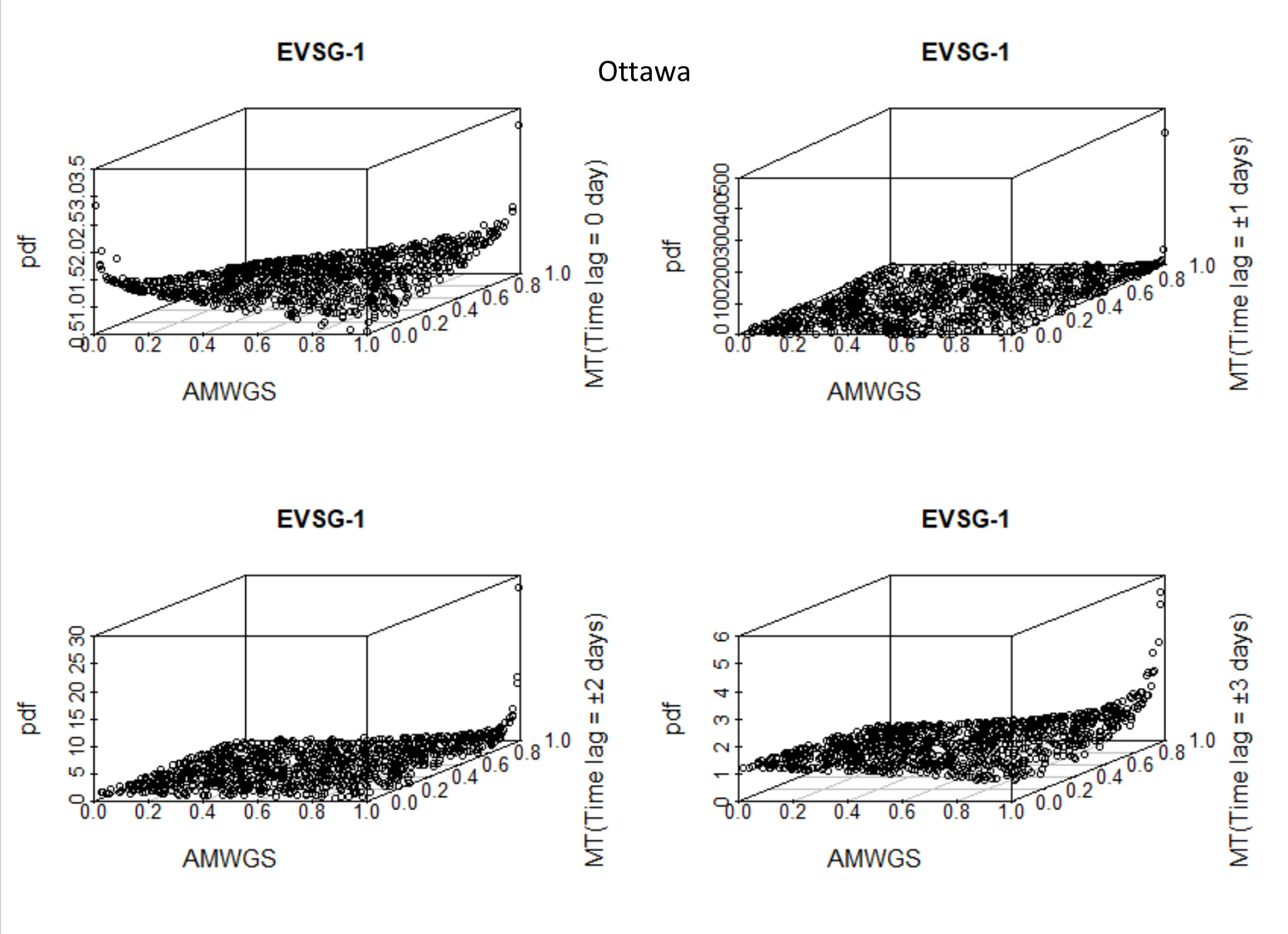
 (c)


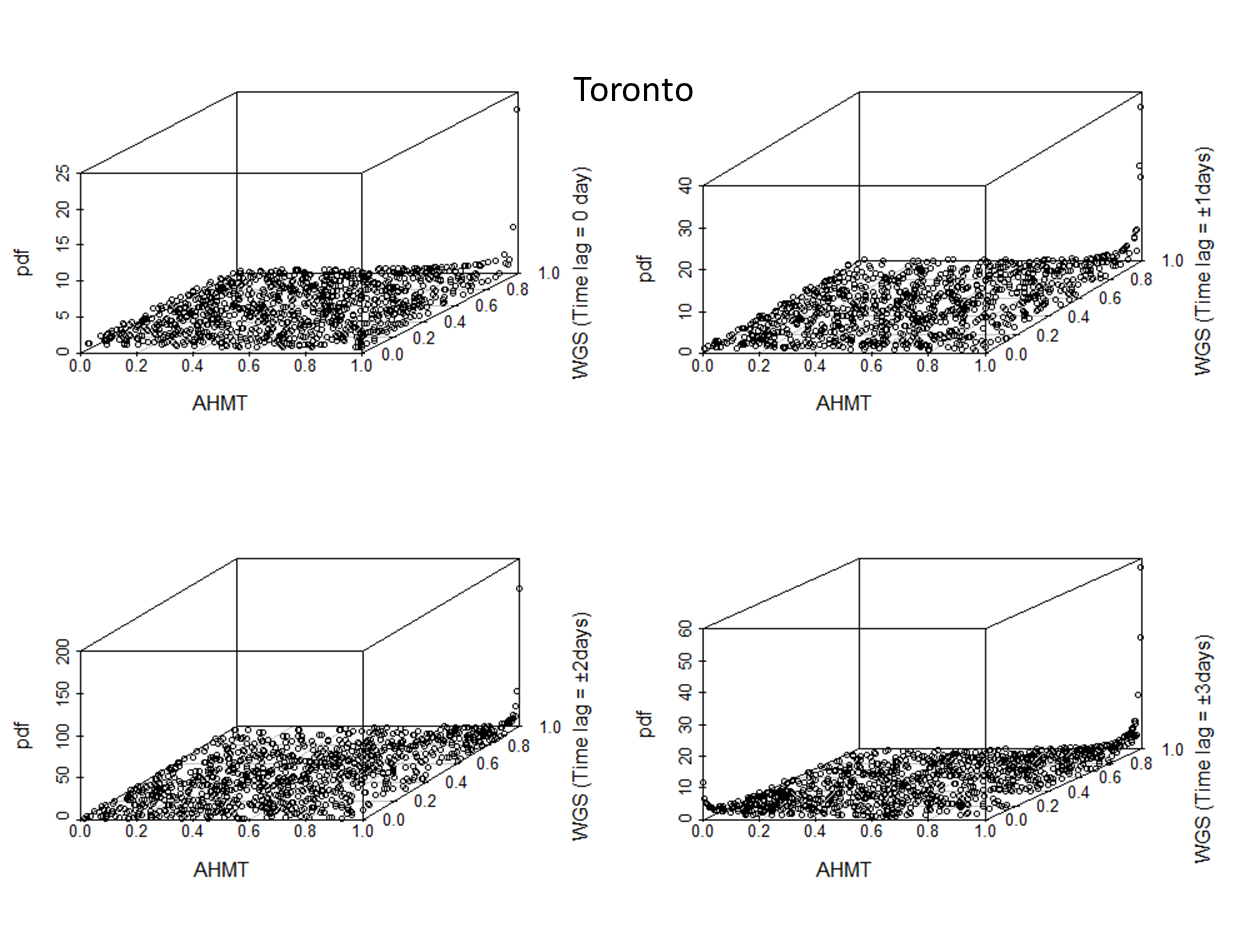
(d)


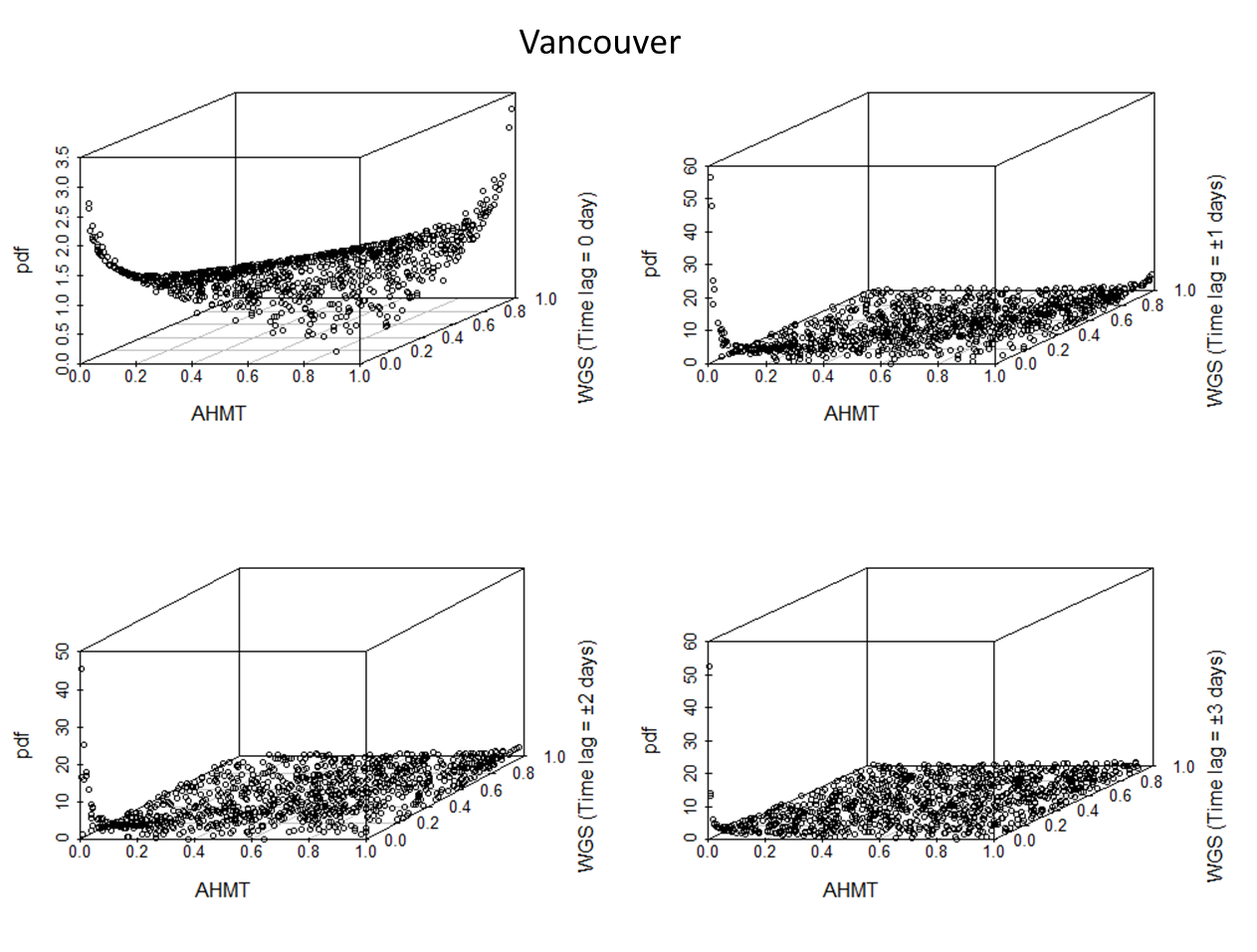
 (e)


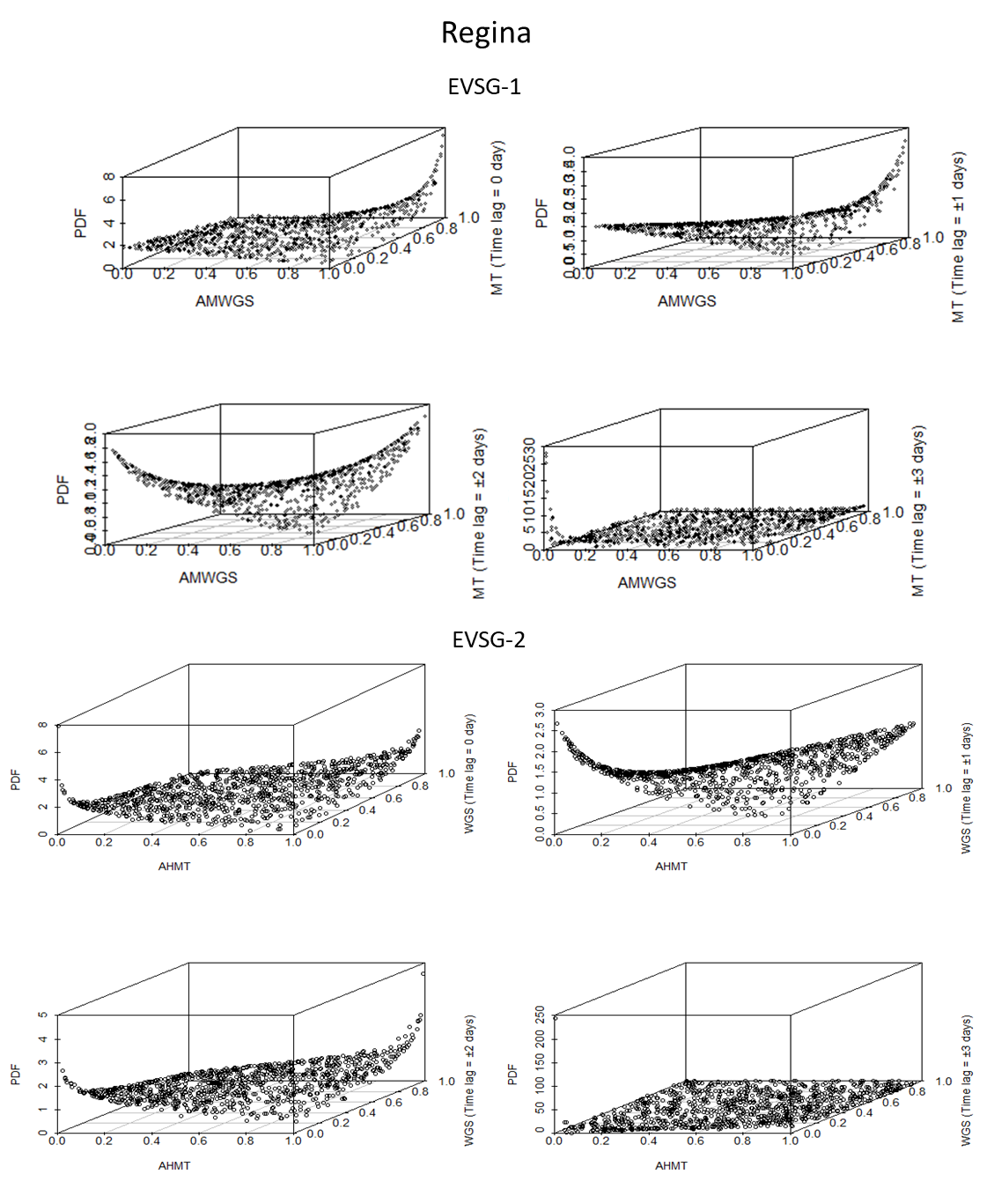
(f)


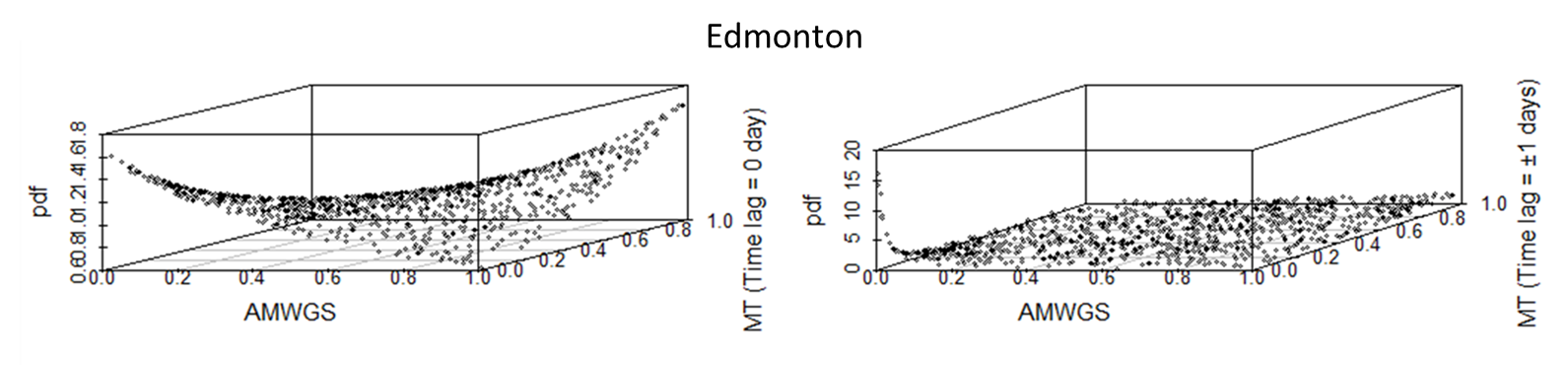
 (g)

**Supplementary SF 8:** Illustrating 3-D scatterplot of joint PDF drawn using 1000 random samples extracted from best-fitted 2-D copula for station (a) Montreal (b) Quebec City (c) Ottawa (d) Toronto (e) Vancouver (f) Regina (g) Edmonton

**
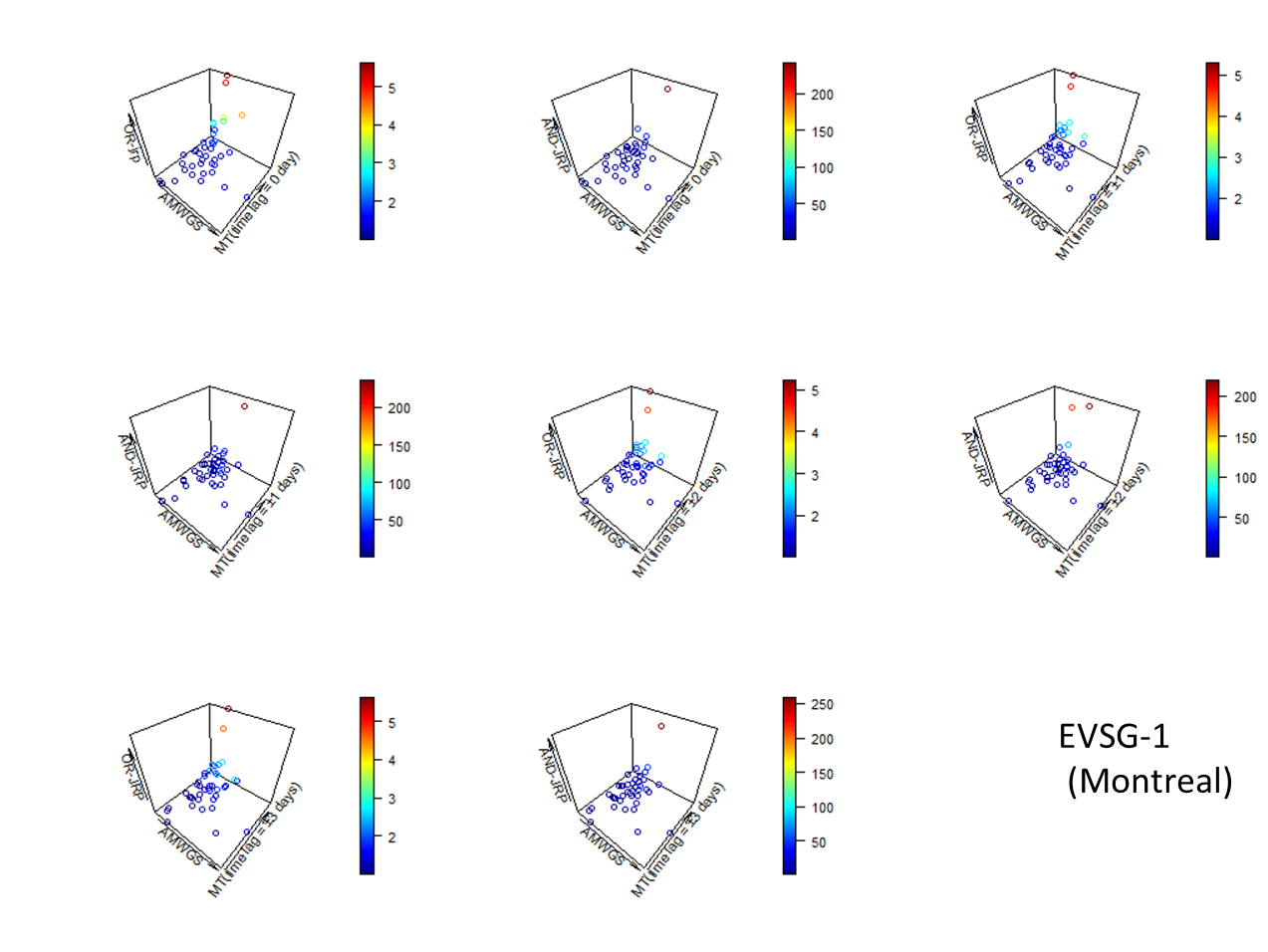
(a-1)**

**
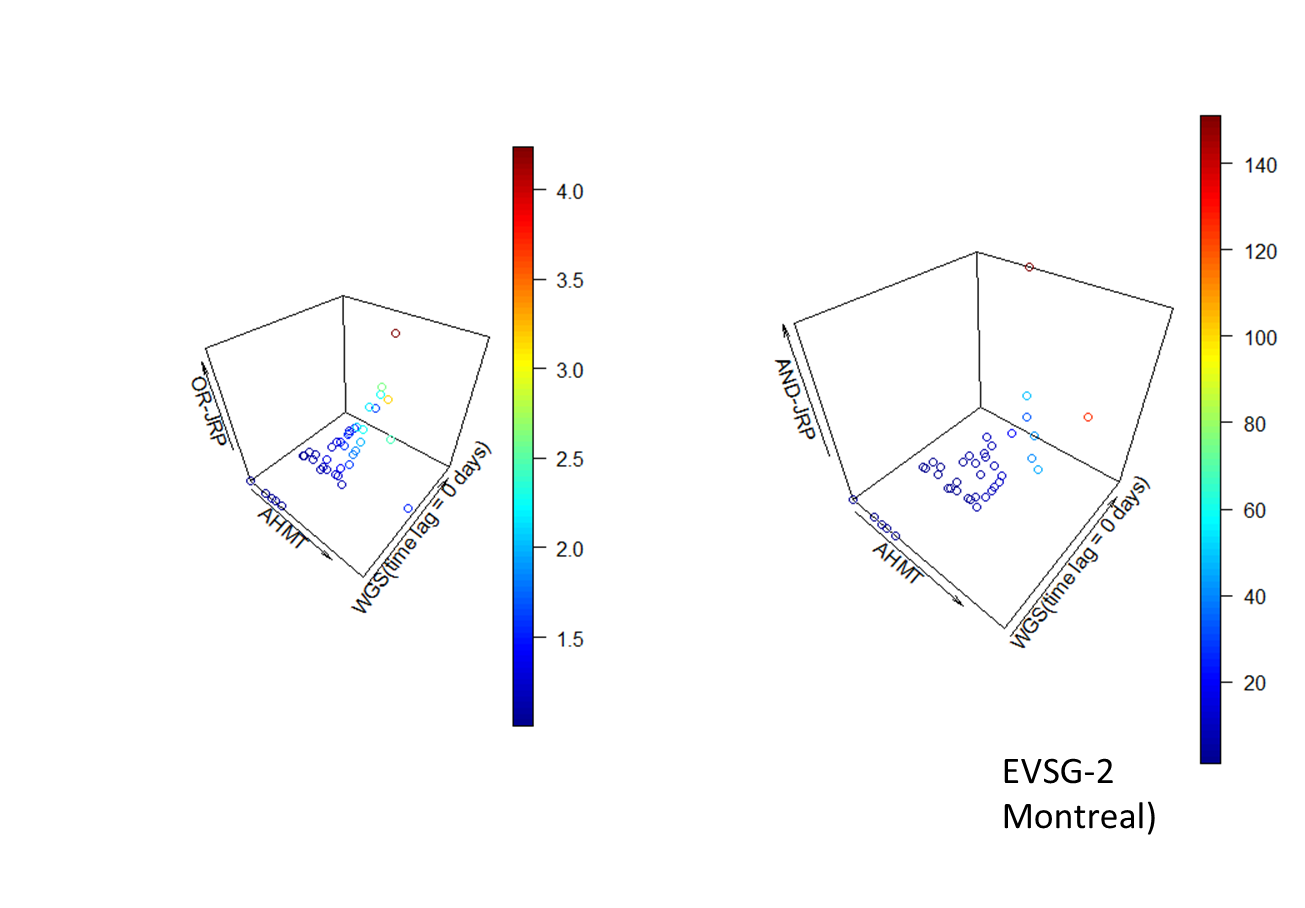
(a-2)**

**
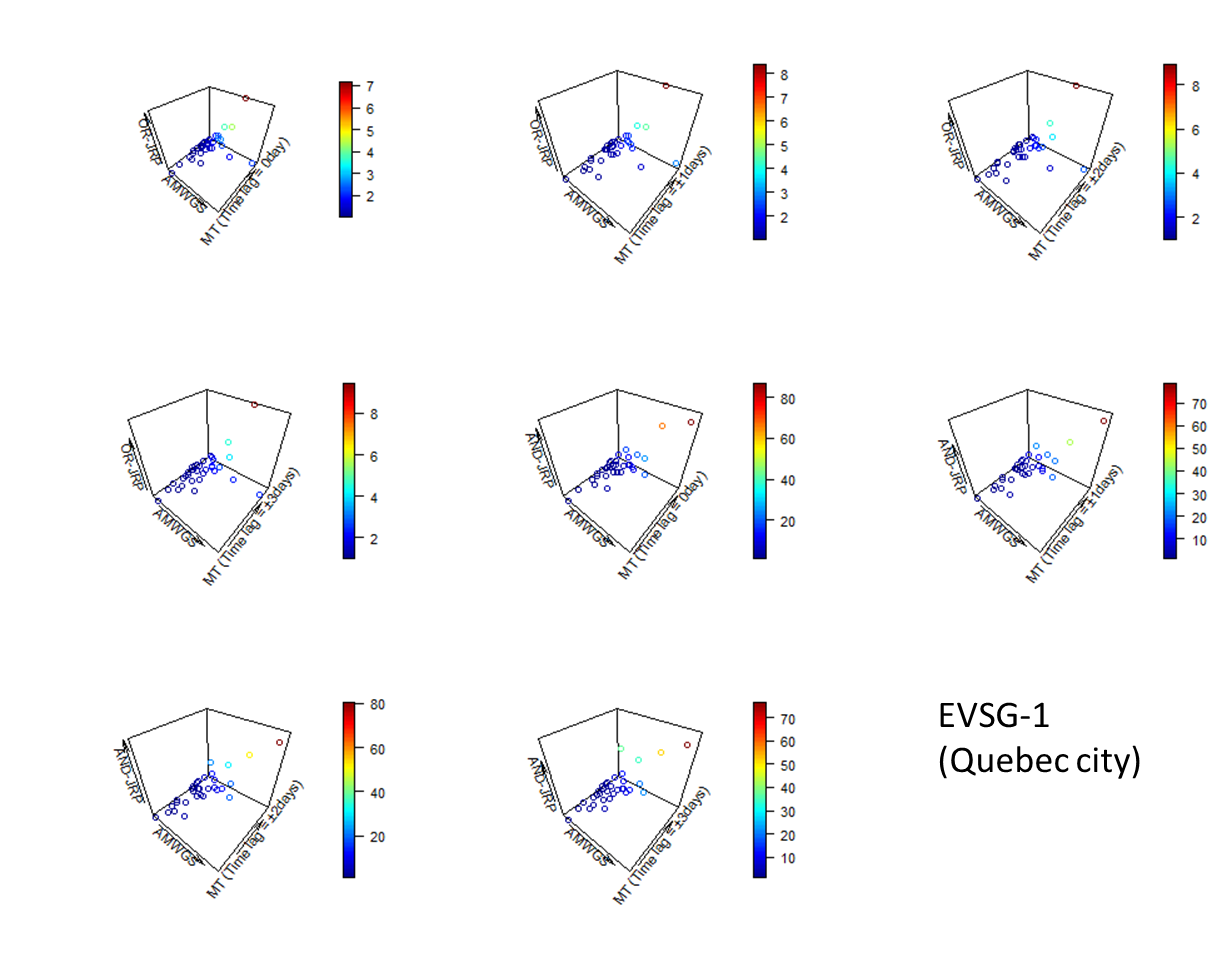
(b)**

**
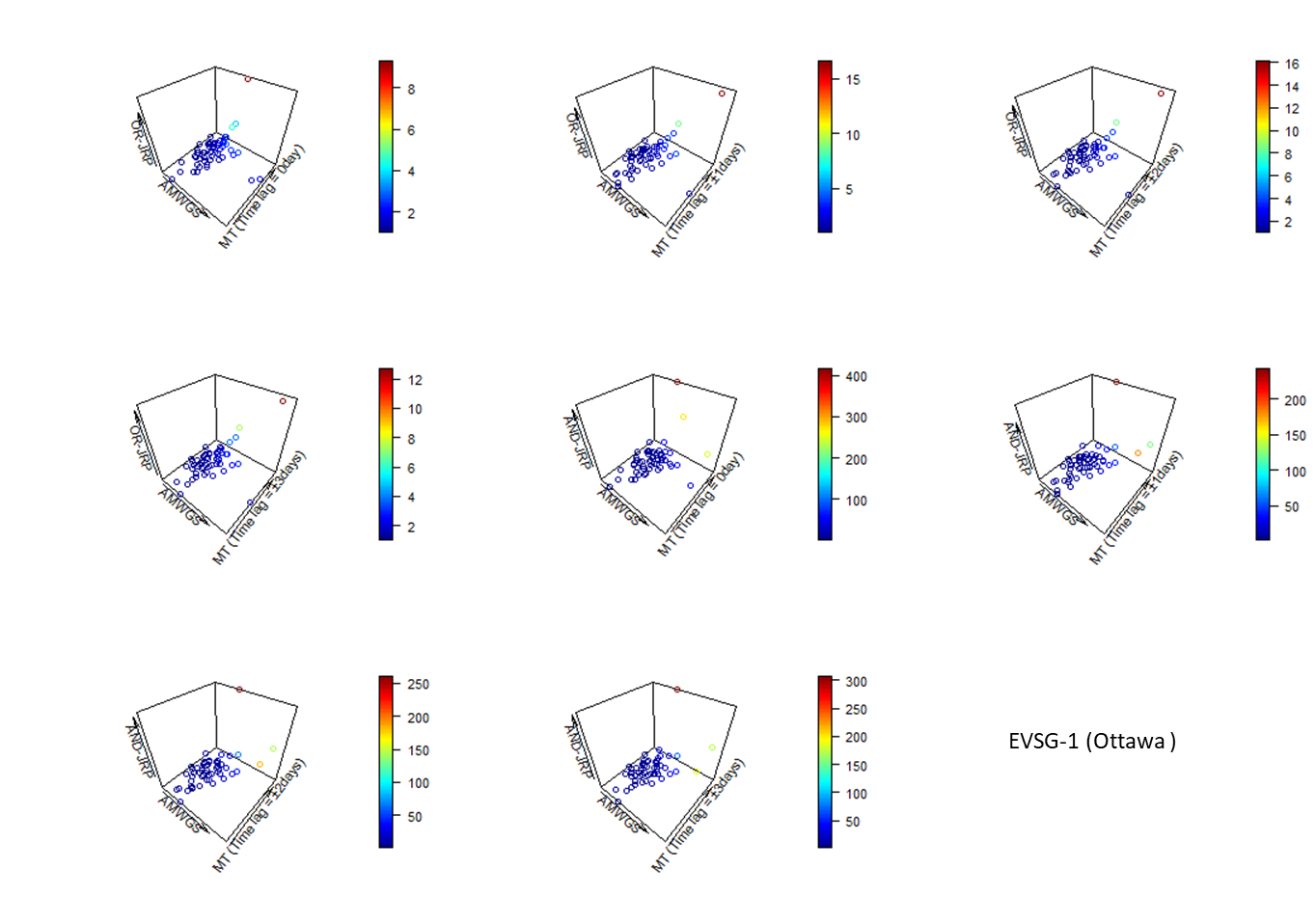
(c)**

**
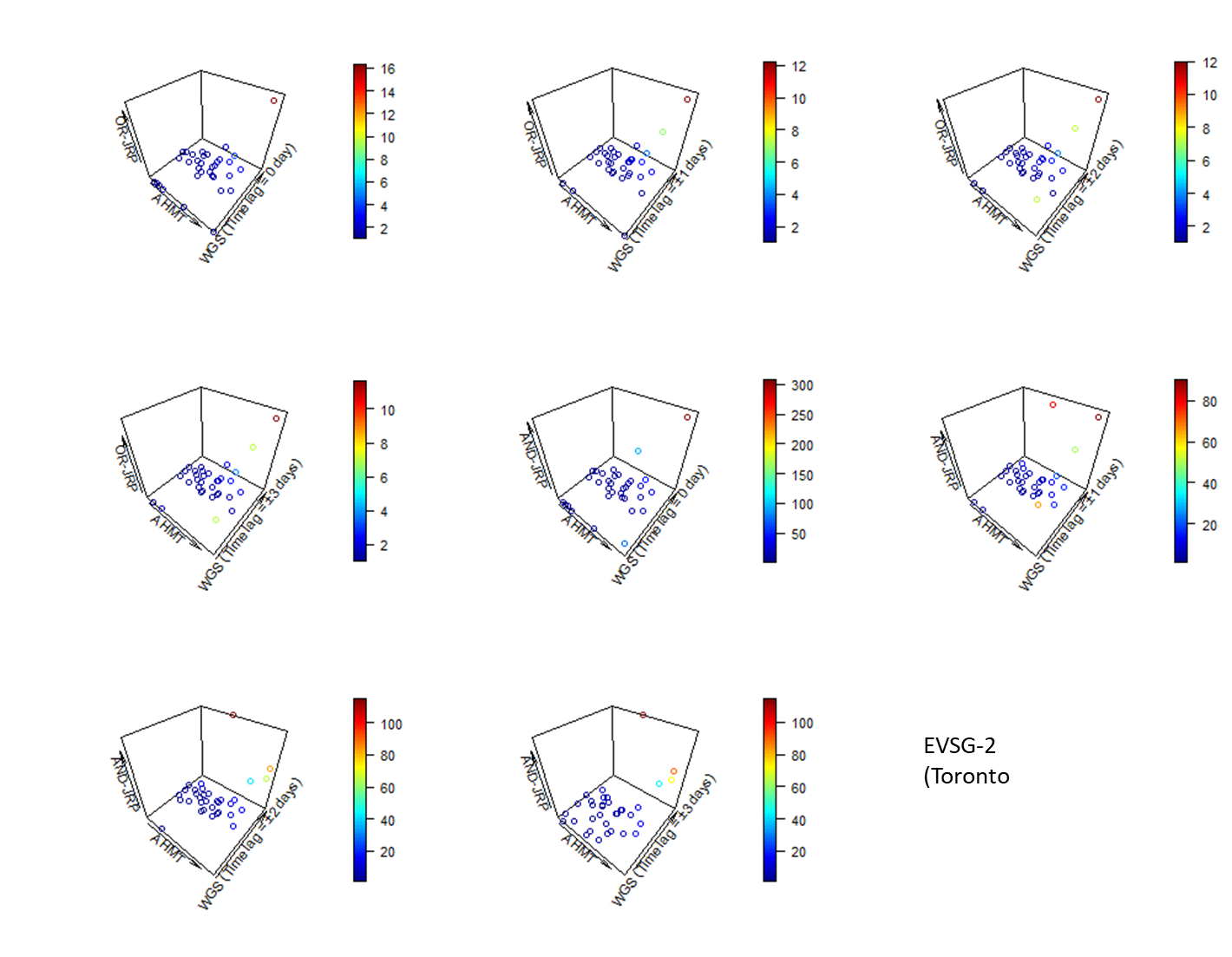
(d)**

**
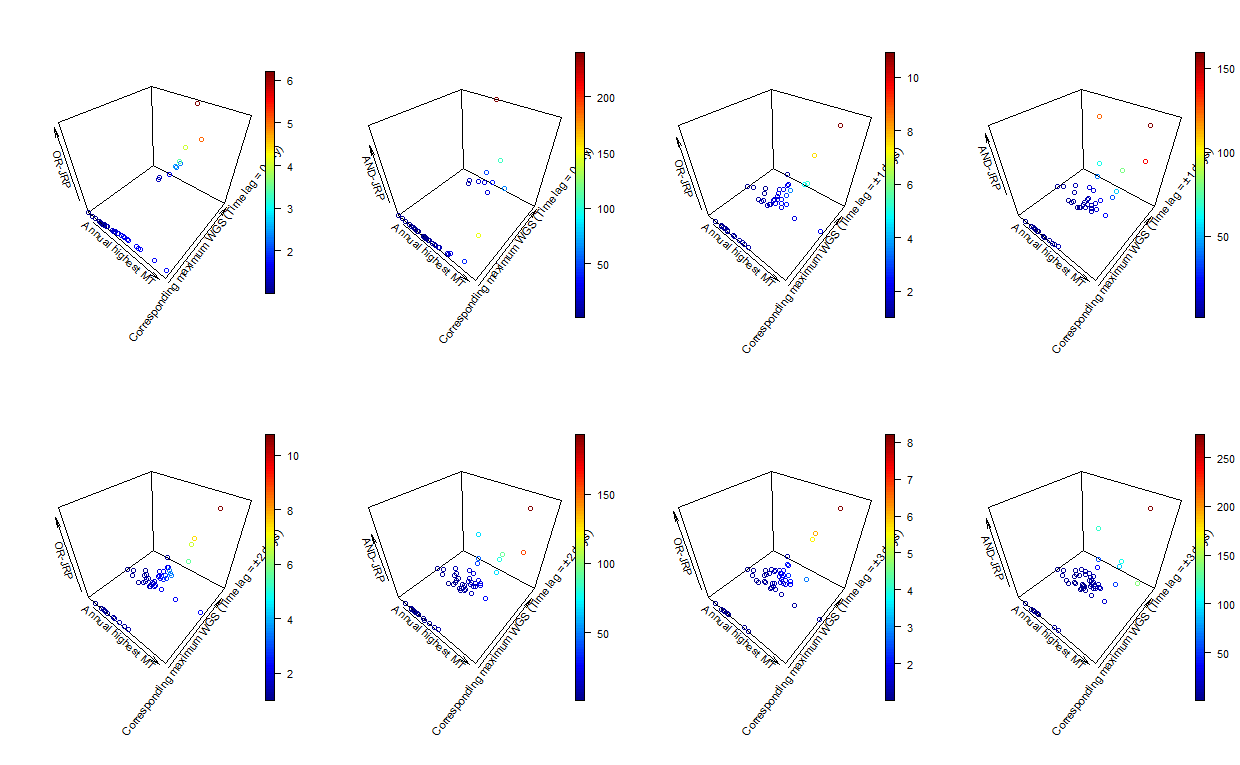
(e)**

**
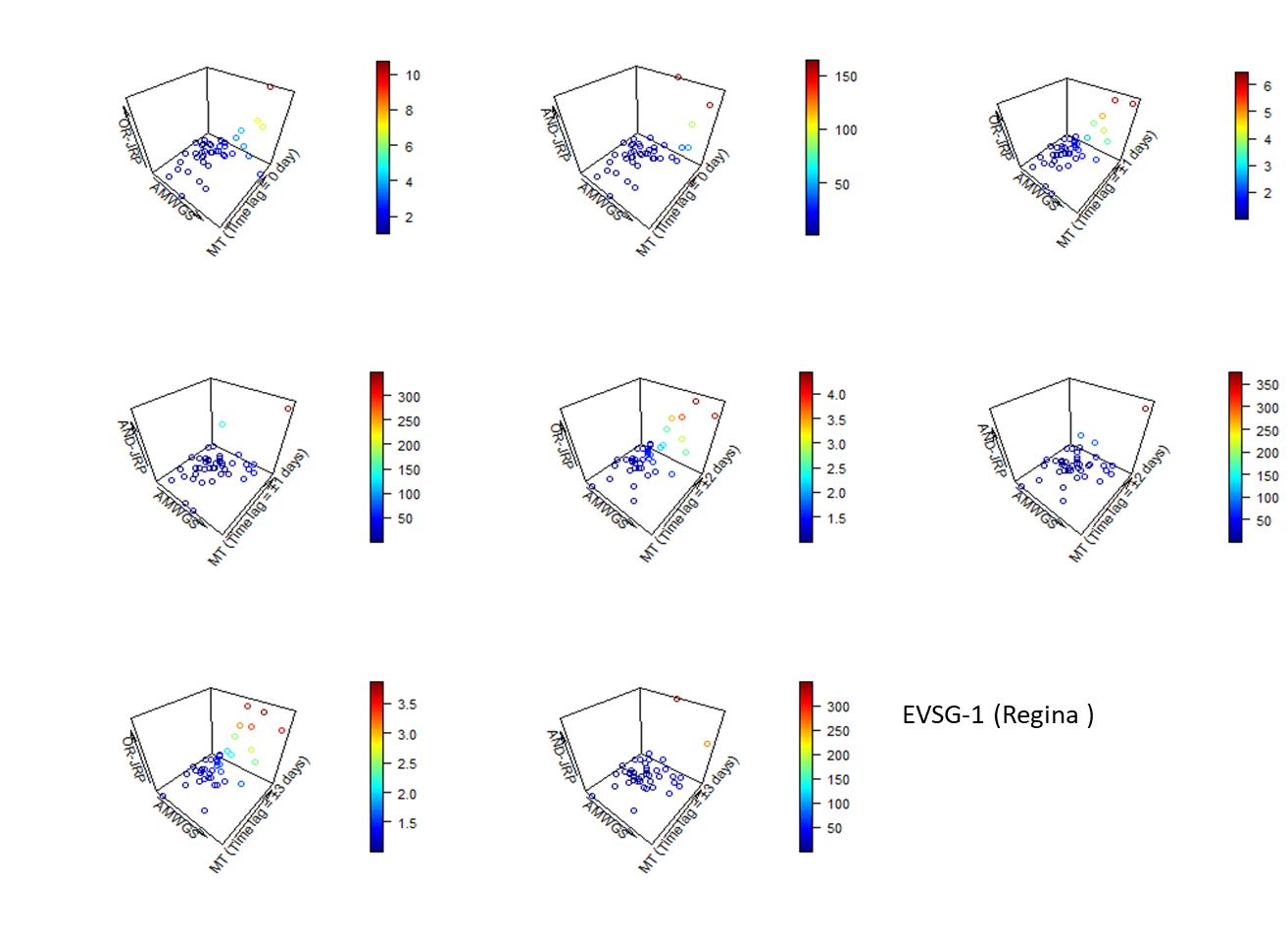
(f-1)**

**
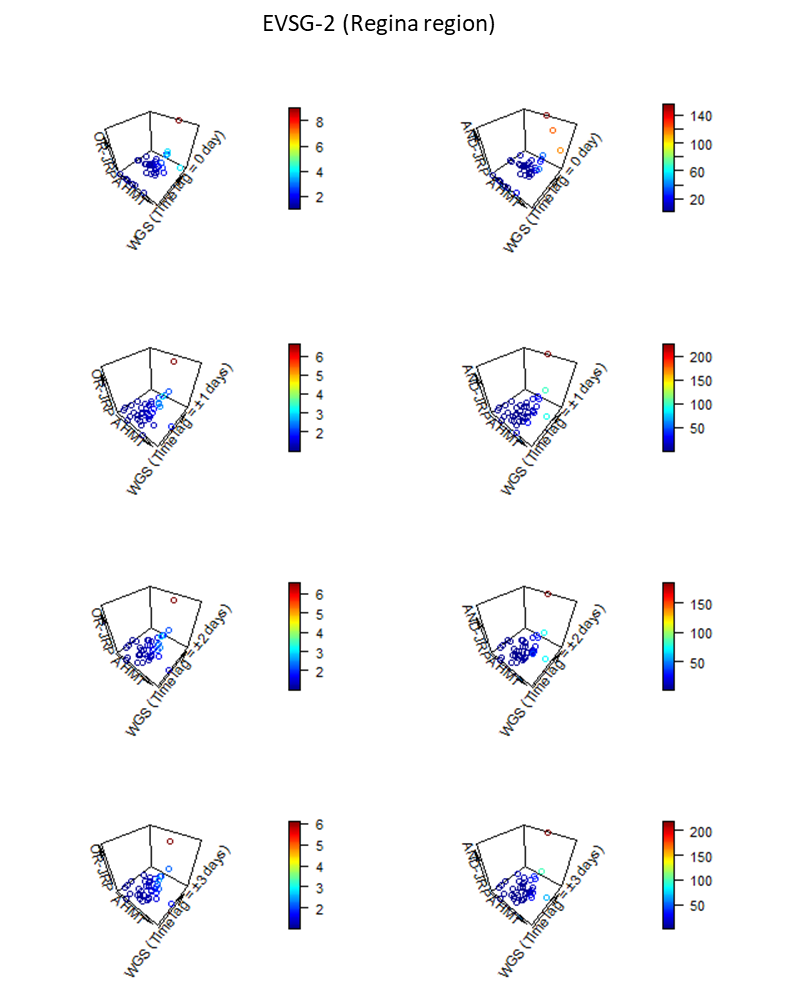
 (f-2)**

**
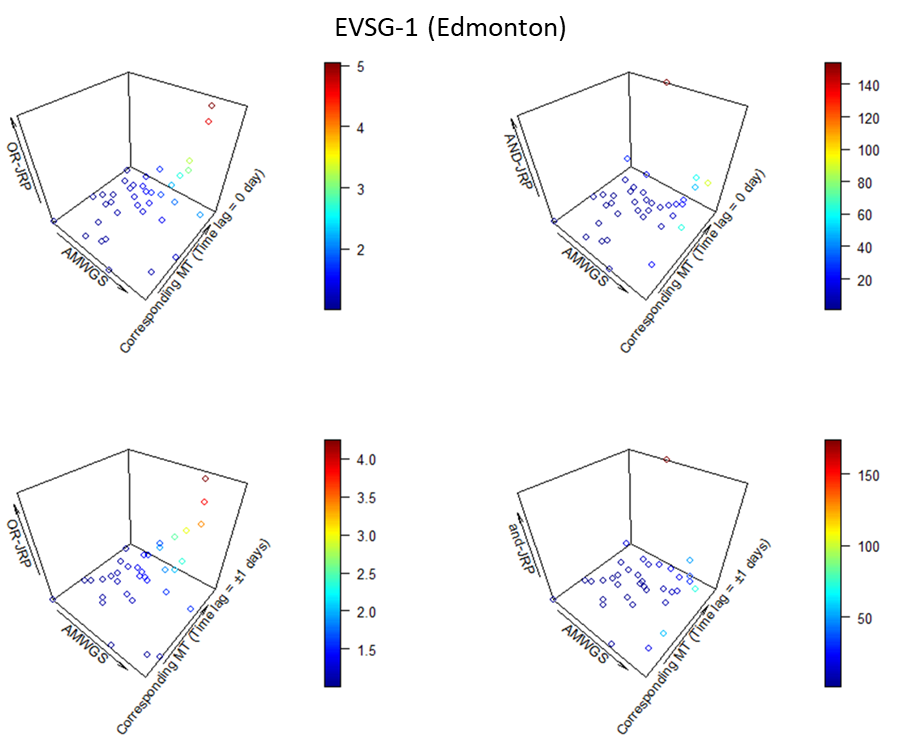
(g)**

**Supplementary SF9**: 3-D scatterplots illustrating the primary joint RPs for bivariate OR and AND hazard scenario for the different possible combination using the historical events (datasets) at the station (a-1, a-2) Montreal (b) Quebec City (c) Ottawa (d) Toronto (e) Vancouver (f-1, f-2) Regina (g) Edmonton


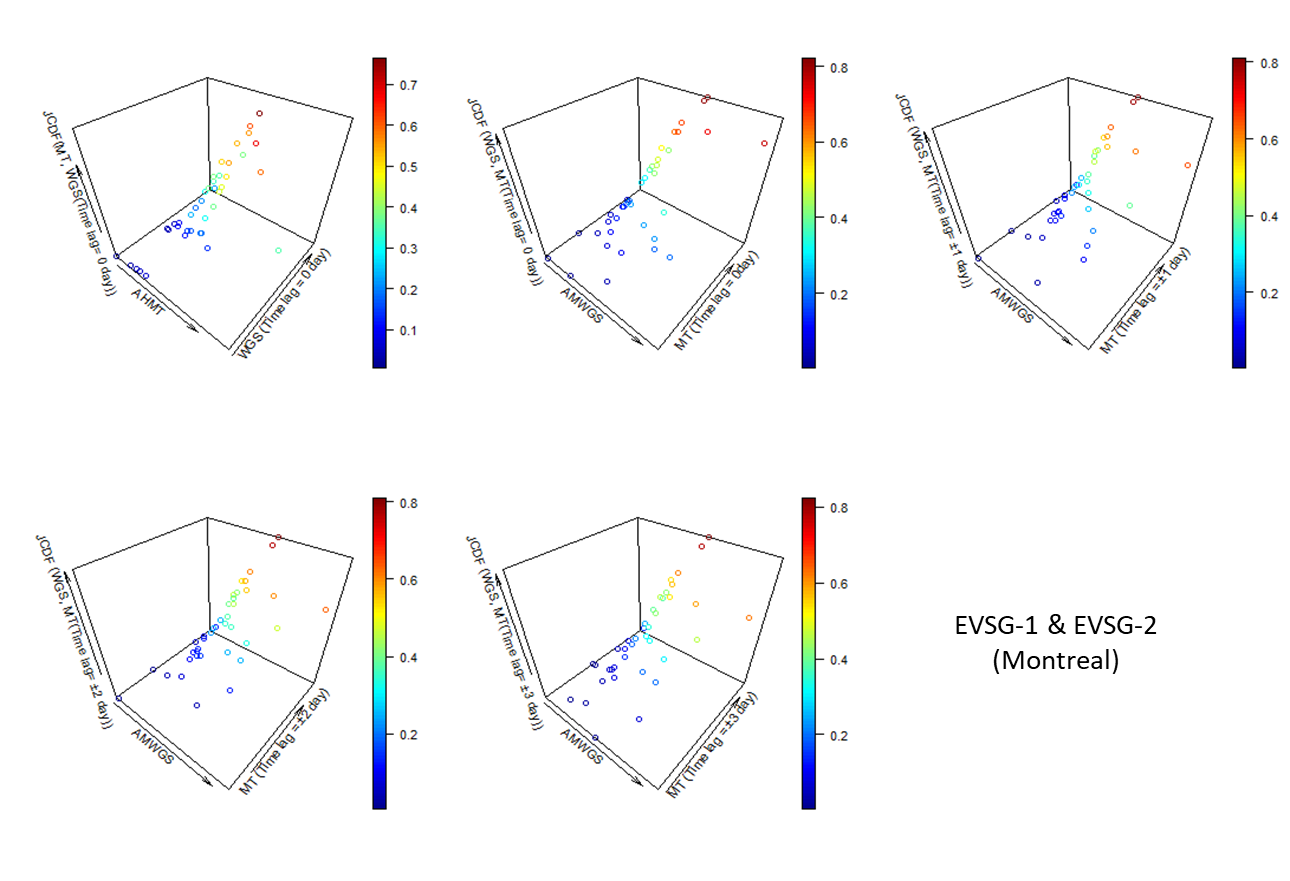
(a)


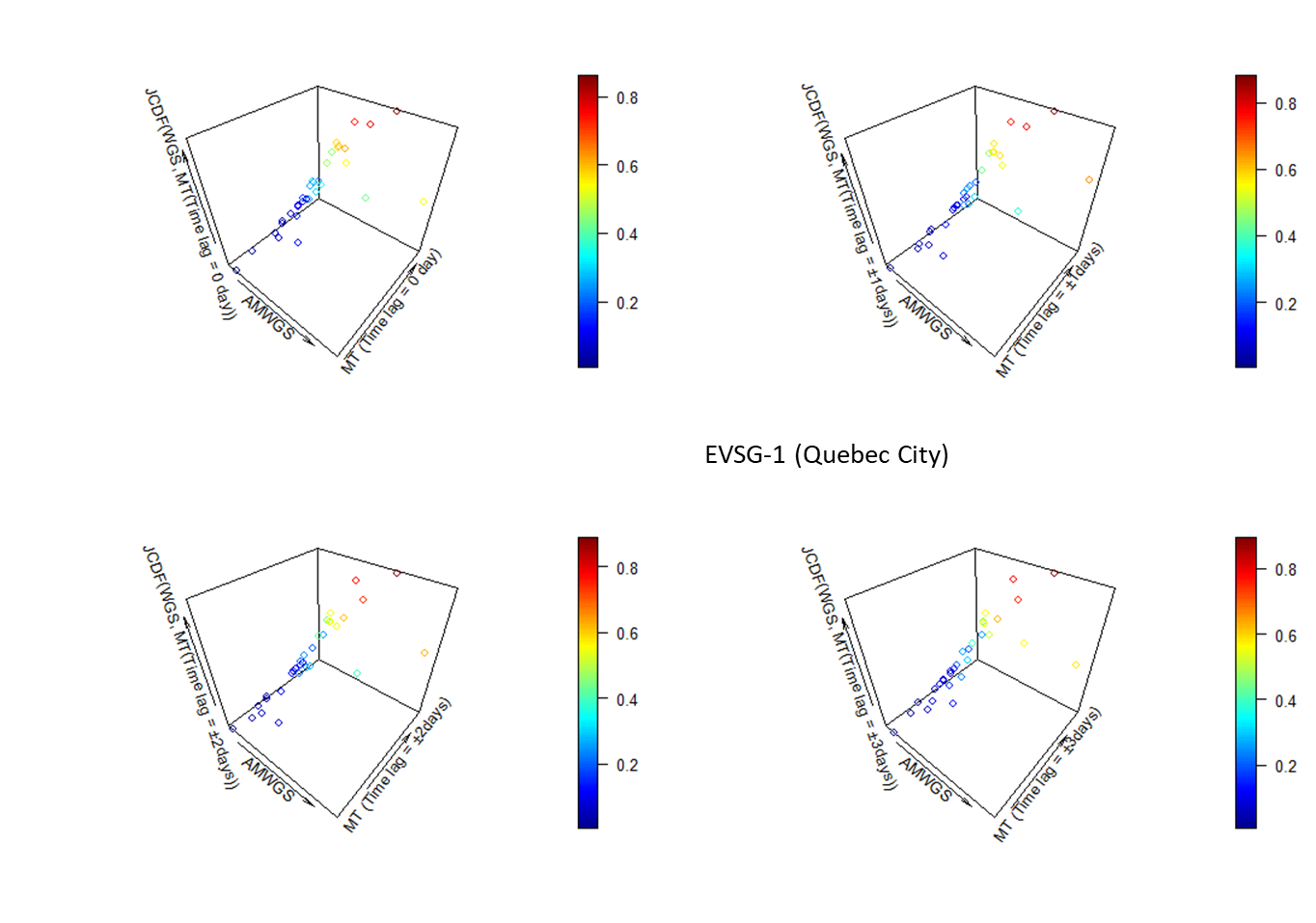
(b)


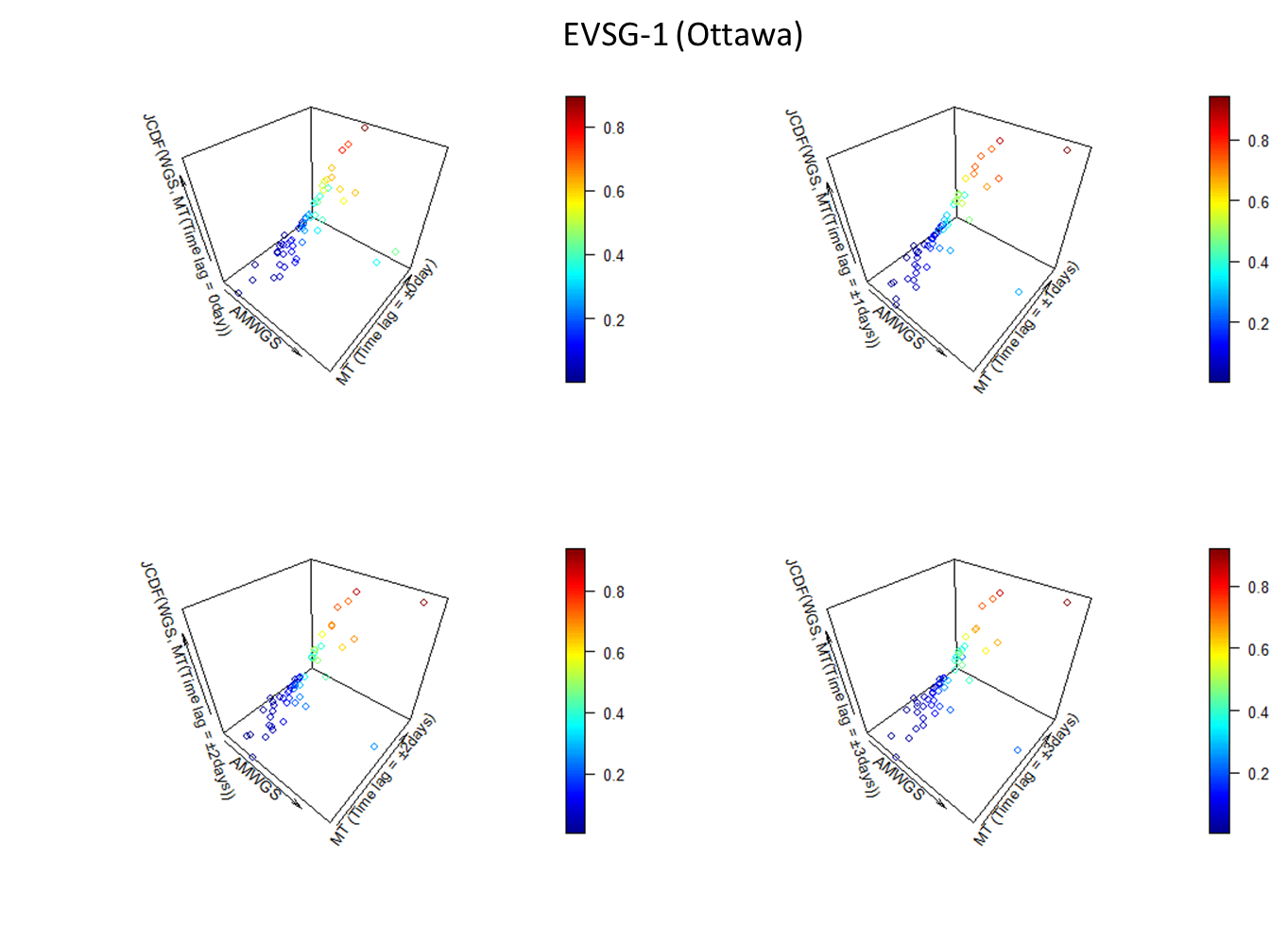
(c)


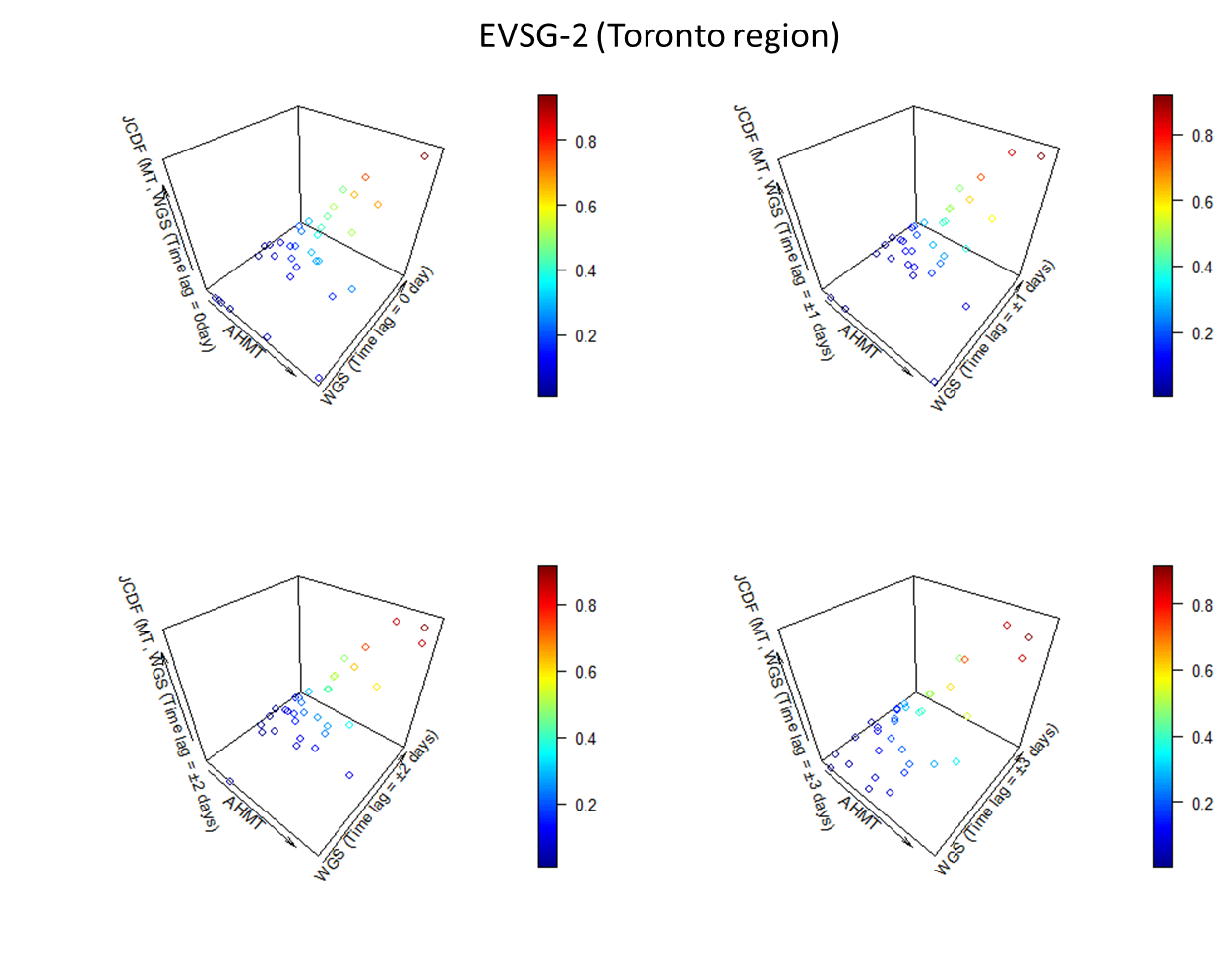
(d)


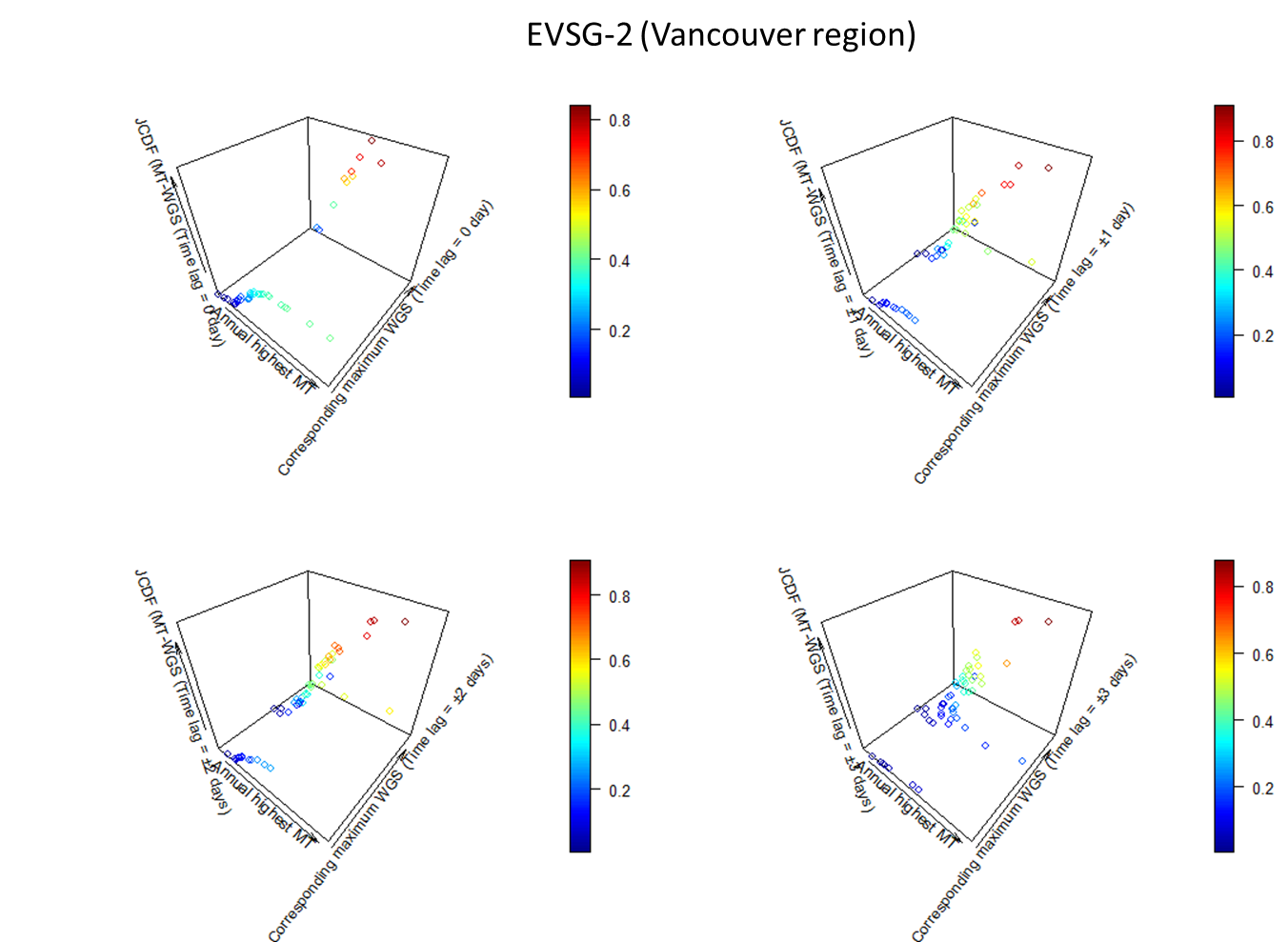


(e)


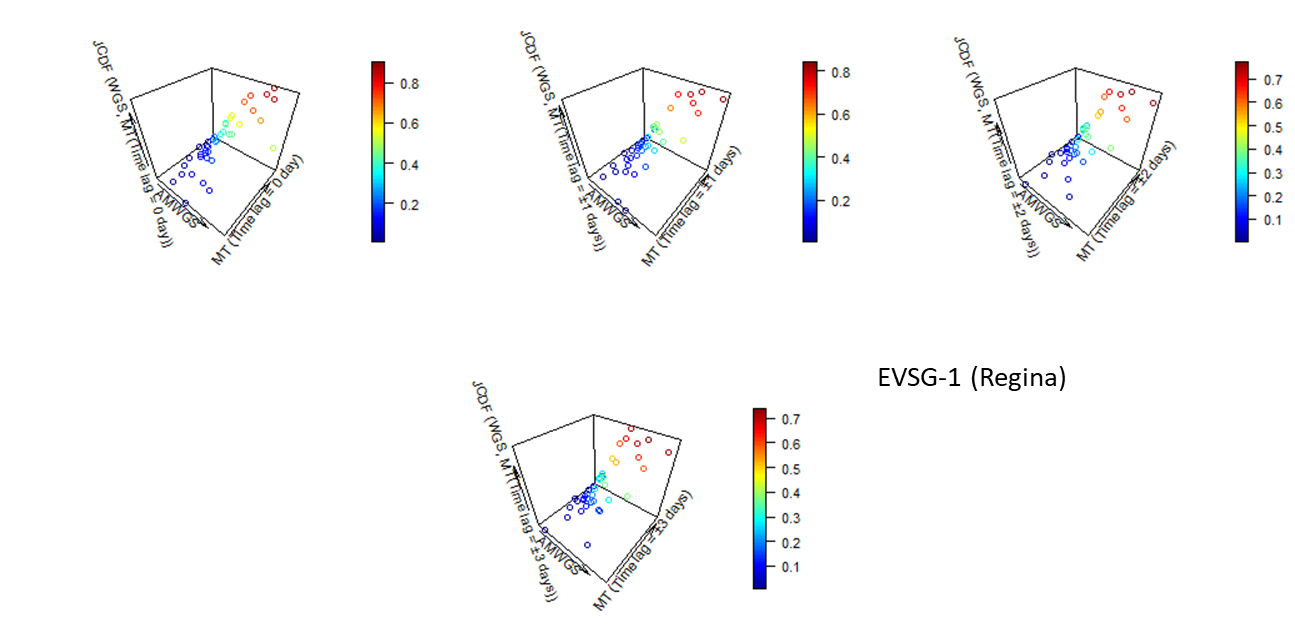
 (f-1


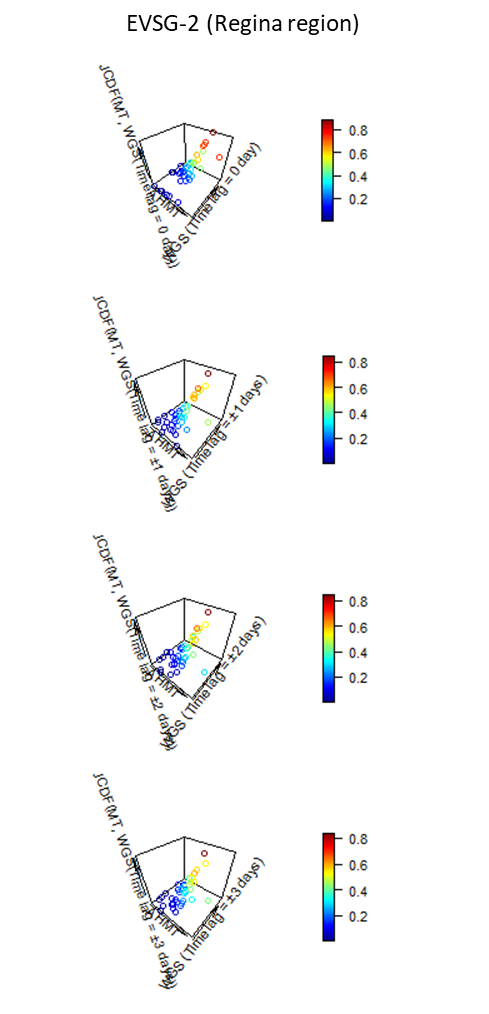
(f-2)


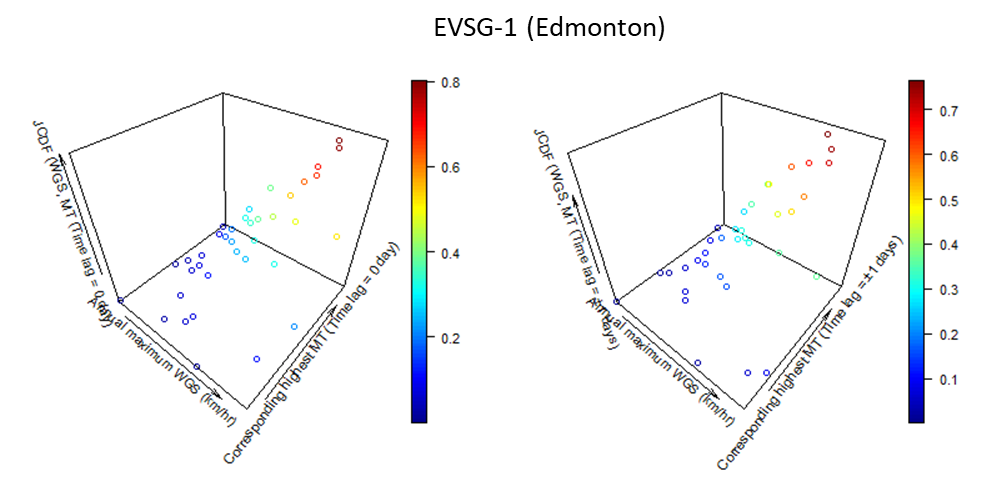


(g)

**Supplementary SF 10** 3-D scatterplots illustrating the Joint CDF (also, called joint nonexceedance probabilities) associated with bivariate extreme pairs of the historical events at the station (a-1, a-2) Montreal (b) Quebec City (c) Ottawa (d) Toronto (e) Vancouver (f-1, f-2) Regina (g) Edmonton


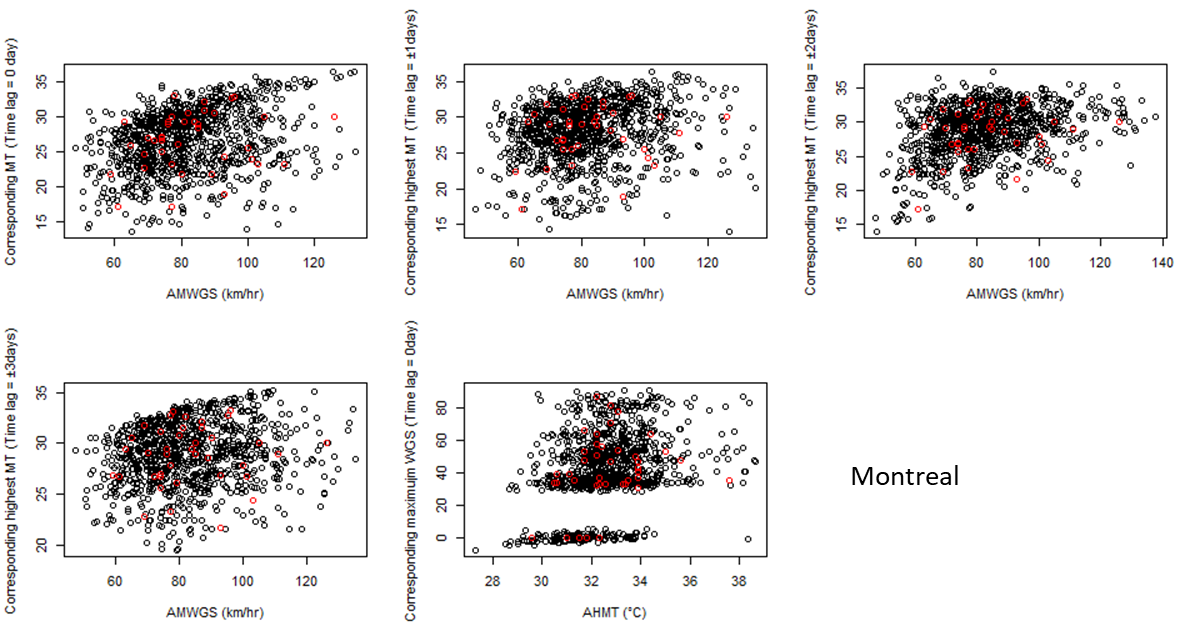
(a)


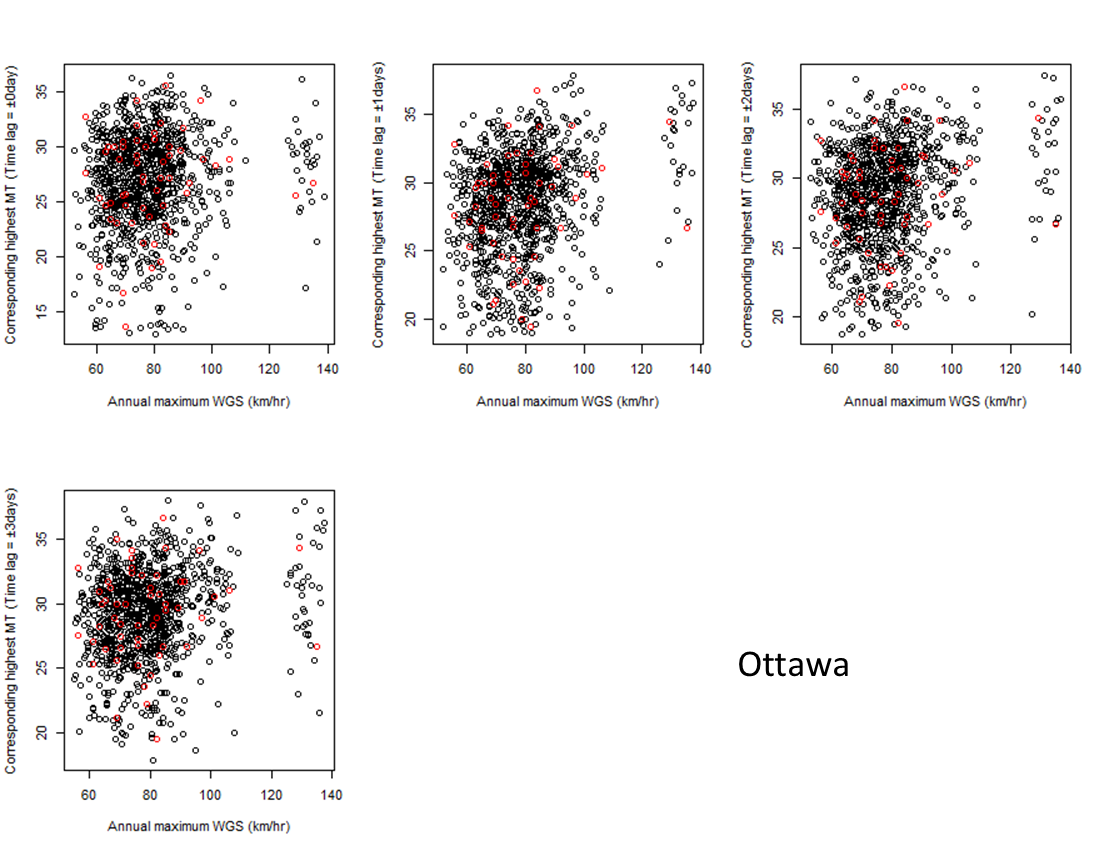


(c)


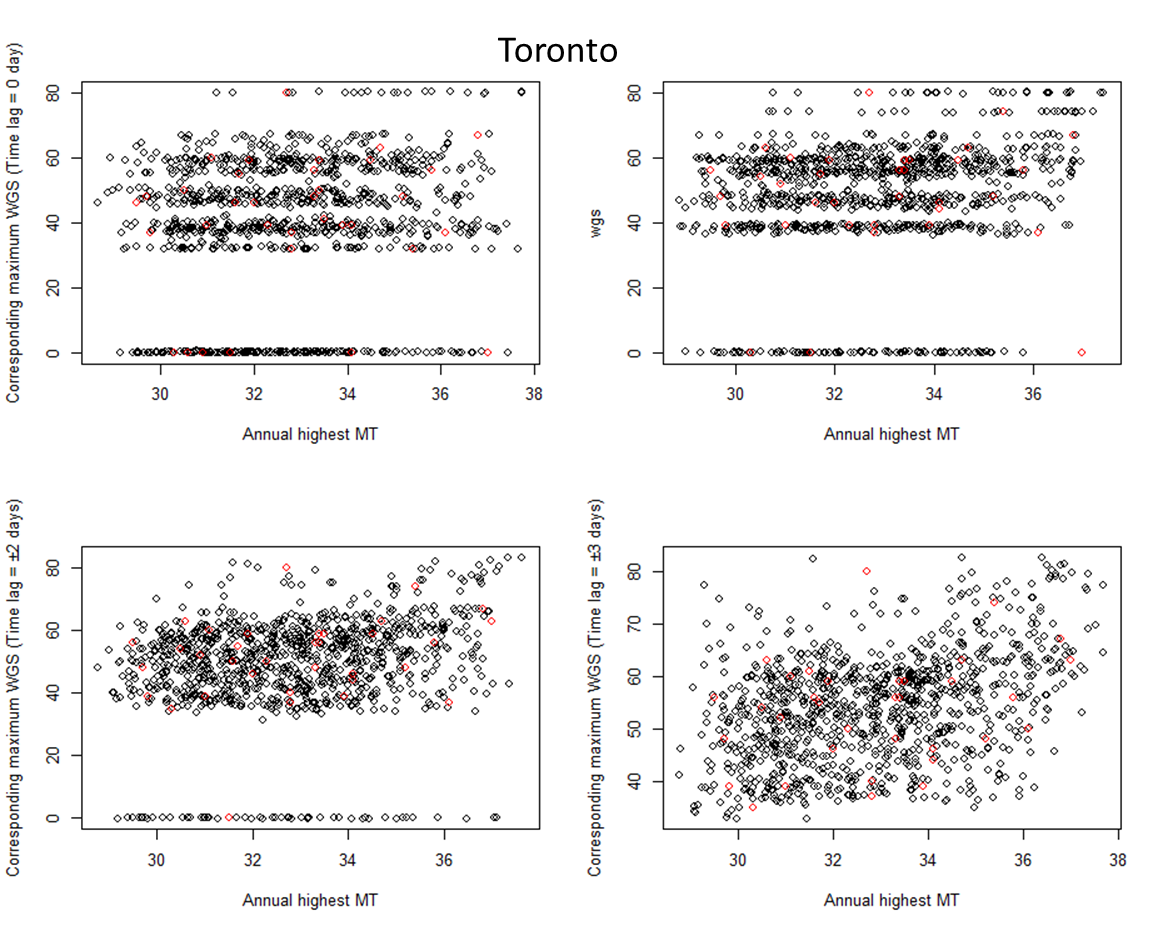


(d)


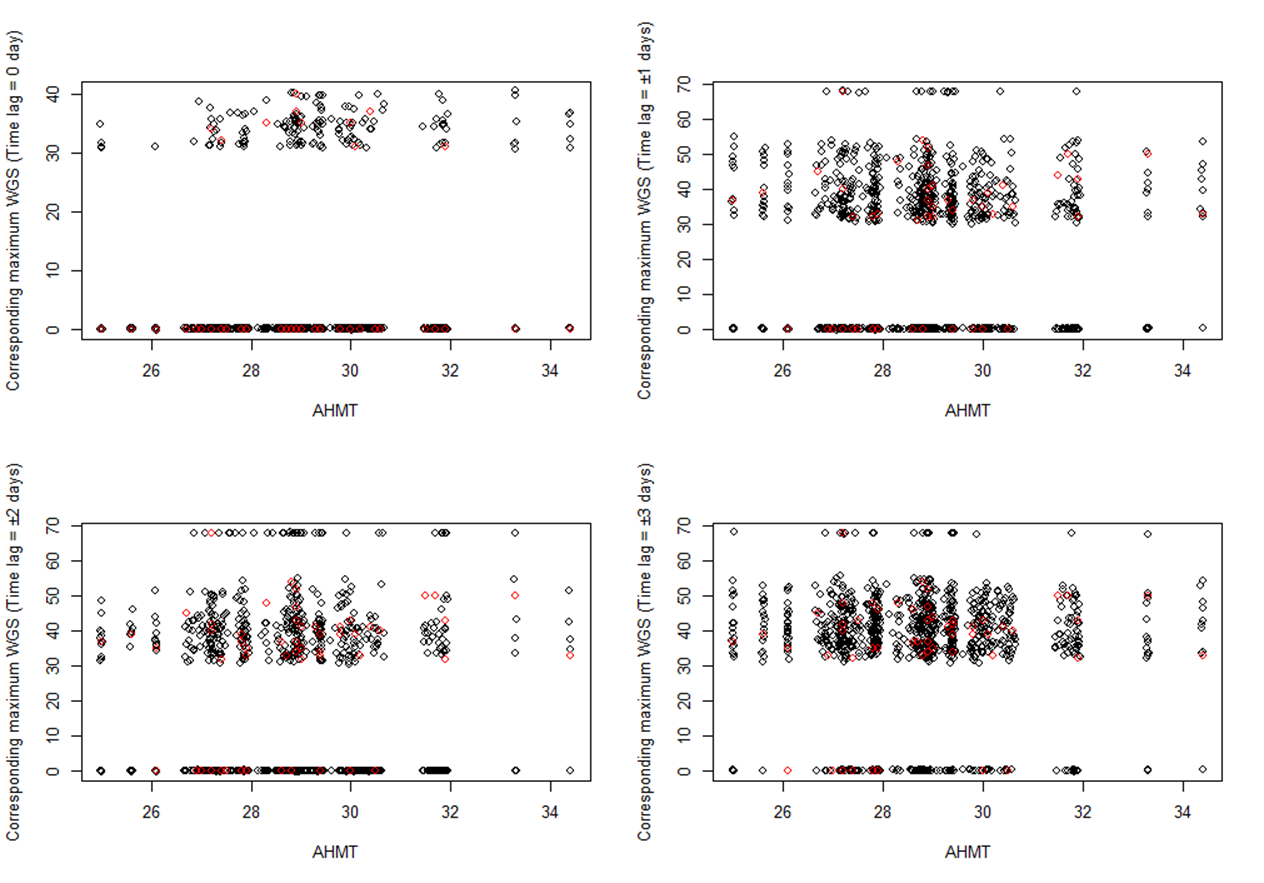
(e)


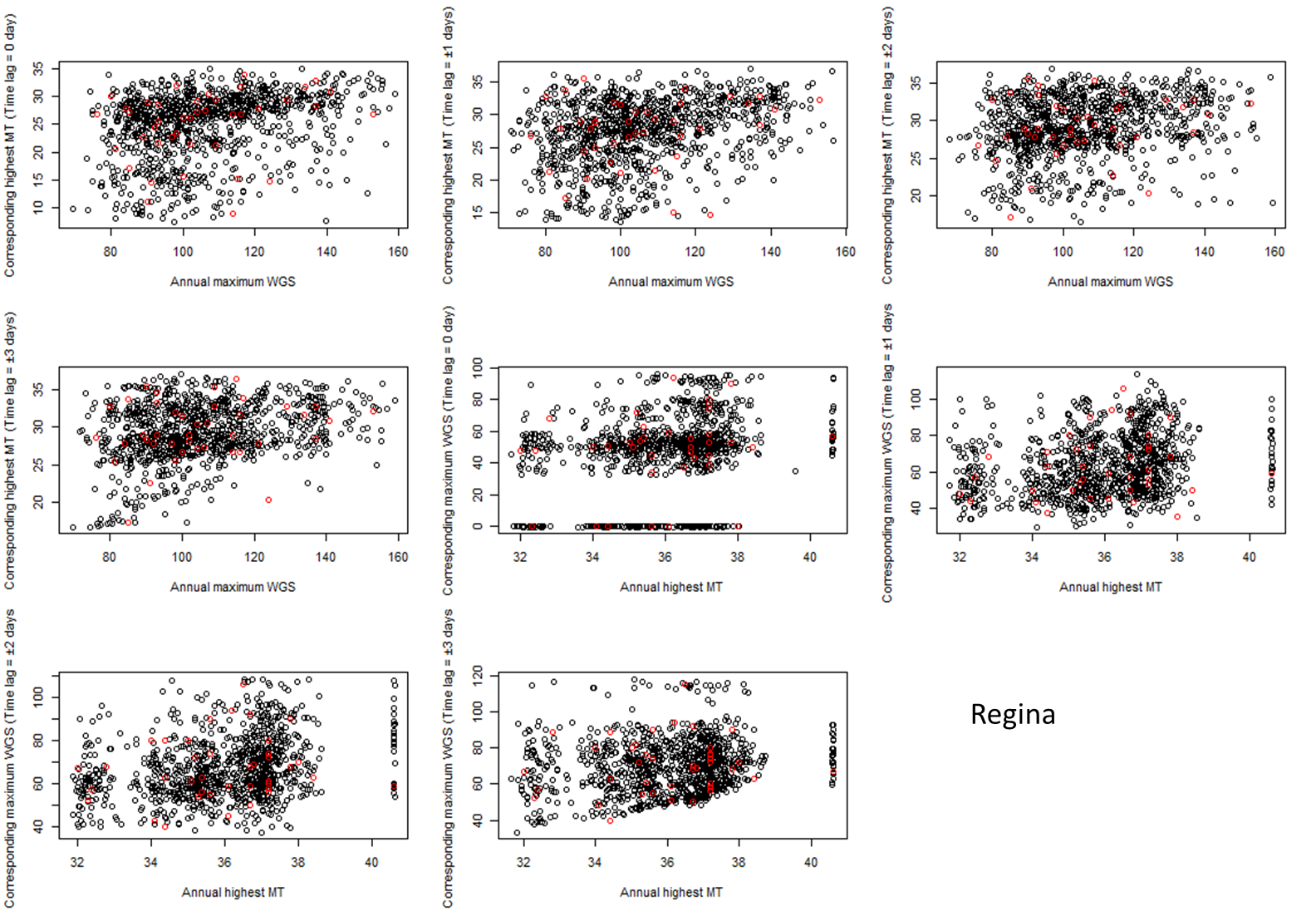
(f)


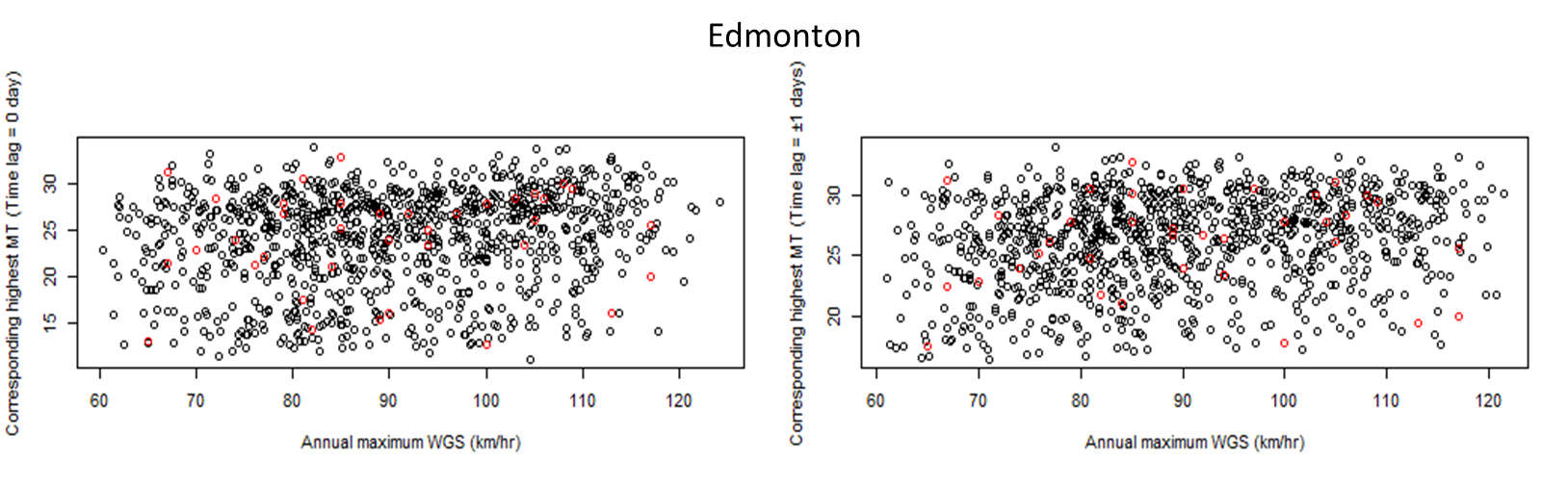
(g)

**Supplementary SF 11** Synthetically generated (sample size, N=1000) bivariate extreme events using the selected semiparametric copula joint density at the station (a-1) Montreal (b) Quebec City (c) Ottawa (d) Toronto (e) Vancouver (f-1, f-2) Regina (g) Edmonton
